# Photocatalytic Addition of *N*‑Oxazolidinone Radicals to Arenes and Heteroarenes in Batch and in Flow Mode

**DOI:** 10.1021/acs.orglett.5c03828

**Published:** 2025-10-23

**Authors:** Sara Ferrario, Sergio Rossi, Niccolò Intini, Julia Bruno-Colmenarez, Marcus Baumann, Maurizio Benaglia

**Affiliations:** † Dipartimento di Chimica, 9304 Università degli Studi di Milano, Via Camillo Golgi 19, 20133 Milano, Italy; ‡ School of Chemistry, 8797 University College Dublin, Science Centre South, D04N2E5 Dublin, Ireland

## Abstract

Recent progress in *N*-centered radical chemistry has paved the way for unprecedented transformations in organic synthesis, particularly through photocatalytic C–N bond formation reactions. Here, unexplored *N*-oxazolidinone radicals are generated through visible light irradiation, enabling a novel, efficient, sustainable, and scalable C–H functionalization process of arenes and heteroarenes. Crucially, the use of continuous flow processing was found to provide further advantages over standard batch conditions, which will enable further applications of this important transformation.

## Graphical abstract

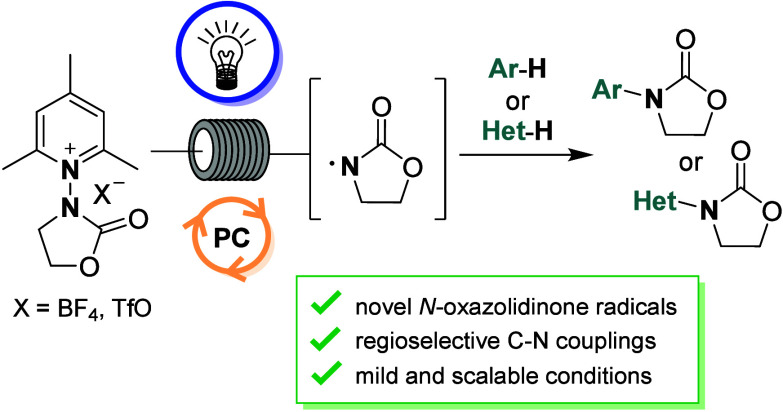

Traditional approaches for the formation of C–N bonds typically involve Pd-catalyzed Buchwald–Hartwig amination or Cu-catalyzed Ullmann-type coupling reactions. However, high temperatures and prefunctionalized coupling partners are often required to successfully provide the desired results.^1,2^ In recent years the chemistry of radicals has been extensively advanced and may offer viable alternatives; for example, *N*-centered radicals (NCRs) can be prepared and introduced onto the appropriate substrates under mild conditions via photoredox catalysis.^1^

In this context, the use of metal complexes, such as ruthenium- or iridium-based compounds with polypyridyl ligands or more green and easily synthesizable organic dyes such as 4CzIPN,^3–5^ can promote C–N bond formation reactions under visible-light irradiation.^6–8^ However, reactions involving nitrogen radicals may proceed not only through photocatalytic cycles but also by perpetuating radical propagation processes.^9^

Among NCRs, amidyl radicals are very versatile species due to their electrophilic chemical behavior and the assumed π-type configuration, resulting from the localization of the unpaired electron in a p orbital (i.e., perpendicular to the nitrogen substituents). Different strategies for the photochemical generation of these reactive species from their precursors are available, such as homolytic cleavage, reductive or oxidative quenching cycles or proton-coupled electron transfer (PCET), all promoted by light.^2,10^ Specifically, a wide variety of *N*-amidyl radical precursors have been recently developed (Figure 1A) involving the cleavage of bonds such as N–O,^11–17^ N–halogen,^18,19^ N–N,^20–23^ and N–H.^24–26^

**Figure 1:**
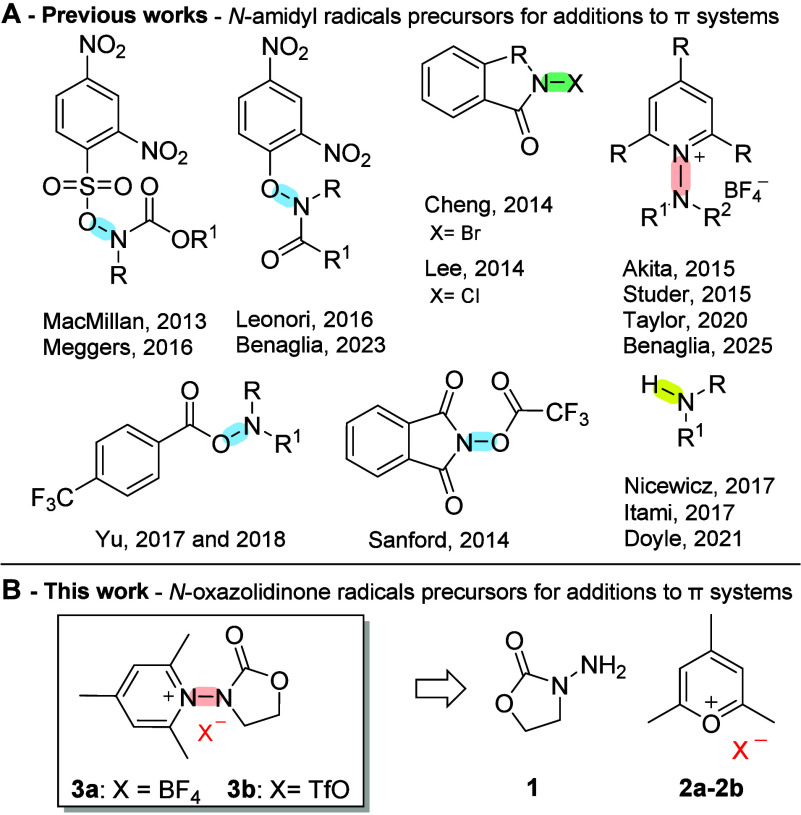
*N*-Amidyl radical precursors.

Furthermore, *N*-centered radicals, when combined with flow chemistry, are also effective in scaled-up processes, overcoming issues related, for example, to the limited light penetration associated with analogous batch processes.^12,14,23,24^ As a result, flow technology offers many clear advantages over batch chemistry in terms of reproducibility, safety, and scale-up.^27–30^

In this context, we envisioned the possibility to exploit visible-light photoredox catalysis for the functionalization of different arenes and heteroarenes, exploiting pyridinium salts, often referred to as Katritzky salts,^31^ as radical precursors for the generation of unprecedented *N*-oxazolidinone radicals (Figure 1B).

First, a straightforward and convenient protocol to prepare two different novel *N*-oxazolidinone radical precursors, 2,4,6-trimethyl-1-(2-oxooxazolidin-3-yl)­pyridinium tetrafluoroborate **3a** and triflate salt **3b**, from **1** and **2a**,**b** was identified. Cyclic voltammogram analysis of pyridinium salts **3a** and **3b** was performed, and the irreversible reduction potentials were determined to be −1.04 and −1.13 V vs Ag/AgCl, respectively (see SI), in agreement with the values of known pyridinium salts.^21,23^

Then, we started to study the photocatalytic activation (by N–N bond cleavage) of compound **3a** (and **3b**) in a model reaction^13,21,23^ under a traditional batch approach (Table 1). The generation and regioselective addition of the *N*-oxazolidinone radical to 1-methylindole **4a** occurs via photoredox catalysis, involving blue light and different set-ups, including cylinder- (setup #1, Figure S1) and plate-based (setup #2, Figure S2) photoreactors. First, various photocatalysts (entries 1–3) and solvents (entries 4–7) were evaluated for the reaction optimization, discovering that reactions performed better in polar solvents such as DMA, DMSO, and DMF. DMA was selected due to its more feasible removal through distillation. Then, the photocatalyst loading (entry 8) and the stoichiometry of the reagents (entries 9 and 12) were evaluated. Different photoreactors were also tested, with the plate setup being identified as the most effective (entry 10). The concentration of the reaction was also varied, showing that a lower concentration of 50 mM gave better outcomes (entry 11). In the end, the reaction time was confirmed to be 16 h, in accordance with the lower yield at 8 h (entry 13). The reaction performed with the pyridinium triflate salt **3b** afforded the desired compound in 70% yield (entry 14), comparable to the yield of tetrafluoroborate salt **3a** (74%, entry 11).

**Table 1:**
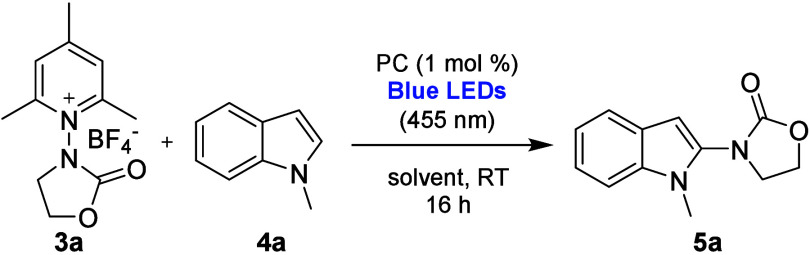
Optimization of Reaction Conditions in Batch

entry	solvent	PC	**3a**:**4a**	setup	conc (M)	yield (%)^a^
1	ACN	Ru(bpy)_3_Cl_2_	1.2:1	#1	0.1	24
2	ACN	4CzIPN	1.2:1	#1	0.1	14
3	ACN	*fac*-Ir(ppy)_3_	1.2:1	#1	0.1	24
4	DCE	*fac*-Ir(ppy)_3_	1.2:1	#1	0.1	37
5	DMF	*fac*-Ir(ppy)_3_	1.2:1	#1	0.1	44
6	DMA	*fac*-Ir(ppy)_3_	1.2:1	#1	0.1	48
7	DMSO	*fac*-Ir(ppy)_3_	1.2:1	#1	0.1	48
8^b^	DMSO	*fac*-Ir(ppy)_3_	1.2:1	#1	0.1	40
9	DMA	*fac*-Ir(ppy)_3_	1:2	#1	0.1	55
10	DMA	*fac*-Ir(ppy)_3_	1:2	#2	0.1	66
11	DMA	*fac*-Ir(ppy)_3_	1:2	#2	0.05	74
12	DMA	*fac*-Ir(ppy)_3_	1:4	#2	0.05	68
13^c^	DMA	*fac*-Ir(ppy)_3_	1:2	#2	0.05	52
14^d^	DMA	*fac*-Ir(ppy)_3_	1:2	#2	0.05	70

a Isolated yields

b PC loading: 2 mol %.

c Reaction time: 8 h.

d Pyridium salt: **3b**.

Taking advantage of continuous flow photochemistry, the C–H functionalization of arenes and heteroarenes with the novel *N*-oxazolidinone radical species was next investigated under flow conditions, using a Vaportec E-Series photochemical reactor (Figure S3). A second round of optimization of the reaction conditions was then necessary, and the stoichiometry, the residence time, as and the nature and the loading of the photocatalyst were investigated. Notably, after a further evaluation of solvents and concentration, a 1:1 mixture of ACN:DMA was identified as good compromise between the experimental setup and reaction conditions, allowing operation with a higher concentration (0.5 M instead of 0.05 M, see SI). From these optimizations, it was found that *fac*-Ir­(ppy)_3_ photocatalyst showed a better chemical efficiency compared to 4CzIPN; by reacting 1 equiv of **3a** with 2 equiv of **4a** dissolved in a 1:1 mixture of ACN:DMA as solvent under blue light irradiation for 15 min as the residence time, it was possible to isolate **5a** in 80% yield (Scheme 1). In the same reaction conditions, it was necessary to employ a 2 mol % loading of 4CzIPN to obtain the desired product **5a** in 65% yield. Nevertheless, since the organic dye 4CzIPN can be considered a greener alternative to iridium-based photocatalysts, it was effectively used in the reaction scope as well (for a series of control experiments of the reaction in batch and in flow, and their comments, see Table S15).

**Scheme 1:**
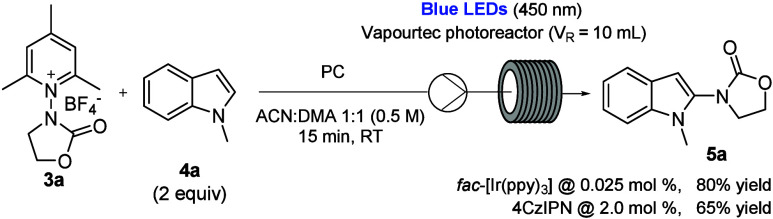
Optimization of Reaction Conditions in Flow

Having identified the optimized conditions, we started to investigate the scope of the reaction with different arenes and heteroarenes, under both batch and flow conditions (Scheme 2).

**Scheme 2:**
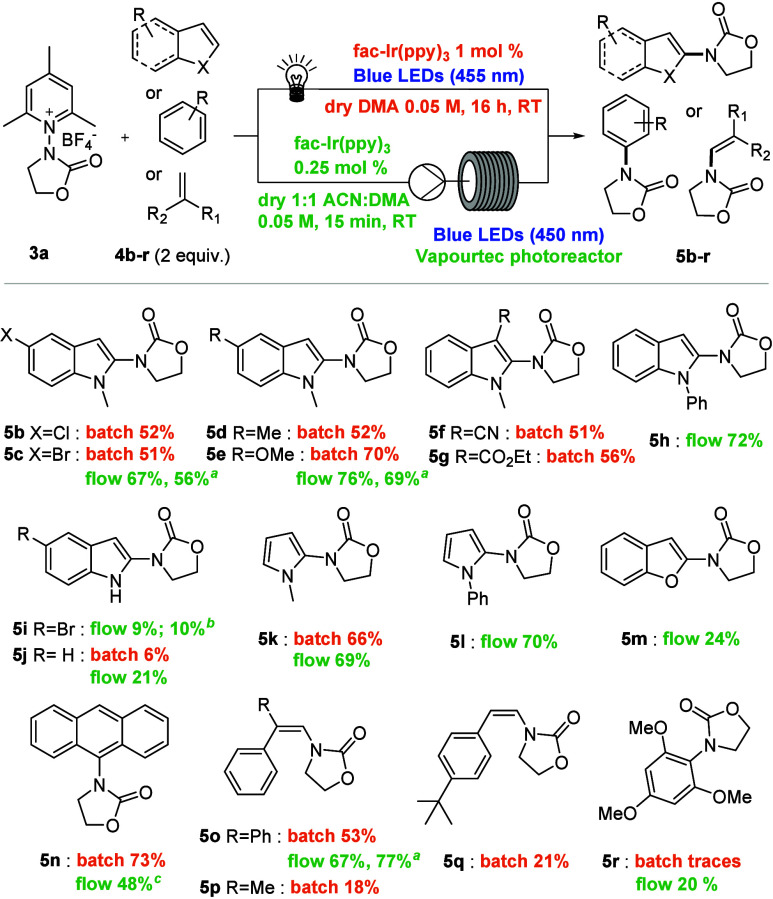
Scope of the Photocatalytic Reaction

a Performed using 4CzIPN with 2 mol % loading.
b 4 equiv of **4i** was used.
c Reaction performed with 0.05 M DMA.

It was found that variously functionalized indoles bearing an alkyl or aryl system with electron-donating or electron-withdrawing groups yielded the products in good to excellent yields (**5b**–**h**). It is worth noting that successful results obtained with 3-substituted indoles may pave the way for the derivatization of tryptophan analogs. The reaction with substituted indoles at position 1 resulted in lower yields (**5i** and **5j**). Notably, heteroarenes, such as functionalized pyrroles and benzofuran, turned out to be suitable substrates for this photocatalytic reaction (**5k**–**m**). The applicability of the method can also be extended to aromatic compounds, such as anthracene (**5n**), functionalized styrenes (**5o**–**q**), and 1,3,5-trimethoxybenzene (**5r**), where the reaction under flow conditions performed much better, likely due to the enhanced light penetration.

The scalability of the process under flow conditions was then evaluated. A gram scale reaction (**3a**, 8 mmol scale) led to the isolation of 1.28 g of product **5a** (yield: 74%, with a productivity of 3.2 g·h^–1^, see SI, section 6). To compare the batch and flow approaches, a kinetic study of the photocatalytic reaction was conducted showing that the performance of the optimized continuous flow process is ca. 4.4 times higher than that in batch (Tables S16–17 and Figure S14).

To better understand the photocatalytic process, single crystal X-ray diffraction structures of the *N*-radical precursors were recorded. The N–N bond length for compounds **3a** (Figure 2A) and **3b** (Figure 2B) was measured to be 1.393 Å. Additionally, the torsional angle around the N–N bonds is 74.93° for tetrafluoroborate salt **3a** and 75.49° for triflate salt **3b**. As a result, the modest energy associated with the labile N–N bond upon photoactivation may be due to the minimal overlap of the π orbitals of the nitrogen atoms.

**Figure 2:**
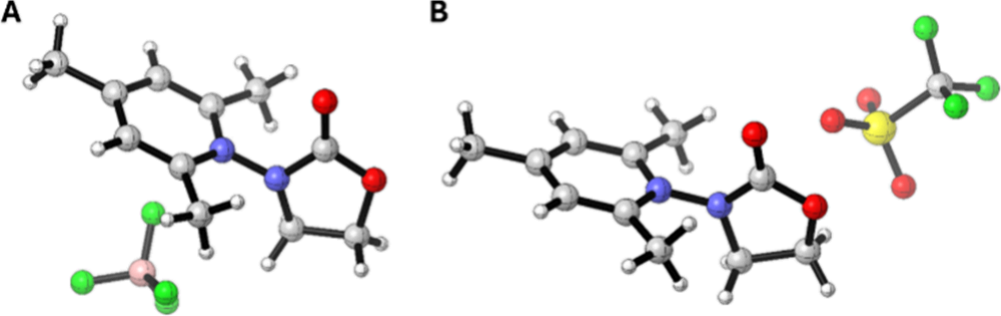
Single crystal structures for **3a** (A) and **3b** (B).

Moreover, it was established that the presence of oxygen is not relevant for the process (SI, section 7), which allowed us to investigate the effect of each reaction component on the photocatalyst photoluminescence. In particular, Stern–Volmer analyses were performed by preparing a solution of each photocatalyst, *fac*-Ir­(ppy)_3_, and 4CzIPN (10^–4^ M) and separately increasing the concentration of the other reaction component, 1-methylindole **4a** or *N*-radical precursor **3a**. The photoluminescence lifetime of both photocatalysts linearly decreased with increasing concentrations of both the reagents, as all the corresponding Stern–Volmer plot graphs confirm (Figures S15–S18), but showed an opposite quenching efficiency trend. In more detail, in accordance with the dynamic (or collisional) quenching of fluorescence described by the Stern–Volmer equation,^32,33^ four Stern–Volmer quenching constants were calculated. Thus, precursor **3a** quenches *fac*-Ir­(ppy)_3_ ten times faster (*K*_D_ = 4.09 × 10^–1^ M^–1^) than 1-methylindole **4a** (K_D_ = 4.04 × 10^–2^ M^–1^) (Figure 3A). On the contrary, the quenching efficiency of the *N*-radical precursor **3a** on 4CzIPN is significantly lower (*K*_D_ = 7.20 × 10^–3^ M^–1^) than that of 1-methylindole **4a** (*K*_D_ = 6.59 × 10^–2^ M^–1^) (Figure 3B). Therefore, two different mechanisms are proposed for the regioselective photocatalytic addition of *N*-oxazolidinone radicals to 1-methylindole (Scheme 3). In the first proposed reaction mechanism (Scheme 3, Pathway A), once a photon is absorbed by the *fac*-tris­(2-phenylpyridine)­iridium­(III) photocatalyst (*E*_red_^0^ Ir­(III)*/Ir­(IV) ∼ −1.73 V vs SCE),^5^ the excited species reduces the pyridinium salt **3a** via single electron transfer (SET). The fragmentation of precursor intermediate **3a′** releases the *N*-oxazolidinone radical and 2,4,6-collidine. The regioselective addition of the radical to the α-position of 1-methylindole provides radical intermediate **5a′**, which is then oxidized via single electron transfer, while the photocatalyst is regenerated. The aminated indole **5a** is formed by deprotonation of **5a″**, since 2,4,6-collidine acts as a base and allows the catalytic cycle.

**Figure 3:**
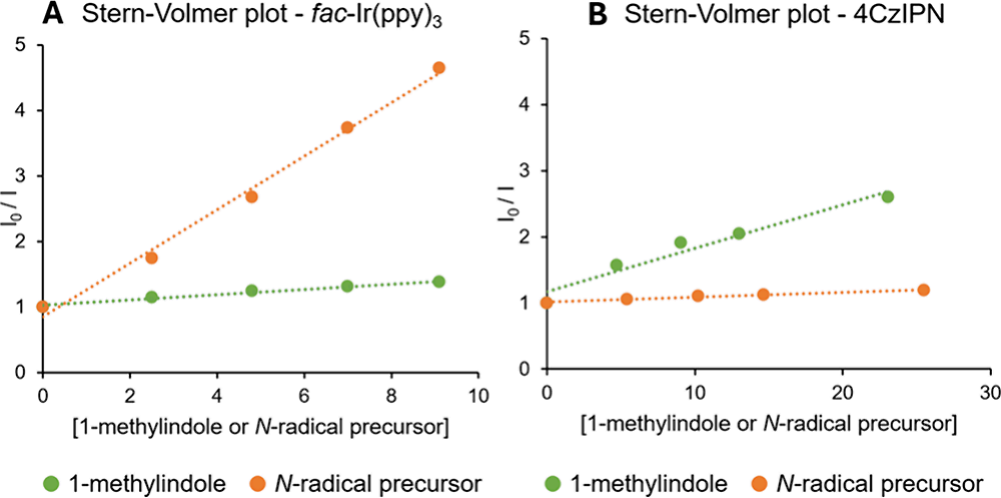
Stern–Volmer plots. Quenching efficiency of precursor **3a** and 1-methylindole **4a** on the fluorescence of *fac*-Ir­(ppy)_3_ (A) and 4CzIPN (B).

**Scheme 3:**
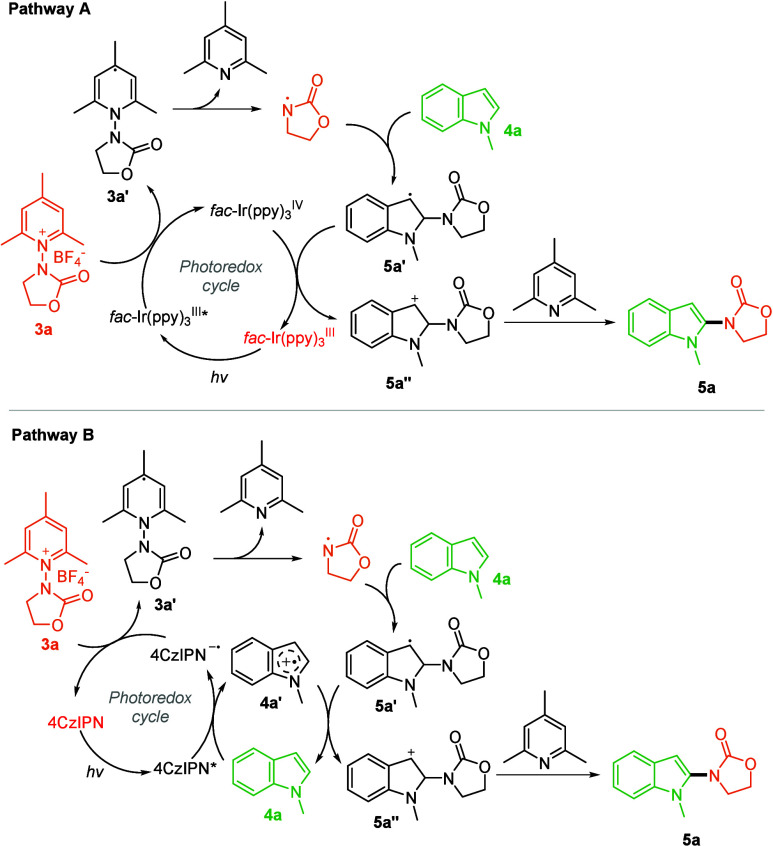
Proposed Reaction Mechanisms

In contrast, in the second proposed mechanism (Scheme 3, Pathway B), the excited 4CzIPN (*E*_red_^*^ = +1.35 V vs SCE)^34^ oxidizes 1-methylindole **4a** via SET.^23^ The 4CzIPN radical anion reduces the *N*-radical precursor **3a**, while the photocatalyst is regenerated. The fragmentation of the precursor intermediate **3a′** provides 2,4,6-collidine and generates the *N*-oxazolidinone radical. The oxazolidinone radical reacts at the α-position of 1-methylindole **4a** to generate the radical intermediate **5a′**, which is subsequently oxidized to **5a″** upon SET. Lastly, the deprotonation of **5a″** yields the desired amidated indole **5a.**

In conclusion, a straightforward protocol was developed for the synthesis of a novel class of *N*-radical precursors. The photocatalytic addition of the *N*-oxazolidinone radical to arenes and heteroarenes was successfully performed under batch and continuous flow conditions, obtaining the desired products in high yields (up to 80%). In addition, the scalability of the process was confirmed by performing a gram-scale reaction, with a good productivity and STY under flow conditions (respectively, 3.2 g·h^–1^ and 200 g·h^–1^·L^–1^). Furthermore, single crystal structures, cyclic voltammetry, and photophysical studies enabled a detailed characterization of the nitrogen radical precursors and a better understanding of the reaction. In the end, two different mechanisms are proposed for the two different photocatalysts used in the transformation.

## Supplementary Material



## Data Availability

The data underlying this study are available in the published article and its Supporting Information and openly available in Dataverse UNIMI at https://doi.org/10.13130/RD_UNIMI/2HB4WC.

## Notes

Experimental procedures, analytical data for reported compounds, X-ray structures, cyclic voltammetry analyses, mechanistic studies, and NMR spectra (PDF)
