# Supplementary material for: Photocatalytic Addition of N‑Oxazolidinone Radicals to Arenes and Heteroarenes in Batch and in Flow Mode
Source: Org Lett. 2025 Oct 23;27(44):12276–80. doi: 10.1021/acs.orglett.5c03828 (PMC12604047; doi:10.1021/acs.orglett.5c03828)
Supplement: Supplementary file 1 [file ol5c03828_si_001.pdf]

Supporting Information

Photocatalytic Addition of *N*-Oxazolidinone Radicals to Arenes and Heteroarenes in Batch and in Flow Mode

Sara Ferrario[1], Sergio Rossi[1], Niccolò Intini[1], Julia Bruno-Colmenarez[2], Marcus Baumann[2], and Maurizio Benaglia*[1]

[1] *Dipartimento di Chimica, Università degli Studi di Milano, Via Camillo Golgi 19, 20133 Milano, Italy.*
[2] *University College Dublin, School of Chemistry, Science Centre South, D04N2E5, Dublin, Ireland.*

Table of Contents

1. **General Information**.. S3
2. **Description of the photoreactors**... S5
    2.1. Cylinder-based photoreactor... S5
    2.2. Plate-based photoreactor.. S5
    2.3. Vapourtec E-series photoreactor.. S6
3. **Synthesis and characterization of the substrates**............................ S8
    3.1. Synthesis of 3-amino-2-oxazolidinone **1**.................................. S8
    3.2. Synthesis of pyrylium salts **2a** and **2b**.................................. S8
    3.3. Synthesis of pyridinium salts **3a** and **3b**................................ S9
    3.4. Single Crystal X-ray diffraction structures of **3a** and **3b**.............. S10
    3.5. Cyclic Voltammetry analyses of **3a** and **3b**............................ S14
    3.6. Synthesis of heteroarenes substrates for the scope **4b-d**, **4f-h**, **4l**............ S16
4. **Optimization of the photocatalytic reaction**................................. S19
    4.1. Screening of the conditions in batch...................................... S20
    4.2. Screening of the conditions in flow.. S23
    4.3. Control experiments... S27
5. **Reaction scope**.. S28
    5.1. Substrate scope in batch and in flow...................................... S28
    5.2. Unsuccessful results.. S36
6. **Gram-scale reaction under continuous flow conditions**.................... S37
    6.1. Comparison between batch and flow approaches.......................... S38
7. **Stability studies**.. S40
8. **Mechanistic investigations**... S41
    8.1. Stern-Volmer studies on *fac*-[Ir(ppy)$_3$] fluorescence..................... S41
    8.2. Stern-Volmer studies on 4CzIPN fluorescence.............................. S43
    8.3. Comparison between *fac*-[Ir(ppy)$_3$] and 4CzIPN........................... S45
9. **NMR spectra**.. S46
10. **References**.. S103

1. General information

Reagents were purchased at the highest commercial quality and used as received. Whenever necessary, 1-methylindole was purify by distillation and stored under nitrogen atmosphere, while other starting materials were synthesized according to literature procedures. The metal-based photocatalysts, Tris(2,2′-bipyridyl) dichlororuthenium (II) hexahydrate ($Ru(bpy)_3Cl_2$) and Tris(2-phenylpyridine)iridium(III) (*fac*-$Ir(ppy)_3$) were commercially available and used without further purification. In contrast, 1,2,3,5-tetrakis(carbazol-9-yl)-4,6-dicyanobenzene (4CzIPN) was synthesized according to a reported protocol.[1] Dry solvents were purchased with AcroSeal packaging and used without further purifications.

If not otherwise stated, reactions were carried out under a positive pressure of nitrogen (5 cm of mercury, or with a spring-loaded silicon oil bubbler set to 100 mbar) and dry solvents were used. Reactions were monitored by thin layer chromatography (TLC) on Macherey-Nagel pre-coated silica gel plates (0.25 mm) and visualized by UV irradiation at 254 nm. Whenever necessary, a vanillin solution (1 g of vanillin, 170 mL of methanol, 10 mL of sulfuric acid 98 %, 10 mL of acetic acid) or a permanganic solution (3 g potassium permanganate, 20 g sodium carbonate, 300 mL of deionized water, sodium hydroxide 5 % solution) were used as stains for developing TLC plates. Flash chromatography was performed both on standard flash column chromatography on Merck silica gel 60 (particle size: 0.04–0.063 mm) and using the automated PuriFlash® Flash Purification System PF-XS 520 (porosity: 60 Ä, particle size: 50 μm). Petroleum ether (PE), hexane, cyclohexane, pentane, ethyl acetate (EtOAc), dichloromethane (DCM), methanol (MeOH), diethyl ether (Et_2O) were used as standard eluent solvents.

^{1}H NMR ^{13}C NMR and ^{19}F NMR spectra were recorded at 25 °C on Bruker Avance spectrometers (300 MHz, 75 MHz, 282 MHz) or JEOL/Varian VNMRS (400 MHz, 101 MHz, 376 MHz). Deuterated solvents acquired from Sigma-Aldrich were used as supplied. The spectra were recorded in ppm using the solvent peak as a reference for ^{1}H and ^{13}C NMR spectra (7.26, 77.16 for $CDCl_3$; 1.94, 1.32 and 118.26 for CD_3CN; 2.50, 39.5 for DMSO-*d6*; 3.31, 49.0 for MeOD-*d4*). ^{1}H NMR data are reported as follows: chemical shift (ppm), multiplicity (s = singlet, br. s. = broad singlet, d = doublet, t = triplet, q = quartet, quint = quintet, sext = sextet, hept = heptet, dd = doublet of doublets, ddd = doublet of doublets of doublets, td = triplet of doublets, qd = quartet of doublets, m = multiplet), coupling constants (Hz), and numbers of protons. ^{19}F and ^{13}C NMR data are reported as follows: chemical shift (ppm). Structural assignments were made with additional information from gCOSY, gHSQC, and gHMBC experiments.

High-resolution mass spectra (HRMS) were obtained from: the central analytic mass spectrometry facilities of School of Chemistry, University College Dublin, on an Agilent 6546 micromass LCT orthogonal time of flight mass spectrometer and quadrupole time-of-flight mass spectrometer with leucine-enkephalin (Tyr-Gly-Phe-Leu) as internal lock mass; the Unitech COSPECT centre, University of Milan, on a Synapt G2-Si HDMS (Waters) using an Acquity UPLC I-Class photodiode array (PDA) detector. The samples were ionized in positive ion mode using a ESI, ESCi or APCI ionization sources.

Single crystal X-Ray diffractions (SCXRD) were obtained from the X-Ray diffraction laboratory of School of Chemistry, University College Dublin, using a Rigaku SuperNova single-crystal four-circle diffractometer. The instrument was equipped with a micro-focus sealed X-ray tube, a mirror monochromator, and an Atlas detector. CuKα radiation (λ = 1.54184 Å) was employed the datasets.

IR spectra were obtained by the use of a Platinum spectrometer (near, ATR sampling, Bruker, Billerica, MA, USA or Jasco FT/IR-4X1-typeA). The intensities of the characteristic signals are reported as weak (w, <20% of tallest signal), medium (m, 21-70% of tallest signal) or strong (s, >71% of tallest signal).

Stern-Volmer analyses were carried out with a Shimadzu RF-6000 Spectro Fluorophotometer, using a quartz cuvette with a path length of 1 cm.

Cyclic voltammetry analyses were carried out by using a Metrohm Autolab Series Potentiostat/Galvanostat Electrochemical System, using a glassy carbon as working electrode, a platinum foil as counter electrode and a silver wire as pseudo reference electrode. The measurements were carried out under static argon atmosphere, in dry DMA, using tetrabutylammonium tetrafluoroborate (0.1 M) as supporting electrolyte. The scan rate was 100 $mV \cdot s^{-1}$.. The potentials were determined versus Ag/AgCl (LiCl 2 M in EtOH).

3D-printed photoreactors were obtained by a Form 3 3D-printer (Formlabs), using Clear V4 or Draft V2 resins. Flow reactions were performed using a Vapourtec E-Series UV-150 photoflow reactor, equipped with high-power LEDs (450 nm, 24 W) and a fluoropolymer tubing as reactor coil of 10 mL volume.

2. Description of the photoreactors

2.1. Cylinder-based photoreactor

The cylinder-based photoreactor (**Figure S1**) is a 3D-printed, double jacketed cylindrical reaction vessel (h = 11 cm; internal diameter = 6.5 cm). A 100 cm long strip of blue LEDs (540 mW/cm^2, 24 V) has been sticked on the internal wall of the vessel and the vial of the reaction is placed at the centre of the vessel (distance from the LEDs = 3 cm). In the external jacket, water flows to cool down the reaction vessel, it is also possible to use compressed air to further cool the reaction vial.
LEDs specifications: Ledpoint Blue LED 2835 120 led/m 24 V with a self-adhesive tape that holds the strip light safely and securely to the photoreactor support. The LEDs wavelength emission profile together with their specific light intensity (expressed as mW/cm^2) have been determined using a compact CCD spectrometer (model CCS200/M) connected to a multimode optical fibre, purchased by Thorlabs. Blue LEDs employed are characterized by an almost monochromatic emission profile, showing a maximum of intensity at ca. 459 nm. The light power intensity was thus checked using a Thorlabs PM200 power meter equipped with a S130VC power head with a Si detector. The measured light intensities resulted to be I = 540.2 mW/cm^2.

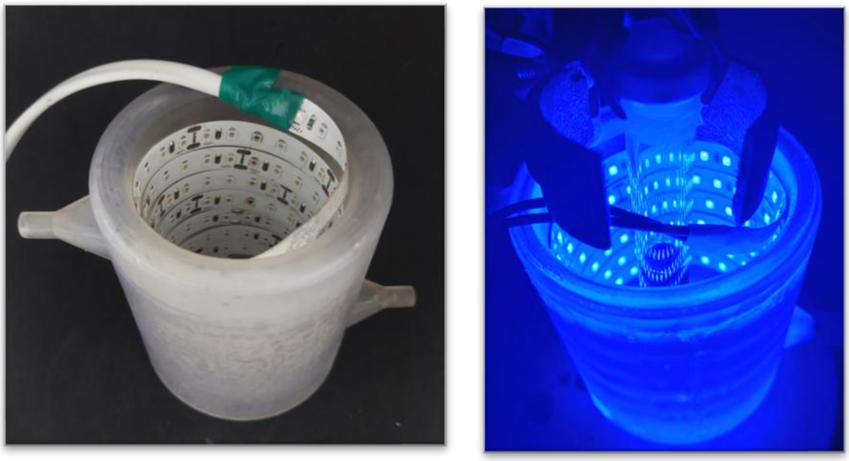

Figure S1: Cylinder-based photoreactor

2.2. Plate-based photoreactor

The plate-based photoreactor (**Figure S2**) is a CNC machined reactor, made of stainless steel and billet. The single LEDs are fixed to an aluminium heatsink plate. The reactions are performed in 7 mL crimp cap vials. Six vials can be simultaneously and homogenously irradiated from the bottom side with six LEDs. The temperatures can be maintained at the desired value (25-50 °C) using a custom-made aluminium cooling block connected to a thermostat.
LEDs specification: Blue LEDs OSRAM Oslon SSL 80 LDCQ7P-1U3U. The LEDs wavelength emission profile resulted to be 455 (± 15) nm. The light intensity (expressed as mW/cm^2) have been determined using and a Thorlabs PM200 power meter equipped with a S130VC power head with a Si detector. The measured light intensities resulted to be 18.25 W/cm^2.

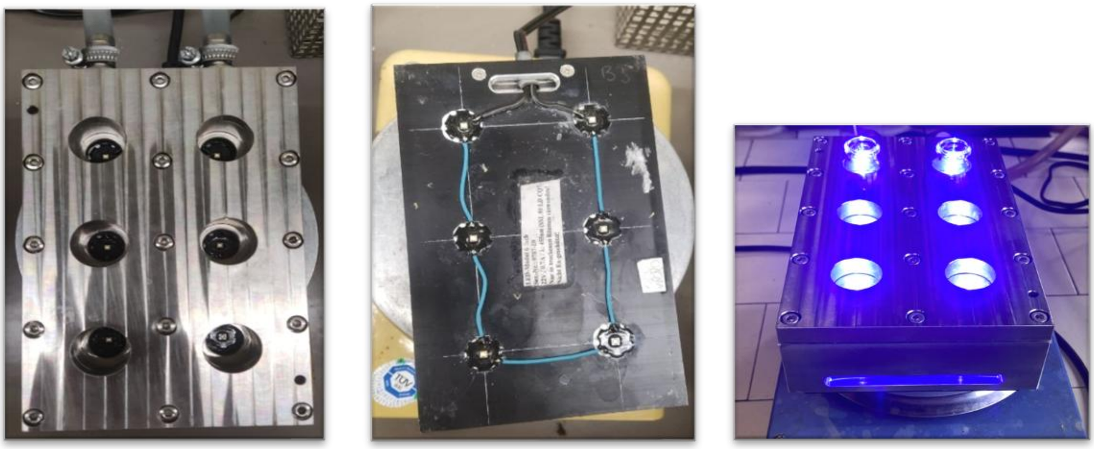

Figure S2: Plate-based photoreactor

2.3. Vapourtec E-series photoreactor

The Vapourtec photochemical reactor (**Figure S3**) is an easy-Photochem E-Series UV-150 reactor, equipped with high-power LEDs (450 nm, 24 W), a power supply and a fluoropolymer tubing as reactor coil of 10 mL volume. For the experiments under nitrogen atmosphere, the degassed mixture in sealed vial for microwave reactions (0.5–2.0 mL vials from Biotage®, or in a V flask for the gram scale reaction) is fluxed by a V-3 pump at the set flow rate through a needle, taking care to use degassed acetonitrile to fill the reactor immediately before and after the reaction mixture.

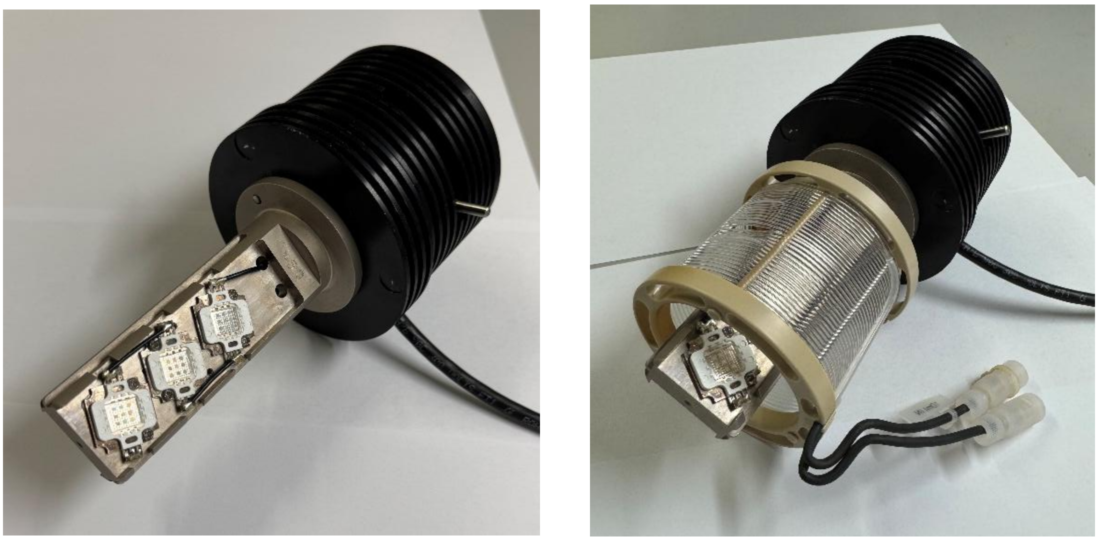

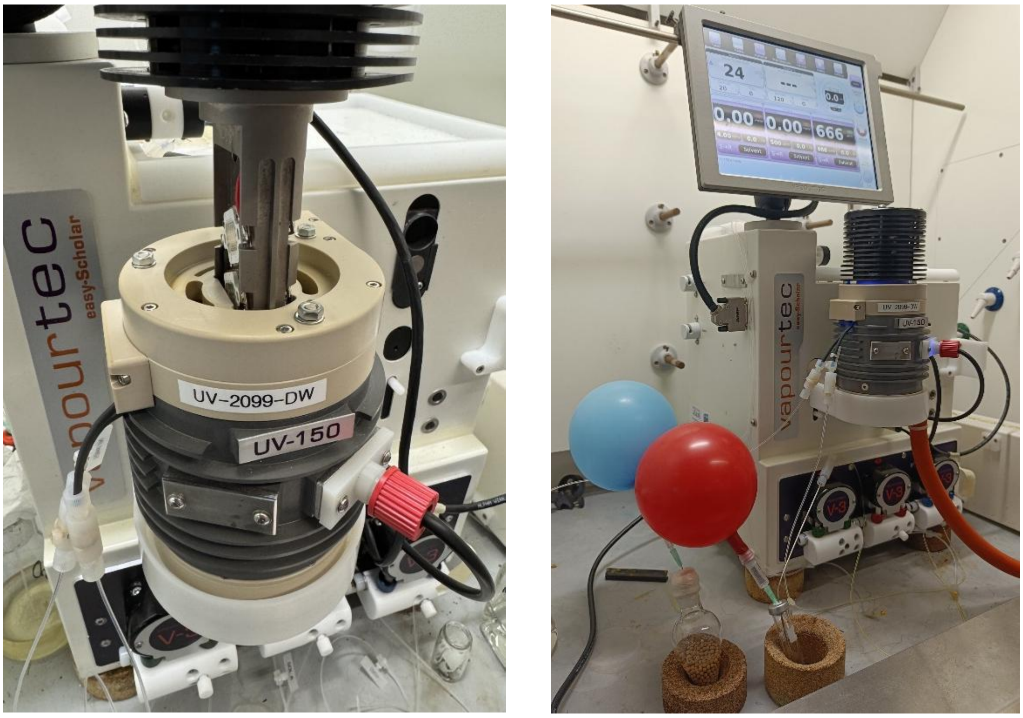

Figure S3: Vapourtec photochemical reactor

3. Synthesis and characterization of the substrates

3.1. Synthesis of 3-amino-2-oxazolidinone **1**

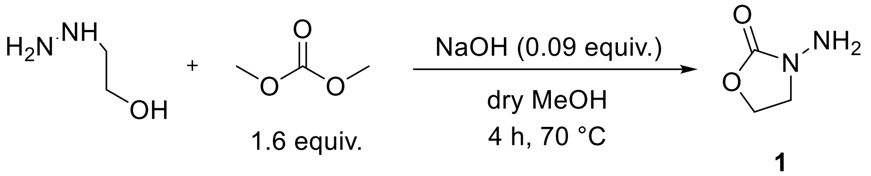

1

72 %

The starting material **1** is commercially available and expensive. Thus, it can be easily prepared in gram scale following a literature protocol.[2] To a stirrer solution of 2-hydroxylethylhydrazine (8.9 mL, 131.4 mmol, 1 equiv., 56 M in methanol) and dimethyl carbonate (17.7 mL, 210.2 mmol, 1.6 equiv.) at room temperature, was added a solution of NaOH (473 mg, 11.82 mmol, 0.09 equiv.) in 2.35 mL of dry methanol. The resulting mixture was then stirred at 70 °C by using an oil bath for 4 h and allowed to cool to room temperature. The unreacted dimethyl carbonate was removed under reduced pressure. The resulting crude, a colourless oil which solidifies upon standing as yellowish solid, was purified by automatic flesh column chromatography on silica gel (PuriFlash®, DCM: MeOH = 96.5: 3.5). The desired product was recovered as a white solid in 72 % yield (9.64 g).

^{1}H NMR (300 MHz, $CDCl_3$) δ 4.25 (t, *J* = 7.5 Hz, 2H), 4.01 (br s, 2H), 3.72 – 3.59 (t, *J* = 7.5 Hz, 2H). **^{13}C NMR** (75 MHz, $CDCl_3$) δ 159.7, 61.2, 48.5. **IR (neat)** v/cm^{-1}: 3462 (w, br), 3331 (m), 3200 (w), 2924 (w), 1727 (s), 1638 (w), 1419 (w), 1206 (w), 1029 (m), 969 (w), 761 (m). **HRMS (ESI-TOF)** m/z: $[M+Na]^+$ Calcd for $C_3H_6N_2O_2Na$ 125.0322; Found 125.0324. **Rf** = 0.18 (DCM: MeOH = 30:1). **M. p.** 62 °C – 64 °C.

3.2. Synthesis of pyrylium salts **2a** and **2b**

2,4,6-trimethylpyrylium tetrafluoroborate (2a)

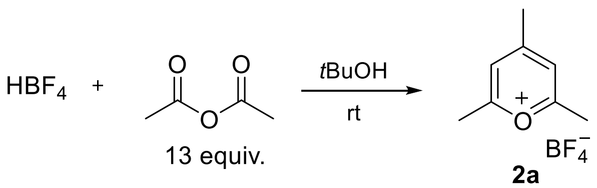

2a

56 %

According to a literature procedure,[3] 250 mL three-necked round bottom flask equipped with a dropping funnel, a condenser and a thermometer was charged with acetic anhydride (56 mL, 591 mmol, 13 equiv.) and *tert*-butanol (4.5 mL, 47 mmol, 1 equiv.), providing a colourless solution. HBF_4 48 wt. % in H_2O (6 mL, 44 mmol, 0.95 equiv.) was added dropwise. The addition was exothermic, the solution turned yellow at first, and then reddish. After completion of the addition, the obtained brown solution was cooled from 80 °C to room temperature and then to 0 °C by dipping the flask in an ice bath. The separation of the salt was observed and it ended by the addition of chilled Et_2O (100 mL). The white precipitate was recovered by filtration on a Buckner under vacuum, washed with chilled Et_2O and dried. The desired product **2a** was recovered in 56 % yield as a white solid (5.14 g). All the analytical data are in agreement with the literature.[4]

^{1}H NMR (300 MHz, MeOD) δ 7.88 (s, 2H), 2.88 (s, 6H), 2.73 (s, 3H). **^{1}H NMR** (300 MHz, CD_3CN) δ 7.70 (s, 2H), 2.80 (s, 6H), 2.65 (s, 3H). **^{19}F NMR** (282 MHz, MeOD) δ -154.69.

2,4,6-trimethylpyrylium trifluoromethanesulfonate (2b)

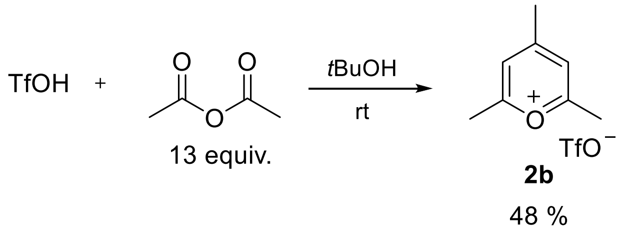

According to a literature procedure[5], 250 mL three-necked round bottom flask equipped with a dropping funnel, a condenser and a thermometer was charged with acetic anhydride (18 mL, 192 mmol, 5.8 equiv.) and *t*BuOH (3.2 mL, 4 mmol, 1.03 equiv.), providing a colourless solution. Trifluoromethasanesulfonic acid (TfOH; 3 mL, 33 mmol, 1 equiv.) was slowly added dropwise under vigorous stirring, controlling that the temperature is kept under 90 °C. At the end of the exothermic addition, the temperature was 60-70 °C. The obtained brown solution was slowly cooled down to room temperature, then to 0°C with an ice bath. Chilled Et_2O (100 mL) was added, whereupon a white precipitate separated. The salt was filtered on a Buckner under vacuum, washed with chilled Et_2O and dried. The desired product **2b** was obtained in 48 % yield (2.9 g) as a brown solid. All the analytical data are in agreement with the literature.[6]

^{1}H NMR (400 MHz, DMSO) δ 7.81 (s, 2H), 2.66 (s, 6H), 2.34 (s, 3H). **^{1}H NMR** (300 MHz, CD_3CN) δ 7.74 (s, 2H), 2.83 (s, 6H), 2.68 (s, 3H). **^{19}F NMR** (377 MHz, DMSO) δ -77.33.

3.3. Synthesis of pyridinium salts **3a** and **3b**

2,4,6-trimethyl-1-(2-oxooxazolidin-3-yl)pyridinium tetrafluoroborate (3a)

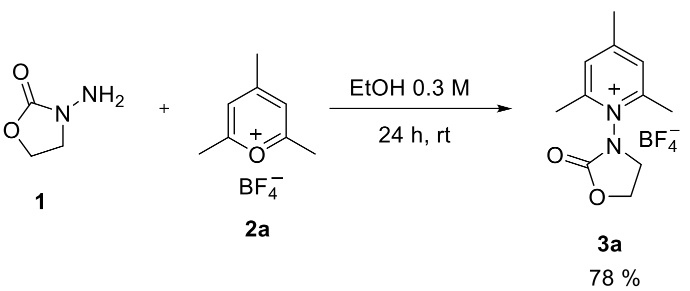

According to a literature procedure[4], 3-amino-2-oxazolidinone **1** (1.164 g, 11.4 mmol, 1 equiv., 0.3 M in EtOH) was dissolved in EtOH (38 mL) and 2,4,6-trimethylpyrylium tetrafluoroborate **2a** (2.394 g, 11.4 mmol, 1 equiv., 0.3 M in EtOH) was added. The reaction mixture was stirred for 24 h at room temperature. Then, the system was cooled to 0 °C, chilled Et_2O (38 mL) was added and the formation of a white precipitate was observed. The salt was filtered on a Buckner, washed with chilled Et_2O and dried. In analogy to literature procedure[7], the crude was purified by recrystallization from DCM-EtOH solution (2:1 v/v ratio), and then completely precipitated via dropwise addition of chilled Et_2O. The resulting solid was filtered on a Buckner, and the precipitate washed with additional chilled Et_2O and then dried to yield the desired pyridinium salt **3a** in 78 % of yield (2.62 g) as a white solid.

^{1}H NMR (400 MHz, CD_3CN) δ 7.72 (s, 2H), 4.76 (td, J = 7.9, 1.7 Hz, 2H), $4.^{15}$ (td, J = 7.9, 1.2 Hz, 2H), 2.70 (s, 6H), 2.59 (s, 3H). **^{19}F NMR** (376 MHz, CD_3CN) δ -151.69, -151.70, -151.71, -151.74, -151.75, -151.76. **^{13}C NMR** (75 MHz, CD_3CN) δ 164.0, 158.8 (2C), 154.2, 129.9 (2C), 64.9, 45.9, 22.4, 19.4 (2C). **IR (neat)** v/cm^{-1}: 3079 (w), 1777 (s), 1635 (m), 1402 (m), 1232 (m), 1145 (w), 1049 (s), 1026 (s), 864 (m), 755 (m). **HRMS (QTOF)** m/z: $[M]^+$ Calcd for $C_{11}H_{15}N_2O_2^+$ 207.1128; Found 207.1130. **M. p.** 210 °C – 212 °C.

2,4,6-trimethyl-1-(2-oxooxazolidin-3-yl)pyridinium trifluoromethanesulfonate (3b)

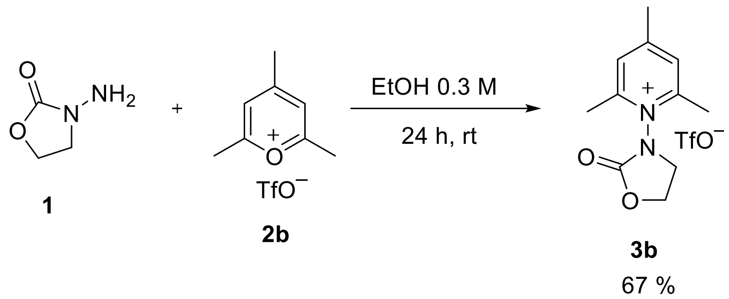

According to a literature procedure[4], 3-amino-2-oxazolidinone **1** (1.103 g, 10.8 mmol, 1 equiv., 0.3 M in EtOH) was dissolved in EtOH (36 mL) and 2,4,6-trimethylpyrylium trifluoromethanesulfonate **2b** (2.940 g, 10.8 mmol, 1 equiv., 0.3 M in EtOH) was added. The reaction mixture was stirred for 24 h at room temperature. Then, the system was cooled to 0°C, chilled Et_2O (36 mL) was added and the formation of a white precipitate was observed. The salt was filtered on a Buckner, washed with chilled Et_2O and dried to yield the desired pyridinium salt **3b** in 67 % of yield (2.57 g) as a white-brownish solid.

^{1}H NMR (400 MHz, CD_3CN) δ 7.73 (s, 2H), 4.76 (td, *J* = 7.9, 1.7 Hz, 2H), 4.15 (td, *J* = 7.9, 1.7 Hz, 2H), 2.70 (s, 6H), 2.59 (s, 3H). **^{19}F NMR** (376 MHz, CD_3CN) δ -79.23. **^{13}C NMR** (75 MHz, CD_3CN) δ 164.0, 158.8 (2C), 154.2, 129.9 (2C), 64.9, 45.9, 22.4, 19.4 (2C). **IR (neat)** ν/cm^{-1}: 3053 (w), 1764 (s), 1634 (m), 1484 (w), 1409 (w), 1259 (s), 1219 (m), 1144 (s), 1021 (s), 859 (w), 751 (m), 625 (m). **HRMS (QTOF)** m/z: $[M]^+$ Calcd for $C_{11}H_{15}N_2O_2^+$ 207.1128; Found 207.1131. **M. p.** 144 °C – 145 °C.

3.4. Single Crystal X-ray diffraction structures of **3a** and **3b**

The crystals were prepared by placing 7-10 mg of pure precursor into a vial. The minimum possible volume of acetonitrile (a few drops) was added to solubilise the salt and each vial was then covered with perforated parafilm to promote a slow evaporation and a suitable crystal growth for the further analysis.

Diffraction data for compound **3a** and compound **3b**, both translucent, colourless, and block-shaped, were collected at temperatures of 107.30(14) K and 100.70(14) K, respectively, using a Rigaku SuperNova single-crystal four-circle diffractometer. The instrument was equipped with a micro-focus sealed X-ray tube, a mirror monochromator, and an Atlas detector. CuKα radiation (λ = 1.54184 Å) was employed for both datasets.

For compound **3a**, a total of 19,417 reflections were recorded, while for compound **3b**, 24,699 reflections were collected. The collected reflections were integrated using CrysAlisPro software, and a Gaussian absorption correction was applied using the SCALE3 ABSPACK[5] method. After data collection, the structures were solved with SHELXT software using dual methods. The structures were then refined using full matrix least squares techniques against F^2 with SHELXL.[6-8]

The refinement results showed that both crystalline structures belong to the monoclinic crystal system and crystallize in the $P2_1/c$ (14) space group. The unit cell parameters obtained were as follows: for compound **3a**, the unit cell dimensions were a = 6.62470(10) Å, b = 8.38880(10) Å, c = 23.4551(2) Å, and β = 96.5120(10)°, with a volume of 1295.07(3) Å^3. For compound **3b**, the unit cell parameters were a = 11.5954(2) Å, b = 11.8325(2) Å, c = 11.1049(2) Å, and β = 104.625(2)°, with a volume of 1474.25(5) Å^3.

Non-hydrogen atoms were refined with anisotropic displacement parameters, while hydrogen atoms were refined isotropically based on calculated positions. For terminal sp^3 carbon atoms, the Uiso values were constrained to 1.5 times the Ueq of the pivot atoms, and for all other carbon atoms, the Uiso values were set to 1.2 times the Ueq of the pivot atoms.

Finally, the refinement parameters for compound **3a** were R1 = 0.0347, wR2 = 0.0854, and a goodness-of-fit value S = 1.034. For compound **3b**, the values were R1 = 0.0405, wR2 = 0.0977, and the goodness-of-fit value S = 1.042. All the crystallographic parameters are reported in **Table S1**.

	Compound **3a**	Compound **3b**
**CCDC number**	2469874	2469875
**Empirical formula**	C11H15BF4N2O2	C12H15F3N2O5S
**Formula weight**	294.06	356.32
**Temperature [K]**	107.30(14)	100.70(14)
**Crystal system**	monoclinic	monoclinic
**Space group (number)**	$P2_1/c$ (14)	$P2_1/c$ (14)
**a [Å]**	6.62470(10)	11.5954(2)
**b [Å]**	8.38880(10)	11.8325(2)
**c [Å]**	23.4551(2)	11.1049(2)
**α [°]**	90	90
**β [°]**	96.5120(10)	104.625(2)
**γ [°]**	90	90
**Volume [Å3]**	1295.07(3)	1474.25(5)
**Z**	4	4
**ρcalc [gcm−3]**	1.508	1.605
**μ [mm−1]**	1.231	2.555
**F(000)**	608	736
**Crystal size [mm3]**	0.41×0.23×0.16	0.35×0.29×0.09
**Crystal colour**	translucent light colourless	translucent light colourless
**Crystal shape**	block	block
**Radiation**	Cu Kα (λ=1.54184 Å)	Cu Kα (λ=1.54184 Å)
**2Θ range [°]**	7.59 to 153.65 (0.79 Å)	7.88 to 153.58 (0.79 Å)
**Index ranges**	-8 ≤ h ≤ 8<br>-10 ≤ k ≤ 10<br>-23 ≤ l ≤ 29	-14 ≤ h ≤ 14<br>-14 ≤ k ≤ 14<br>-13 ≤ l ≤ 14
**Reflections collected**	19417	24699
**Independent reflections**	2729<br>Rint = 0.0252<br>Rsigma = 0.0124	3091<br>Rint = 0.0435<br>Rsigma = 0.0188
**Completeness**	100.0 %	100.0 %
**Data / Restraints / Parameters**	2729/0/184	3091/0/211
**Goodness-of-fit on F2**	1.034	1.042
**Final R indexes [I≥2σ(I)]**	R1 = 0.0317<br>wR2 = 0.0820	R1 = 0.0352<br>wR2 = 0.0926
**Final R indexes [all data]**	R1 = 0.0347<br>wR2 = 0.0854	R1 = 0.0405<br>wR2 = 0.0977
**Largest peak/hole [eÅ−3]**	0.22/-0.27	0.27/-0.47

Table S1. Crystallographic parameters for compounds **3a** and **3b**.

The asymmetric unit of pyridinium tetrafluoroborate **3a** and pyridinium triflate **3b** with atoms labelled and thermal ellipsoids are shown in **Figure S4** and **Figure S5**.

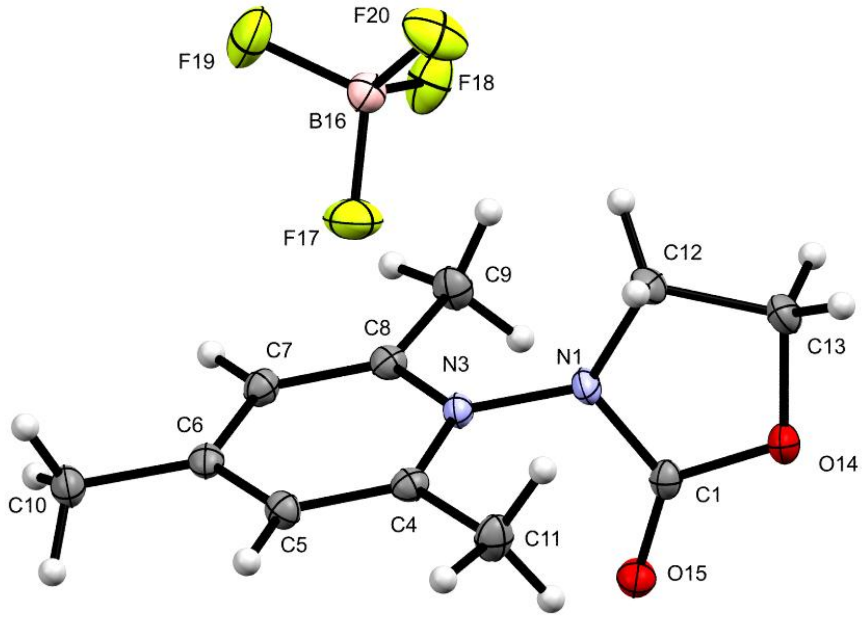

Figure S4. The asymmetric unit of **3a** with atoms labelled and thermal ellipsoids shown at 50% probability.

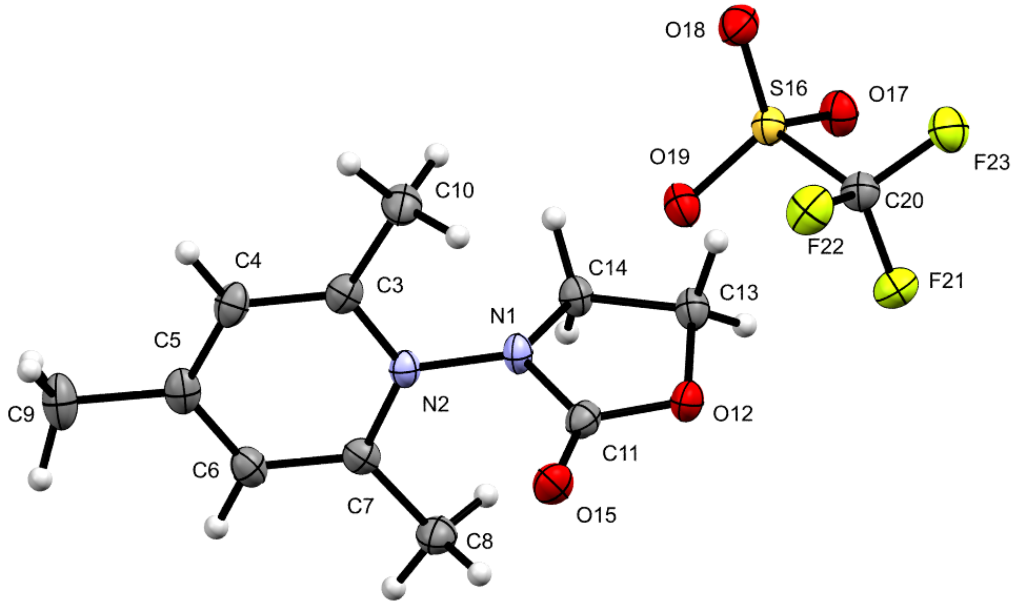

Figure S5. The asymmetric unit of **3b** with atoms labelled and thermal ellipsoids shown at 50% probability.

The hydrogen bond parameters for structures **3a** and **3b** are reported in **Table S2**.

D-H…A	D-H (Å)	H…A (Å)	D…A(Å)	D-H…A (°)
		**Compound 3a**		
C5-H5…O15	0.95	2.49	3.1251(13)	124
C7-H7…F18	0.95	2.45	3.3357(14)	156
C12-H12B…F18	0.99	2.42	3.2184(14)	138
C13-H13B…O15	0.99	2.46	3.2608(14)	137
		**Compound 3b**		
C6-H6…O17	0.95	2.40	3.289(2)	156
C8-H8B…O18	0.98	2.52	3.500(2)	174
C13-H13A…O19	0.99	2.32	3.289(2)	167

Table S2. Hydrogen Bond Parameters for Structures **3a** and **3b**.

The crystal packing of **3a** and **3b** viewed along the a-axis is illustrated in **Figure S6** and **Figure S7**.

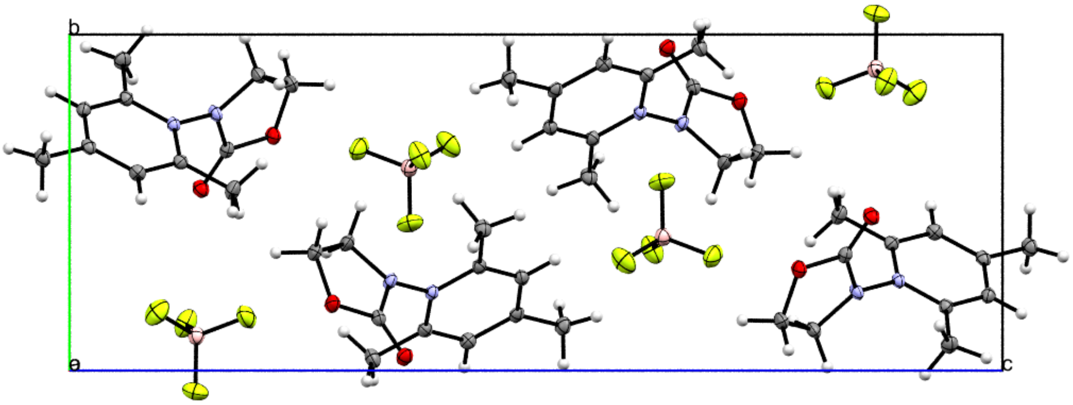

Figure S6. Crystal packing of compound **3a**, viewed along the a-axis.

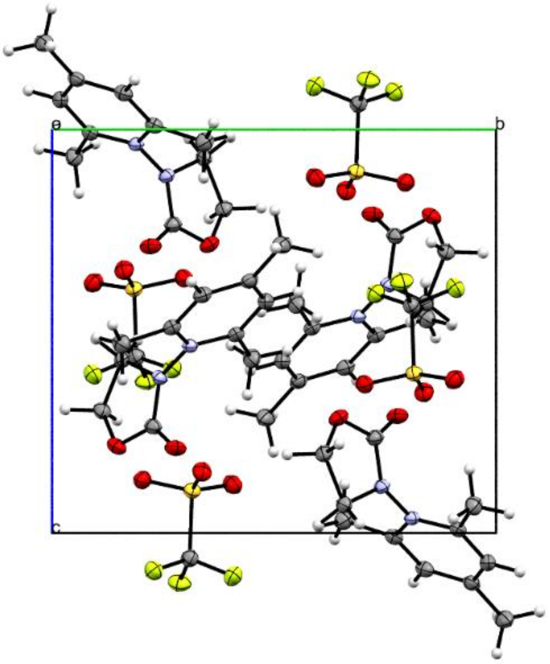

Figure S7. Crystal packing of compound **3b**, viewed along the a-axis.

3.5. Cyclic Voltammetry analyses of **3a** and **3b**

Cyclic Voltammetry analyses of compounds **3a** and **3b** were carried out with a Metrohm Series Potentiostat/ Galvanostat Electrochemical System Autolab at room temperature. Glassy carbon was employed as working electrode, a platinum foil as counter electrode and a silver wire as reference electrode. Glassy carbon electrode and platinum foil were purchased by IKA. The surface in solution of the glassy carbon plate and platinum foil has been calculate to be 0.4 cm^2. The set-up used is reported in **Figure S8**.

Tetrabutylammonium tetrafluoroborate (TBATFB) was selected as a supporting electrolyte at 0.1 M to avoid any possible interferences with other anions. Dry DMA was used as solvent to replicate the reaction conditions. Two measurements were carried out for each compound (at 4.5 mM and 9 mM). Prior to each measurement, which was recorded three times, the solution was degassed with argon and all the experiments are performed under argon atmosphere. Cyclic voltammetry analyses were scanned from an initial potential of 0 V to 1.3 V, then from 1.3 V to – 2.2 V. The scan rate was 100 $mV \cdot s^{-1}$. The solution of LiCl for the reference electrode in the inner jacket was freshly prepared.

Polishing materials and methods for the electrodes: The glassy carbon working electrode was polished with aluminium oxide in distilled water, then sonicated in distilled water and ethanol before measurements. The platinum counter electrode and the jacket of the reference electrode were washed with distilled water and ethanol before measurements.

The reduction potential was determined to be – 1.04 V and – 1.13 V versus Ag/AgCl (LiCl 2 M in EtOH) respectively for compounds **3a** (**Figure S9**) and **3b** (**Figure S10**).

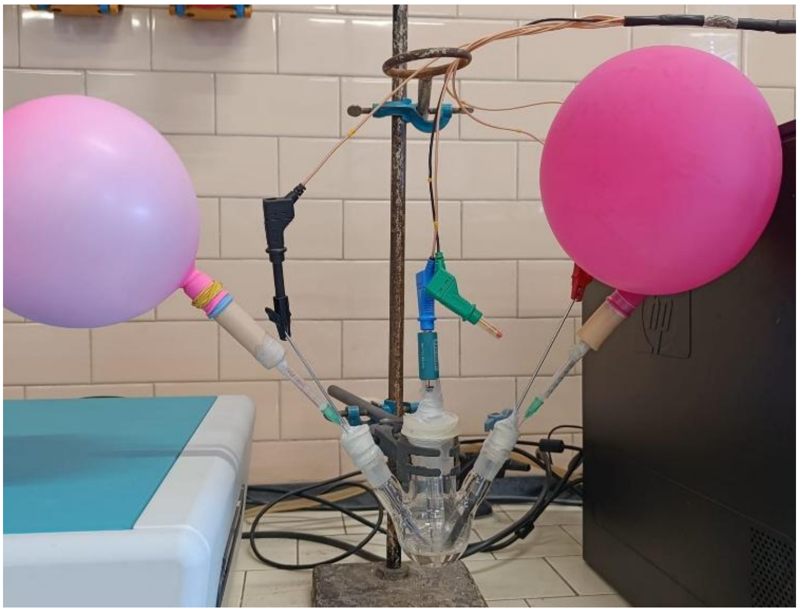

Figure S8: Set-up for CV analyses

2,4,6-trimethyl-1-(2-oxooxazolidin-3-yl)pyridinium tetrafluoroborate (3a)

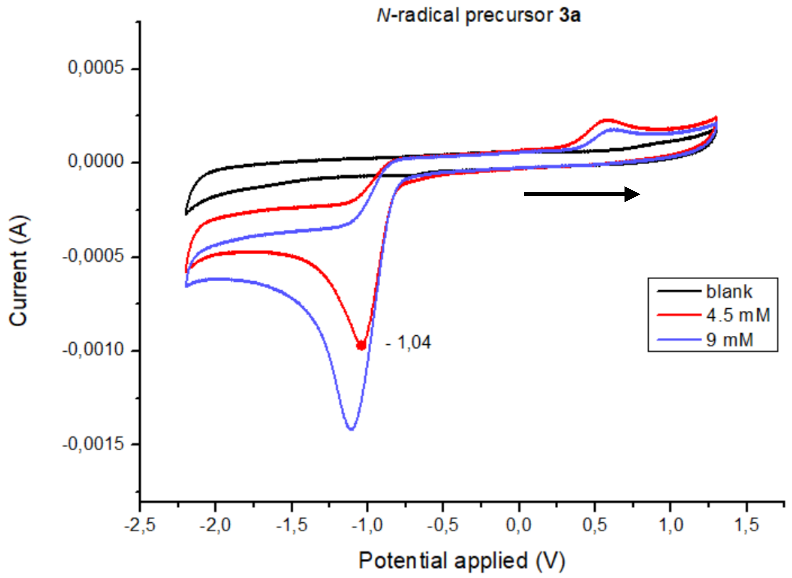

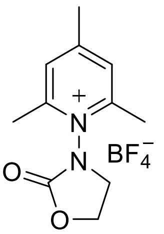

3a

Figure S9: Cyclic voltammogram for compound **3a** versus Ag/AgCl (LiCl 2 M in EtOH) with TBATFB 0.1 M in dry DMA at 100 mV/s from 0 V to + 1.3 V and from + 1.3 V to – 2.2 V at room temperature. Glassy carbon was employed as working electrode, a platinum foil as counter electrode and a silver wire as reference electrode. CV plotting convention is IUPAC.

2,4,6-trimethyl-1-(2-oxooxazolidin-3-yl)pyridinium trifluoromethanesulfonate (3b)

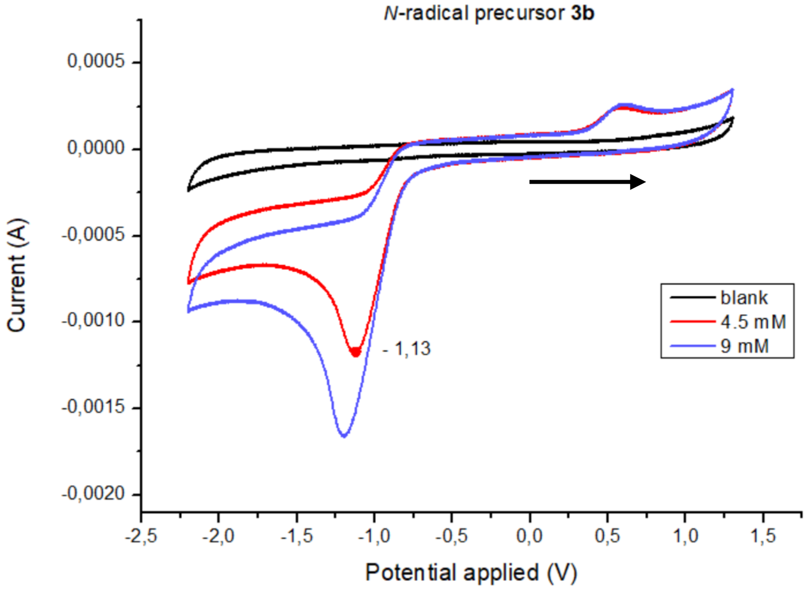

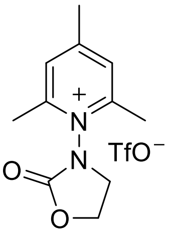

3b

Figure S10: Cyclic voltammogram for compound **3b** versus Ag/AgCl (LiCl 2 M in EtOH) with TBATFB 0.1 M in dry DMA at 100 mV/s from 0 V to + 1.3 V and from + 1.3 V to – 2.2 V at room temperature. Glassy carbon was employed as working electrode, a platinum foil as counter electrode and a silver wire as reference electrode. CV plotting convention is IUPAC.

3.6. Synthesis of heteroarenes substrates for the scope **4b-d**, **4f-h**, **4l**

General procedure for the methylation of indoles (GP1)

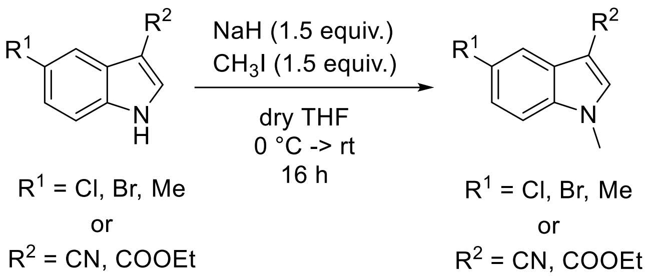

According to a literature procedure[12], the indole derivative (2.64 mmol, 1 equiv., 0.3 M in THF) was dissolved in dry THF (8.8 mL) and 158.4 mg of NaH 60% dispersion in mineral oil (3.96 mmol, 1.5 equiv.) was added at 0° C portion wise. After stirring for 30 min at 0 °C by using an ice bath, the reaction mixture was allowed to warm to room temperature and stirred for further 30 min. Then, 0.247 mL of methyl iodide (3.96 mmol, 1.5 equiv.) was added dropwise at 0 °C, afterwards the reaction mixture was allowed to warm to room temperature and stirred overnight. After cooling back to 0 °C through an ice bath, the reaction was quenched with water, extracted with ethyl acetate; then the combined organic layers were washed with brine, dried over anhydrous Na_2SO_4, and concentrated under reduced pressure. The residue was purified by flash column chromatography to give the N-methylated indoles.

5-chloro-1-methyl-1H-indole (4b)

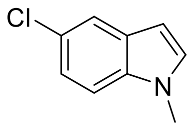

Starting from commercially available 5-chloro-1-H-indole (400 mg, 2.64 mmol), 5-chloro-1-methyl-1H-indole **4b** was prepared according to GP1. After purification by flash column chromatography (hexane: EtOAc = 98: 2 → 96: 4), it was obtained in 86 % (375 mg) as a yellowish oil which solidified upon standing. All the data are in agreement with the literature.[13]

^{1}H NMR (300 MHz, $CDCl_3$) δ 7.58 (d, *J* = 1.9 Hz, 1H), 7.23 (d, *J* = 8.3 Hz, 1H), 7.16 (dd, *J* = 8.6, 2.0 Hz, 1H), 7.07 (d, *J* = 3.1 Hz, 1H), 6.42 (d, *J* = 3.1 Hz, 1H), 3.78 (s, 3H). **Rf** = 0.28 (Hexane: EtOAc = 9: 1).

5-bromo-1-methyl-1H-indole (4c)

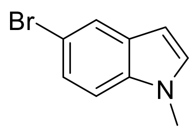

Starting from the commercially available 5-bromo-1-H-indole (600 mg, 3.06 mmol), 5-bromo-1-methyl-1H-indole **4c** was prepared according to GP1. It was obtained without further purification in 98 % (627 mg) as a yellow oil which solidified upon standing as a yellowish solid. All the data are in agreement with the literature.[13]

^{1}H NMR (300 MHz, $CDCl_3$) δ 7.74 (d, *J* = 1.9 Hz, 1H), 7.30 (dd, *J* = 8.7, 1.9 Hz, 1H), 7.19 (d, *J* = 8.7 Hz, 1H), 7.05 (d, *J* = 3.1 Hz, 1H), 6.42 (d, *J* = 3.1 Hz, 1H), 3.78 (s, 3H). **Rf** = 0.25 (Hexane: EtOAc = 9: 1).

1,5-dimethyl-1H-indole (4d)

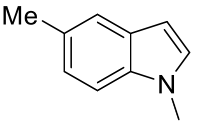

Starting from the commercially available 5-methyl-1-H-indole (400 mg, 3.05 mmol), 1,5-dimethyl-1H-indole **4d** was prepared according to GP1. It was obtained in 90 % yield (390 mg) as a yellow oil after purification by flash column chromatography on silica gel (Hexane: EtOAc = 99.7: 0.3 → 98.5: 1.5). All the analytical data are in agreement with the literature.[14]

^{1}H NMR (400 MHz, $CDCl_3$) δ 7.48 (s, 1H), 7.27 (d, J = 8.3 Hz, 1H), 7.12 (d, J = 8.7 Hz, 1H), 7.06 (d, J = 3.1 Hz, 1H), 6.46 (d, J = 3.1 Hz, 1H), 3.81 (s, 3H), 2.52 (s, 3H). **Rf** = 0.27 (Hexane-EtOAc = 99.5: 0.5).

1-methyl-1H-indole-3-carbonitrile (4f)

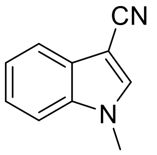

Starting from the commercially available 1H-indole-3-carbonitrile (600 mg, 4.22 mmol), 1-methyl-1H-indole-3-carbonitrile **4f** was prepared according to GP1. It was obtained without further purification in 93 % yield (613 mg) as an orange oil which solidified upon standing as a light brown solid. All the analytical data are in agreement with the literature.[15]

^{1}H NMR (300 MHz, $CDCl_3$) δ 7.77 (d, J = 7.7 Hz, 1H), 7.57 (s, 1H), 7.42 – 7.28 (m, 3H), 3.86 (s, 3H). **Rf** = 0.14 (Hexane-EtOAc = 85: 15).

Ethyl 1-methyl-1H-indole-3-carboxylate (4g)

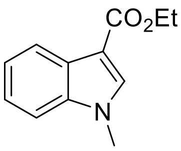

Starting from the commercially available ethyl 1H-indole-3-carboxylate (800 mg, 4.23 mmol), ethyl 1-methyl-1H-indole-3-carboxylate **4g** was prepared according to GP1. It was obtained without further purification in 77 % yield (659 mg) as a purple oil which solidified upon standing as a pink solid. All the analytical data are in agreement with the literature.[16]

^{1}H NMR (300 MHz, $CDCl_3$) δ 8.18 – 8.15 (m, 1H), 7.77 (s, 1H), 7.35 – 7.24 (m, 3H), 4.37 (q, J = 7.1 Hz, 2H), 3.82 (s, 3H), 1.41 (t, J = 7.1 Hz, 3H). **Rf** = 0.16 (Hexane-EtOAc = 9: 1).

General procedure for the arylation of heteroarenes (GP2)

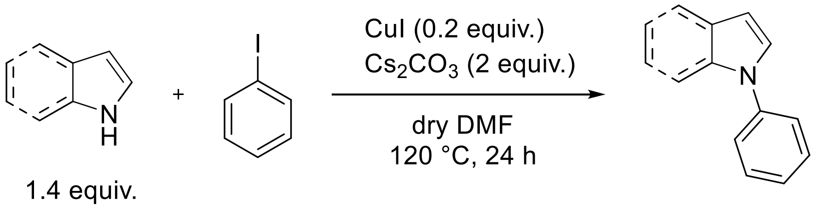

According to a literature procedure[17], a mixture of the heteroarene (7.0 mmol, 1.4 equiv.), 0.564 mL of iodobenzene (5.0 mmol, 1 equiv., 0.5 M in DMF), 191 mg of CuI (1.0 mmol, 0.2 equiv.), 3.26 g of Cs_2CO_3 (10 mmol, 2 equiv.), in dry DMF (10 mL) was vigorously stirred at 120 °C under nitrogen atmosphere for 24 h using an oil bath. After cooling the reaction mixture to room temperature, it was diluted with EtOAc (40 mL) and washed with water (2 × 30 mL). The aqueous phase was extracted with EtOAc and the combined organic layers were dried over anhydrous Na_2SO_4. After filtration and evaporation of the solvents under vacuum, the residue was purified by flash column chromatography on silica gel to give the N-arylated heteroarene.

1-phenyl-1H-indole (4h)

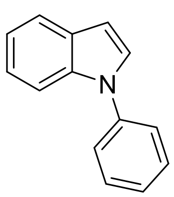

Starting from the commercially available 1H-indole (820 mg), 1-phenyl-1H-indole **4h** was prepared according to general procedure GP2. The desired product was obtained after purification by flash column chromatography on silica gel (cyclohexane: pentane = 8: 2 → 6: 4) in 87 % yield (838 mg) as a brown oil. All the analytical data are in agreement with the literature.[17]

^{1}H NMR (400 MHz, $CDCl_3$) δ 7.70 (ddd, J = 7.7, 1.5, 0.8 Hz, 1H), 7.57 (dq, J = 8.3, 0.9 Hz, 1H), 7.53 (d, J = 1.3 Hz, 2H), 7.52 (s, 2H), 7.40 – 7.33 (m, 2H), 7.23 (td, J = 7.1, 1.3 Hz, 1H), 7.17 (td, J = 7.5, 1.2 Hz, 1H), 6.69 (dd, J = 3.3, 0.9 Hz, 1H). **Rf** = 0.27 (cyclohexane: pentane = 75: 25).

1-phenyl-1H-pyrrole (4l)

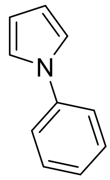

Starting from the commercially available 1H-pyrrole (470 mg), 1-phenyl-1H-pyrrole **4l** was prepared according to general procedure GP2. The desired product was obtained after purification by flash column chromatography on silica gel (cyclohexane: pentane = 9: 1 → 8: 2) in 76 % yield (542 mg) as a white solid. All the analytical data are in agreement with the literature.[17]

^{1}H NMR (400 MHz, $CDCl_3$) δ 7.48 – 7.35 (m, 4H), 7.28 – 7.22 (m, 1H), 7.10 (t, J = 2.2 Hz, 2H), 6.36 (t, J = 2.2 Hz, 2H). **Rf** = 0.22 (cyclohexane).

4. Optimization of the photocatalytic reaction

The photocatalytic activation of compound **3a** (by N-N bond breaking) and its subsequent reaction with 1-methylindole **4a** was deeply investigated, optimizing all the impacting parameters (**Tables S3-S9**).

The metal-based photocatalysts, Tris(2,2′-bipyridyl) dichlororuthenium (II) hexahydrate ($Ru(bpy)_3Cl_2 \cdot 6H_2O$) and Tris(2-phenylpyridine)iridium(III) (*fac*-$Ir(ppy)_3$) were purchased from Sigma-Aldrich and used without further purification.
In contrast, 1,2,3,5-tetrakis(carbazol-9-yl)-4,6-dicyanobenzene (4CzIPN) has been synthesized according to a literature protocol.[1]

Synthesis of 1,2,3,5-tetrakis(carbazol-9-yl)-4,6-dicyanobenzene (4CzIPN)

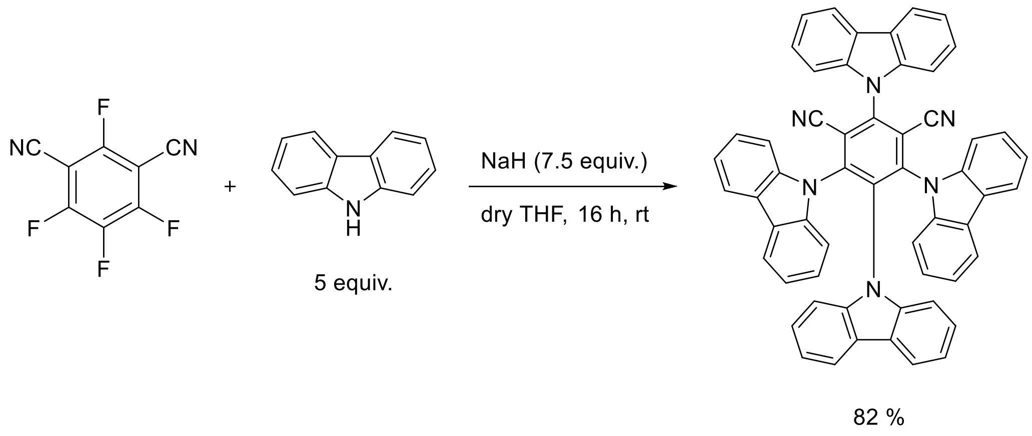

Under nitrogen atmosphere, NaH (60% in mineral oil, 0.60 g, 15.0 mmol, 7.5 equiv.) was added slowly to a stirred solution of carbazole (1.67 g, 10.0 mmol, 5 equiv.) in dry THF (40 mL) at room temperature. After 30 min, tetrafluoroisophthalonitrile (0.40 g, 2.0 mmol, 1 equiv., 0.05 M) was added. After stirring for 16 h at room temperature, 2 mL of water was added to the mixture to quench the excess NaH. The resulting mixture was then concentrated under reduced pressure and the solid residue was dissolved in DCM (50 mL). The organic phase was washed with brine (2 x 50 mL) and dried over anhydrous Na_2SO_4. The solvent was removed and the residue was purified by automated flash column chromatography (pentane: DCM = 85:15 → 4:6), obtaining the desired product in 82 % yield (1.294 g) as a yellow solid. All the analytical data are in agreement with the literature.[18]

^{1}H NMR (400 MHz, $CDCl_3$) δ 8.23 (d, *J* = 7.7 Hz, 2H), 7.84 – 7.61 (m, 8H), 7.59 – 7.40 (m, 2H), 7.33 (d, *J* = 7.7 Hz, 2H), 7.30 – 7.18 (m, 4H), 7.17 – 7.02 (m, 8H), 6.96 – 6.75 (m, 4H), 6.71 – 6.53 (m, 2H).

4.1. Screening of the conditions in batch

General procedure for the photocatalytic addition of oxazolidinone radicals to 1-methyl-1H-indole in BATCH (GP3).

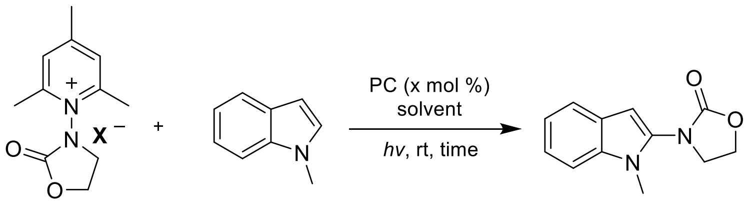

3a: X = BF_4 **4a** **5a**
3b: X = TfO

Two 7 mL vials for each reaction, were charged with 36.8 mg of the nitrogen radical precursor **3a** or 44.5 mg of **3b** (0.125 mmol, 1 equiv., 0.05 M in DMA), 0.8 mg of *fac*-Ir(ppy)$_3$ (1 mol %) and 31 µL of 1-methyl-1H-indole **4a** (0.25 mmol, 2 equiv.). The vials were sealed with a septum cap and three nitrogen-vacuum cycles were done. 2.5 mL of dry DMA were added to each vial and other three nitrogen-vacuum cycles were performed using the freeze pump thaw method. The reaction mixture was irradiated for 16 hours using the plate-based photoreactor (PR). Then, the vials were combined and the solvent was removed under vacuum. The crude was purified by flash column chromatography (hexane: EtOAc = 8: 2 → 5: 5) to provide compound **5a**. For 0.1 M reaction, one vial was used on a 0.25 mmol scale. For the cylinder PR, a 10 mL vial was used.

- Photocatalyst screening

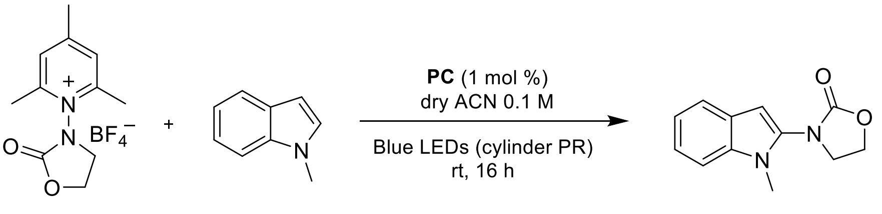

1.2 equiv.
3a **4a** **5a**

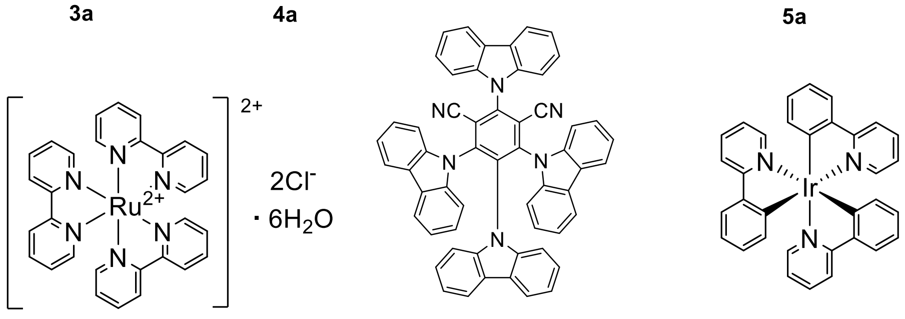

$Ru(bpy)_3Cl_2 \cdot 6H_2O$ **4czIPN** ***fac*-[Ir(ppy)$_3$]**

ENTRY	PHOTOCATALYST	YIELD (%)
1	$Ru(bpy)_3Cl_2$	24
2	4CzIPN	14
3	*fac*-Ir(ppy)$_3$	24

Table S3: Photocatalyst screening in batch

- Solvent screening

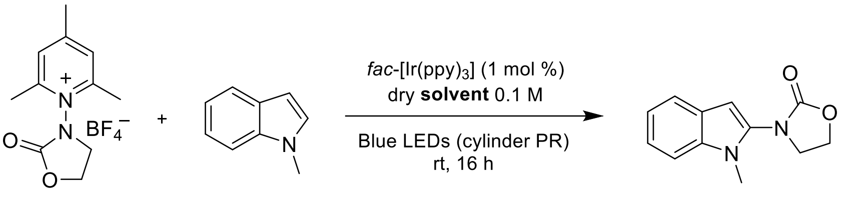

1.2 equiv.
3a **4a** **5a**

ENTRY	SOLVENT	YIELD (%)
**1**	ACN	24
**2**	DCM	31
**3**	DCE	37
**4**	EtOAc	35
**5**	DMF	44
**6**	DMA	48
**7**	DMSO	48
**8**	DMSO: water = 1:1	24

Table S4: Solvent screening in batch

- Photocatalyst loading and stoichiometry

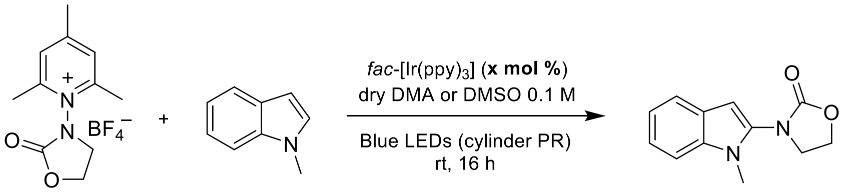

x equiv. **x equiv.**
3a **4a** **5a**

ENTRY	PC LOADING (mol %)	RATIO 3a : 4a	YIELD (%)
**1**	1	1.2 : 1	48
**2**	**2**	1.2 : 1	40
**3**	1	**1 : 2**	55

Table S5: Photocatalyst loading and stoichiometry screening in batch

- Photoreactor screening

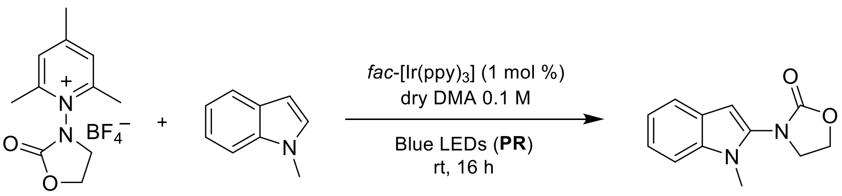

2 equiv.
3a **4a** **5a**

ENTRY	PHOTOREACTOR	YIELD (%)
**1**	cylinder-based	55
**2**	plate-based	66

Table S6: Photoreactor screening in batch

- Molarity and stoichiometry screening

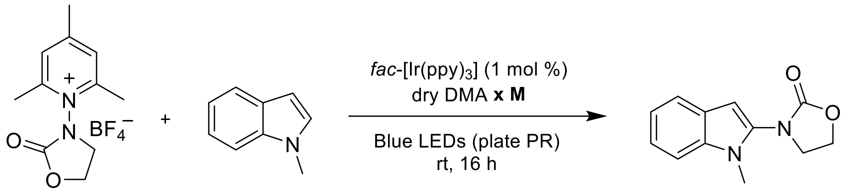

x equiv.

3a **4a** **5a**

ENTRY	MOLARITY (M)	RATIO 3a : 4a	YIELD (%)
**1**	0.1	1 : 2	66
**2**	**0.05**	1 : 2	**74**
**3**	0.05	**1 : 4**	68

Table S7: Molarity and stoichiometry screening in batch

- Time screening

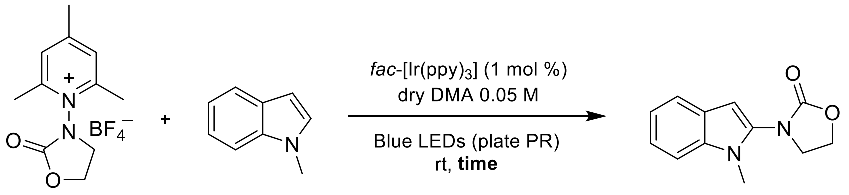

2 equiv.

3a **4a** **5a**

ENTRY	TIME (h)	YIELD (%)
**1**	16	**74**
**2**	8	52

Table S8: Time screening in batch

- Counter ion screening

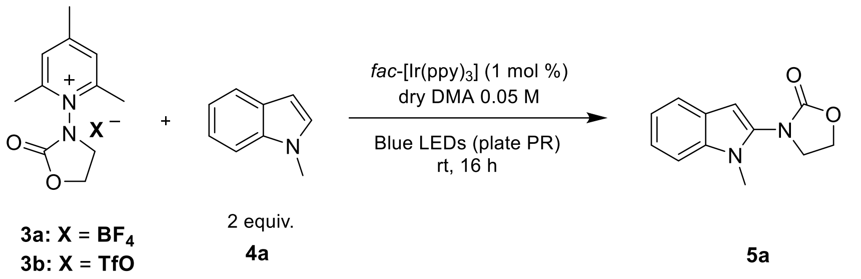

ENTRY	COUNTER ION	YIELD (%)
**1**	BF_4	**74**
**2**	TfO	70

Table S9: Counter ion screening in batch

4.2. Screening of the conditions in flow

To achieve a faster reaction optimization in flow (**Tables S10-S14**), ^{1}H-NMR yields were calculated using 1,1,2,2-tetrachloroethane as standard. 6-8 percentual points of difference between NMR and isolated yields are constantly present due to the IS batch used, affording the desired product in higher yields after the chromatographic purification. The experimental error is not present if dibromomethane is used as standard, whenever possible. In the following results, isolated yields are reported in brackets.

General procedure for the photocatalytic addition of oxazolidinone radicals to 1-methyl-1H-indole in FLOW (GP4).

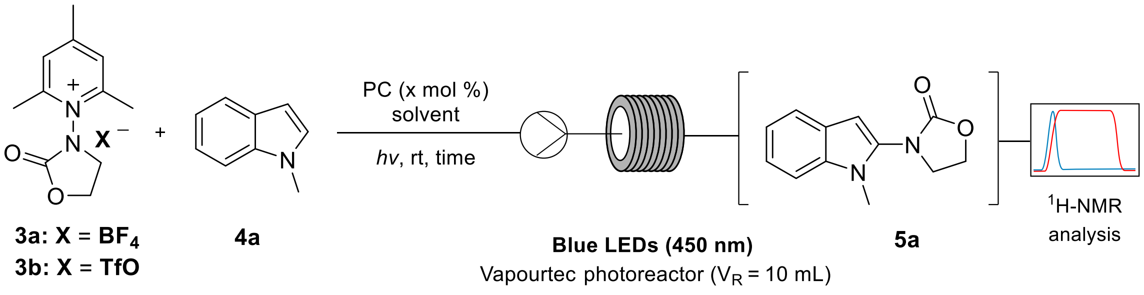

A microwave vial, was charged with 147 mg of the nitrogen radical precursor **3a** or 178 mg of **3b** (0.5 mmol, 1 equiv., 0.5 M in the solvent mixture ACN:DMA =1:1), 0.8 mg of *fac*-Ir(ppy)$_3$ (0.25 mol %) or 7.9 mg of 4CzIPN (2 mol %) and 124 µL of 1-methyl-1H-indole **4a** (1 mmol, 2 equiv.). The vial was sealed with a septum cap and three nitrogen-vacuum cycles were done. The dry solvent mixture ACN:DMA =1:1 (1 mL) was added and the reaction mixture was degassed for 5 minutes, unless otherwise indicated. The reaction was carried out under continuous flow conditions, irradiating the mixture for 15 min, with a Vapourtec photoreactor equipped with blue LEDs (450 nm, 24 W). After the collection, the solvent mixture was removed under reduced pressure and the crude was purified by flash column chromatography (pentane: EtOAc = 75: 25 → 5: 5) to provide 3-(1-methyl-1H-indol-2-yl)oxazolidin-2-one **5a**.

- Photocatalyst loading and molarity screening

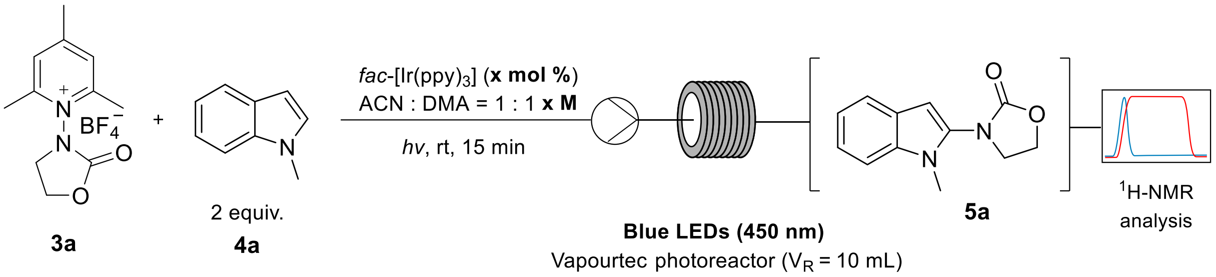

ENTRY	PC LOADING (mol %)	MOLARITY (M)	YIELD (%)[a]
**1**	1	0.05	71 (77)
**2**	1	0.1	71 (79)
**1**	1	0.2	71
**4**	1	0.25	71
**5**	0.5	0.3	72
**6**	0.5	0.35	72
**7**	0.5	0.4	71 (79)
**8**	0.25	0.5	72 (**80**)
**9**	0.2	0.5	63

[a] Yields determined by ^{1}H-NMR analysis, using 1,1,2,2- tetrachloroethane as standard; isolated yields in brackets.

Table S10: Photocatalyst loading and molarity screening in flow

- Residence time screening

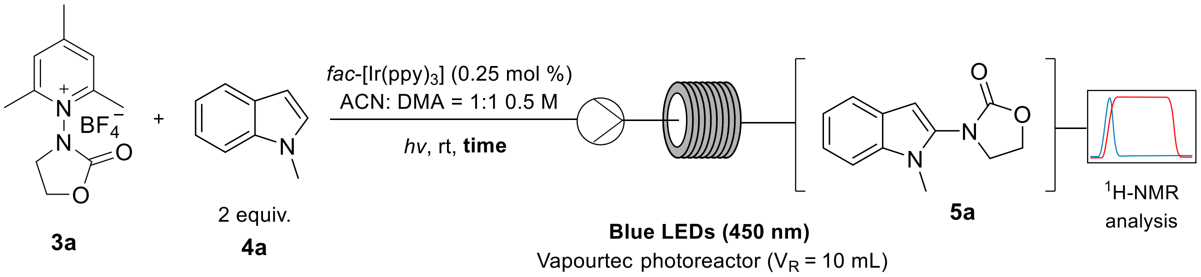

ENTRY	TIME (h)	YIELD (%)[a]
**1**	17.5	71
**2**	15	72 (**80**)
**3**	12.5	50
**4**	10	42

[a] Yields determined by ^{1}H-NMR analysis, using 1,1,2,2- tetrachloroethane as standard; isolated yields in brackets.

Table S11: Residence time screening in flow

- Photocatalyst screening

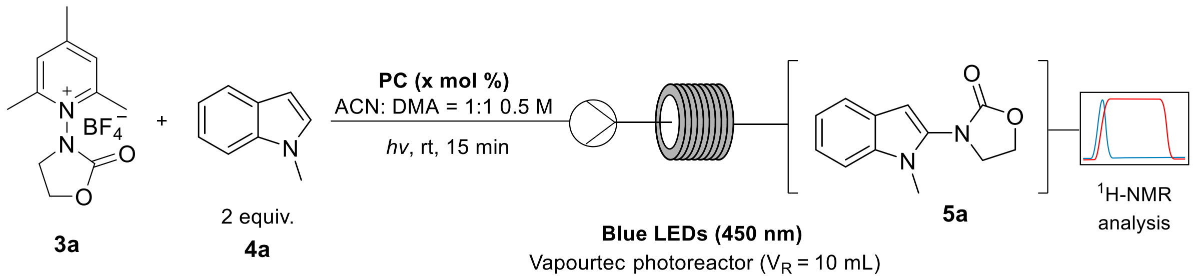

ENTRY	PHOTOCATALYST	PC LOADING (mol %)	YIELD (%)[a]
**1**	*fac*-Ir(ppy)$_3$	0.25	72 (**80**)
**2**	4CzIPN	0.25	29
**3**	4CzIPN	1	44
**4**	4CzIPN	2	59 (65)

[a] Yields determined by ^{1}H-NMR analysis, using 1,1,2,2- tetrachloroethane as standard; isolated yields in brackets.

Table S12: Photocatalyst screening in flow

- Stoichiometry screening

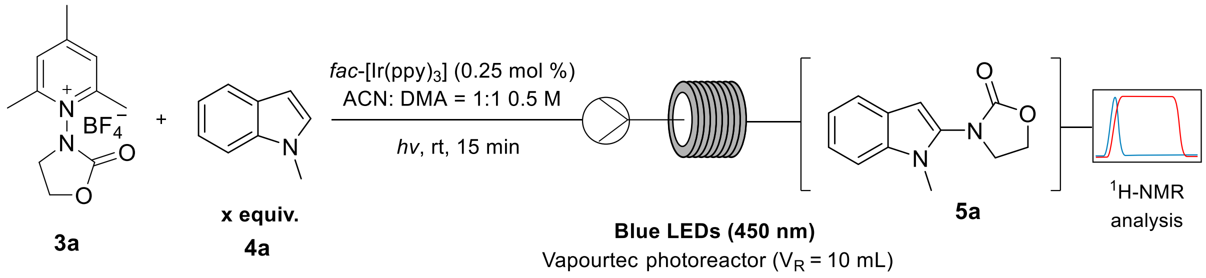

ENTRY	4a EQUIV.	YIELD (%)[a]
**1**	1	47
**2**	1.5	54
**3**	2	72 (**80**)
**4**	3	76
**5**	4	79
**6**	6	82
**7**	10	84

[a] Yields determined by ^{1}H-NMR analysis, using 1,1,2,2- tetrachloroethane as standard; isolated yields in brackets.

Table S13: Stoichiometry screening in flow

The study was conducted by slowly increasing the equivalents of 1-methyl-1H-indole **4a** up to 10. Considering the turning point within a correlation between the equivalents of 1-methyl-1H-indole **4a** and the corresponding ^{1}H-NMR yield when 2 equivalents are used (**Figure S11**), the result shown in entry 3 (80 % of isolated yield)

is still considered as optimized experiment in agreement with the green chemistry statements, even though higher values could be achieved in further increasing the amount of the substrate.

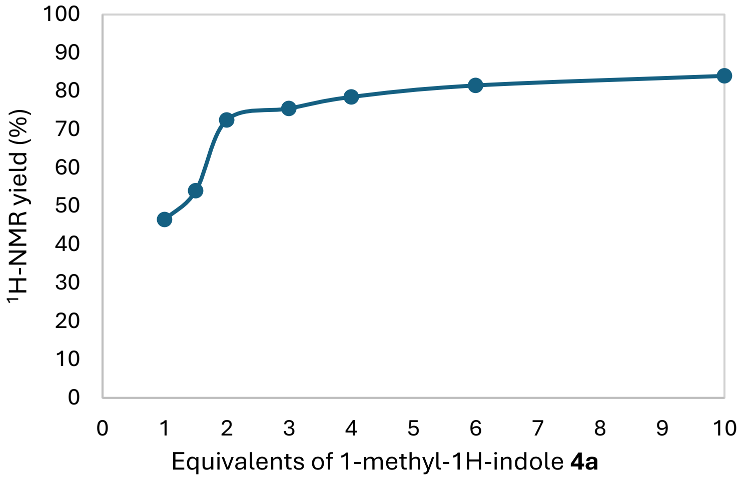

Figure S11: Correlation between equivalents of 1-methyl-1H-indole **4a** and ^{1}H-NMR yield

To fully understand the nature of the process, compound **6a** was identified as the only side-product recovered. It was formed due to a double radical attack onto the heteroaromatic substrate **4a** (**Figure S12**) and obtained in higher amount as 1-methyl-1H- indole equivalents decrease.

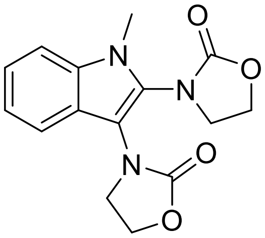

6a

Figure S12: Side-product obtained from the photocatalytic reaction

- Counter ion screening

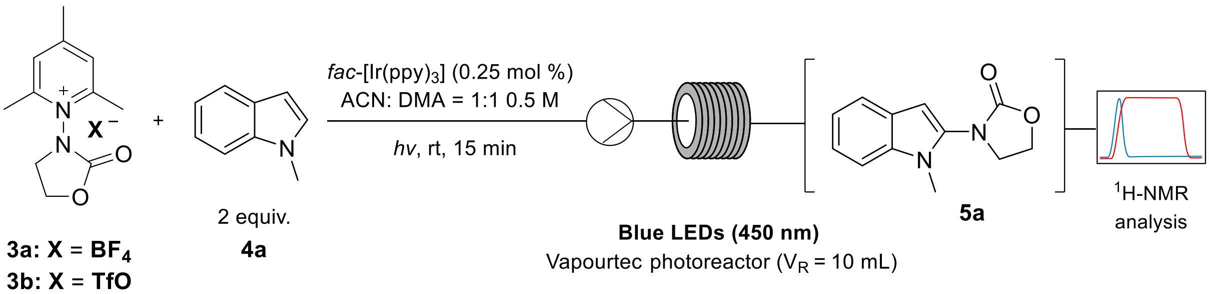

ENTRY	COUNTER ION	YIELD (%)[a]
**1**	BF_4	72 (**80**)
**2**	TfO	58

[a] Yields determined by ^{1}H-NMR analysis, using 1,1,2,2- tetrachloroethane as standard; isolated yields in brackets.

Table S14: Counter ion screening in flow

4.3. Control experiments

Different control experiments were performed without the iridium-base photocatalyst and in the dark, both at room temperature (25 °C) and heating a vial for 16 h through an oil bath at 80 °C (**Table S15**).

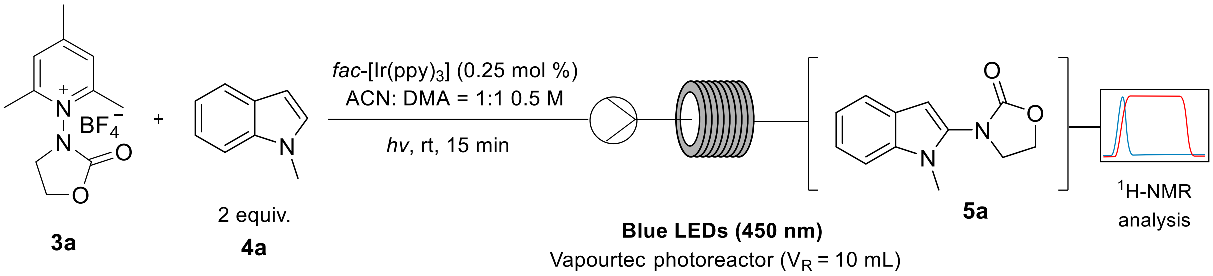

ENTRY	NOTES	YIELD (%)
**1**	no *fac*-[Ir(ppy)$_3$]	< 3
**2**	no light	< 1
**3***	no light, 80 °C	< 1

* reaction performed in batch, using the optimized condition for the batch.

Table S15: Control experiments

5. Reaction scope

5.1. Substrate scope in batch and in flow

General procedure for the photocatalytic addition of oxazolidinone radicals to arenes and heteroarenes in BATCH (GP5).

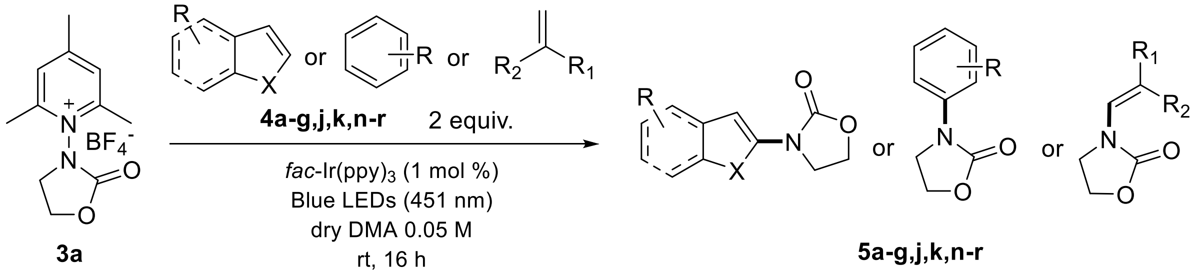

Two 7 mL vials for each reaction, were charged with 36.8 mg of the nitrogen radical precursor **3a** (0.125 mmol, 1 equiv., 0.05 M in DMA), 0.82 mg of *fac*-Ir(ppy)3 (1 mol %) and the arene or heteroarene **4a-g,j,k,n-r** (0.25 mmol, 2 equiv.). The vials were sealed with a septum cap and three nitrogen-vacuum cycles were done. 2.5 mL of dry DMA were added to each vial and other three nitrogen-vacuum cycles were performed using the freeze pump thaw method. The reaction mixture was irradiated for 16 hours using the plate-based photoreactor. Then, the vials were combined and the solvent was removed under reduced pressure. The crude was purified by flash column chromatography to provide the arylated or heteroarylated oxazolidinone.

General procedure for the photocatalytic addition of oxazolidinone radicals to arenes and heteroarenes in FLOW (GP6).

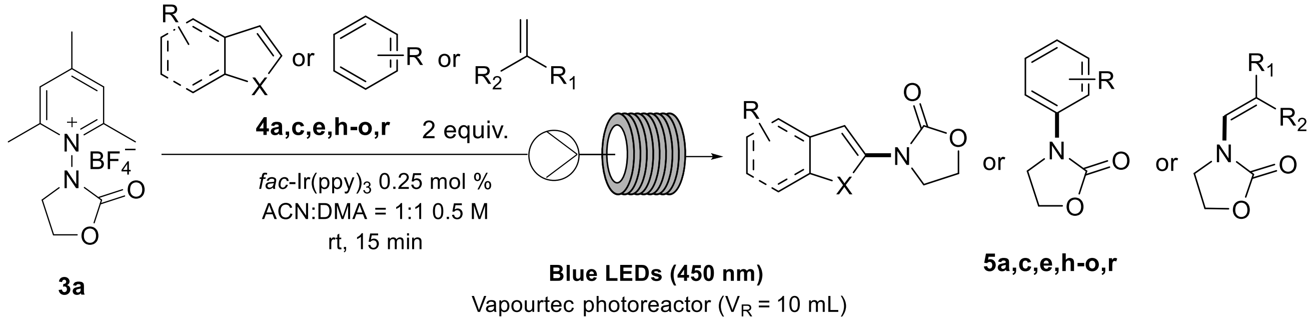

A microwave vial, was charged with 147 mg of the nitrogen radical precursor **3a** (0.5 mmol, 1 equiv., 0.5 in the solvent mixture ACN: DMA = 1:1), 0.82 mg of *fac*-Ir(ppy)3 (0.25 mol %) or 8.9 mg of 4CzIPN (2 mol %) and the arene or heteroarene **4a,c,e,h-o,r** (1 mmol, 2 equiv.). The vial was sealed with a septum cap and three nitrogen-vacuum cycles were done. 0.5 mL of dry DMA and 0.5 mL of dry acetonitrile were added and the reaction mixture was degassed for 5 minutes. The reaction was carried out under continuous flow conditions, irradiating the mixture for 15 minutes as residence time, in a Vapourtec photoreactor equipped with blue LEDs (450 nm, 24 W). After the collection, the solvent mixture was removed under reduced pressure and the crude was purified by flash column chromatography to provide the arylated or heteroarylated oxazolidinone.

3-(1-methyl-1H-indol-2-yl)oxazolidin-2-one (5a)

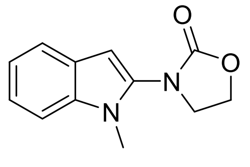

Starting from the commercially available 1-methyl-1H-indole **4a** (65.5 or 131 mg), 3-(1-methyl-1H-indol-2-yl)oxazolidin-2-one **5a** was prepared according to **GP5** and **GP6**. It was obtained in 74 % yield in batch (40 mg) and 80 % in flow (86 mg) as a pink solid after purification by flash column chromatography on silica gel (hexane: EtOAc = 8: 2 → 5: 5 or pentane: EtOAc = 75: 25 → 5: 5).

^{1}H NMR (300 MHz, $CDCl_3$) δ 7.61 (d, *J* = 7.8 Hz, 1H), 7.38 – 7.27 (m, 2H), 7.16 (ddd, *J* = 8.0, 6.9, 1.2 Hz, 1H), 6.43 (s, 1H), 4.61 (dd, *J* = 8.7, 7.0 Hz, 2H), 4.07 (dd, *J* = 8.7, 7.1 Hz, 2H), 3.72 (s, 3H). **^{13}C NMR** (101 MHz, $CDCl_3$) δ 156.8, 135.4, 133.0, 126.4, 122.3, 120.8, 120.3, 109.7, 95.5, 62.8, 48.8, 29.7. **IR (neat)** v/cm^{-1}: 2903 (w), 1751 (s), 1560 (m), 1407 (m), 1204 (m), 1102 (s), 1030 (m), 752 (s). **HRMS (QTOF)** m/z: $[M+H]^+$ Calcd for $C_{12}H_{13}N_2O_2^+$ 217.0972; Found 217.0972. **Rf** = 0.26 (pentane: EtOAc = 6: 4). **M. p.** = 147 °C – 149 °C.

3-(5-chloro-1-methyl-1H-indol-2-yl)oxazolidin-2-one (5b)

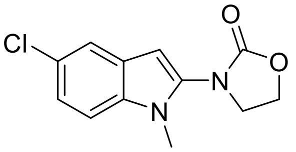

Starting from the synthesized 5-chloro-1-methyl-1H-indole **4b** (83 mg), 3-(5-chloro-1-methyl-1H-indol-2-yl) oxazolidin-2-one **5b** was prepared according to **GP5**. It was obtained in 52 % yield (32.5 mg) as a white solid after purification by flash column chromatography on silica gel (hexane: EtOAc = 8: 2 → 5: 5).

^{1}H NMR (300 MHz, $CDCl_3$) δ 7.53 (d, *J* = 1.5 Hz, 1H), 7.24 – 7.17 (m, 2H), 6.33 (s, 1H), 4.60 (dd, *J* = 8.7, 7.0 Hz, 2H), 4.06 (dd, *J* = 8.7, 7.0 Hz, 2H), 3.67 (s, 3H). **^{13}C NMR** (75 MHz, $CDCl_3$) δ 156.5, 134.2, 133.7, 127.2, 125.9, 122.5, 120.0, 110.6, 94.7, 62.8, 48.6, 29.9. **IR (neat)** v/cm^{-1}: 2960 (w), 2919 (w), 1748 (s), 1556 (m), 1471 (m), 1403 (m), 1332 (w), 1214 (m), 1110 (m), 1023 (m), 855 (w), 795 (w). **HRMS (ESI-TOF)** m/z: $[M+Na]^+$ Calcd for $C_{12}H_{11}ClN_2O_2Na$ 273.0401; Found 273.0400. **Rf** = 0.23 (pentane: EtOAc = 6: 4). **M. p.** = 177 °C – 180 °C.

3-(5-bromo-1-methyl-1H-indol-2-yl)oxazolidin-2-one (5c)

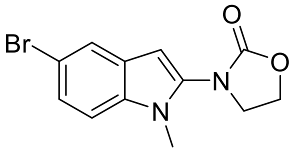

Starting from the synthesized 5-bromo-1-methyl-1H-indole **4c** (105 or 210 mg), 3-(5-bromo-1-methyl-1H-indol-2-yl) oxazolidin-2-one **5c** was prepared according to **GP5** and **GP6**. It was obtained in 51 % yield in batch (37.5 mg) and 67 % in flow (99 mg) as a white solid after purification by flash column chromatography on silica gel (hexane: EtOAc = 8: 2 → 5: 5 or pentane: EtOAc = 75: 25 → 5: 5).

^{1}H NMR (400 MHz, $CDCl_3$) δ 7.69 (d, *J* = 1.6 Hz, 1H), 7.32 (dd, *J* = 8.7, 1.9 Hz, 1H), 7.18 (d, *J* = 8.7 Hz, 1H), 6.32 (d, *J* = 0.8 Hz, 1H), 4.64 – 4.55 (m, 2H), 4.11 – 4.00 (m, 2H), 3.67 (s, 3H). **^{13}C NMR** (101 MHz, $CDCl_3$) δ 156.6, 134.2, 134.1, 128.0, 125.2, 123.2, 113.5, 111.2, 94.7, 62.9, 48.7, 30.0. **IR (neat)** v/cm^{-1}: 2922 (m), 2854 (w) 1751 (s), 1553 (w), 1469 (m), 1401 (m), 1213 (m), 1111 (m), 1030 (m), 793 (m). **HRMS (QTOF)** m/z: $[M+H]^+$ Calcd for $C_{12}H_{12}BrN_2O_2^+$ 295.0077; Found 295.0097. **Rf** = 0.24 (pentane: EtOAc = 6: 4). **M. p.** = 190 °C – 192 °C.

3-(1,5-dimethyl-1H-indol-2-yl)oxazolidin-2-one (5d)

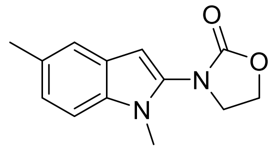

Starting from the synthetized 1,5-dimethyl-1H-indole **4d** (72.5 mg), 3-(1,5-dimethyl-1H-indol-2-yl)oxazolidin-2-one **5d** was prepared according to **GP5**. It was obtained in 52 % yield (30 mg) as a white solid after purification by flash column chromatography on silica gel (hexane: diethyl ether = 1:1 → 3: 7).

^{1}H NMR (300 MHz, $CDCl_3$) δ 7.35 (s, 1H), 7.19 (d, *J* = 8.4 Hz, 1H), 7.06 (d, *J* = 8.4 Hz, 1H), 6.30 (s, 1H), 4.57 (t, *J* = 7.9 Hz, 2H), 4.05 (t, *J* = 7.9 Hz, 2H), 3.66 (s, 3H), 2.43 (s, 3H). **^{13}C NMR** (75 MHz, $CDCl_3$) δ 156.7, 133.7, 132.9, 129.4, 126.5, 123.8, 120.3, 109.2, 94.8, 62.7, 48.7, 29.6, 21.4. **IR (neat)** ν/cm^{-1}: 2911 (w), 1742 (s), 1561 (w), 1470 (w), 1406 (w), 1217 (w), 1117 (w), 1030 (w), 791 (w). **HRMS (ESI-TOF)** m/z: $[M+Na]^+$ Calcd for $C_{13}H_{14}N_2O_2Na$ 253.0947; Found 253.0947. **Rf** = 0.18 (hexane: diethyl ether = 2: 8). **M. p.** = 133 °C – 135 °C.

3-(5-methoxy-1-methyl-1H-indol-2-yl)oxazolidin-2-one (5e)

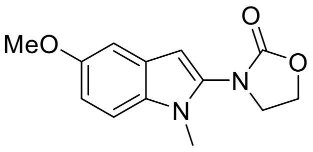

Starting from the commercially available 5-methoxy-1-methyl-1H-indole **4e** (80.5 or 161 mg), 3-(5-methoxy-1-methyl-1H-indol-2-yl)oxazolidin-2-one **5e** was prepared according to **GP5** and **GP6**. It was obtained in 70 % yield in batch (43 mg) and 76 % in flow (94 mg) as a beige solid after flash column chromatography on silica gel (hexane: EtOAc = 85: 15 → 1: 1 or pentane: Et_2O = 1: 1 → 2 : 8).

^{1}H NMR (400 MHz, $CDCl_3$) δ 7.20 (d, *J* = 8.9 Hz, 1H), 7.03 (d, *J* = 2.4 Hz, 1H), 6.90 (dd, *J* = 8.9, 2.5 Hz, 1H), 6.31 (s, 1H), 4.59 – 4.55 (m, 2H), 4.05 – 4.01 (m, 2H), 3.84 (s, 3H), 3.65 (s, 3H). **^{13}C NMR** (101 MHz, $CDCl_3$) δ 156.8, 154.6, 133.3, 130.7, 126.8, 112.6, 110.5, 102.6, 95.2, 62.8, 56.0, 48.8, 29.8. **IR (neat)** ν/cm^{-1}: 2921 (w), 1760 (m), 1746 (s), 1477 (m), 1405 (m), 1204 (s), 1139 (m), 1030 (m), 811 (m). **HRMS (QTOF)** m/z: $[M+H]^+$ Calcd for $C_{13}H_{15}N_2O_3^+$ 247.1077; Found 247.1081. **Rf** = 0.21 (pentane: Et_2O = 3: 7). **M. p.** = 170 °C – 172 °C.

1-methyl-2-(2-oxooxazolidin-3-yl)-1H-indole-3-carbonitrile (5f)

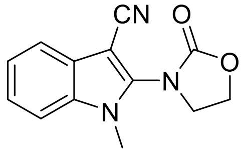

Starting from the synthetized 1-methyl-1H-indole-3-carbonitrile **4f** (78 mg), 1-methyl-2-(2-oxooxazolidin-3-yl)-1H-indole-3-carbonitrile **5f** was prepared according to **GP5**. It was obtained in 51 % yield (31 mg) as a white solid after purification by flash column chromatography on silica gel (Hexane: EtOAc = 8: 2 → 6: 4).

^{1}H NMR (300 MHz, $CDCl_3$) δ 7.68 (dt, *J* = 7.6, 1.2 Hz, 1H), 7.42 – 7.23 (m, 3H), 4.66 (dd, *J* = 8.8, 6.9 Hz, 2H), 4.32 (dd, *J* = 8.8, 6.9 Hz, 2H), 3.71 (s, 3H). **^{13}C NMR** (75 MHz, $CDCl_3$) δ 155.4, 138.9, 134.3, 125.9, 124.5, 123.0, 119.6, 114.8, 110.7, 80.5, 63.6, 46.9, 30.8. **IR (neat)** ν/cm^{-1}: 2922 (w), 2850 (w), 2214 (m), 1775 (s), 1560 (m), 1466 (m), 1447 (m), 1394 (m), 1214 (m), 1200 (m), 1144 (m), 1038 (m), 1025 (m), 806 (w), 745 (s), 731 (s). **HRMS (QTOF)** m/z: $[M+H]^+$ Calcd for $C_{13}H_{12}N_3O_2^+$ 242.0924; Found 242.0925. **Rf** = 0.17 (Hexane: EtOAc = 6: 4). **M. p.** = 152 °C – 154 °C.

Ethyl 1-methyl-2-(2-oxooxazolidin-3-yl)-1H-indole-3-carboxylate (5g)

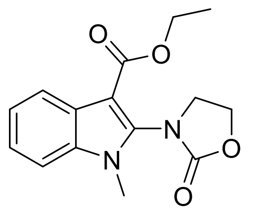

Starting from the synthetized ethyl 1-methyl-1H-indole-3-carboxylate **4g** (102 mg), ethyl 1-methyl-2-(2-oxooxazolidin-3-yl)-1H-indole-3-carboxylate **5g** was prepared according to **GP5**. It was obtained in 56 % yield (40.5 mg) as a white solid after purification by flash column chromatography on silica gel (Hexane: EtOAc = 8: 2 → 58: 42).

^{1}H NMR (300 MHz, $CDCl_3$) δ 8.17 (dd, *J* = 6.9, 1.7 Hz, 1H), 7.45 – 7.18 (m, 3H), 4.80 – 4.52 (m, 2H), 4.40 (q, *J* = 7.1 Hz, 2H), 4.32 (q, *J* = 8.3 Hz, 1H), 3.85 (q, *J* = 8.0 Hz, 1H), 3.72 (s, 3H), 1.45 (t, *J* = 7.1 Hz, 3H). **^{13}C NMR** (75 MHz, $CDCl_3$) δ 164.1, 157.2, 137.6, 134.5, 125.2, 123.8, 122.6, 122.3, 110.1, 101.8, 63.6, 60.1, 47.5, 29.3, 14.6. **IR (neat)** v/cm^{-1}: 2923 (w), 2851 (w), 1756 (s), 1686 (s), 1552 (m), 1474 (m), 1444 (m), 1289 (w), 1182 (s), 1131 (s), 1097 (s), 1032 (m), 1018 (m), 974 (w), 738 (m), 722 (m). **HRMS (QTOF)** m/z: $[M+H]^+$ Calcd for $C_{15}H_{17}N_2O_4^+$ 289.1183; Found 289.1183. **Rf** = 0.15 (Hexane: EtOAc = 6: 4). **M. p.** = 136 °C – 138 °C.

3-(1-phenyl-1H-indol-2-yl)oxazolidin-2-one (5h)

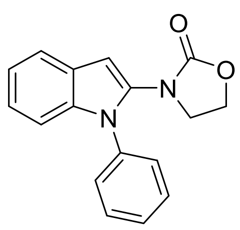

Starting from the synthesized 1-phenyl-1H-indole **4h** (193 mg), 3-(1-phenyl-1H-indol-2-yl) oxazolidin-2-one **5h** was prepared according to **GP6**. It was obtained in 72 % yield (100 mg) as a white-beige solid after purification by flash column chromatography on silica gel (cyclohexane: EtOAc = 8: 2 → 6: 4).

^{1}H NMR (400 MHz, $CDCl_3$) δ 7.69 – 7.61 (m, 1H), 7.55 (ddt, *J* = 9.4, 6.9, 2.2 Hz, 2H), 7.49 – 7.44 (m, 3H), 7.24 – 7.13 (m, 3H), 6.66 (s, 1H), 4.37 – 4.18 (m, 2H), 3.67 – 3.50 (m, 2H). **^{13}C NMR** (101 MHz, $CDCl_3$) δ 157.2, 136.5, 136.0, 132.4, 129.85 (2C), 128.3, 127.29 (2C), 126.6, 123.1, 121.2, 121.0, 110.5, 99.7, 62.5, 48.1. **IR (neat)** v/cm^{-1}: 2962 (w), 1746 (s), 1594 (w), 1453 (m), 1259 (m), 1228 (m), 1078 (m), 1016 (s), 796 (s), 745 (m), 695 (m). **HRMS (QTOF)** m/z: $[M+H]^+$ Calcd for $C_{17}H_{15}N_2O_2^+$ 279.1128; Found 279.1131. **Rf** = 0.19 (cyclohexane: EtOAc = 8: 2); 0.46 (cyclohexane: EtOAc = 6: 4). **M. p.** = 150 °C – 152 °C.

3-(5-bromo-1H-indol-2-yl)oxazolidin-2-one (5i)

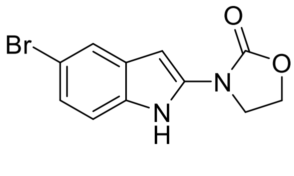

Starting from the commercially available 5-bromo-1H-indole **4i** (196 mg), 3-(5-bromo-1H-indol-2-yl)oxazolidin-2-one **5i** was prepared according to **GP6**. It was obtained in 9 % yield (12.5 mg) as a white-beige solid after purification by flash column chromatography on silica gel (pentane: DCM = 4: 6 → 3: 7).

^{1}H NMR (400 MHz, CD_3CN) δ 10.25 (br, s, 1H), 7.60 (d, *J* = 2.0 Hz, 1H), 7.34 (d, *J* = 8.5 Hz, 1H), 7.16 (dd, *J* = 8.6, 2.0 Hz, 1H), 5.84 (dd, *J* = 2.2, 0.8 Hz, 1H), 4.58 – 4.50 (m, 2H), 4.07 – 4.00 (m, 2H). **^{13}C NMR** (151 MHz, $CDCl_3$) δ 155.3, 135.3, 131.7, 128.9, 123.9, 121.8, 113.5, 112.2, 84.8, 62.8, 44.2. **IR (neat)** v/cm^{-1}: 3361 (w), 1730 (s), 1581 (m), 1412 (m), 1248 (m), 1123 (m), 1031 (m), 797 (s), 654 (m). **HRMS (QTOF)** m/z: $[M+H]^+$ Calcd for $C_{11}H_{10}BrN_2O_2^+$ 280.9920; Found 280.9920. **Rf** = 0.19 (pentane: DCM = 4: 6). **M. p.** = 214 °C – 216 °C.

3-(1H-indol-2-yl)oxazolidin-2-one (5j)

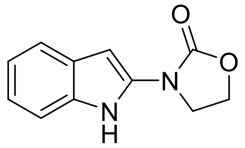

Starting from the commercially available 1H-indole **4j** (58.5 or 117 mg), 3-(1H-indol-2-yl)oxazolidin-2-one **5j** was prepared according to **GP5** and **GP6**. It was obtained in 6 % yield in batch (3 mg) and 21 % in flow (21 mg) as a beige solid after flash column chromatography on silica gel purification (hexane: EtOAc = 85: 15 → 55: 45 or cyclohexane: EtOAc = 8: 2 → 6 : 4).

^{1}H NMR (400 MHz, $CDCl_3$) δ 9.94 (br, s, 1H), 7.55 – 7.44 (m, 1H), 7.34 (dd, *J* = 7.8, 1.2 Hz, 1H), 7.19 – 7.05 (m, 2H), 5.78 (dd, *J* = 2.2, 0.9 Hz, 1H), 4.69 – 4.55 (m, 2H), 4.16 – 4.03 (m, 2H). **^{13}C NMR** (101 MHz, $CDCl_3$) δ 155.4, 134.5, 133.2, 127.2, 121.3, 120.5, 119.4, 110.9, 85.3, 62.9, 44.5. **IR (neat)** v/cm^{-1}: 3359 (w), 1729 (s), 1587 (m), 1411 (m), 1225 (m), 1118 (m), 1025 (m), 763 (m), 741 (s), 682 (m). **HRMS (QTOF)** m/z: $[M+H]^+$ Calcd for $C_{11}H_{11}N_2O_2^+$ 203.0815; Found 203.0817. **Rf** = 0.26 (cyclohexane: EtOAc = 7: 3). **M. p.** = 236 °C – 239 °C.

3-(1-methyl-1H-pyrrol-2-yl)oxazolidin-2-one (5k)

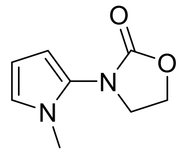

Starting from the commercially available 1-methylpyrrole **4k** (40.5 or 81 mg), 3-(1-methyl-1H-pyrrol-2-yl)oxazolidin-2-one **5k** was prepared according to **GP5** and **GP6**. It was obtained in 66 % yield in batch (27.5 mg) - just degassing the reaction mixture according to the volatility of **4k** - and 69 % in flow (57 mg) as a yellow oil after purification by flash column chromatography on silica gel (hexane: EtOAc = 8: 2 → 5: 5 or cyclohexane: EtOAc = 75: 25 → 55 : 45).

^{1}H NMR (400 MHz, $CDCl_3$) δ 6.54 (dd, *J* = 3.0, 1.9 Hz, 1H), 6.09 (dd, *J* = 3.8, 3.0 Hz, 1H), 6.03 (dd, *J* = 3.8, 1.9 Hz, 1H), 4.55 – 4.42 (m, 2H), 3.94 – 3.84 (m, 2H), 3.53 (s, 3H). **^{13}C NMR** (101 MHz, $CDCl_3$) δ 157.4, 125.7, 120.5, 107.0, 103.6, 62.6, 48.8, 33.1. **IR (neat)** v/cm^{-1}: 2876 (w), 1742 (s), 1562 (m), 1494 (m), 1400 (m), 1258 (m), 1214 (m), 1118 (m), 1033 (m), 763 (m), 710 (s). **HRMS (QTOF)** m/z: $[M+H]^+$ Calcd for $C_8H_{11}N_2O_2^+$ 167.0815; Found 167.0817. **Rf** = 0.24 (cyclohexane: EtOAc = 6: 4).

3-(1-phenyl-1H-pyrrol-2-yl)oxazolidin-2-one (5l)

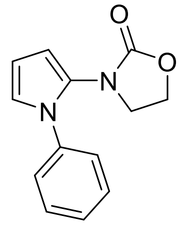

Starting from the synthetized 1-phenypyrrole **4l** (143 mg), 3-(1-phenyl-1H-pyrrol-2-yl)oxazolidin-2-one **5l** was prepared according to **GP6**. It was obtained in 70 % yield (80 mg) as an orange solid after purification by flash column chromatography on silica gel (cyclohexane: EtOAc = 85: 15 → 6 : 4).

^{1}H NMR (400 MHz, $CDCl_3$) δ 7.50 – 7.41 (m, 2H), 7.38 (ddd, *J* = 6.1, 3.2, 1.8 Hz, 3H), 6.80 (dd, *J* = 2.9, 2.1 Hz, 1H), 6.27 (dd, *J* = 2.5, 1.2 Hz, 2H), 4.31 – 4.19 (m, 2H), 3.60 – 3.46 (m, 2H). **^{13}C NMR** (101 MHz, $CDCl_3$) δ 157.9, 138.6, 129.61 (2C), 127.8, 125.3, 125.19 (2C), 121.2, 108.5, 107.0, 62.4, 48.5. **IR (neat)** v/cm^{-1}: 2923 (w), 1739 (s), 1499 (m), 1403 (m), 1221 (m), 1123 (m), 1033 (m), 757 (m), 690 (s), 556 (m). **HRMS (QTOF)** m/z: $[M+H]^+$ Calcd for $C_{13}H_{13}N_2O_2^+$ 229.0972; Found 229.0974. **Rf** = 0.26 (cyclohexane: EtOAc = 7: 3). **M. p.** = 102 °C – 104 °C.

3-(benzofuran-2-yl)oxazolidin-2-one (5m)

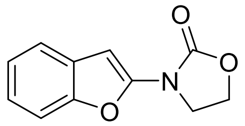

Starting from the commercially available 2,3-benzofuran **4m** (118 mg), 3-(benzofuran-2-yl)oxazolidin-2-one **5m** was prepared according to **GP6**. It was obtained in 24 % yield (24 mg) as a white solid after flash column chromatography on silica gel purification (cyclohexane: EtOAc = 8: 2 → 78 : 22).

^{1}H NMR (400 MHz, $CDCl_3$) δ 7.56 – 7.45 (m, 1H), 7.38 (ddd, *J* = 7.2, 1.9, 0.8 Hz, 1H), 7.25 – 7.16 (m, 2H), 6.66 (d, *J* = 0.9 Hz, 1H), 4.64 – 4.49 (m, 2H), 4.31 – 4.17 (m, 2H). **^{13}C NMR** (101 MHz, $CDCl_3$) δ 153.8, 150.6, 147.8, 129.3, 123.6, 122.9, 120.6, 110.5, 89.7, 62.8, 44.2. **IR (neat)** v/cm^{-1}: 2923 (w), 1748 (m), 1598 (s), 1455 (m), 1412 (s), 1210 (s), 1111 (m), 1014 (m),789 (m), 746 (m). **HRMS (QTOF)** m/z: $[M+H]^+$ Calcd for $C_{11}H_{10}NO_3^+$ 204.0655; Found 204.0656. **Rf** = 0.34 (cyclohexane: EtOAc = 75: 25). **M. p.** = 145 °C – 146 °C.

3-(anthracen-9-yl)oxazolidin-2-one (5n)

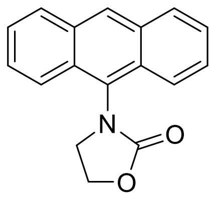

Starting from the commercially available anthracene **4n** (89 or 178 mg), 3-(anthracen-9-yl)oxazolidin-2-one **5n** was prepared according to **GP5** and **GP6**. It was obtained in 73 % yield in batch (48 mg) and 48 % in flow (63 mg) as a white-beige solid after purification by flash column chromatography on silica gel (hexane: EtOAc = 9: 1 → 65: 35 or cyclohexane: EtOAc = 75: 25 → 7: 3).

^{1}H NMR (400 MHz, $CDCl_3$) δ 8.52 (s, 1H), 8.11 – 8.04 (m, 2H), 8.00 (dq, *J* = 8.8, 1.0 Hz, 2H), 7.59 (ddd, *J* = 8.8, 6.6, 1.3 Hz, 2H), 7.51 (ddd, *J* = 7.9, 6.6, 1.2 Hz, 2H), 4.87 – 4.77 (m, 2H), 4.21 – 4.10 (m, 2H). **^{13}C NMR** (101 MHz, $CDCl_3$) δ 157.7, 132.08 (2C), 129.24 (2C), 129.01 (2C), 128.5, 128.1, 127.46 (2C), 125.70 (2C), 122.20 (2C), 63.0, 48.4. **IR (neat)** v/cm^{-1}: 1746 (s), 1422 (m), 1238 (m), 1077 (m), 1036 (m), 913 (m), 791 (m), 742 (m). **HRMS (QTOF)** m/z: $[M+H]^+$ Calcd for $C_{17}H_{14}NO_2^+$ 264.1019; Found 264.1021. **Rf** = 0.20 (cyclohexane: EtOAc = 7: 3). **M. p.** = 252 °C – 255 °C.

3-(2,2-diphenylvinyl)oxazolidin-2-one (5o)

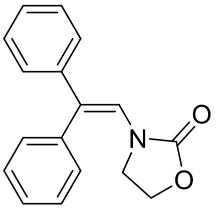

Starting from the commercially available 1,1-diphenylethylene **4o** (180 or 90 mg), 3-(2,2-diphenylvinyl)oxazolidin-2-one **5o** was prepared according to **GP5** and **GP6**. It was obtained in 53 % yield in batch (35 mg). For the flow approach, it was obtained in 67 % yield using *fac*-Ir(ppy)$_3$ as photocatalyst (88.5 mg), and 77 % yield using 4CzIPN as photocatalyst (102 mg) as a white solid after purification by flash column chromatography on silica gel (cyclohexane: EtOAc = 95: 5 → 7: 3).

^{1}H NMR (400 MHz, $CDCl_3$) δ 7.42 – 7.31 (m, 3H), 7.28 – 7.23 (m, 5H), 7.20 – 7.16 (m, 2H), 7.14 (s, 1H), 4.24 – 4.12 (m, 2H), 3.19 – 3.07 (m, 2H). **^{13}C NMR** (101 MHz, , $CDCl_3$) δ 157.4, 141.0, 138.2, 131.02 (2C), 128.39 (2C), 128.37 (2C), 128.0, 127.20 (2C), 127.2, 126.3, 122.6, 62.8, 45.0. **IR (neat)** v/cm^{-1}: 2923 (w), 1733 (s), 1633 (m), 1476 (m), 1406 (m), 1210 (m), 1087 (m), 1036 (m), 874 (m), 758 (s), 692 (s). **HRMS (QTOF)** m/z: $[M+H]^+$ Calcd for $C_{17}H_{16}NO_2^+$ 266.1176; Found 266.1178. **Rf** = 0.21 (cyclohexane: EtOAc = 8: 2). **M. p.** = 116 °C – 118 °C.

(Z) -3-(2-phenylprop-1-en-1-yl)oxazolidin-2-one (5p)

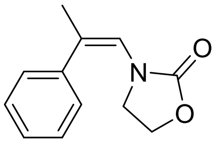

Starting from the commercially available 2-phenylpropene **4p** (59 mg), (Z)-3-(2-phenylprop-1-en-1-yl)oxazolidin-2-one **5p** was prepared according to **GP5**. It was obtained in 18 % yield (9 mg) as a white solid after purification by flash column chromatography on silica gel (Hexane: EtOAc = 92: 8 → 8: 2).

^{1}H NMR (300 MHz, $CDCl_3$) δ 7.38 – 7.23 (m, 5H), 6.56 (q, *J* = 1.5 Hz, 1H), 4.21 – 4.10 (m, 2H), 3.16 – 3.05 (m, 2H), 2.07 (d, *J* = 1.5 Hz, 3H). **^{13}C NMR** (75 MHz, $CDCl_3$) δ 157.3, 140.1, 128.44 (2C), 128.19 (2C), 127.4, 122.9, 120.4, 62.5, 45.0, 23.8. **IR (neat)** v/cm^{-1}: 2965 (w), 2916 (w), 1739 (s), 1654 (w), 1472 (w), 1413 (m), 1307 (w), 1257 (m), 1068 (m), 1035 (m), 753 (s), 695 (s), 608 (m). **HRMS (QTOF)** m/z: $[M+H]^+$ Calcd for $C_{12}H_{14}NO_2^+$ 204.1019; Found 204.1020. **Rf** = 0.33 (Hexane: EtOAc = 7: 3). **M. p.** = 71 °C – 72 °C.

(Z) -3-(4-(tert-butyl)styryl)oxazolidin-2-one (5q)

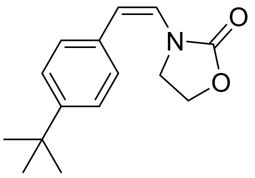

Starting from the commercially available 1-(tert-butyl)-4-vinylbenzene **4q** (80 mg), (Z)-3-(4-(tert-butyl)styryl)oxazolidin-2-one **5q** was prepared according to **GP5**. It was obtained in 21 % yield (13 mg) as a colorless oil after purification by flash column chromatography on silica gel (Hexane: EtOAc = 9: 1 → 84: 16).

^{1}H NMR (300 MHz, $CDCl_3$) δ 7.33 (d, *J* = 8.0 Hz, 2H), 7.14 (d, *J* = 8.1 Hz, 2H), 6.62 (d, *J* = 9.7 Hz, 1H), 5.96 (d, *J* = 9.7 Hz, 1H), 4.39 – 4.18 (m, 2H), 3.49 – 3.35 (m, 2H), 1.32 (s, 9H). **^{13}C NMR** (75 MHz, $CDCl_3$) δ 157.4, 150.4, 132.6, 129.08 (2C), 124.98 (2C), 124.0, 113.3, 62.8, 45.1, 34.7, 31.45 (3C). **IR (neat)** v/cm^{-1}: 2960 (w), 2868 (w), 1753 (s), 1652 (m), 1413 (m), 1393 (m), 1234 (m), 1213 (m), 1068 (m), 1037 (m), 827 (w), 757 (m), 733 (m). **HRMS (QTOF)** m/z: $[M+H]^+$ Calcd for $C_{15}H_{20}NO_2^+$ 246.1489; Found 246.1489. **Rf** = 0.37 (Hexane: EtOAc = 8: 2).

3-(2,4,6-trimethoxyphenyl)oxazolidin-2-one (5r)

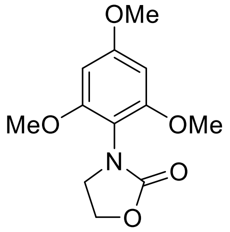

Starting from the commercially available 1,3,5-trimethoxybenzene **4r** (90 or 180 mg), 3-(2,4,6-trimethoxyphenyl)oxazolidin-2-one **5r** was prepared according to **GP5** and **GP6**. It was obtained in traces (< 5%) in batch and 20 % in flow (25 mg) as a white solid after purification by flash column chromatography on silica gel (DCM: MeOH = 98: 2 → 96: 4).

^{1}H NMR (300 MHz, $CDCl_3$) δ 6.13 (s, 2H), 4.47 (dd, *J* = 9.0, 7.2 Hz, 2H), 3.80 (d, *J* = 3.7 Hz, 11H). **^{13}C NMR** (75 MHz, $CDCl_3$) δ 161.1, 158.00 (2C), 157.7, 107.7, 91.23 (2C), 62.6, 56.09 (2C), 55.6, 46.5. **IR (neat)** v/cm^{-1}: 2926 (w), 2841 (w), 1739 (s), 1591 (m), 1514 (m), 1441 (m), 1416 (m), 1232 (m), 1211 (m), 1161 (m), 1119 (s), 1031 (m), 811 (m), 760 (m). **HRMS (ESI-TOF)** m/z: $[M+Na]^+$ Calcd for $C_{12}H_{15}NO_5Na$ 276.0842; Found 276.0846. **Rf** = 0.23 (DCM: MeOH = 98: 2). **M. p.** = 178 °C – 181 °C.

Characterization of side-products

3,3'-(1-methyl-1H-indole-2,3-diyl)bis(oxazolidin-2-one) (6a)

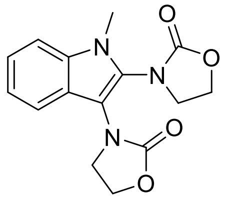

Starting from the commercially available 1-methyl-1H-indole **4a** (131 mg), 3,3'-(1-methyl-1H-indole-2,3-diyl)bis(oxazolidin-2-one) **6a** was recovered as the only side-product according to **GP4** and **GP6**. It was obtained in 10 % yield as a light-brown solid after purification by flash column chromatography on silica gel (pentane: EtOAc = 5: 5 → 1: 9).

^{1}H NMR (400 MHz, $CDCl_3$) δ 7.50 (dt, *J* = 7.9, 1.0 Hz, 1H), 7.36 – 7.26 (m, 2H), 7.18 (ddd, *J* = 8.0, 6.5, 1.6 Hz, 1H), 4.56 (dt, *J* = 11.3, 8.0 Hz, 4H), 4.08 (t, *J* = 8.1 Hz, 4H), 3.68 (s, 3H). **^{13}C NMR** (101 MHz, $CDCl_3$) δ 157.6, 157.1, 133.4, 128.2, 123.3, 122.4, 120.8, 117.9, 109.9, 108.0, 63.4, 62.9, 46.9, 46.8, 29.3. **IR (neat)** v/cm^{-1}: 2958 (w), 2922 (w), 1757 (s), 1783 (s), 1408 (m), 1249 (m), 1130 (m), 1068 (m), 1036 (m), 743 (s), 711 (m). **HRMS (QTOF)** m/z: $[M+H]^+$ Calcd for $C_{15}H_{16}N_3O_4^+$ 302.1135; Found 302.1137. **Rf** = 0.44 (pentane: EtOAc = 2: 8). **M. p.** = 184 °C – 186 °C.

5.2. Unsuccessful results

The photocatalytic reaction was unsuccessfully performed in batch for a small library of compounds.

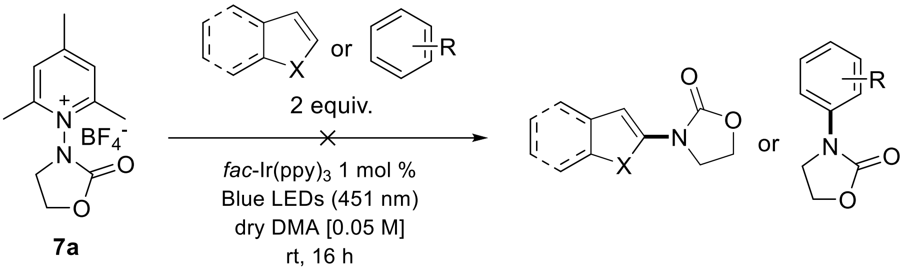

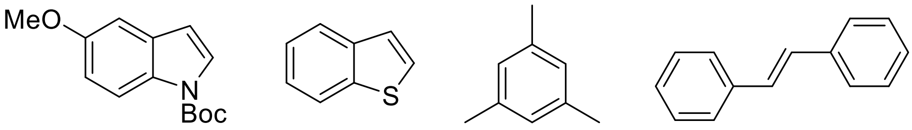

The photocatalytic reaction was unsuccessfully performed in flow for a small library of compounds.

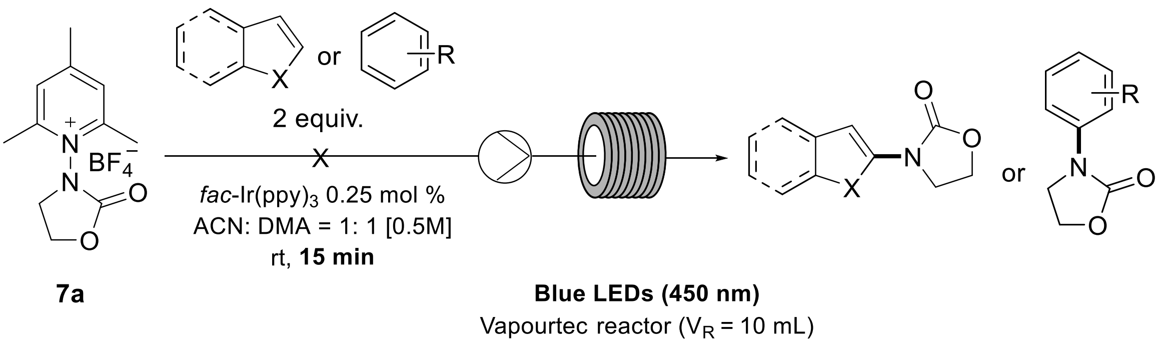

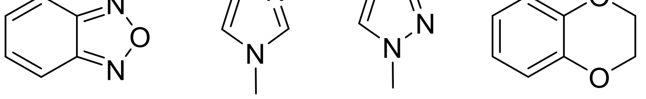

6. Gram-scale reaction under continuous flow conditions

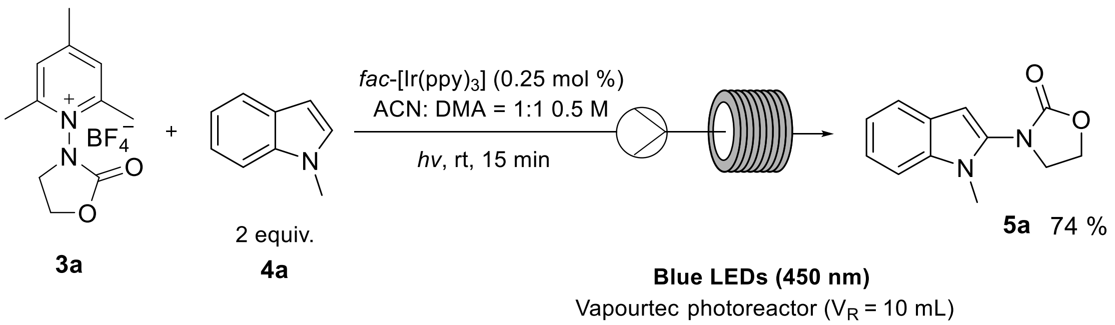

A round bottom V-flask, was charged with the nitrogen radical precursor **3a** (2.352 g, 8 mmol, 1 equiv., 0.5 M in ACN: DMA = 1:1), *fac*-Ir(ppy)3 (13.1 mg, 0.25 mol %) and 1-methyl-1H-indole **4a** (2.099 g, 16 mmol, 2 equiv.). Three nitrogen-vacuum cycles were done. 8 mL of dry acetonitrile and 8 mL of dry DMA were added and the reaction mixture was degassed for 30 minutes. The reaction was carried out under continuous flow conditions, irradiating the mixture for 15 minutes as residence time, with a Vapourtec photoreactor equipped with blue LEDs (450 nm, 24 W). After the collection, the solvent mixture was removed under reduced pressure and the crude was purified by flash column chromatography (petroleum ether: EtOAc = 8: 2 → 5: 5) to provide 3-(1-methyl-1H-indol-2-yl)oxazolidin-2-one **5a** as a pink solid (1.28 g, 74 %).

The set-up used for the gram-scale reaction is illustrated in **Figure S13**.

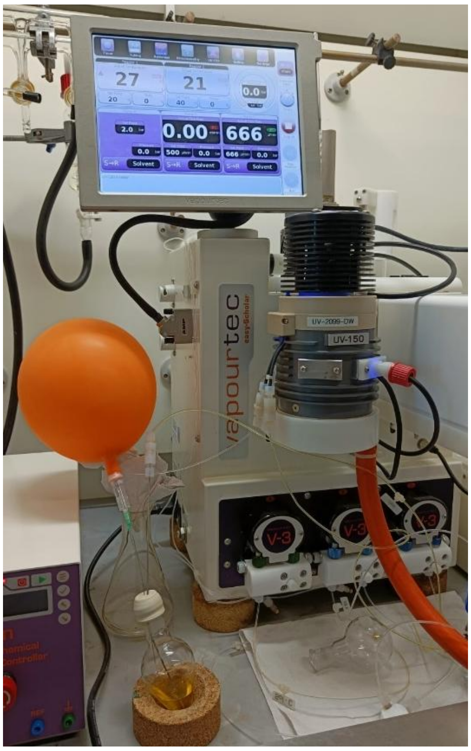

Figure S13: Set-up for the gram-scale photocatalytic reaction

6.1. Comparison between batch and flow approaches

To compare the batch and flow approaches, a kinetic study of the photocatalytic reaction has been conducted in batch using the optimized conditions obtained in flow. The combination of the reaction solvent with the high molarity of the reaction mixture allows to obtain a comparable result in 1 hour only, probably due to the more efficient light penetration (**Table S16**, **Figure S14**).

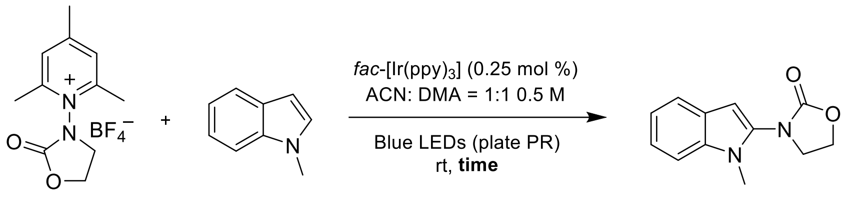

ENTRY	TIME (h)	YIELD (%)[a]
**1**	0.25	37
**2**	0.5	58
**3**	0.75	68
**4**	1	75
**5**	2	75
**6**	4	76
**7**	6	76

[a] Yields determined by ^{1}H-NMR analysis, using 1,1,2,2- tetrachloroethane as standard.

Table S16: Reaction time screening for the comparison between batch and flow approaches

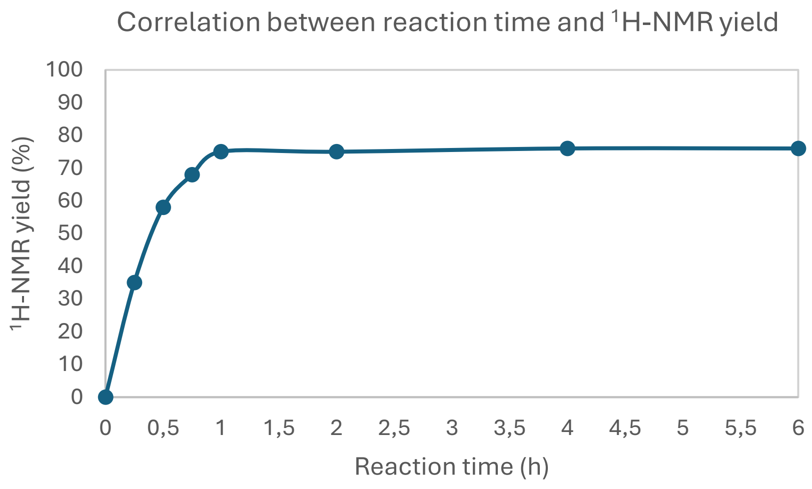

Figure S14: Correlation between reaction time and ^{1}H-NMR

The performances of the experiments under continuous flow conditions were evaluated in terms of productivity and space-time yield. The productivity is defined as the amount of product per hour provided by the system, while the space-time yield (STY) as the amount of product per hour, per unit of volume.

$$\textbf{\textit{Productivity}} = \frac{\text{mol limiting reagent } \cdot \text{yield } \cdot \text{MW product}}{\text{time (h)}}$$

$$\textbf{\textit{STY}} = \frac{\text{mol limiting reagent } \cdot \text{yield } \cdot \text{MW product}}{\text{time (h) } \cdot \text{volume (L)}}$$

The productivity and the space-time yield for the photocatalytic reaction have been calculated considering the same amount of starting material (0.5 mmol) as illustrated in **Table S17**.

	YIELD (%)	PRODUCTIVITY ($g \cdot h^{-1}$)	RELATIVE FACTOR	STY ($g \cdot h^{-1} \cdot L^{-1}$)	RELATIVE FACTOR
**BATCH 1**	74	$5 \cdot 10^{-3}$	-	0.5	-
**BATCH 2**	75	$8 \cdot 10^{-2}$	1	80	1
**FLOW**	80	$3.5 \cdot 10^{-1}$	**4.4**	350	**4.4**

Table S17: Productivity and STY calculated for the photocatalytic reaction.
BATCH 1: optimized conditions in batch (**GP3**, DMA 0.05 M, PC 1 mol %, 16 h)
BATCH 2: batch procedure performed with the optimized flow conditions
(dry DMA: ACN = 1: 1, 0.5 M, PC 0.25 mol %, 1 h)

For the gram-scale reaction, the productivity was calculated to be 3.2 g · h^{-1}; the space-time yield (STY) was calculated to be 200 $g \cdot h^{-1} \cdot L^{-1}$).

7. Stability studies

- Stability to light

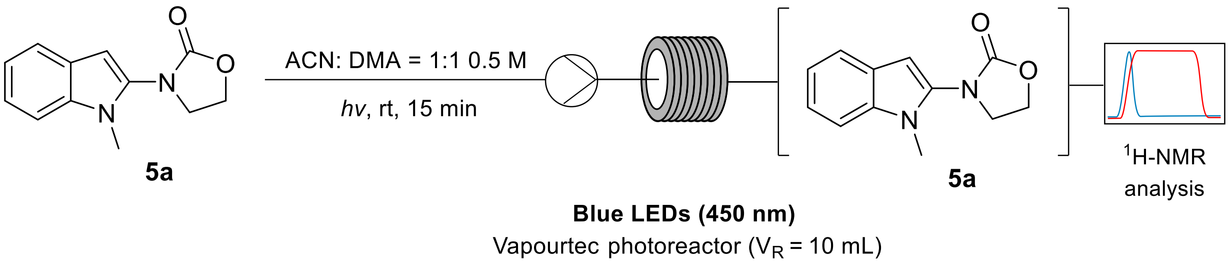

A 10 mL microwave vial was charged with 3-(1-methyl-1H-indol-2-yl)oxazolidin-2-one **5a** (54 mg, 0.25 mmol, 0.5 M in ACN: DMA = 1:1), 2.5 mL of dry ACN and 2.5 mL of dry DMA. The solution was irradiated for 15 min under continuous flow conditions, with a Vapourtec E-series photoreactor equipped with blue LEDs (450 nm, 24 W). Compound **5a** was fully recovered at the end of the experiment (53.8 mg). No degradation and colour change have been observed.

- Stability to oxygen

As shown in **Table S18**, the photocatalytic reaction can be performed at different concentrations both in the open air and under nitrogen atmosphere; additionally, no more effective oxygen removal methods, such as the freeze pump thaw method, are strictly required.

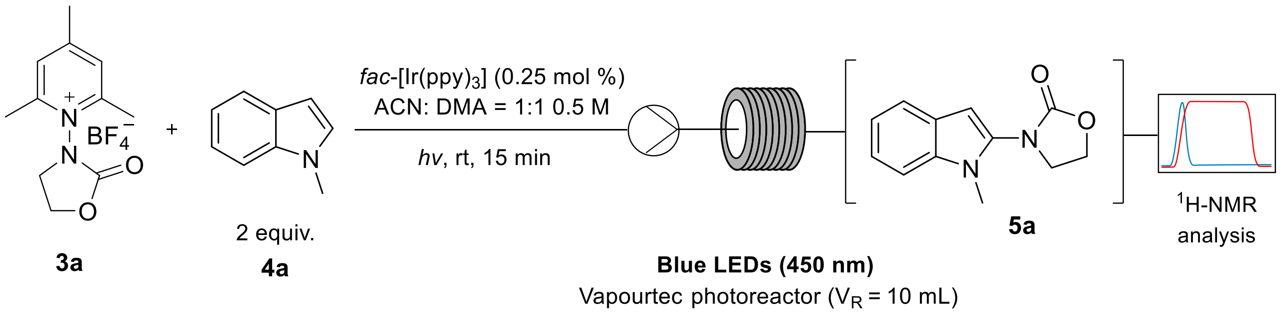

O_2 REMOVAL METHOD	MOLARITY (M)	YIELD (%) [a]
Freeze Pump Thaw	0.05	71 (77)
Degassing (N_2)	0.05	66
None	0.05	64 (71)
Freeze Pump Thaw	0.5	72 (80)
Degassing (N_2)	0.5	71 (78)
None	0.5	69

[a] Yields determined by ^{1}H-NMR analysis, using 1,1,2,2- tetrachloroethane as standard; isolated yields in brackets.

Table S18: Stability to oxygen study for the photocatalytic reaction

8. Mechanistic study

Stern-Volmer analyses were carried out in the open air with a Shimadzu RF-6000 Spectro Fluorophotometer, using a quartz cuvette with a path length of 1 cm, 450 nm as excitation wavelength and 465-800 nm as emission wavelength range. The measurements were carried out with the following setting: data interval = 1.0 nm, scan speed = 600 nm/min, excitation and emission bandwidth = 5.0 nm.

8.1. Stern-Volmer studies on *fac*-[Ir-(ppy)$_3$] fluorescence

Preparation of solutions

Solution **A** - photocatalyst *fac*-[Ir(ppy)$_3$]: a 2 mL volumetric flask was charged with 1.4 mg of *fac*-[Ir(ppy)$_3$]; the photocatalyst was dissolved in DMA, first diluting to the mark and then diluting 1:10 in a 20 mL volumetric flask to give a ~10^{-4} M solution of the photocatalyst.

Solution **A1** - quencher *N*-radical precursor **3a**: a 2 mL volumetric flask was charged with 58.8 mg of **3a** and a dilution to the mark with the solution of the photocatalyst was conducted to give a 0.1 M solution of quencher **3a** in a ~10^{-4} M solution of the photocatalyst.

Solution **A2** – quencher 1-methyl-1H-indole **4a**: a 2 mL volumetric flask was charged with 26.2 mg (25 mL) of **4a** and a dilution to the mark with the solution of the photocatalyst was conducted to give a 0.1 M solution of quencher **4a** in a ~10^{-4} M solution of the photocatalyst.

Recording of spectra

First, the photoluminescence spectrum of solution **A** was recorded. Then, an increasing amount of solution **A1** was added to study, through Stern-Volmer plot, the correlation between the quencher (*N*-radical precursor **3a**) concentration and I_0/I, which is the ratio between the fluorescence intensities in the absence of a quencher to the fluorescence intensities in its presence (**Figure S15**).

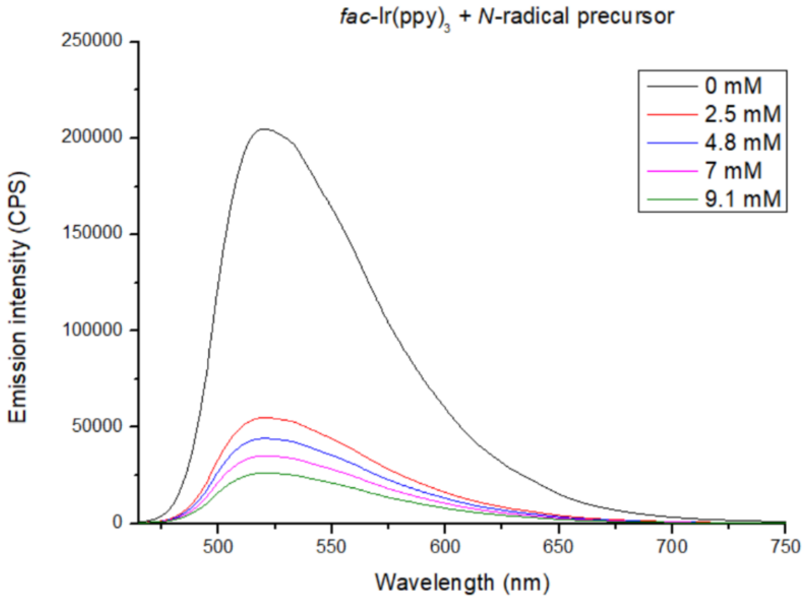

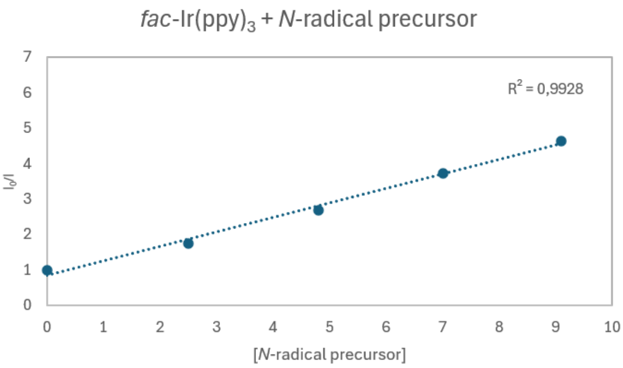

Figure S15: Quenching efficiency of precursor **3a** on the fluorescence of *fac*-Ir(ppy)$_3$ and Stern-Volmer plot

Also in this case, first the photoluminescence spectrum of solution **A** was recorded. Then, an increasing amount of solution **A2** was added to study the correlation between the quencher **4a** concentration and I_0/I in the Stern-Volmer plot (**Figure S16**).

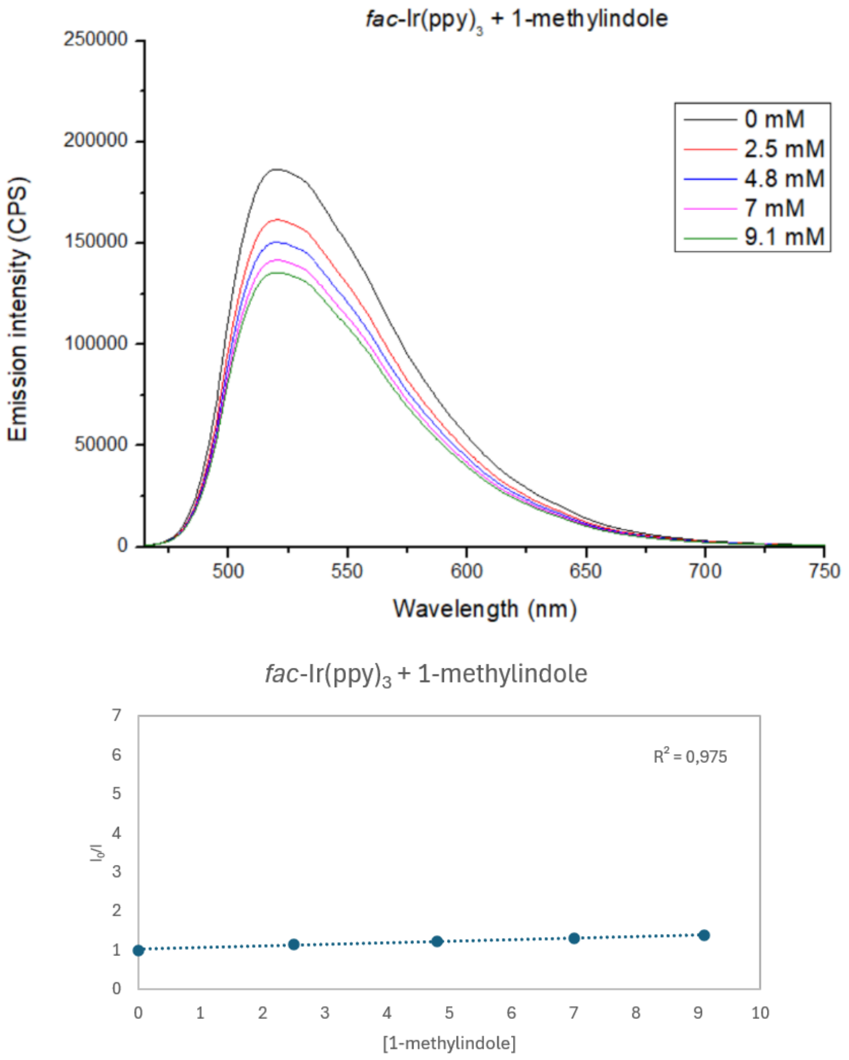

Figure S16: Quenching efficiency of 1-methyl-1H-indole **4a** on the fluorescence of *fac*-Ir(ppy)$_3$ and Stern-Volmer plot

8.2. Stern-Volmer studies on 4CzIPN

Preparation of solutions

Solution **B** - photocatalyst 4CzIPN: a 2 mL volumetric flask was charged with 1.6 mg of 4CzIPN; the photocatalyst was dissolved in DMA, first diluting to the mark and then diluting 1:10 in a 20 mL volumetric flask to give a ~10^{-4} M solution of the photocatalyst.

Solution **B1** - quencher *N*-radical precursor **3a**: a 2 mL volumetric flask was charged with 58.8 mg of **3a** and a dilution to the mark with the solution of the photocatalyst was conducted to give a 0.1 M solution of quencher **3a** in a ~10^{-4} M solution of the photocatalyst.

Solution **B2** – quencher 1-methyl-1H-indole **4a**: a 2 mL volumetric flask was charged with 26.2 mg (25 mL) of **4a** and a dilution to the mark with the solution of the photocatalyst was conducted to give a 0.1 M solution of quencher **4a** in a ~10^{-4} M solution of the photocatalyst.

Recording of spectra

First, the photoluminescence spectrum of solution **B** was recorded. Then, an increasing amount of solution **B1** was added to study the correlation between the quencher **3a** concentration and I_0/I (the ratio of the fluorescence intensities in the absence of a quencher to the fluorescence intensities in its presence) in the Stern-Volmer plot (**Figure S17**).

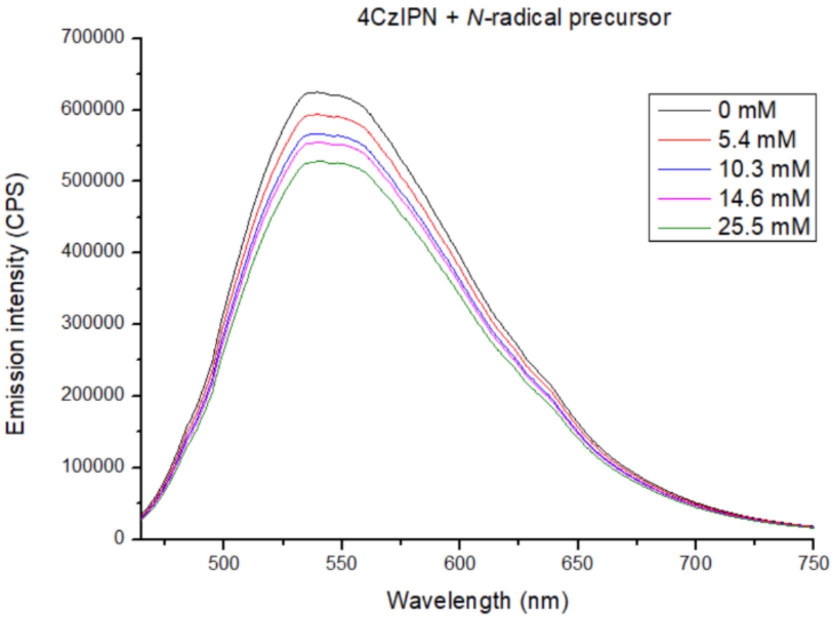

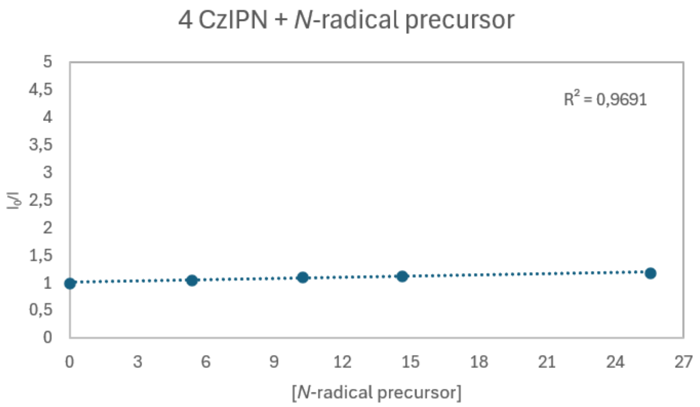

Figure S17: Quenching efficiency of precursor **3a** on the fluorescence of 4CzIPN and Stern-Volmer plot

Also in this case, first the photoluminescence spectrum of solution **A** was recorded. Then, an increasing amount of solution **A2** was added to study the correlation between the quencher **4a** concentration and I_0/I in the Stern-Volmer plot (**Figure S18**).

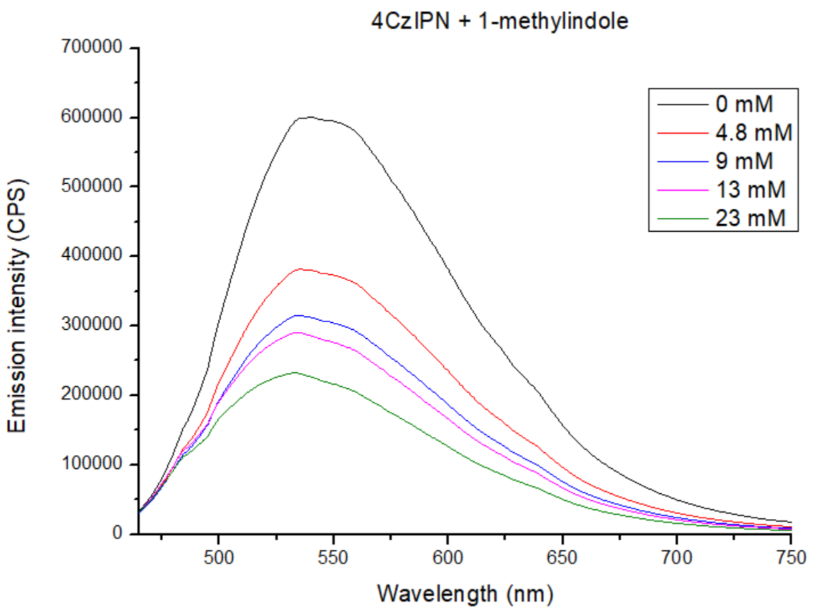

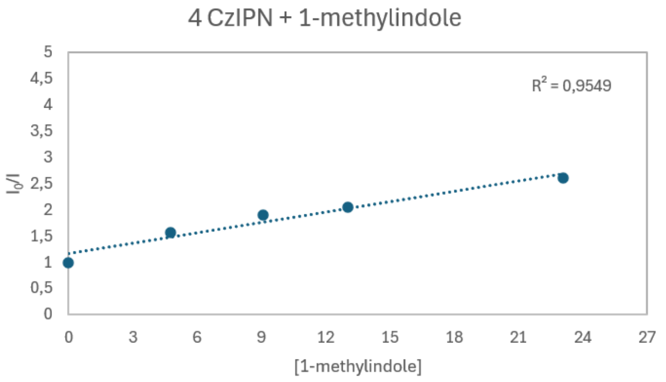

Figure S18: Quenching efficiency of 1-methyl-1H-indole **4a** on the fluorescence of 4CzIPN and Stern-Volmer plot

8.3. Comparison between *fac*-[Ir(ppy)$_3$] and 4CzIPN

Calculation of Stern-Volmer quenching constants

The Stern-Volmer quenching constants for the fluorescence of the photocatalysts, both in the presence of the *N*-radical precursor **3a** or the 1-methyl-1H-indole **4a** as quenchers have been calculated according to the Stern-Volmer equation for the dynamic (or collisional) quenching of fluorescence:

$$F_0/F = 1 + k_q\tau_0 [Q] = 1 + K_D [Q]$$

where F_0 and F (or I_0 and I) are respectively the fluorescence intensities in the absence and presence of a quencher; k_q is the bimolecular quenching constant; τ_0 is the lifetime of the fluorophore in the absence of the quencher; Q is the concentration of the quencher.

Considering that in dynamic quenching, the quenching data are usually presented as plots of I_0/I versus [Q], since a linearly correlation between the concentration of a quencher and the emission intensity is expected, the Stern-Volmer quenching constant, given by $K_D = k_q\tau_0$, is the slope of the plot F_0/F with an intercept of one.[19]

For *fac*-Ir(ppy)$_3$ fluorescence, these constants have been calculated to be: $K_D = 4.09 \times 10^{-1}$ M^{-1} for **3a** and $K_D = 4.04 \times 10^{-2}$ M^{-1} for **4a**. For 4CzIPN fluorescence: $K_D = 7.20 \times 10^{-3}$ M^{-1} for **3a** and $K_D = 6.59 \times 10^{-2}$ M^{-1} for **4a**.

Stern-Volmer plots

The photoluminescence lifetime of the photocatalysts linearly decreased with increasing concentrations of both the reagents, as the Stern-Volmer plot graphs confirm, but with an opposite quenching efficiency trend. The comparison between Stern-Volmer plots for the iridium-based and the metal-free photocatalysts is illustrated in **Figure S19**.

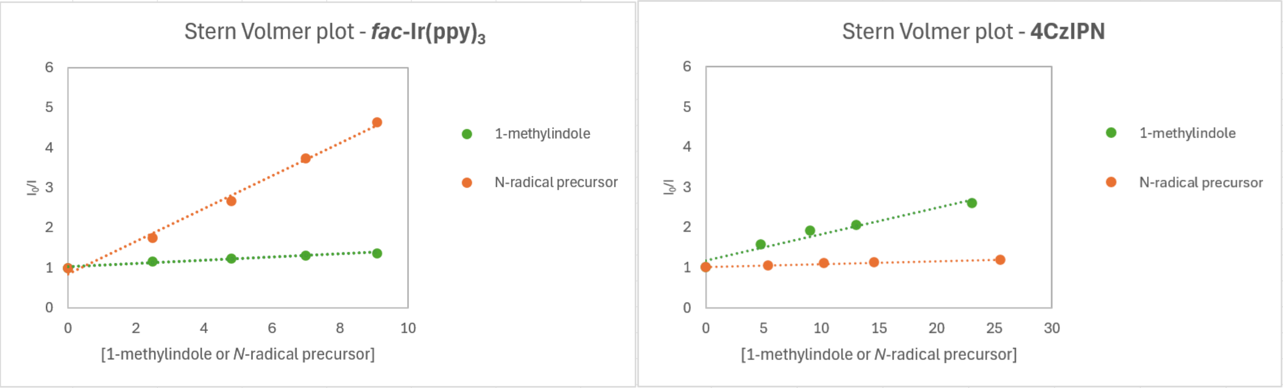

Figure S19: Stern-Volmer plots – quenching of the fluorescence of *fac*-Ir(ppy)$_3$ and 4CzIPN

9. NMR spectra

3-amino-2-oxazolidinone (1) **¹H-NMR** (300 MHz; $CDCl_3$)

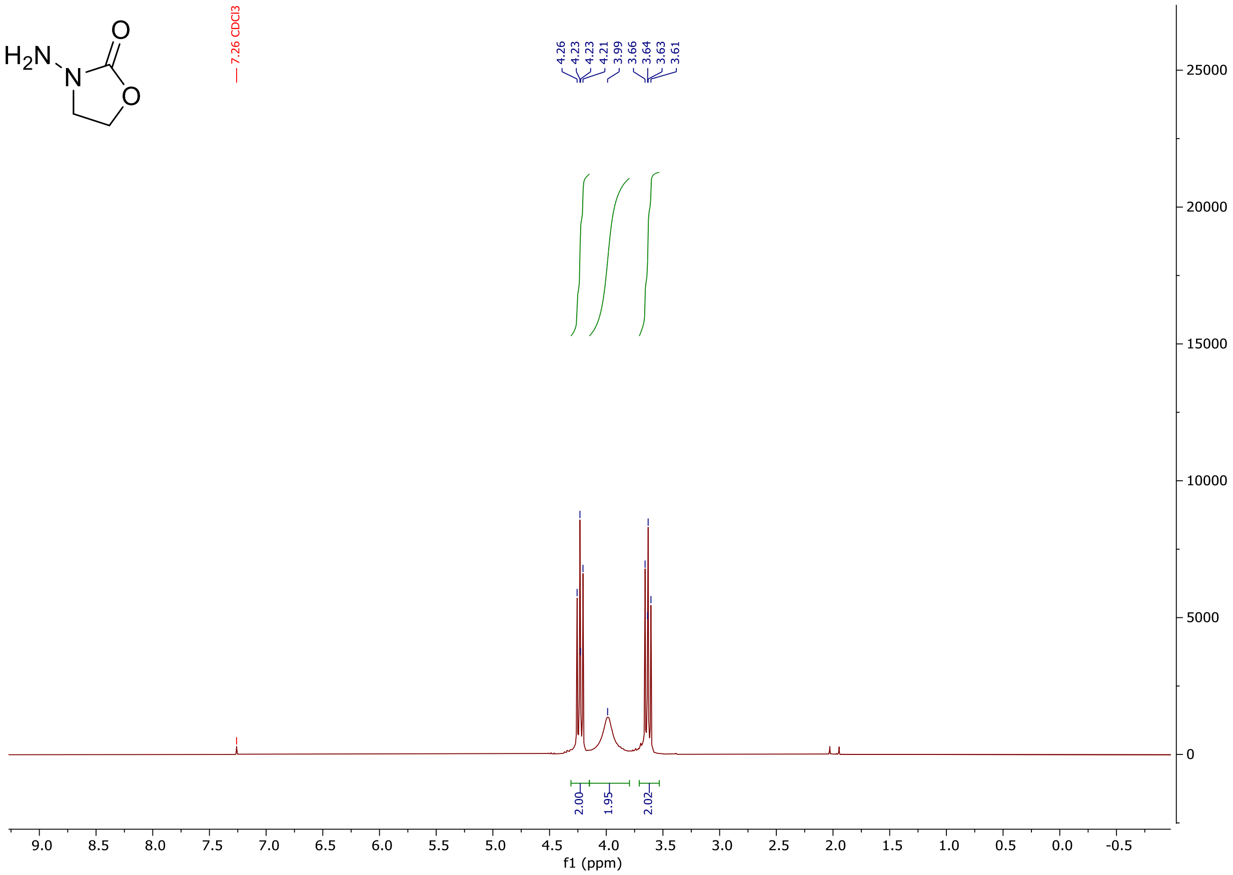

¹³C-NMR (75 MHz; $CDCl_3$)

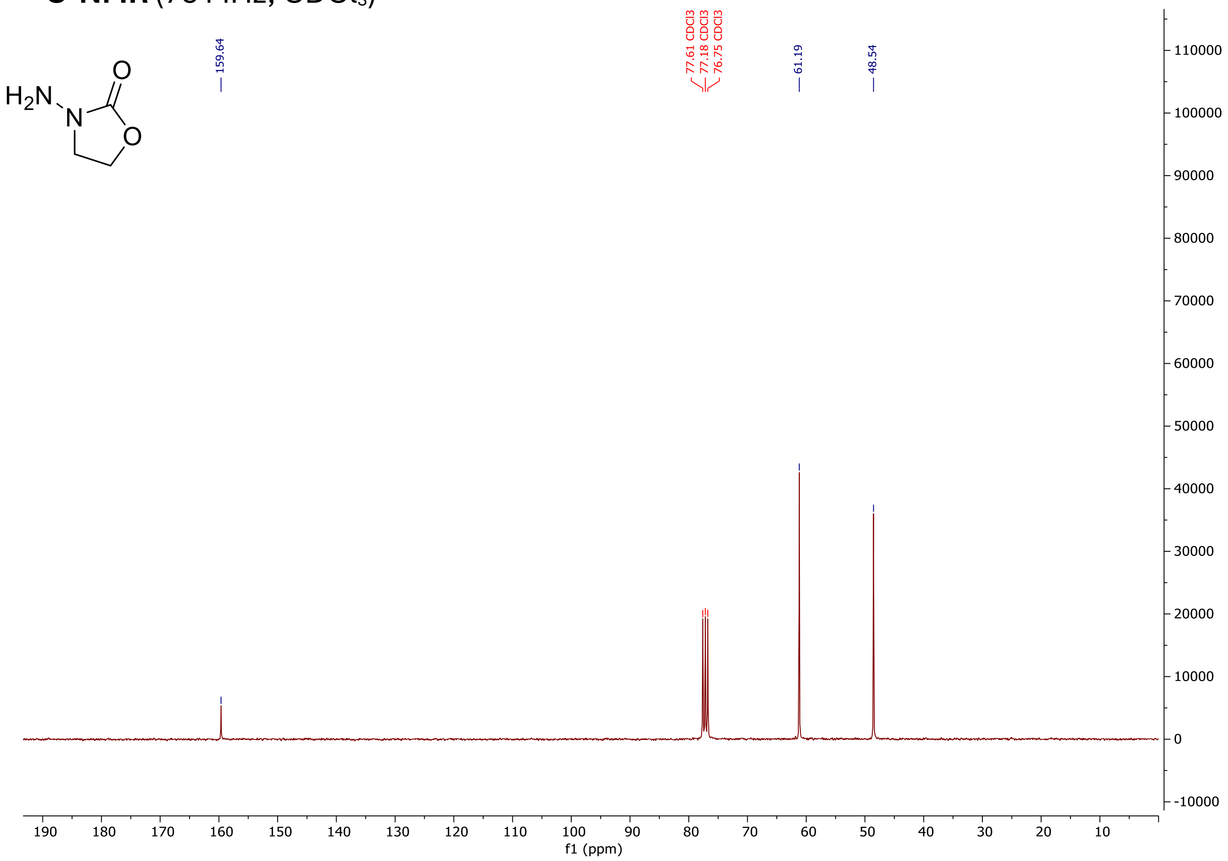

COSY ($CDCl_3$)

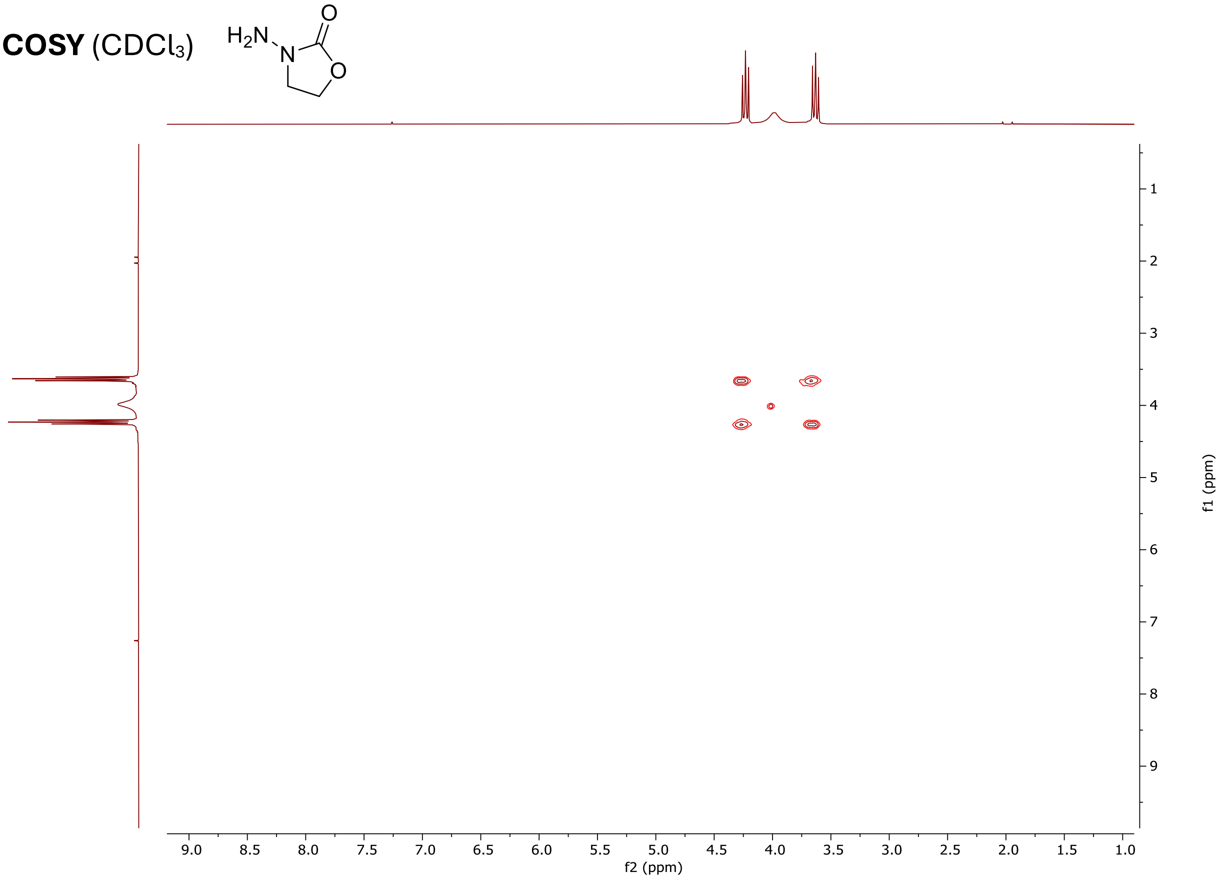

HSQC ($CDCl_3$)

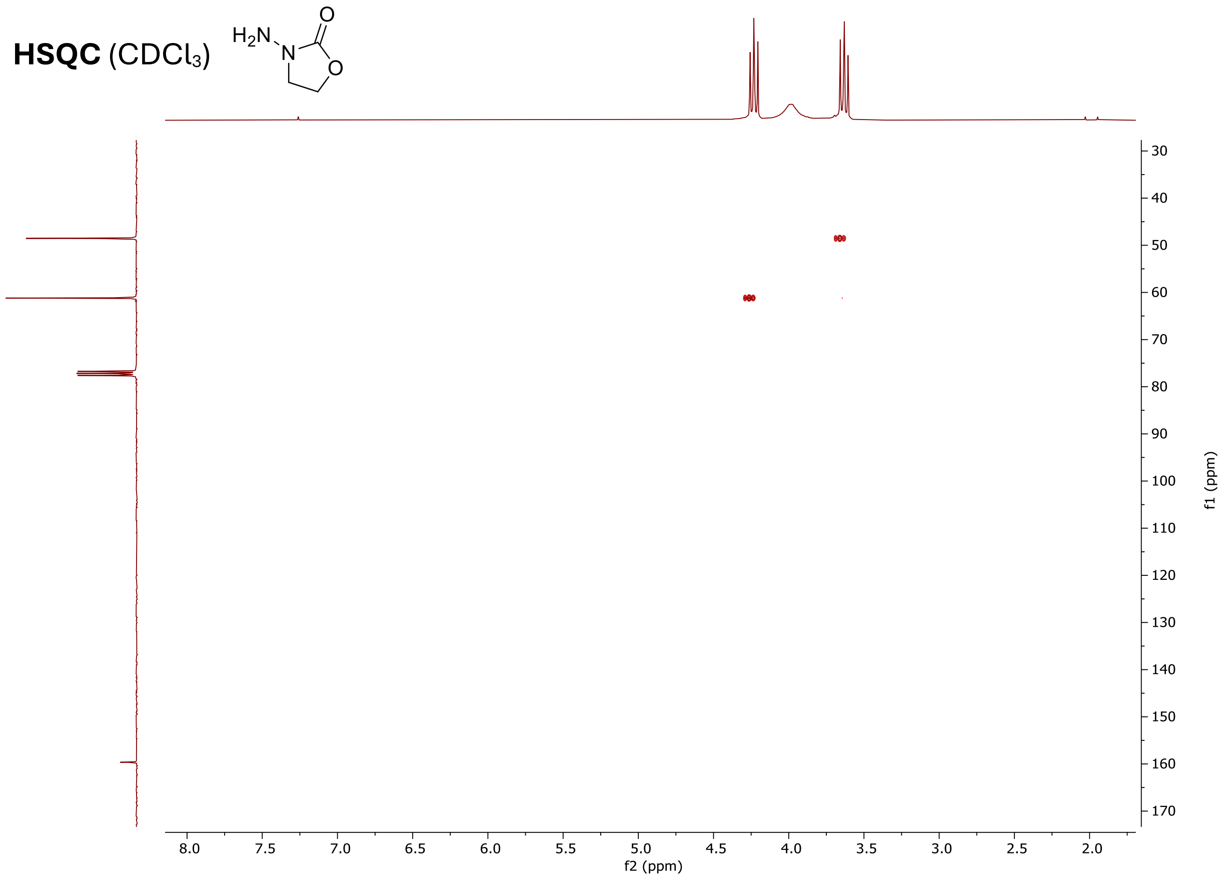

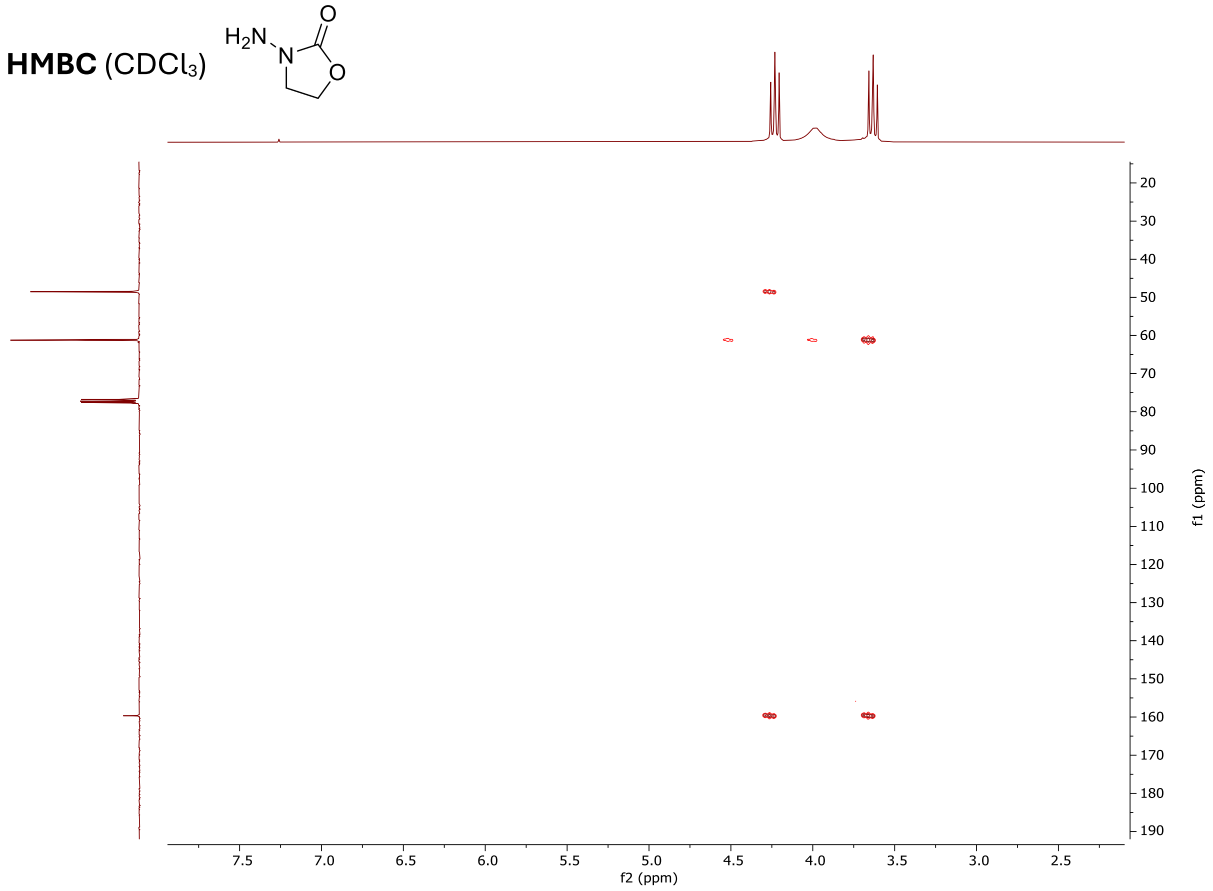

2,4,6-trimethyl-1-(2-oxooxazolidin-3-yl)pyridinium tetrafluoroborate (3a)

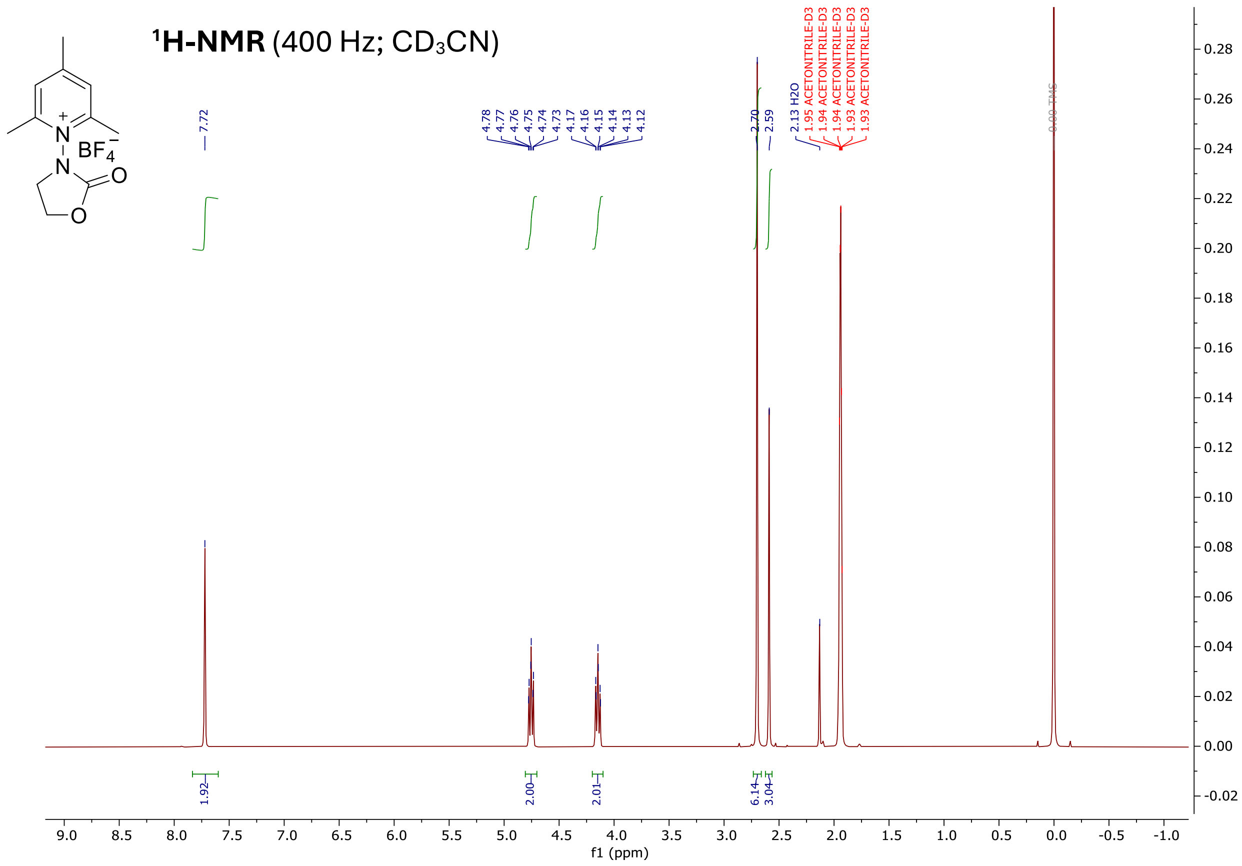

^{19}F-NMR (376 MHz; CD_3CN)

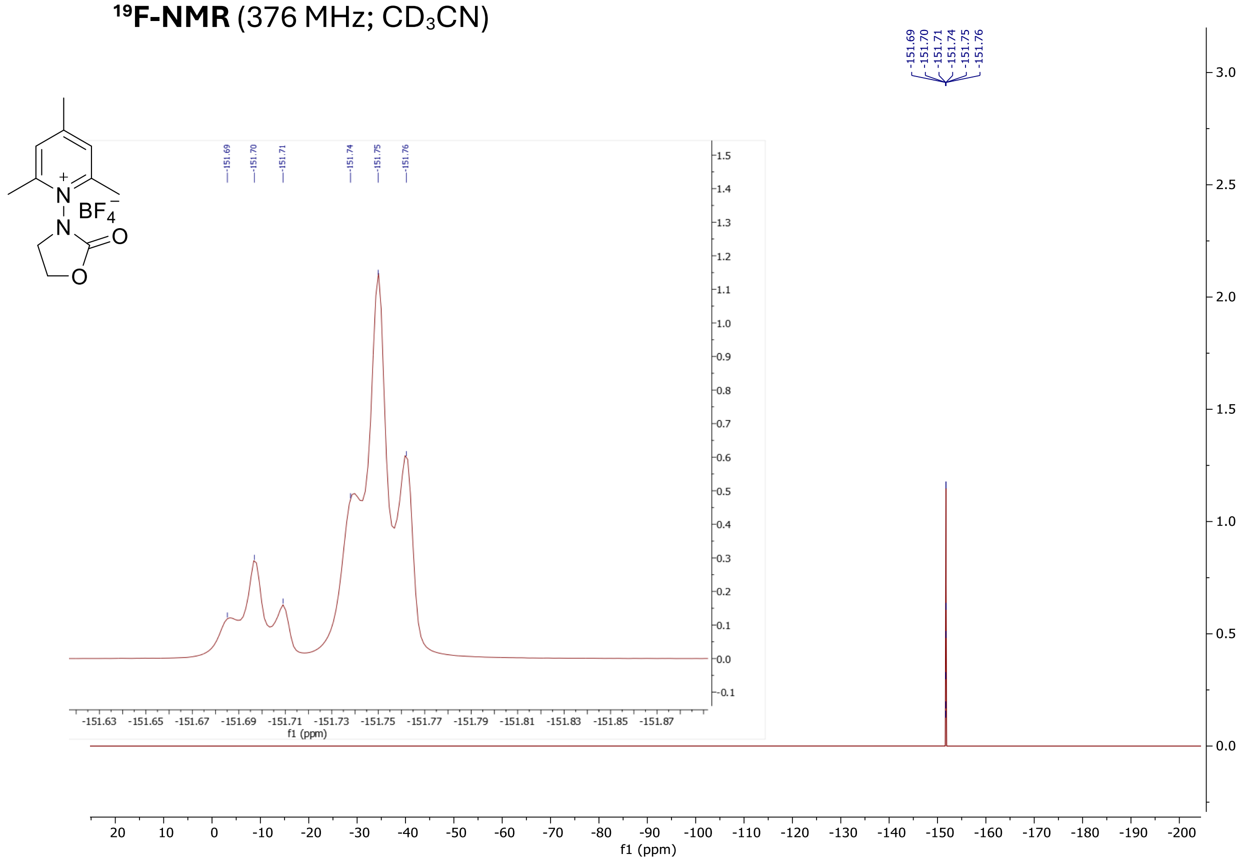

^{13}C-NMR (101 MHz; CD_3CN)

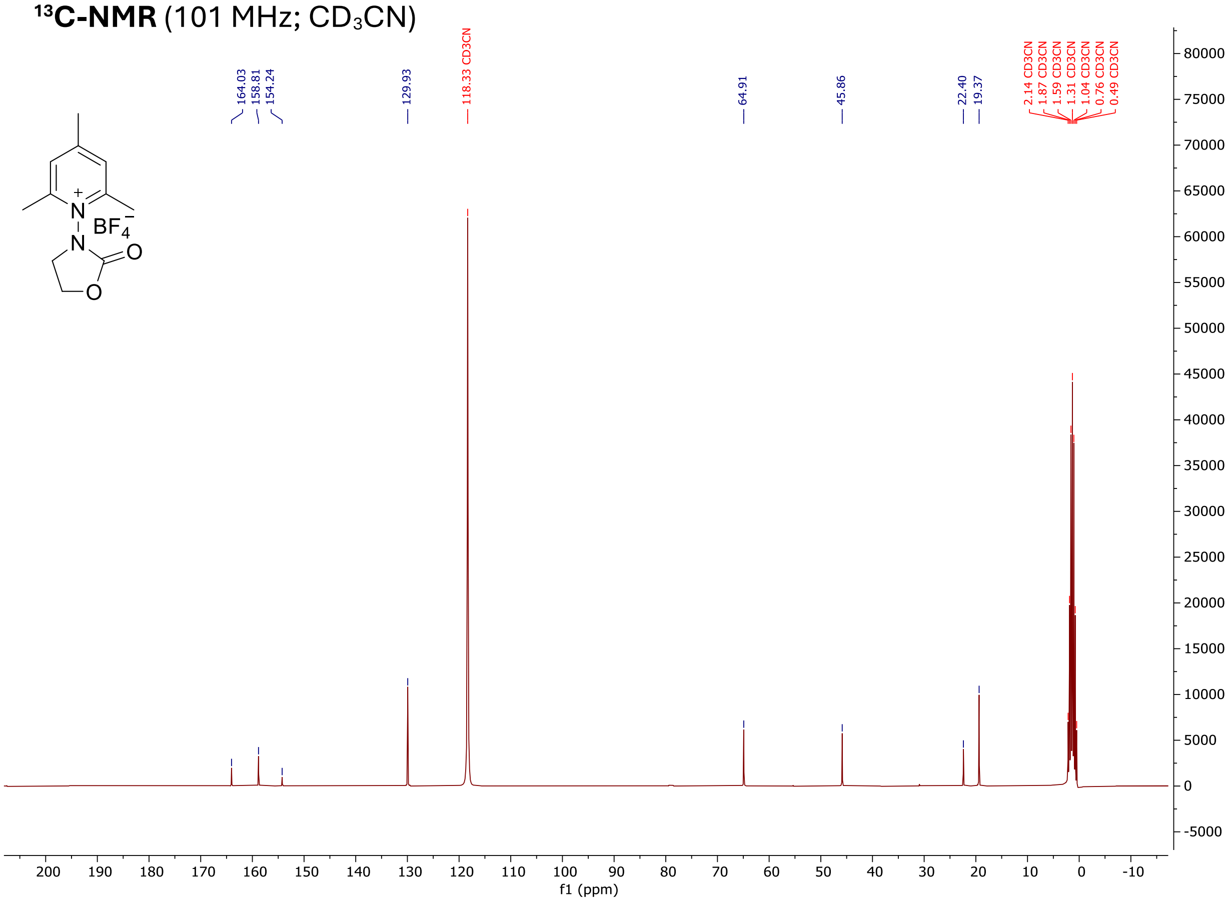

COSY ($CDCl_3$)

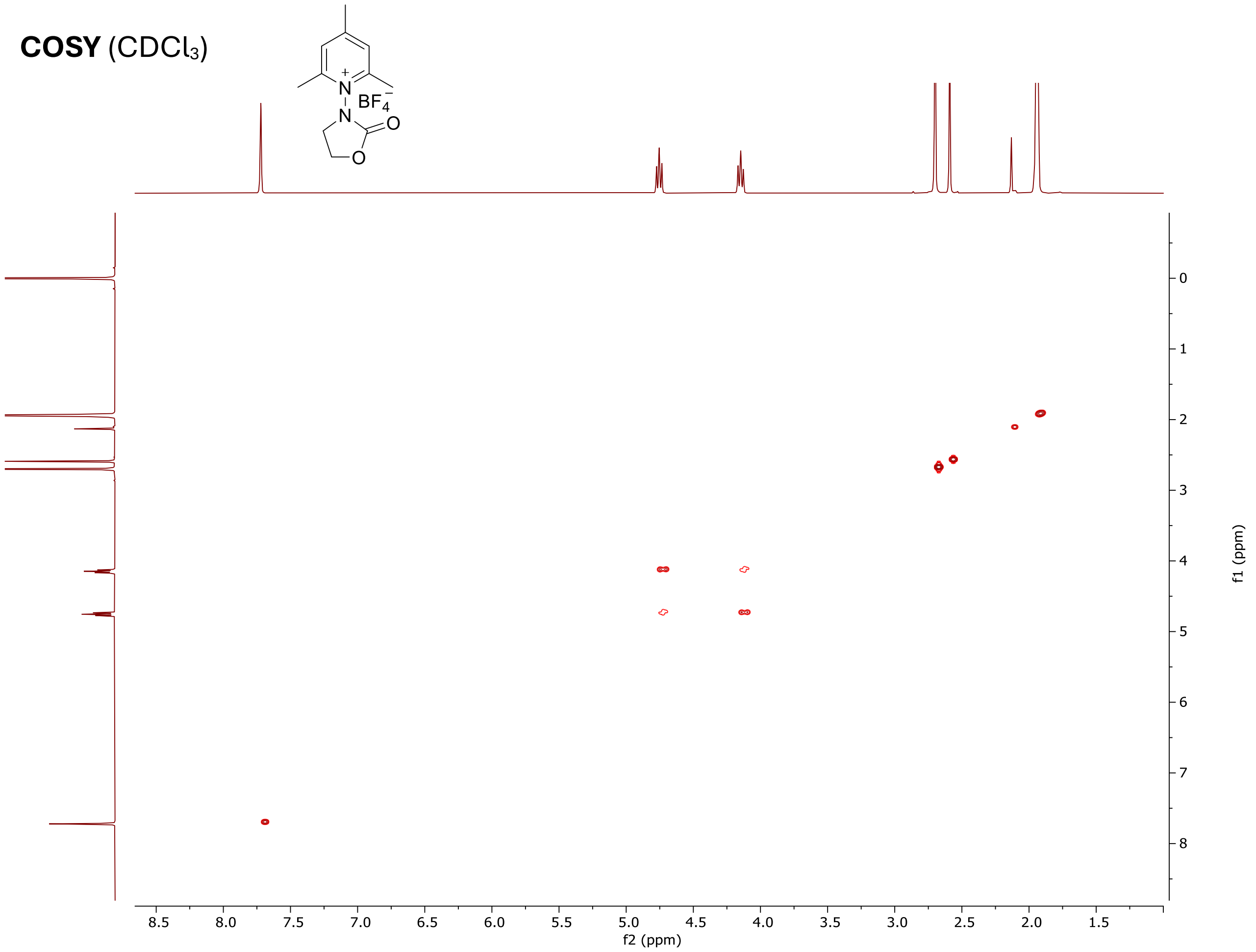

HSQC ($CDCl_3$)

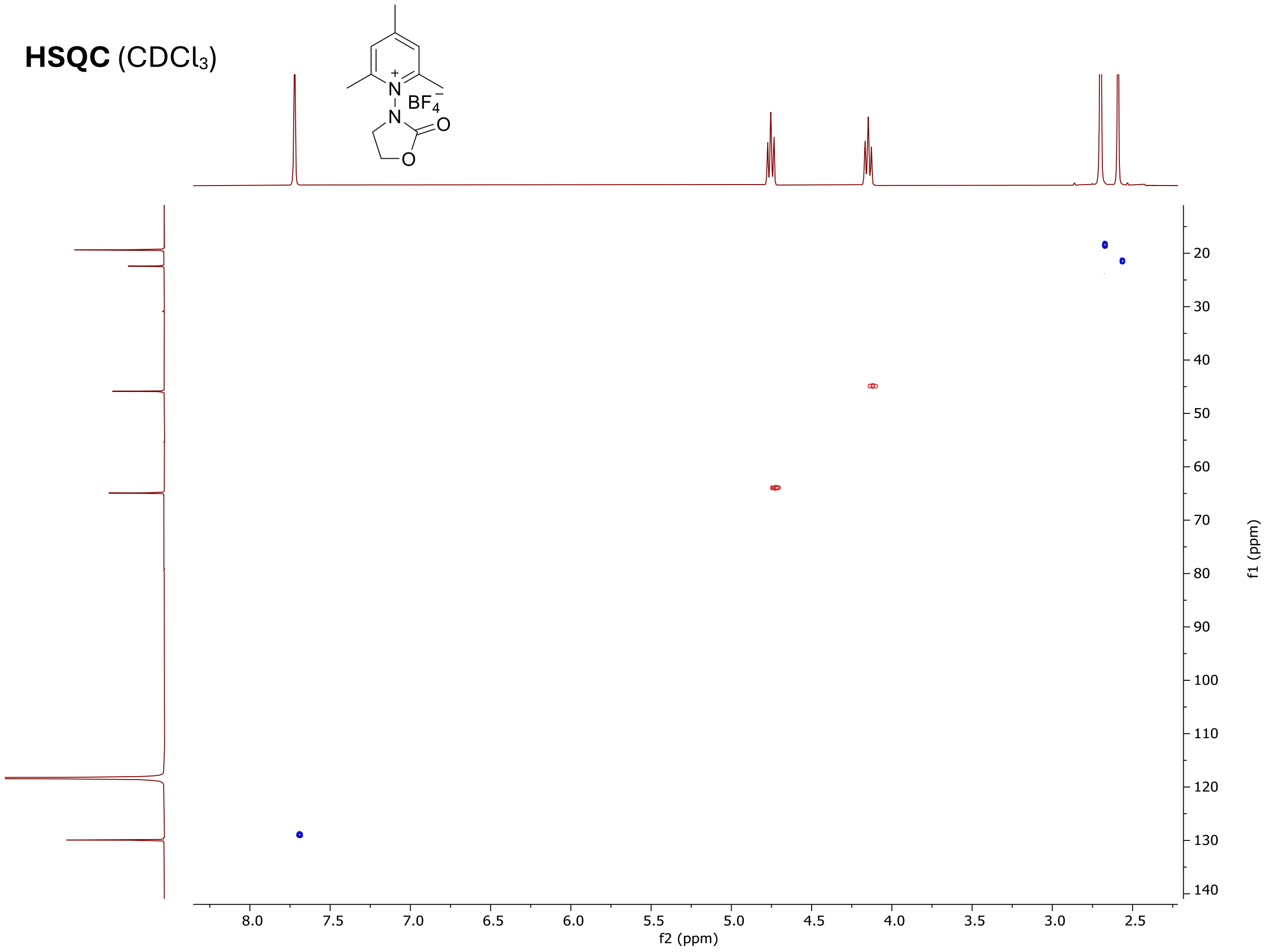

HMBC ($CDCl_3$)

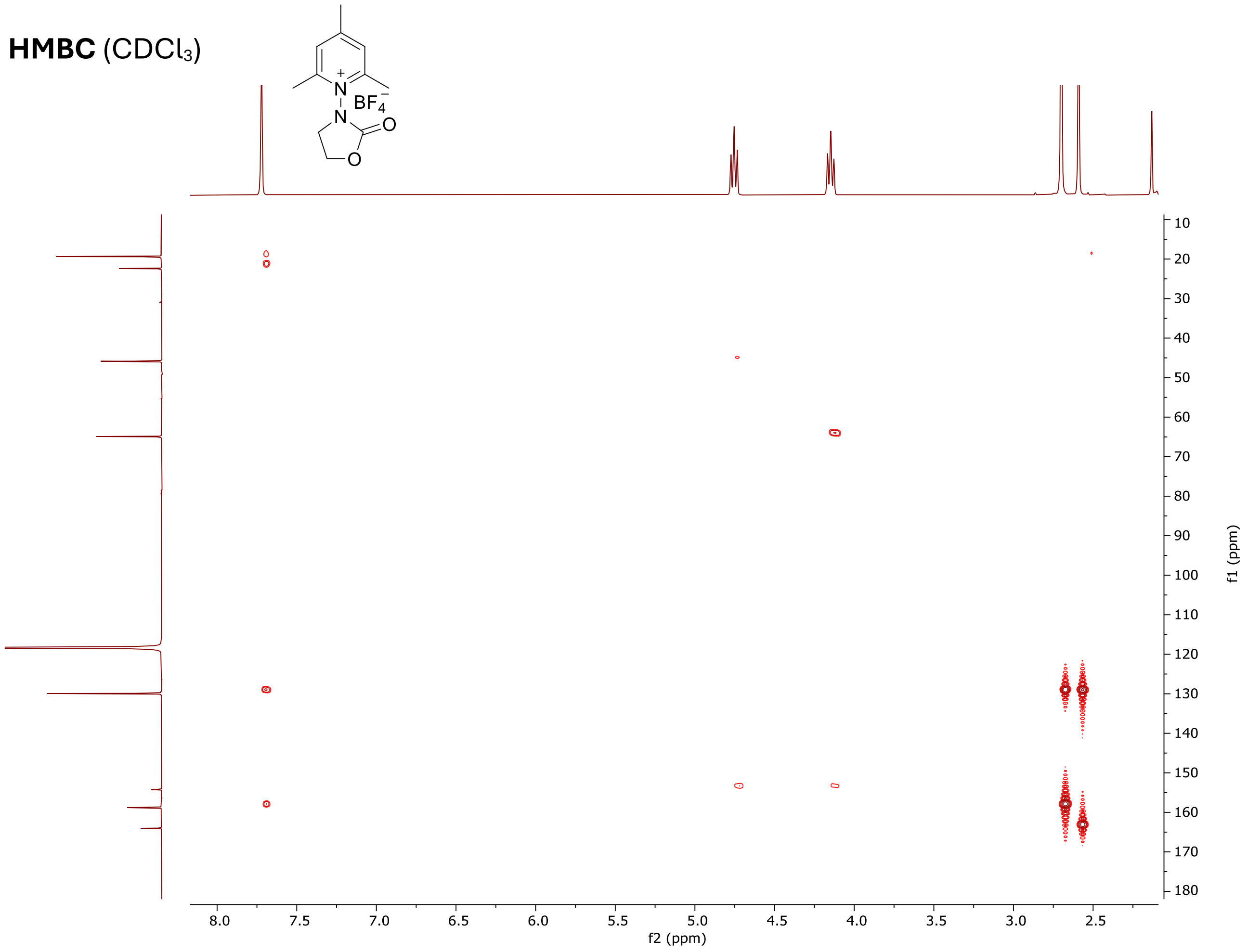

2,4,6-trimethyl-1-(2-oxooxazolidin-3-yl)pyridinium trifluoromethanesulfonate (3b)

¹H-NMR (400 MHz; CD_3CN)

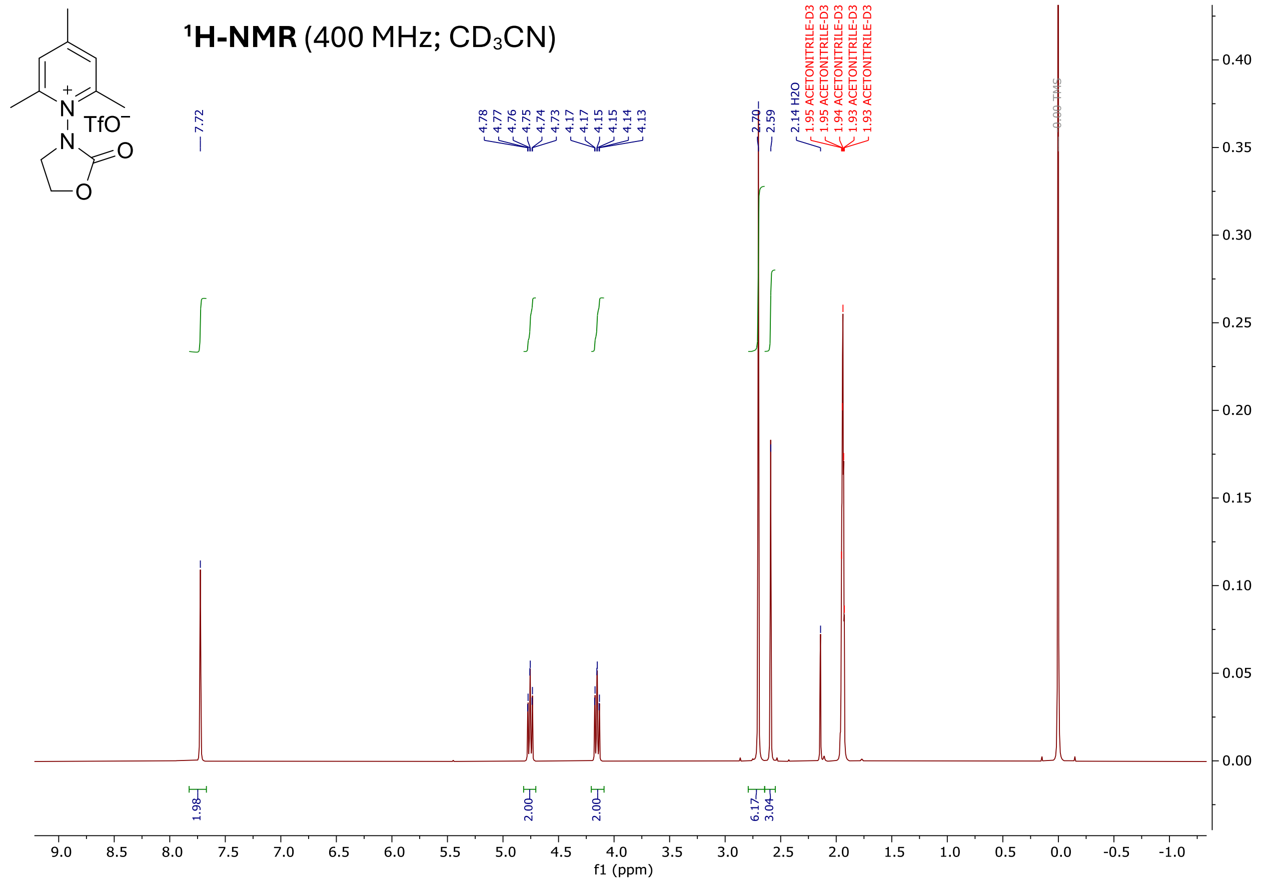

^{19}F-NMR (376 MHz; CD_3CN)

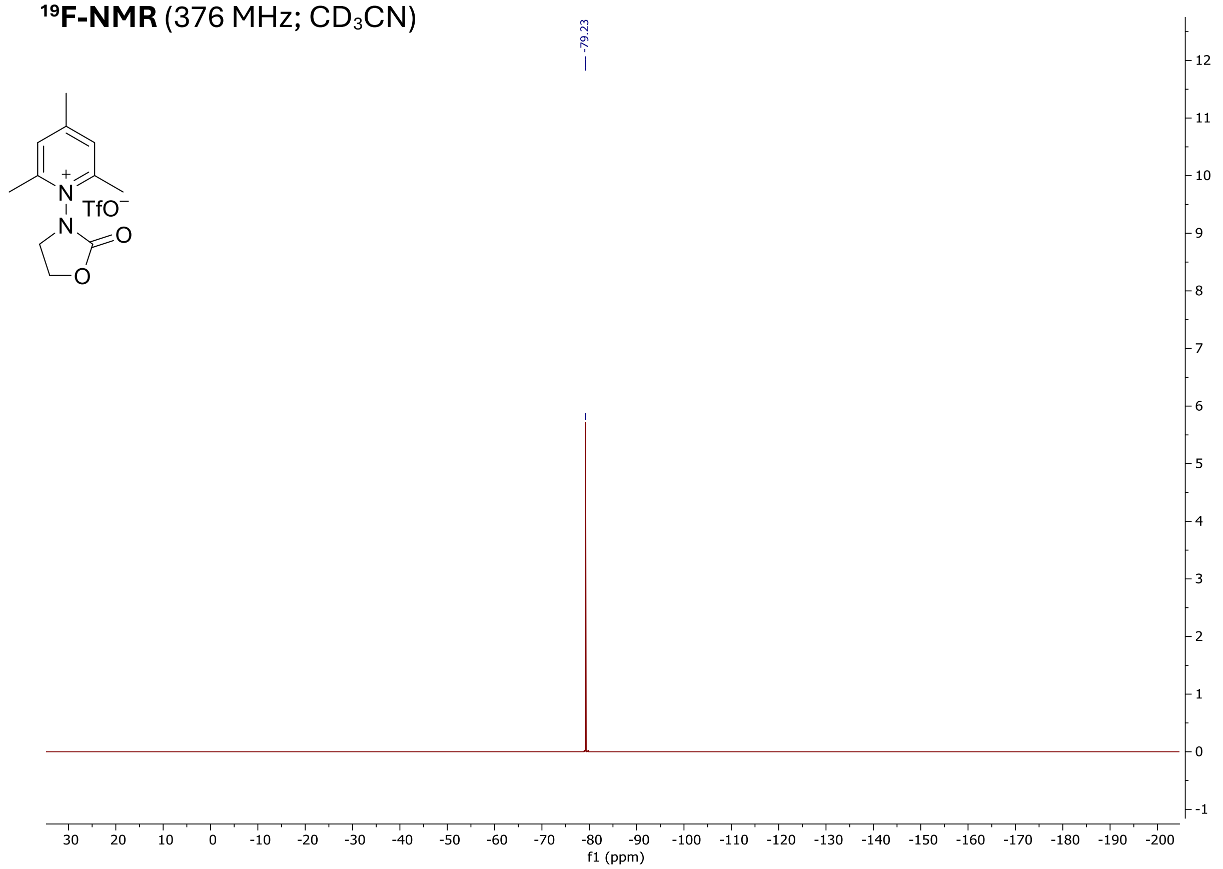

^{13}C-NMR (75 MHz; CD_3CN)

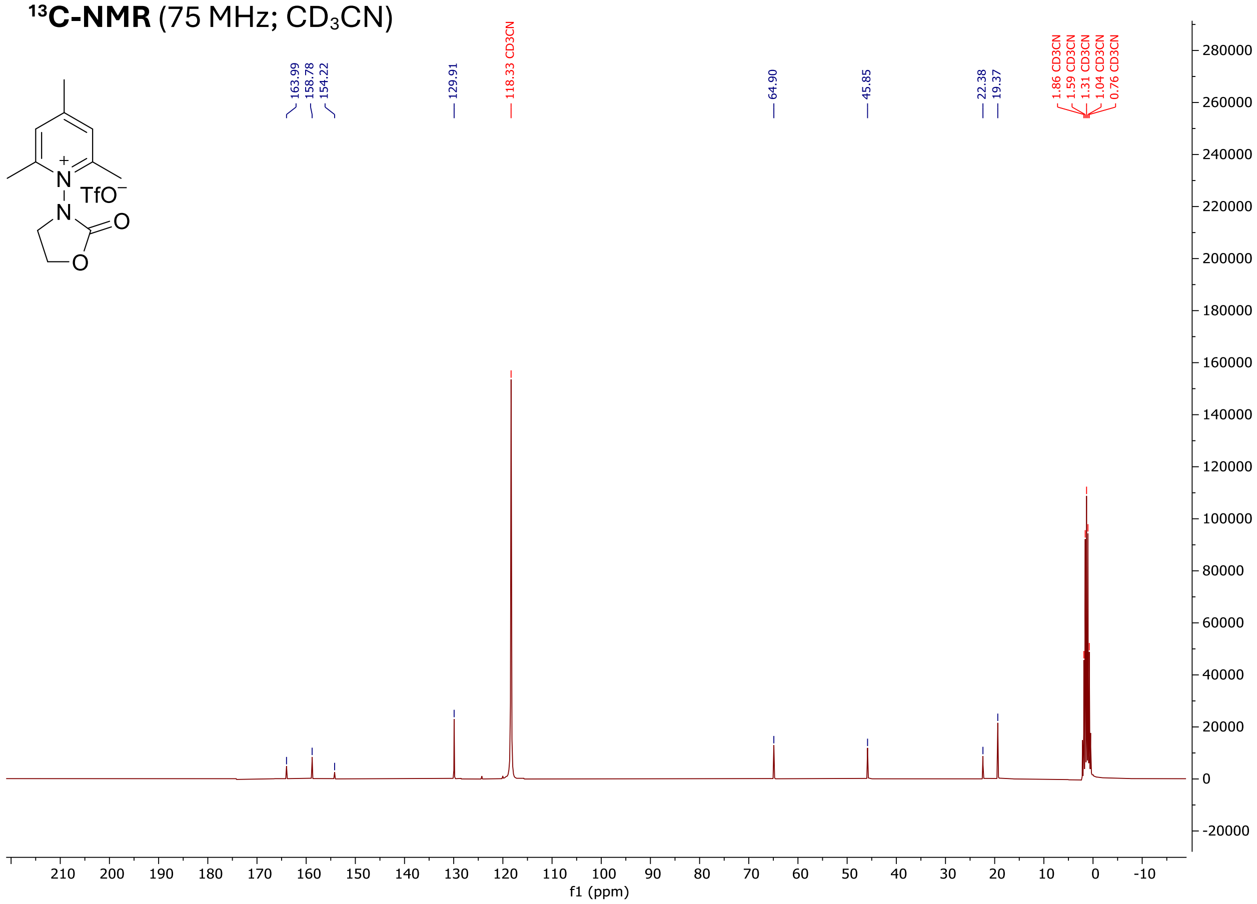

COSY ($CDCl_3$)

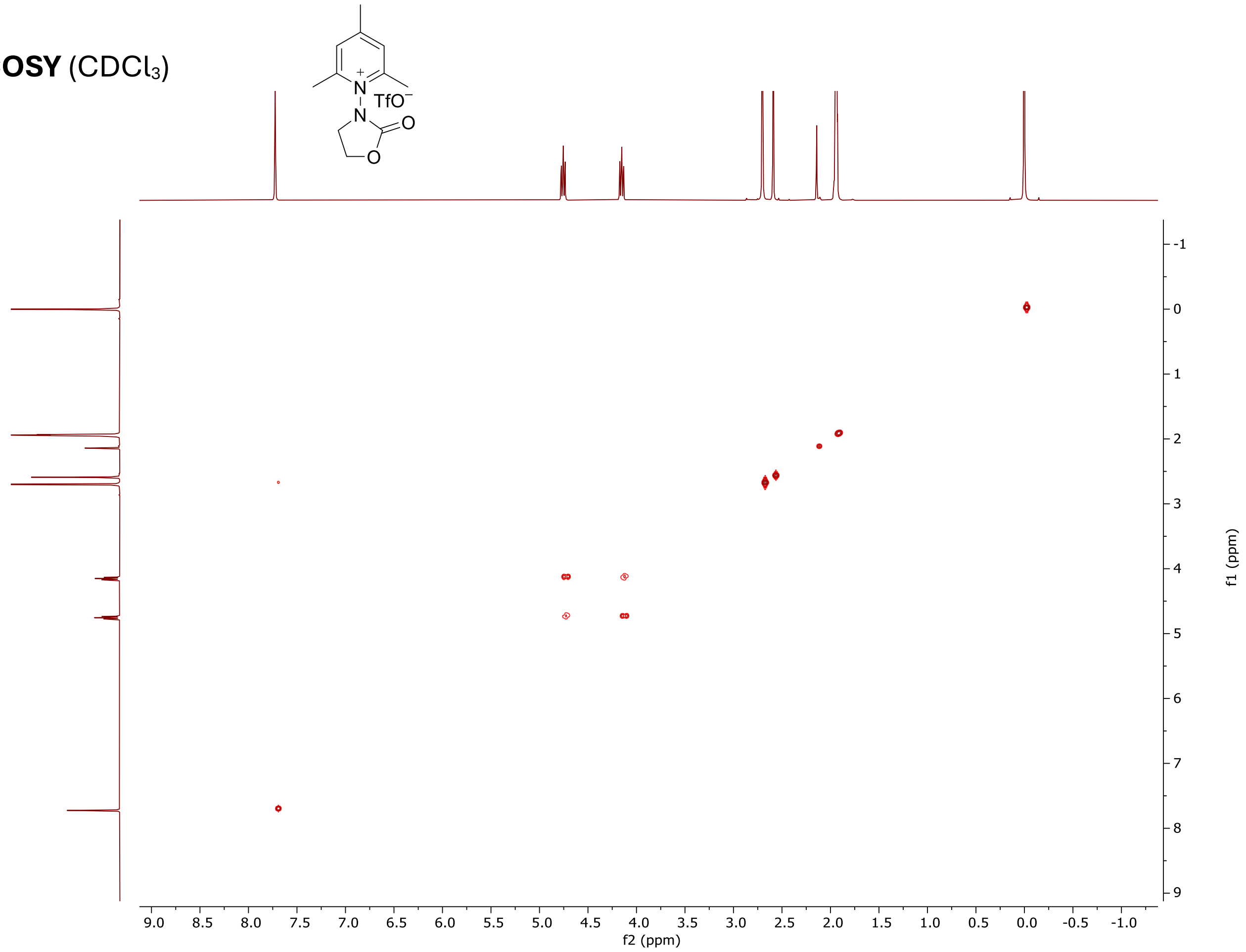

HSQC ($CDCl_3$)

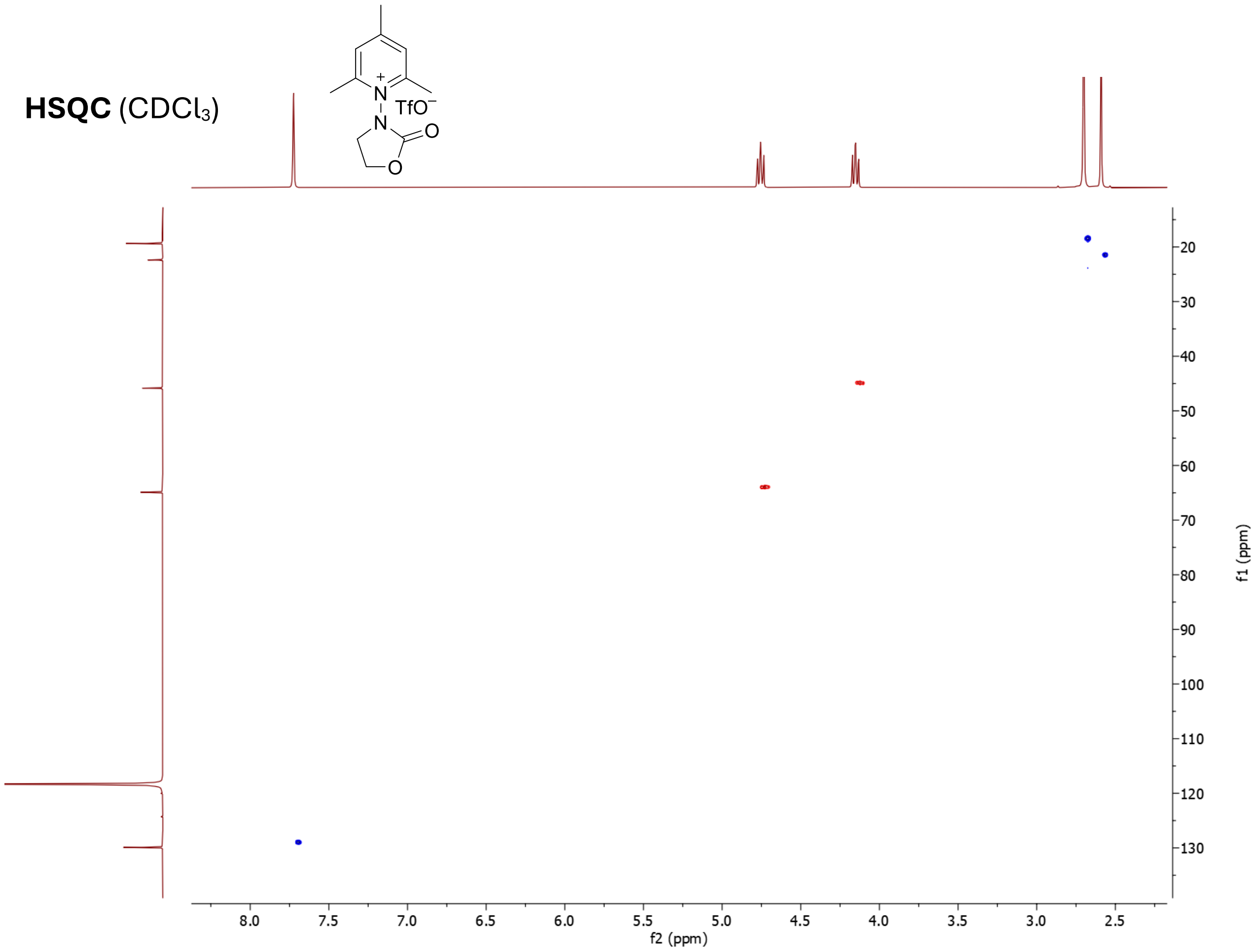

HMBC ($CDCl_3$)

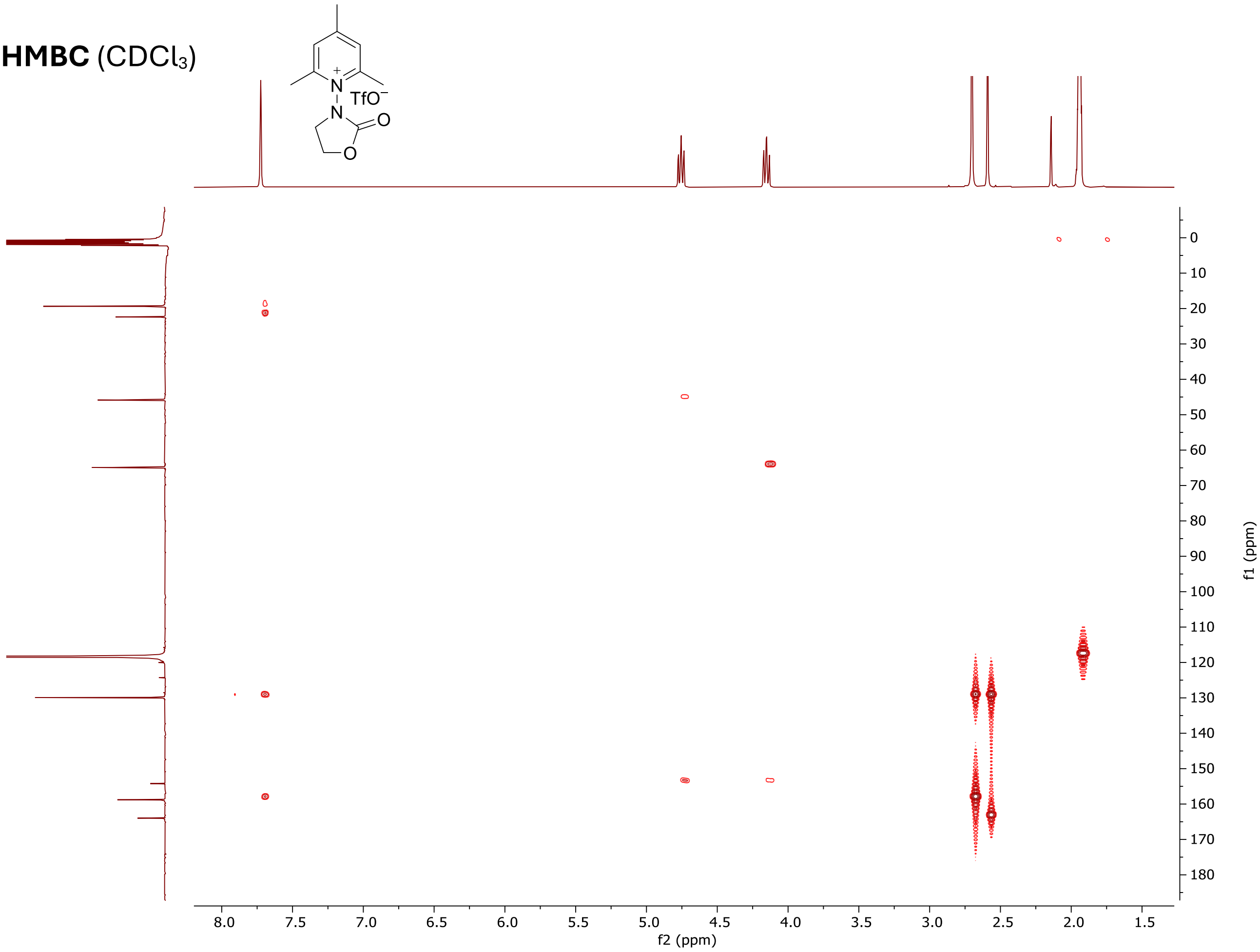

3-(1-methyl-1H-indol-2-yl)oxazolidin-2-one (5a)

^{1}H-NMR (300 MHz; $CDCl_3$)

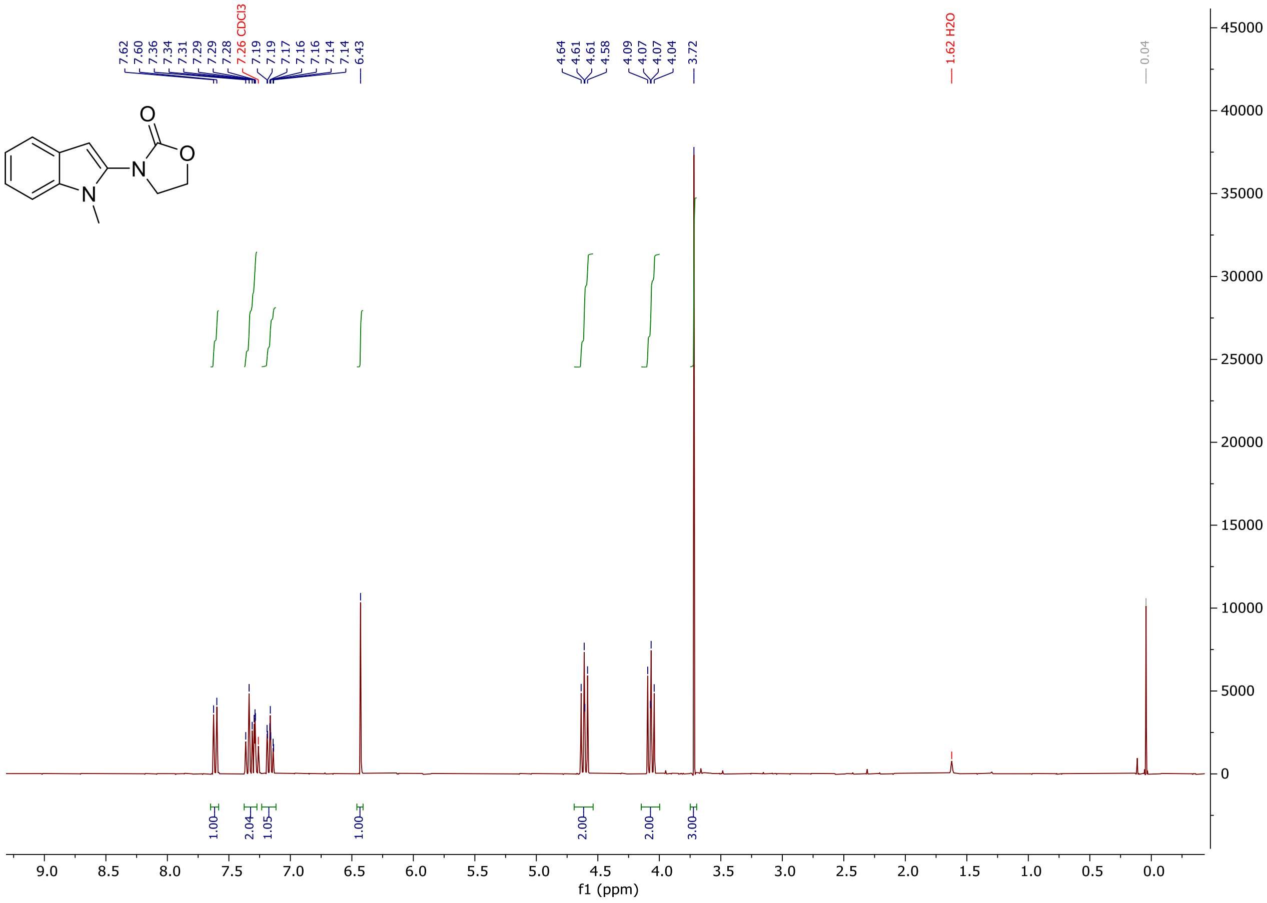

^{13}**C-NMR** (101 MHz; $CDCl_3$)

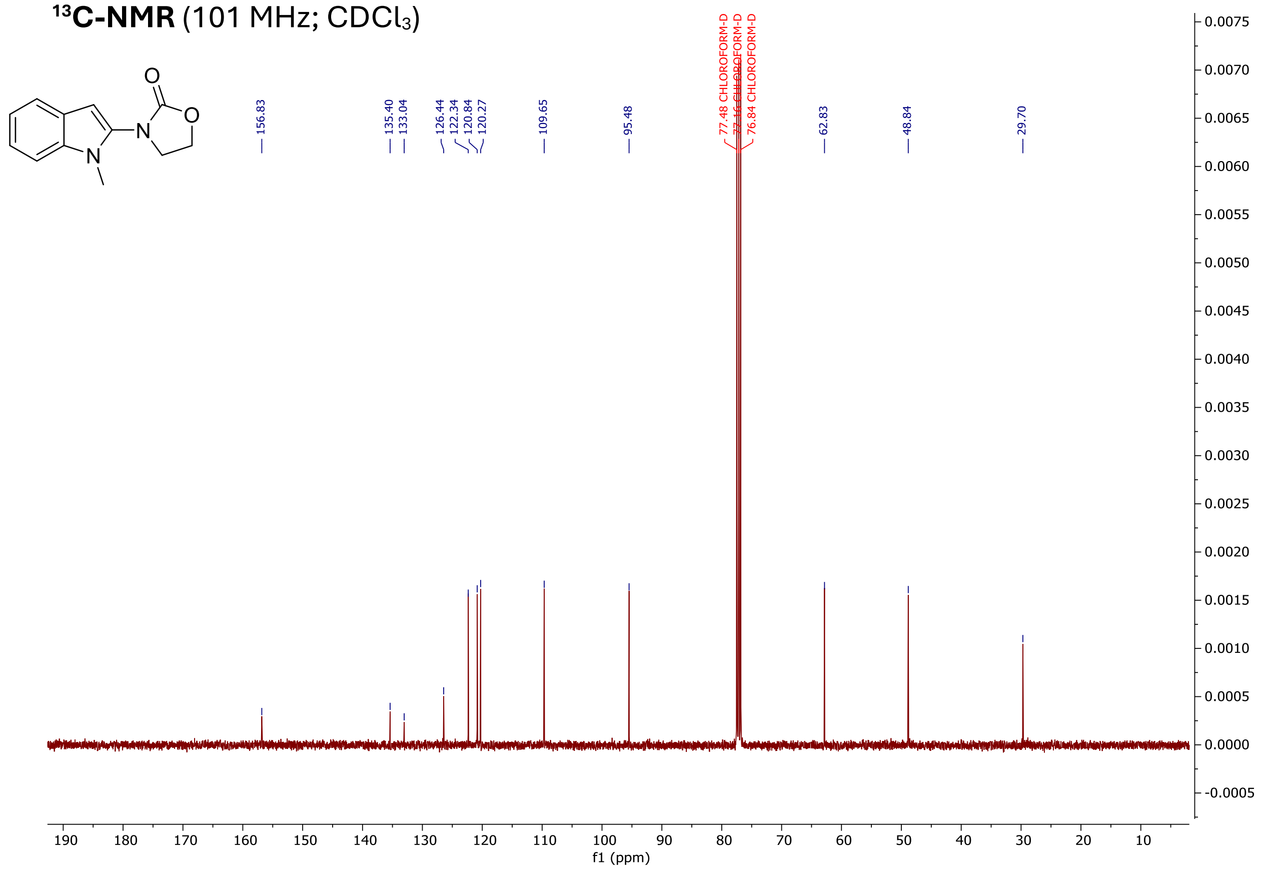

COSY ($CDCl_3$)

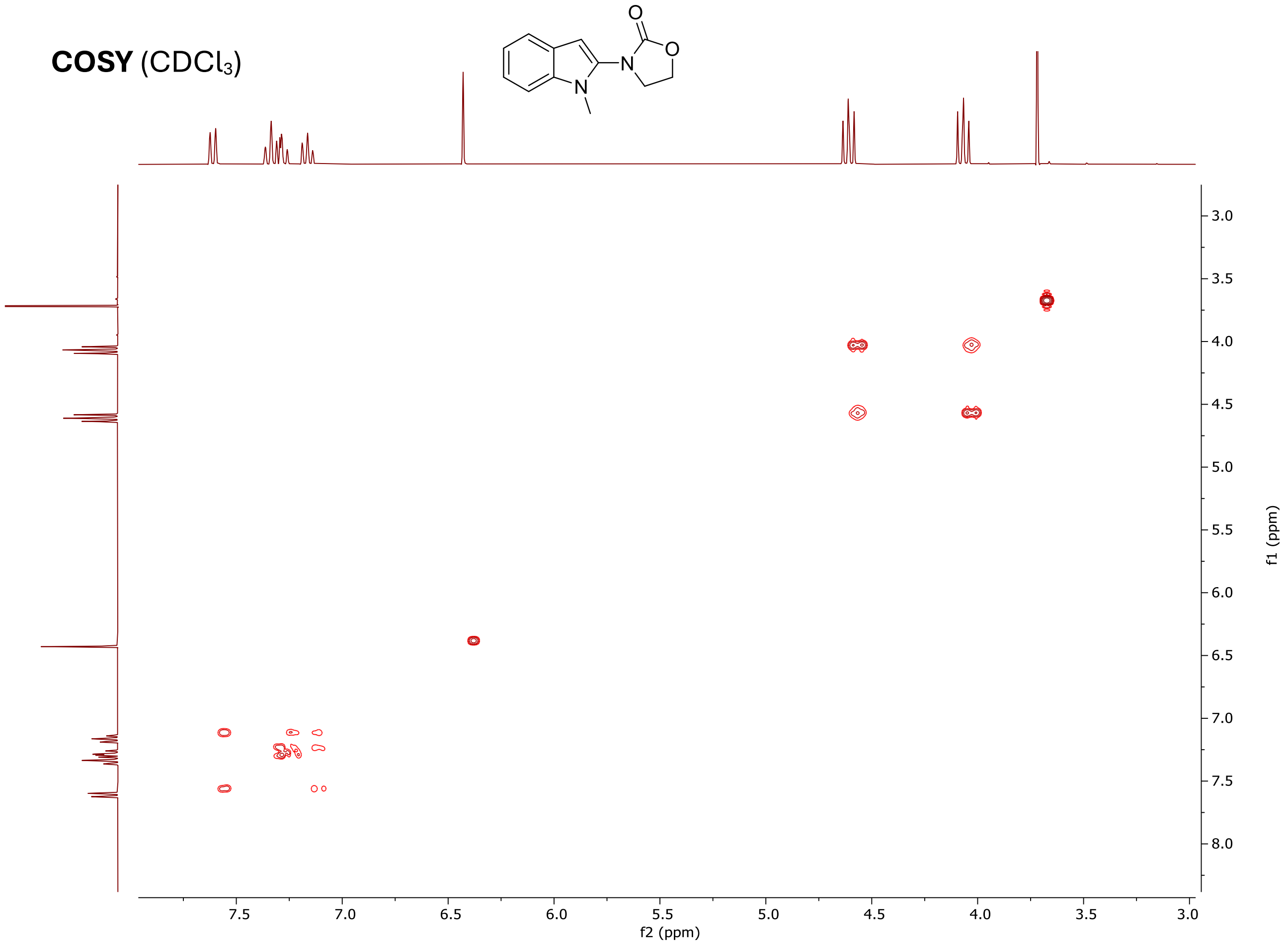

HSQC (CDCl$_3$)

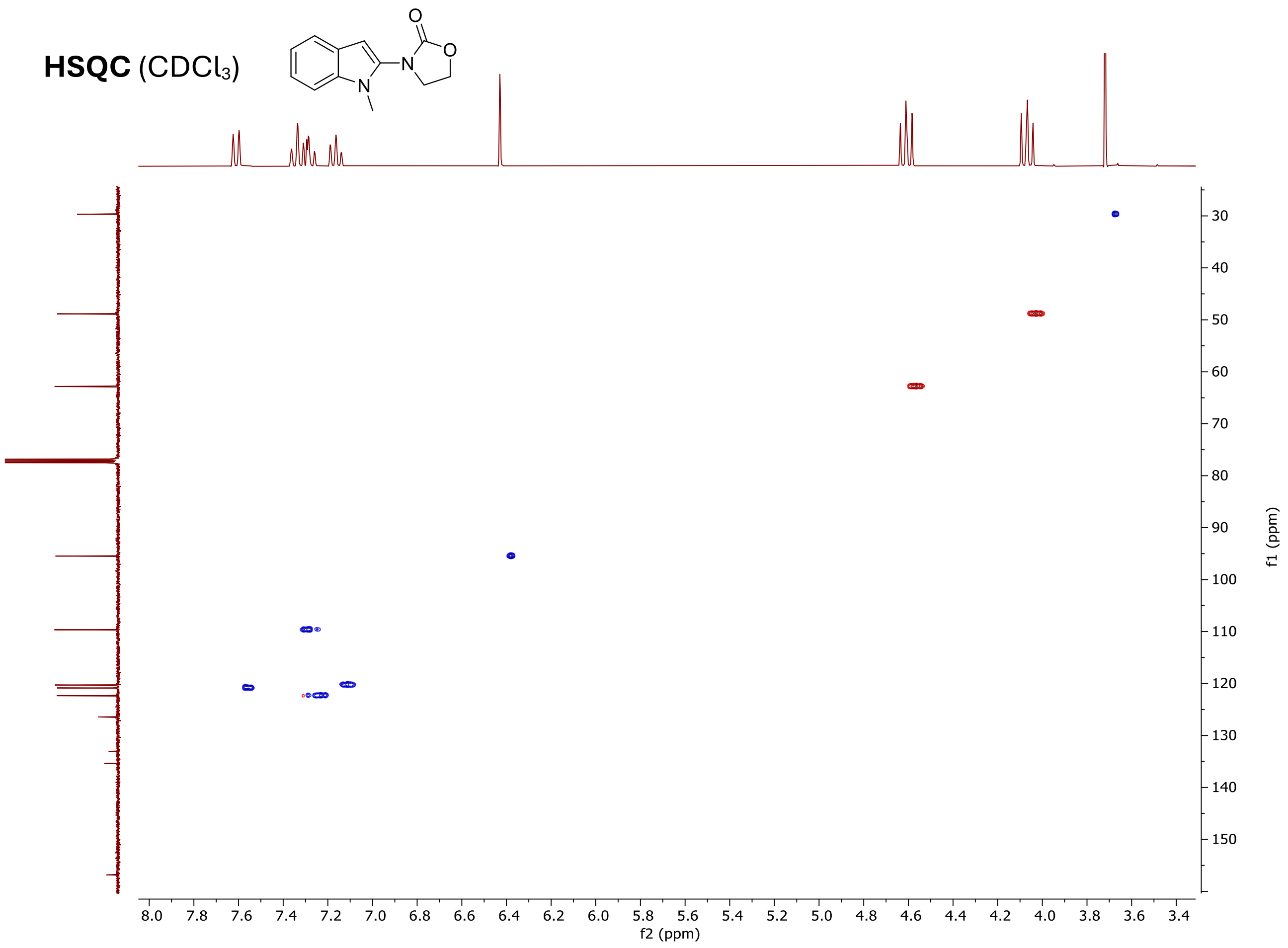

HMBC (CDCl$_3$)

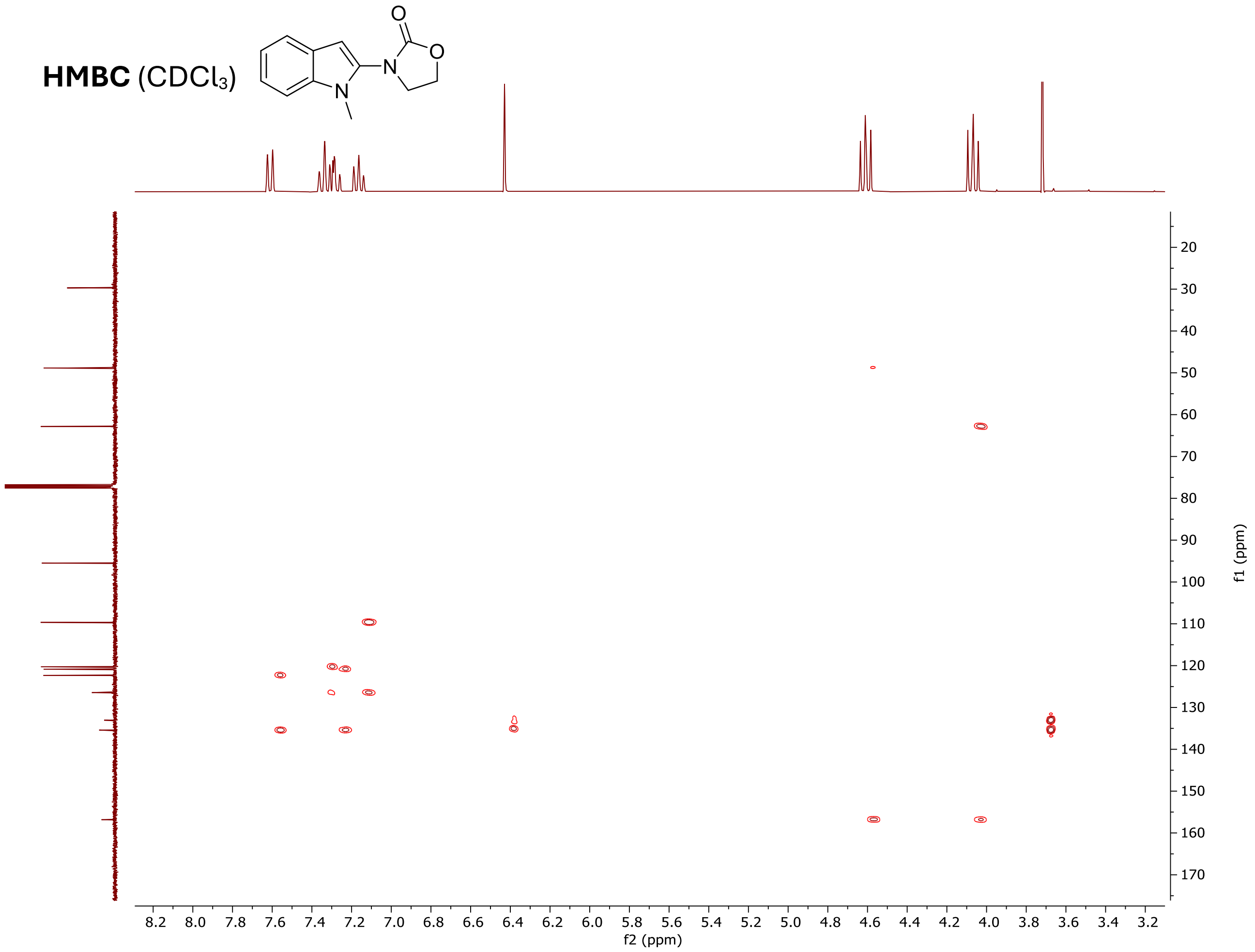

3-(5-chloro-1-methyl-1H-indol-2-yl)oxazolidin-2-one (5b) **^{1}H-NMR** (300 MHz; $CDCl_3$)

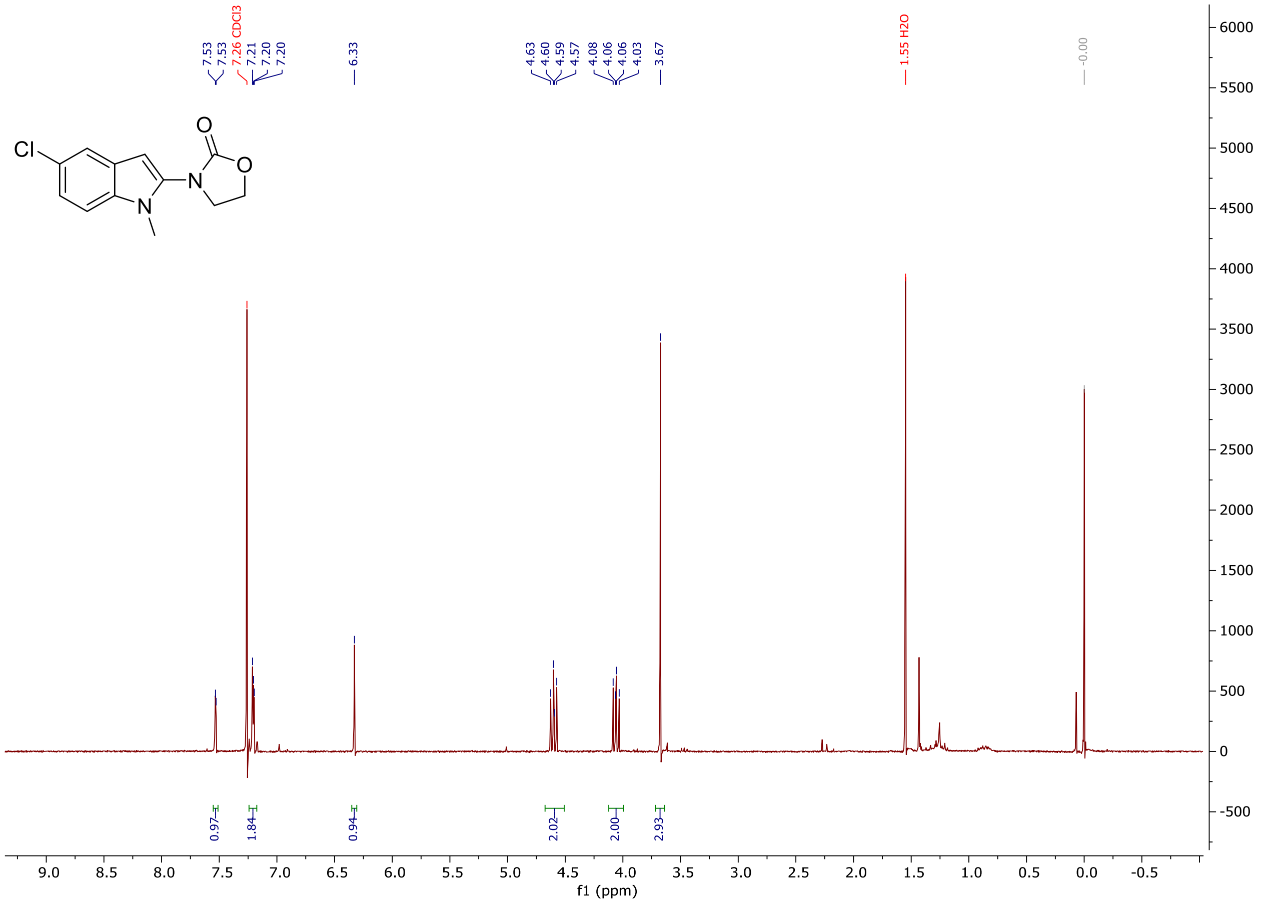

^{13}C-NMR (75 MHz; $CDCl_3$)

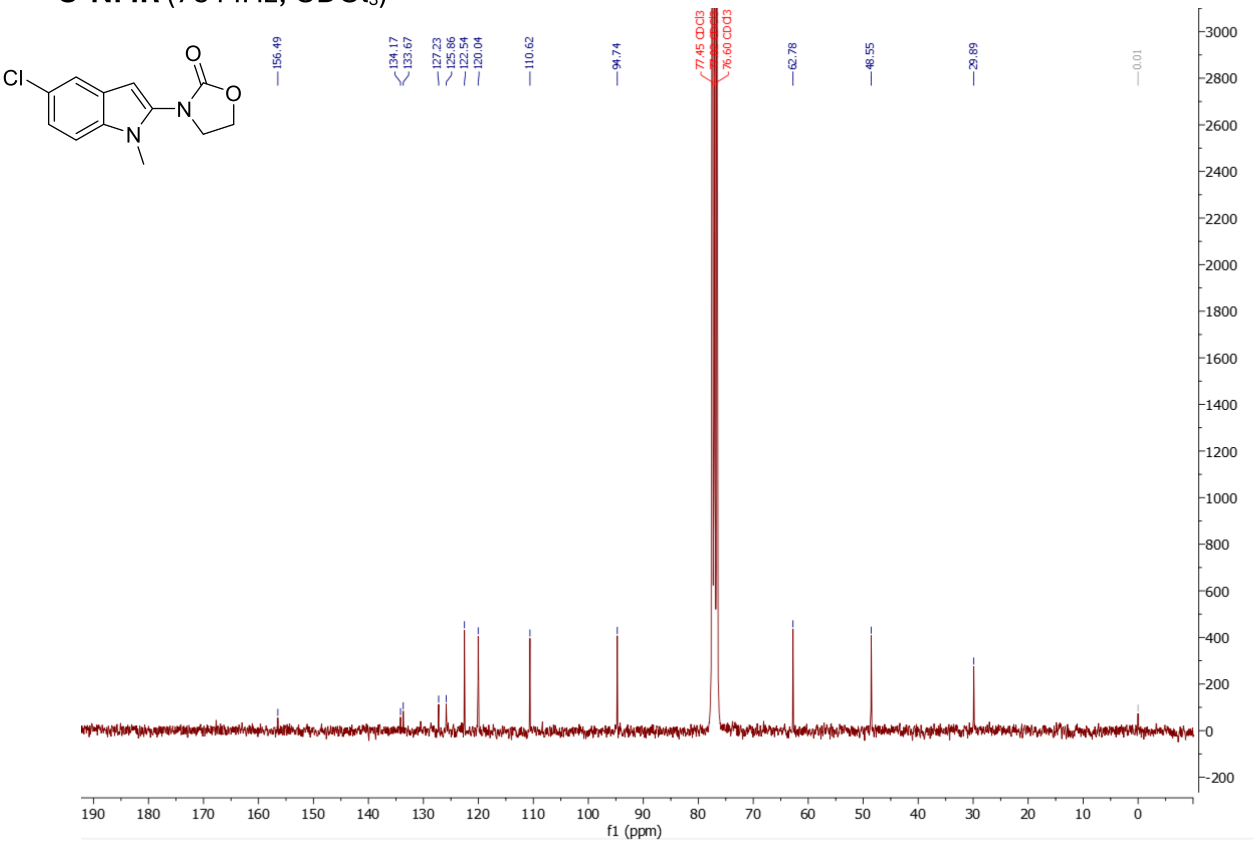

COSY ($CDCl_3$)

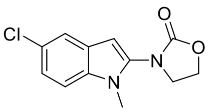

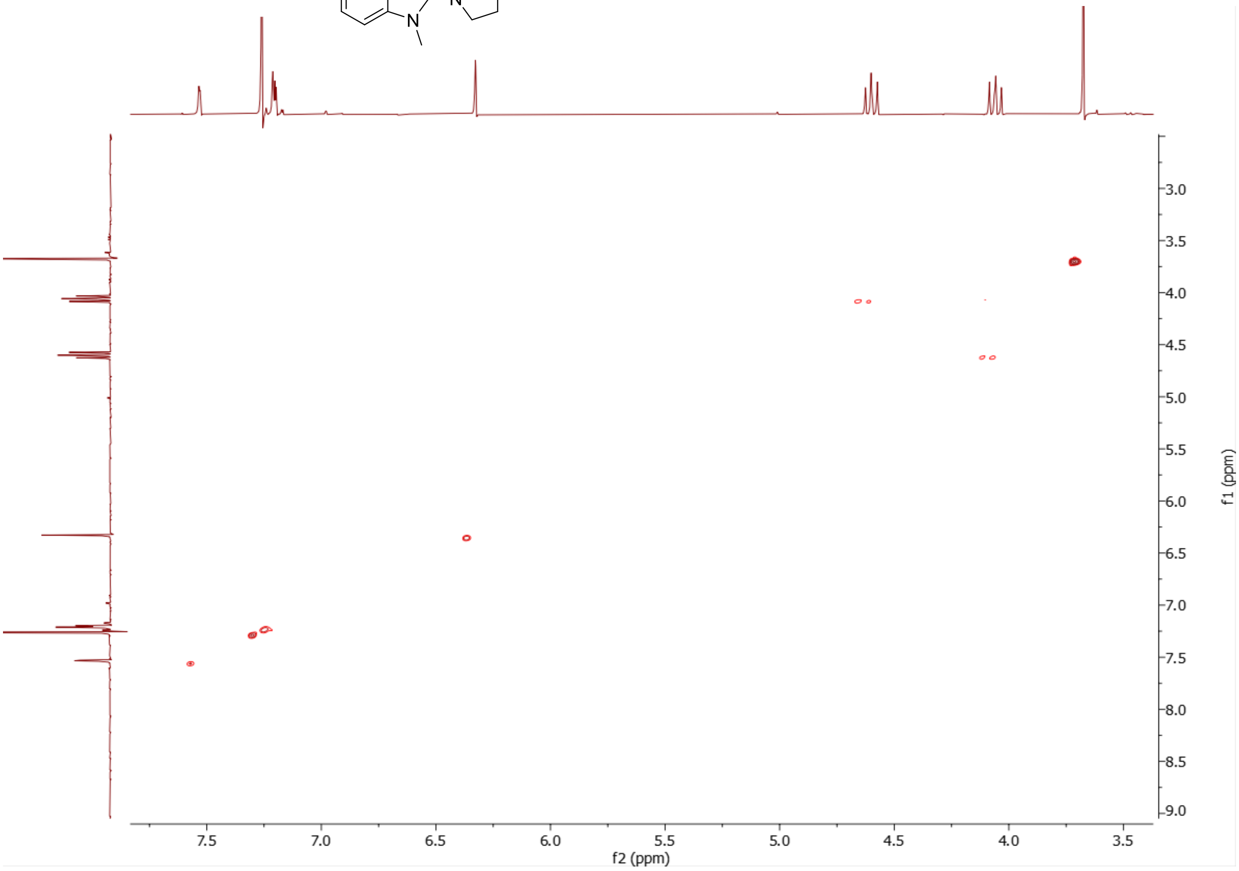

HSQC ($CDCl_3$)

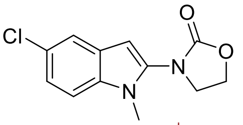

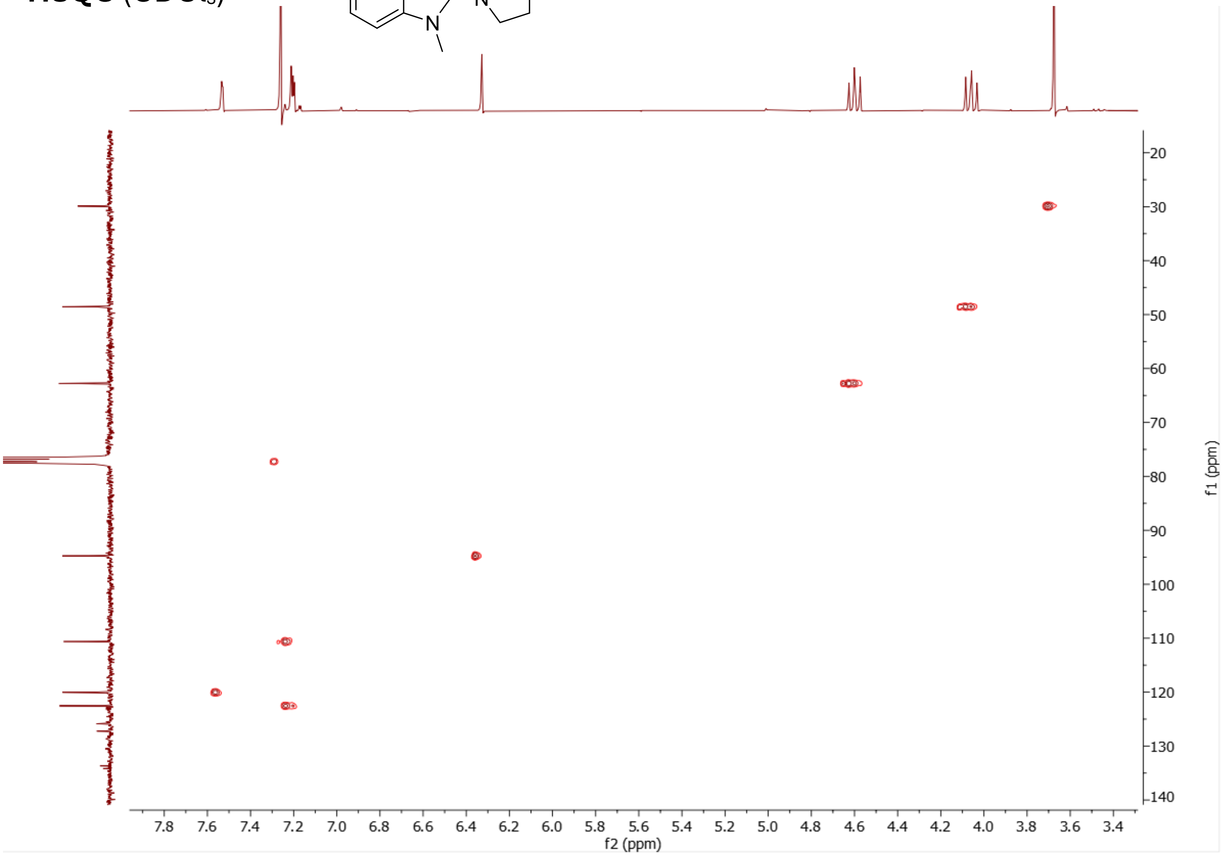

HMBC (CDCl$_3$)

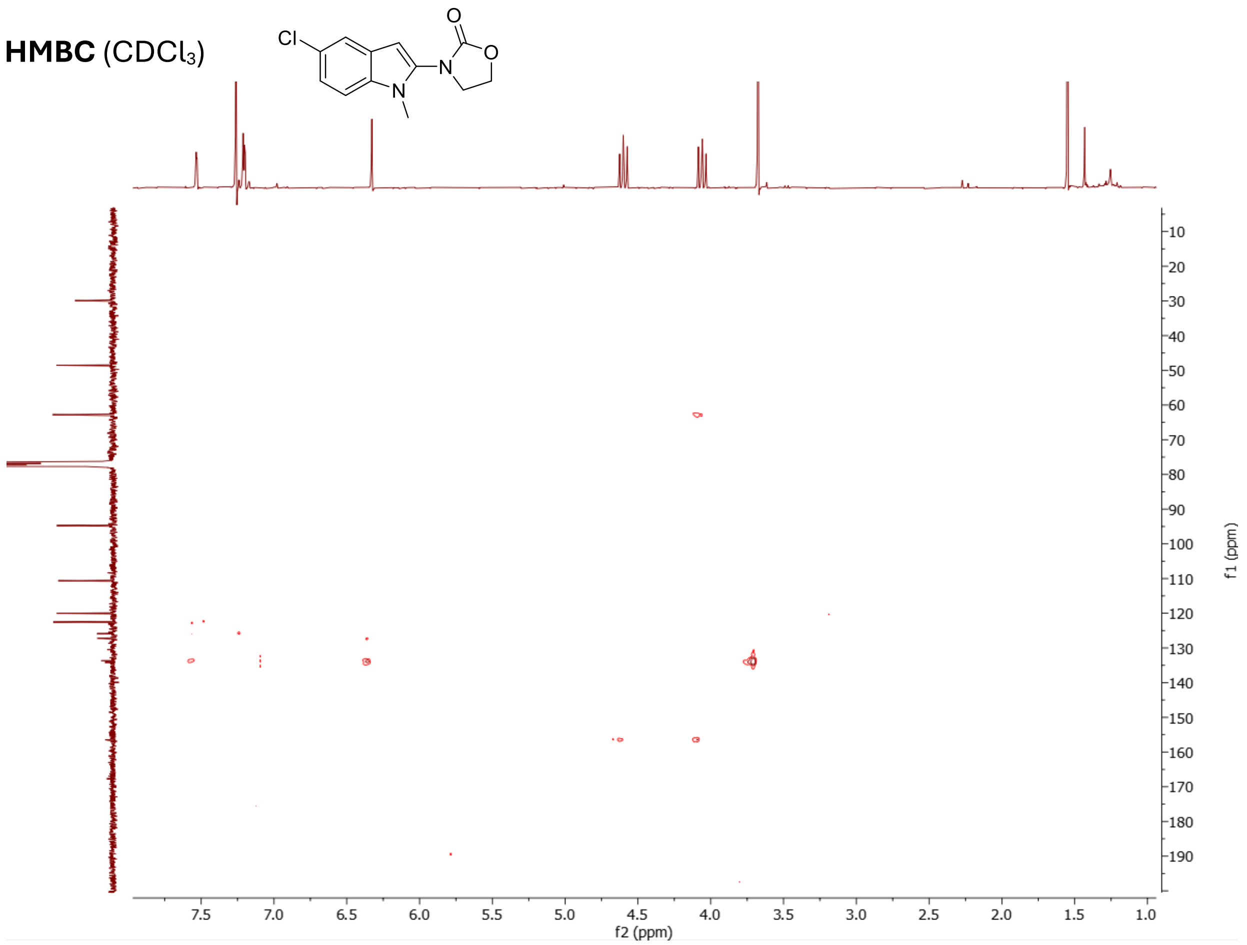

3-(5-bromo-1-methyl-1H-indol-2-yl)oxazolidin-2-one (5c)

¹H-NMR (400 MHz; CDCl$_3$)

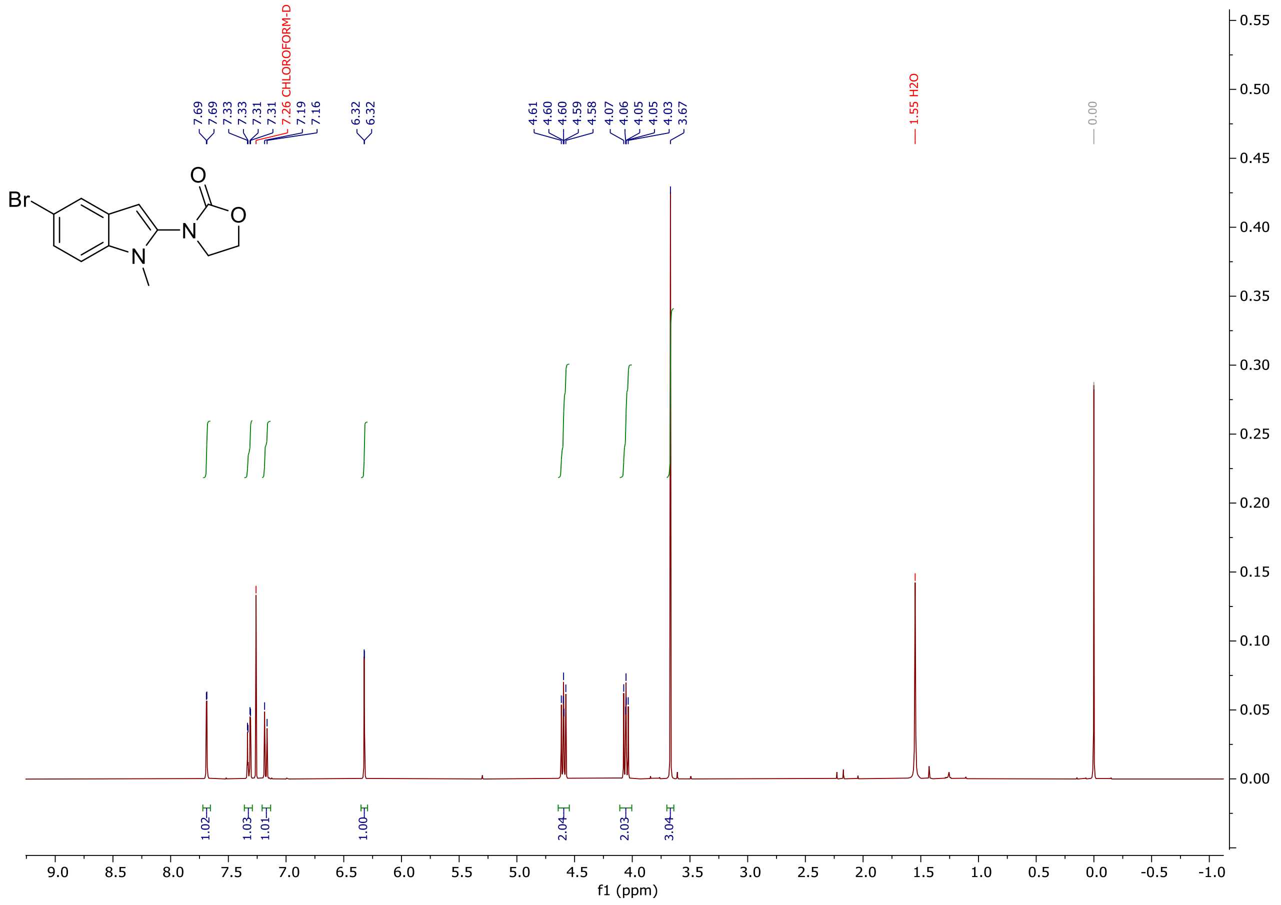

^{13}C-NMR (101 MHz; $CDCl_3$)

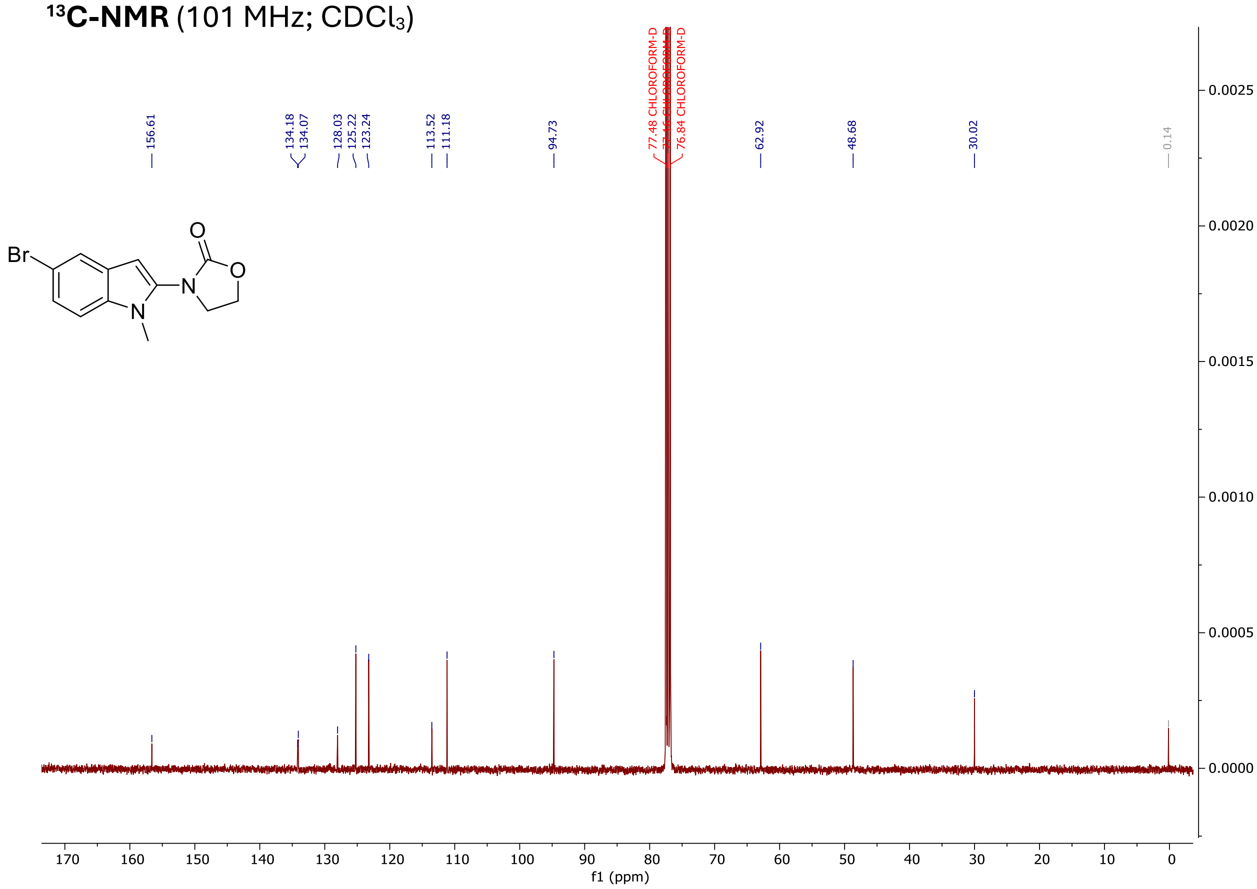

COSY ($CDCl_3$)

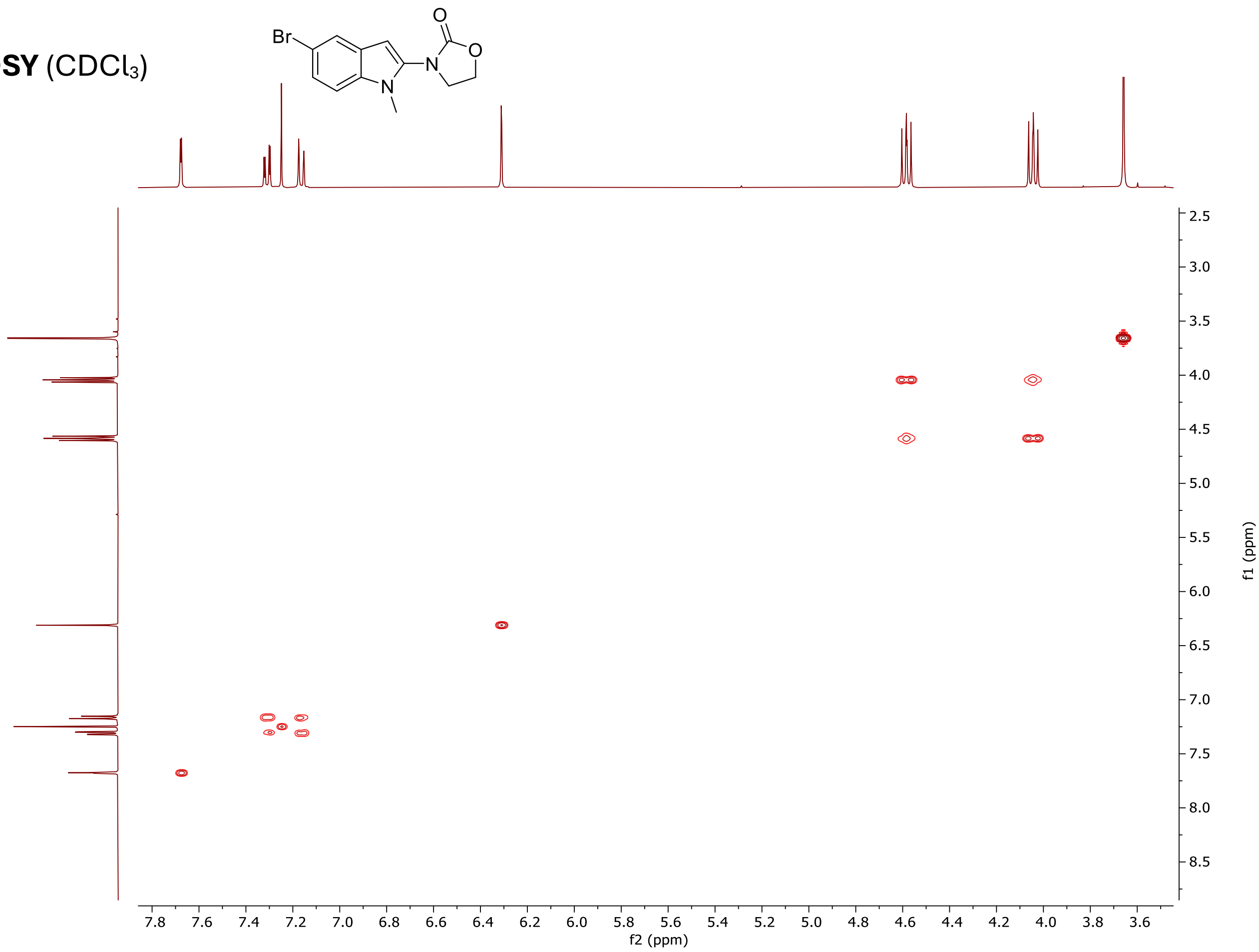

HSQC ($CDCl_3$)

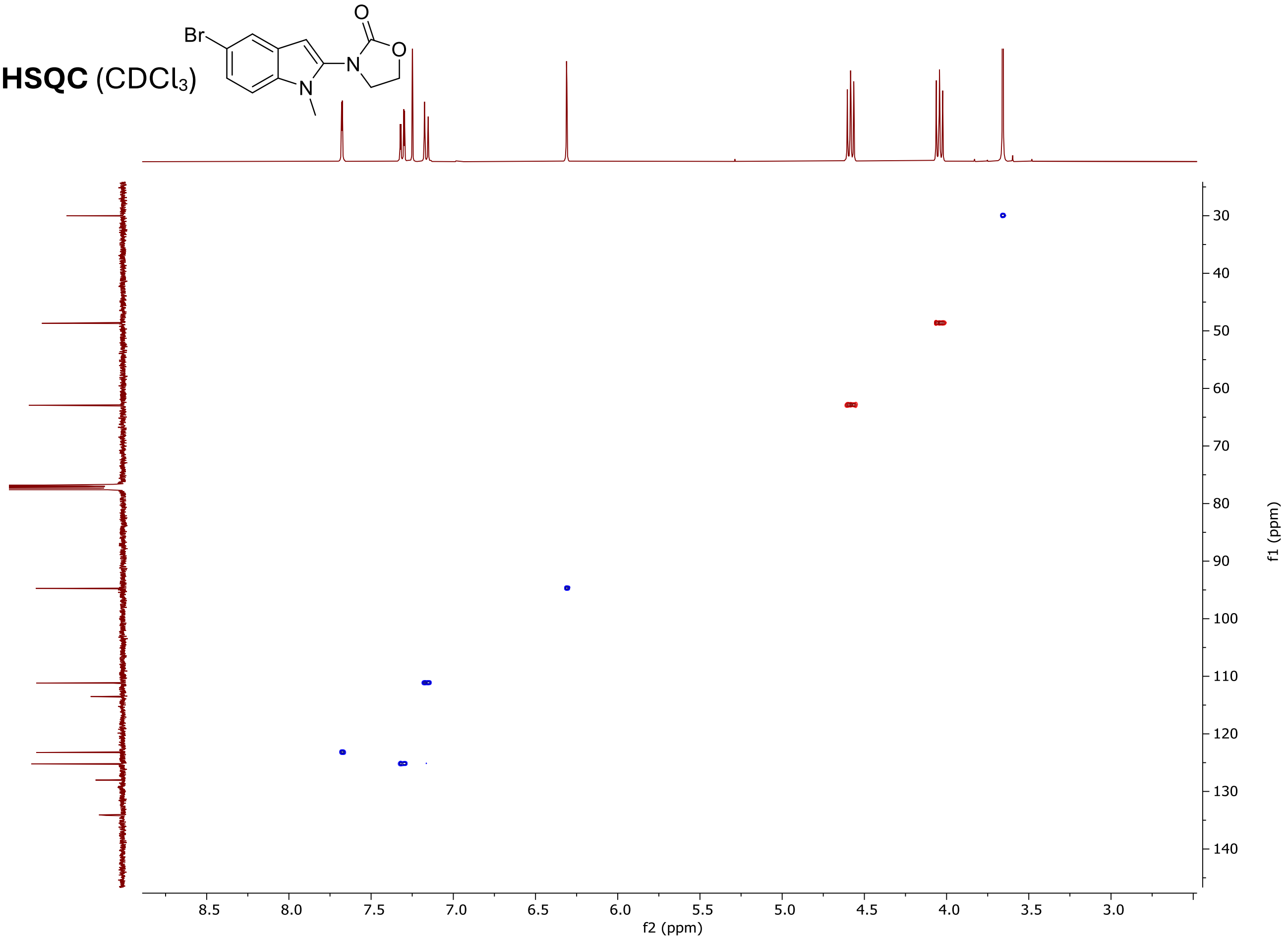

HMBC ($CDCl_3$)

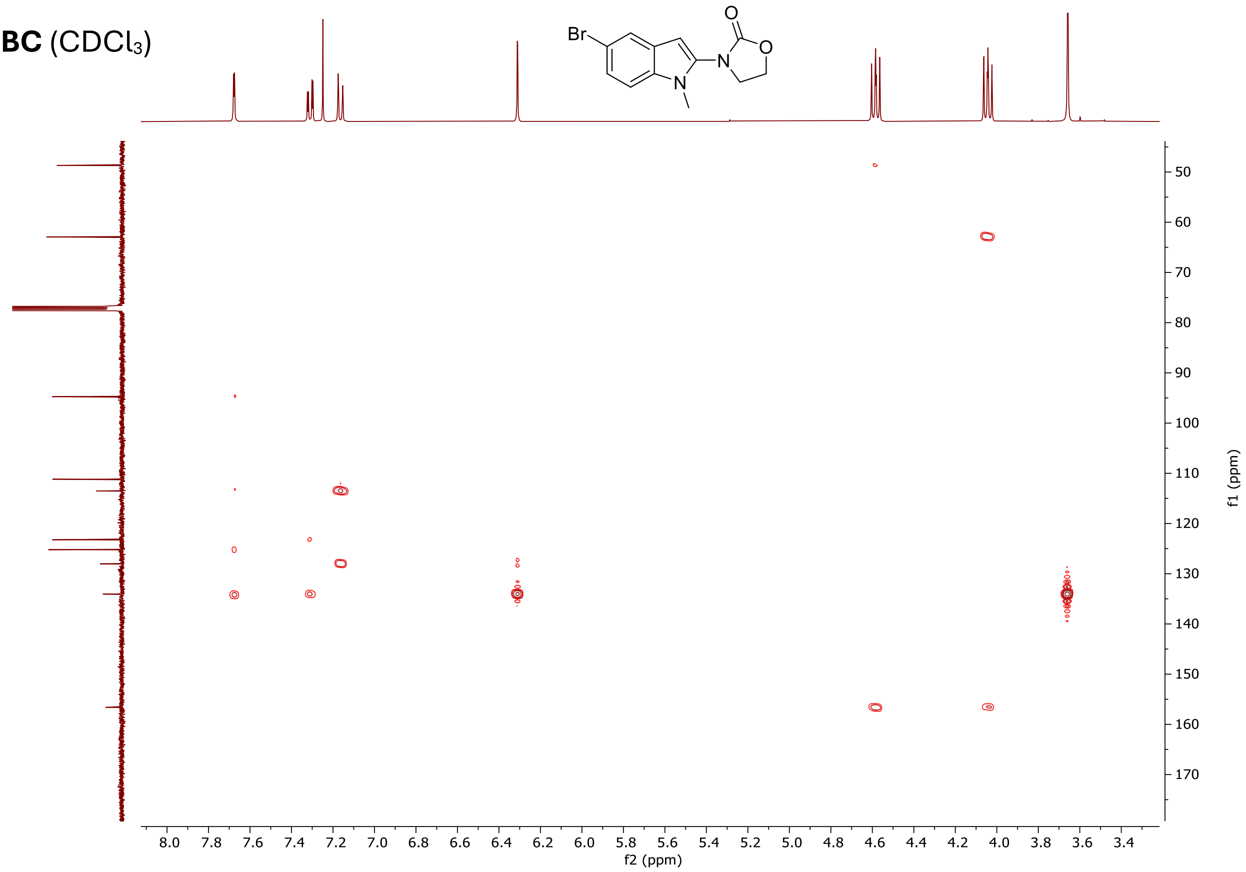

3-(1,5-dimethyl-1H-indol-2-yl)oxazolidin-2-one (5d)

^{1}H-NMR (300 MHz; $CDCl_3$)

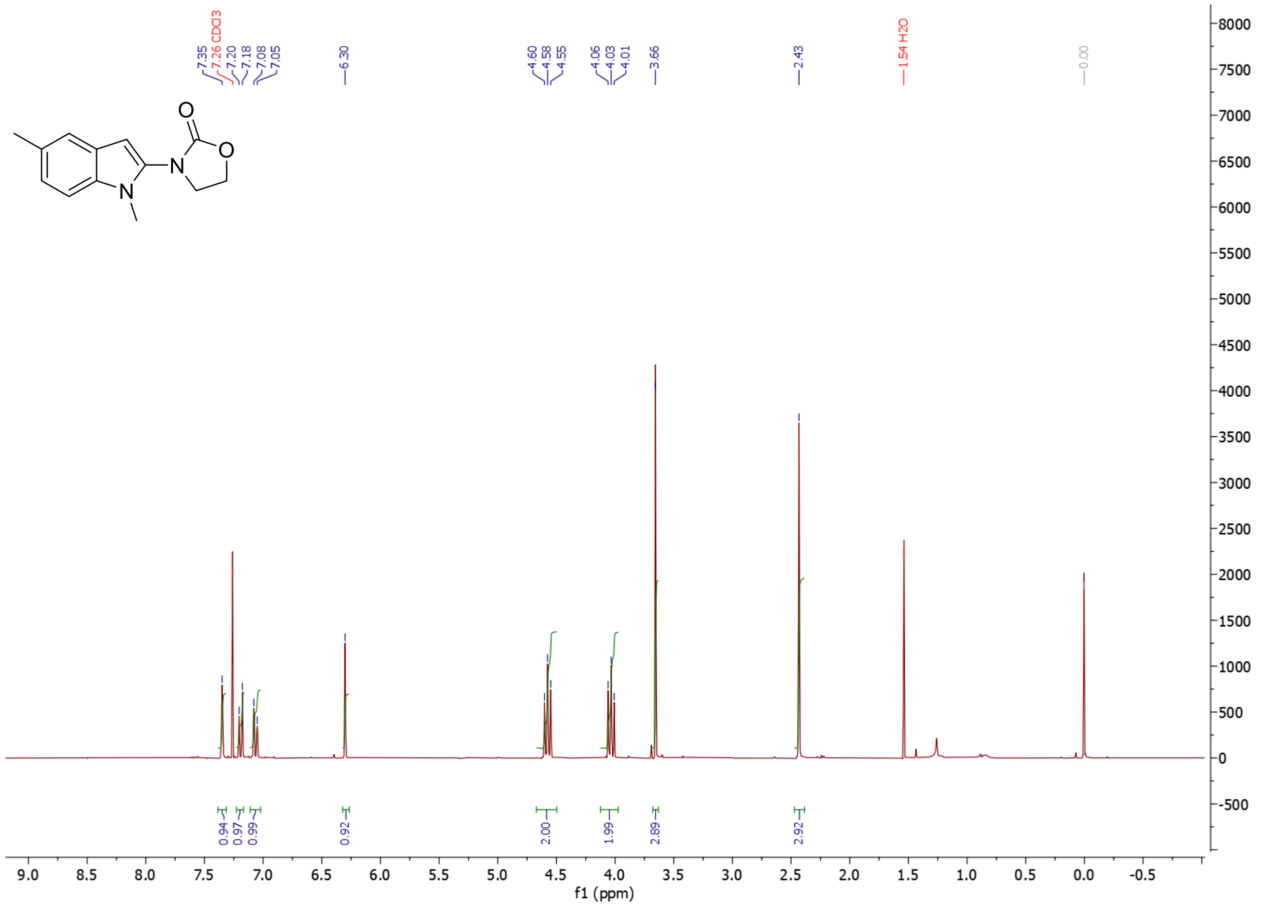

^{13}C-NMR (75 MHz; $CDCl_3$)

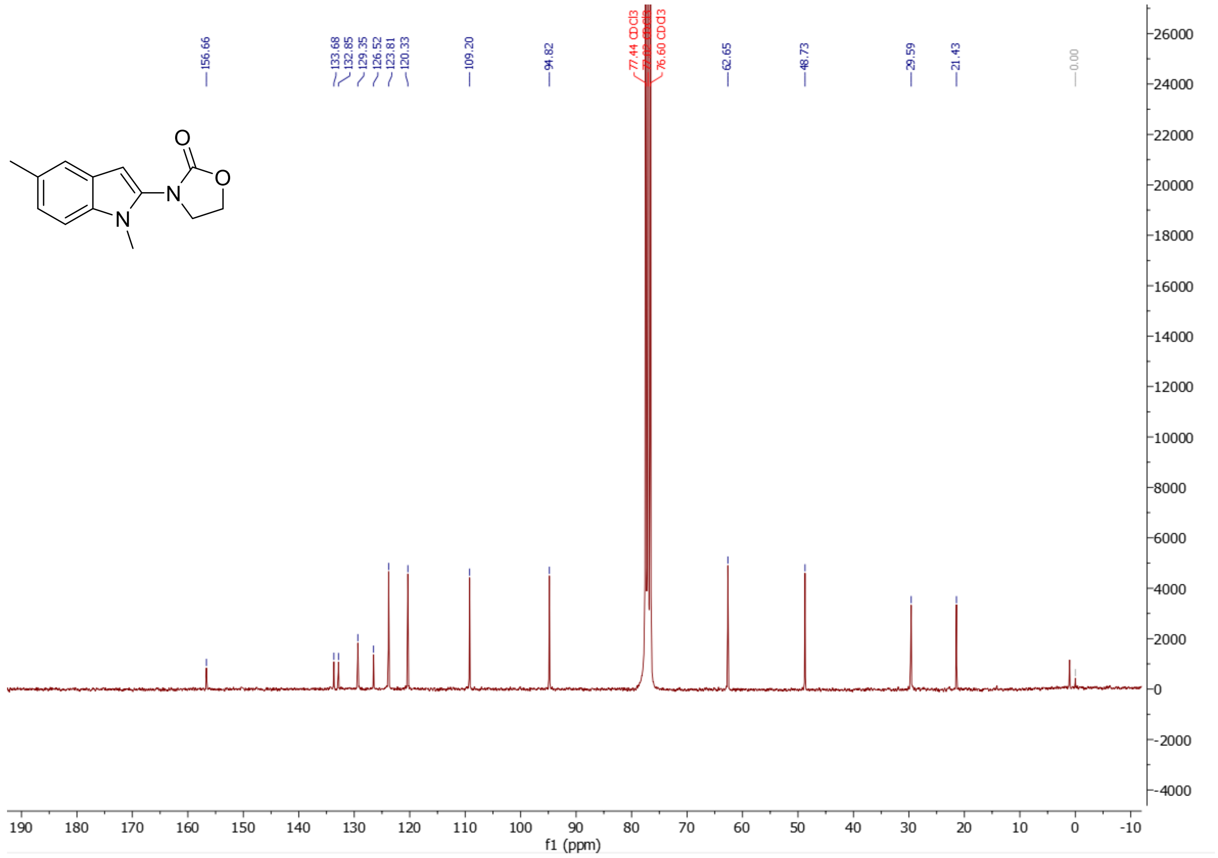

COSY ($CDCl_3$)

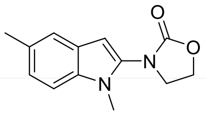

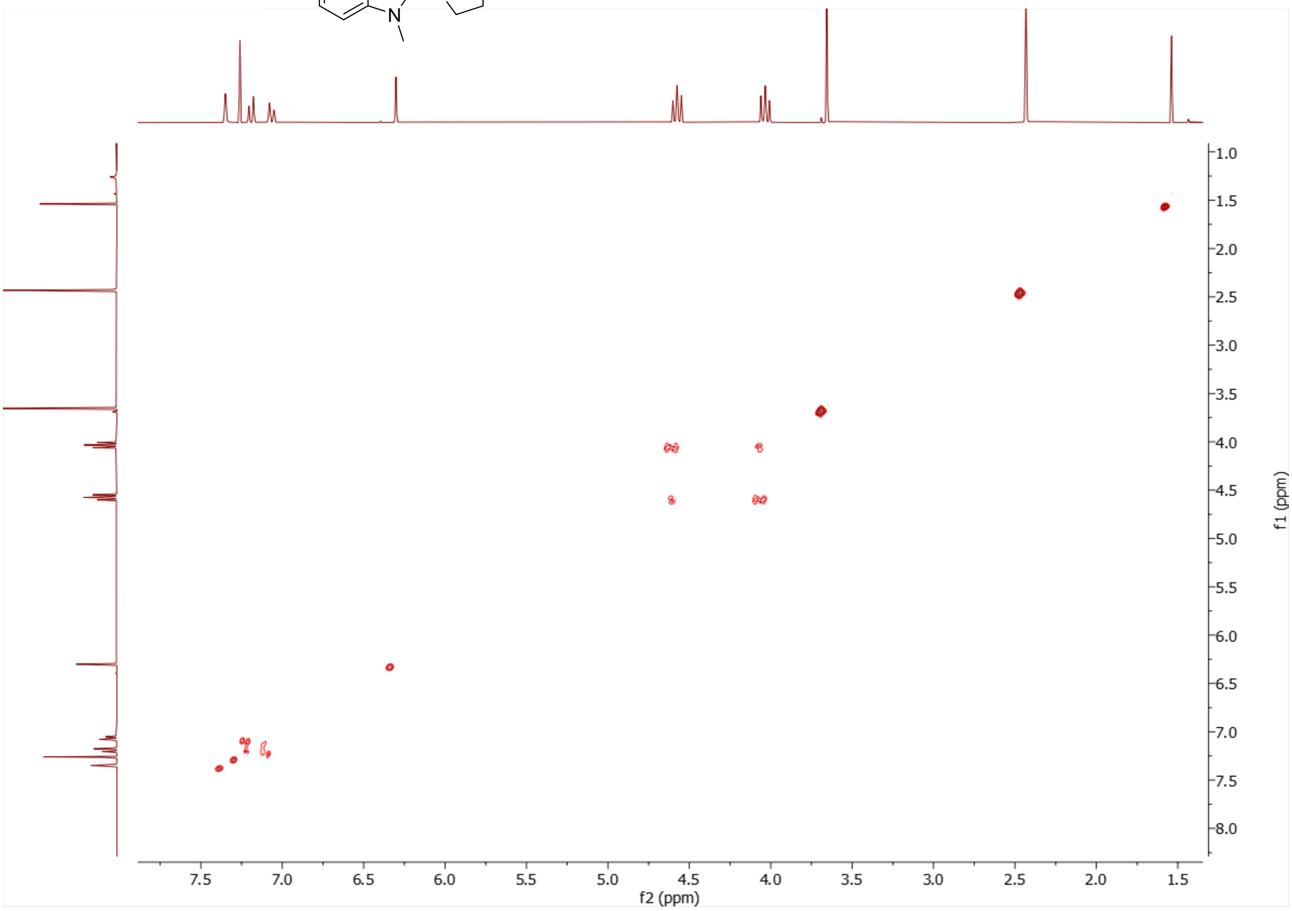

HSQC ($CDCl_3$)

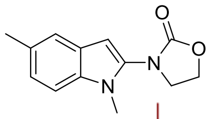

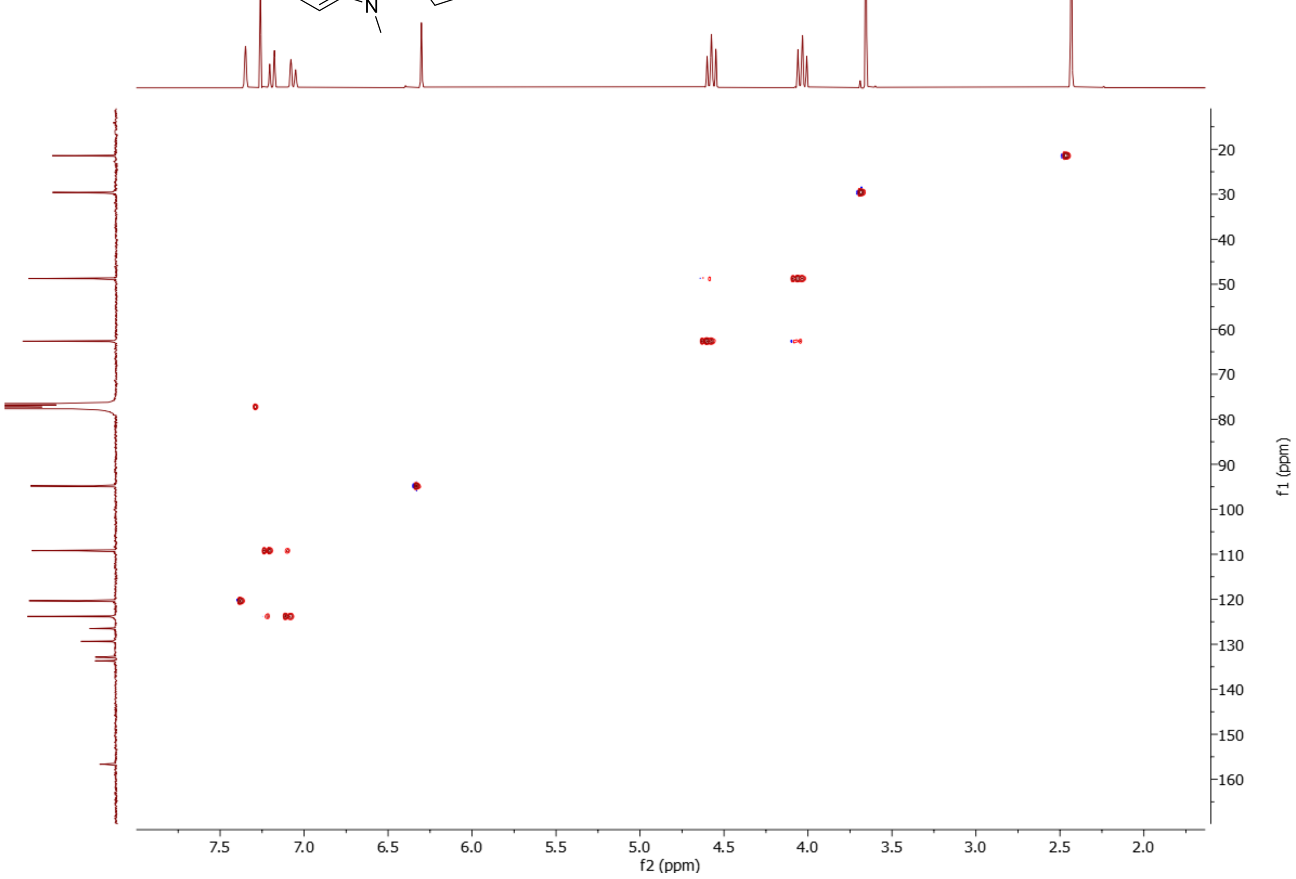

HMBC (CDCl$_3$)

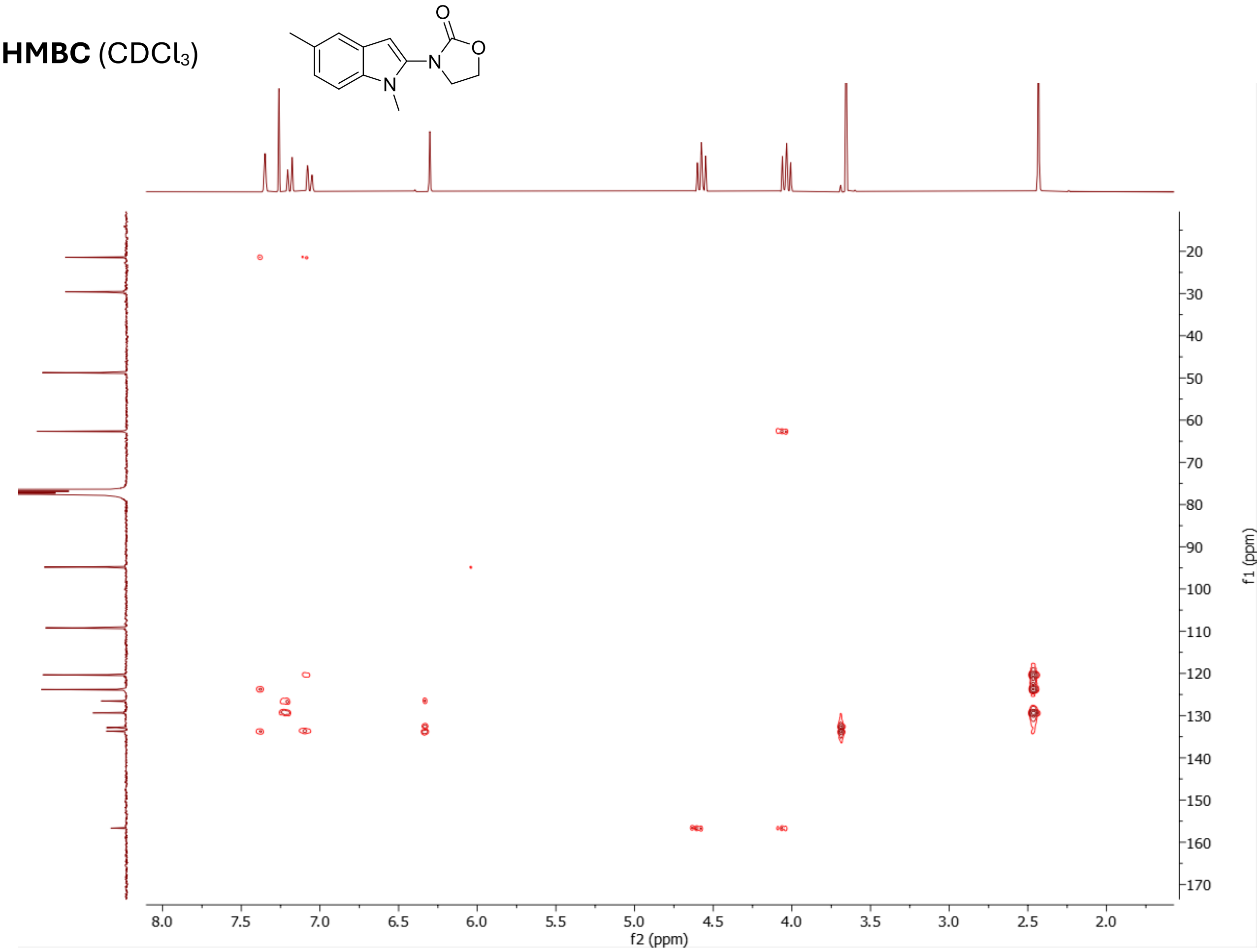

3-(5-methoxy-1-methyl-1H-indol-2-yl)oxazolidin-2-one (5e) ^{1}**H-NMR** (400 MHz; CDCl$_3$)

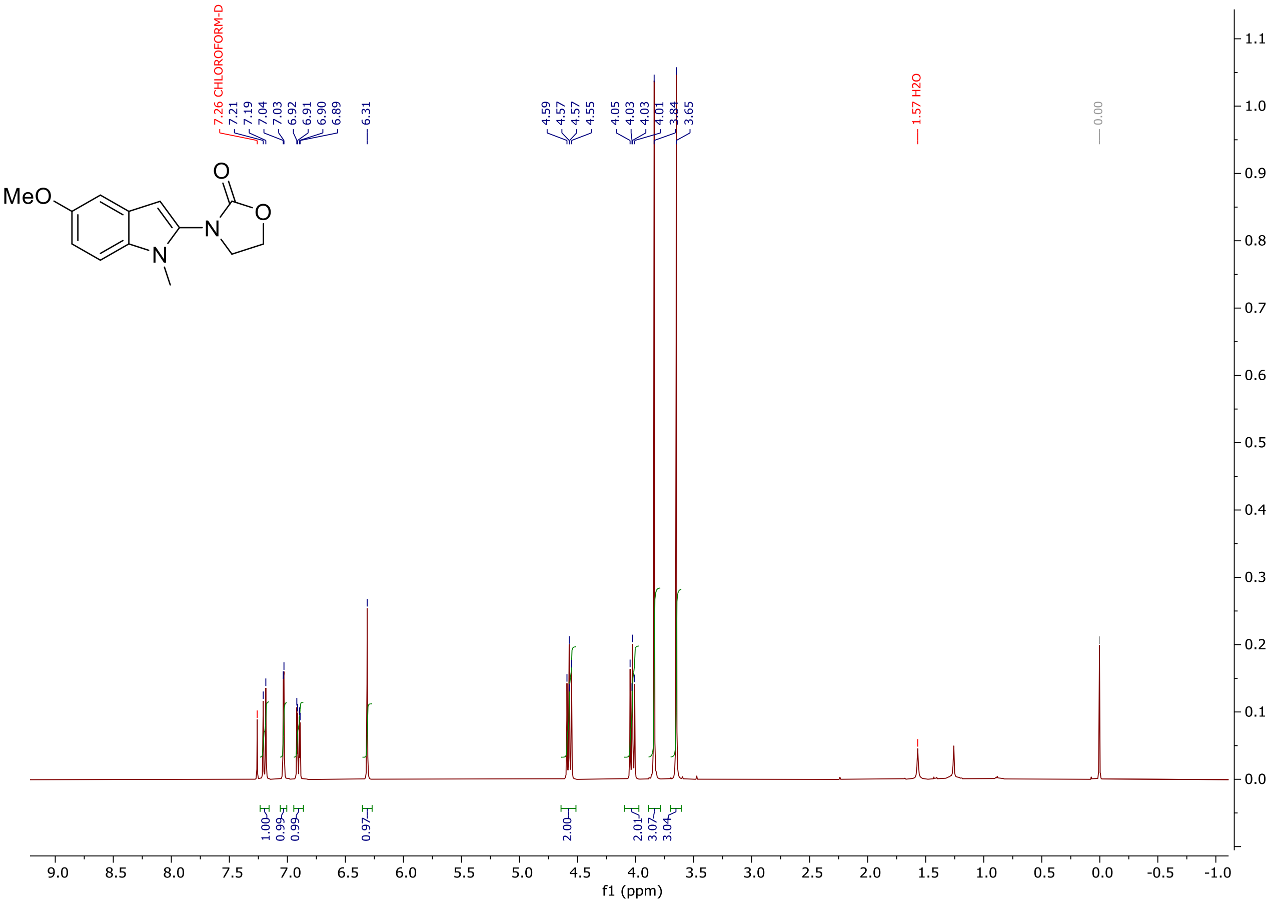

^{13}C-NMR (101 MHz; $CDCl_3$)

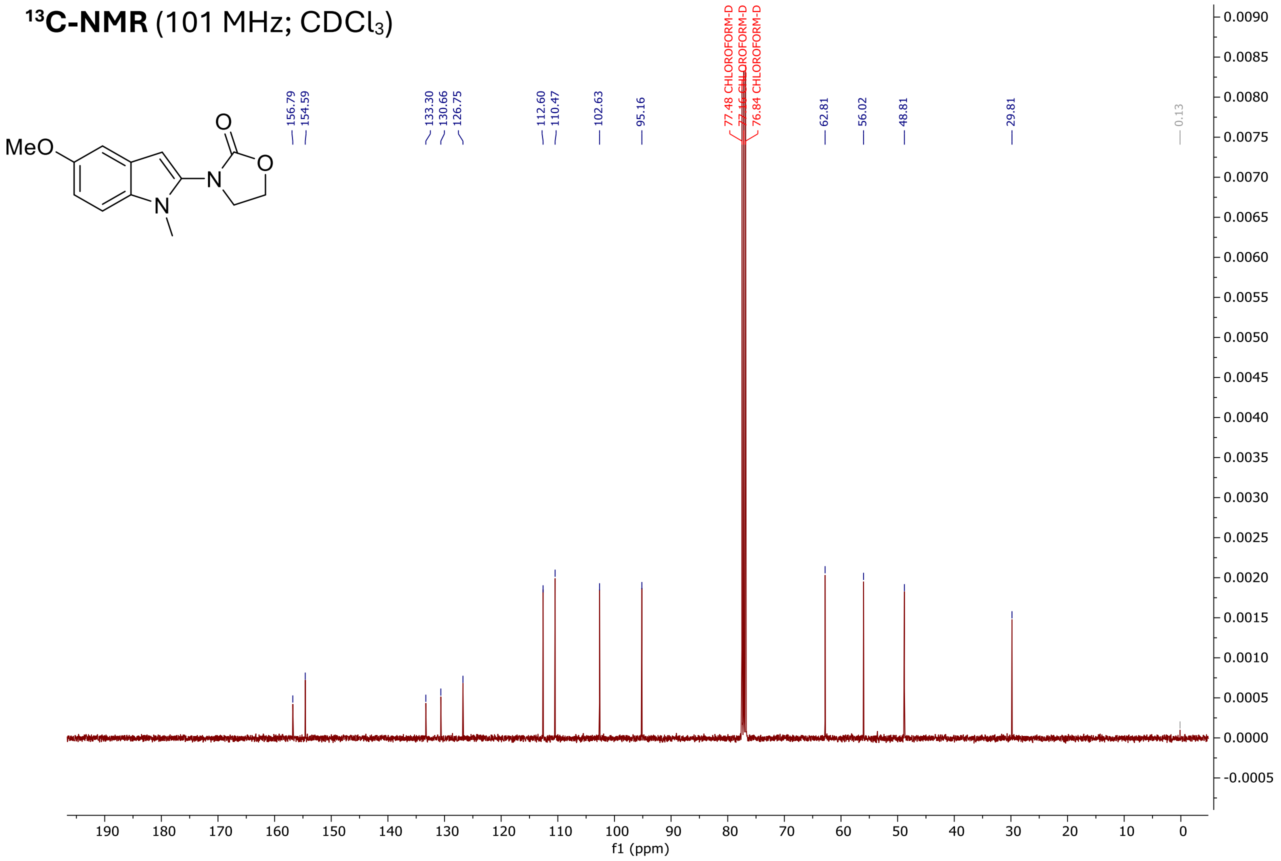

COSY ($CDCl_3$)

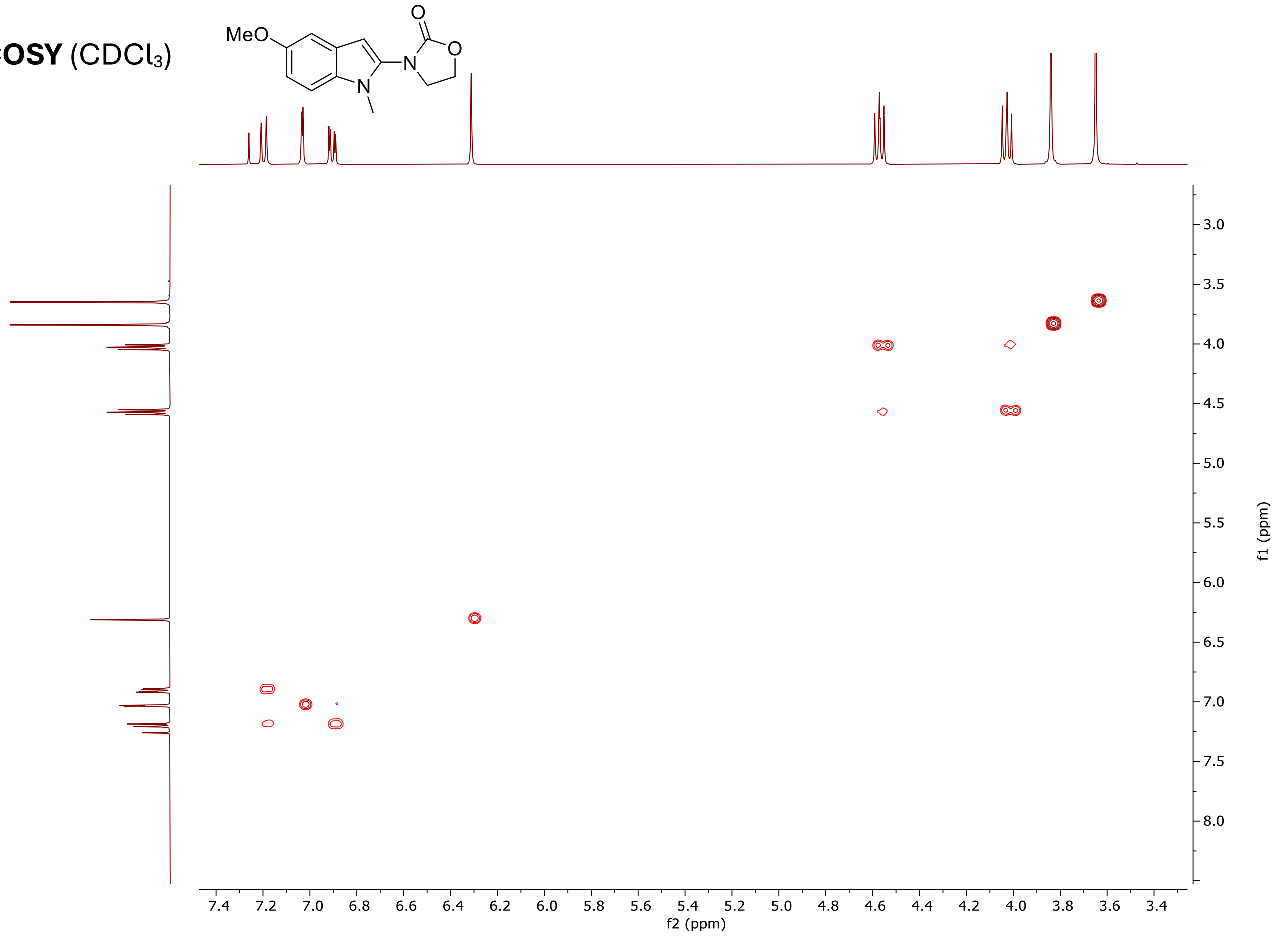

HSQC ($CDCl_3$)

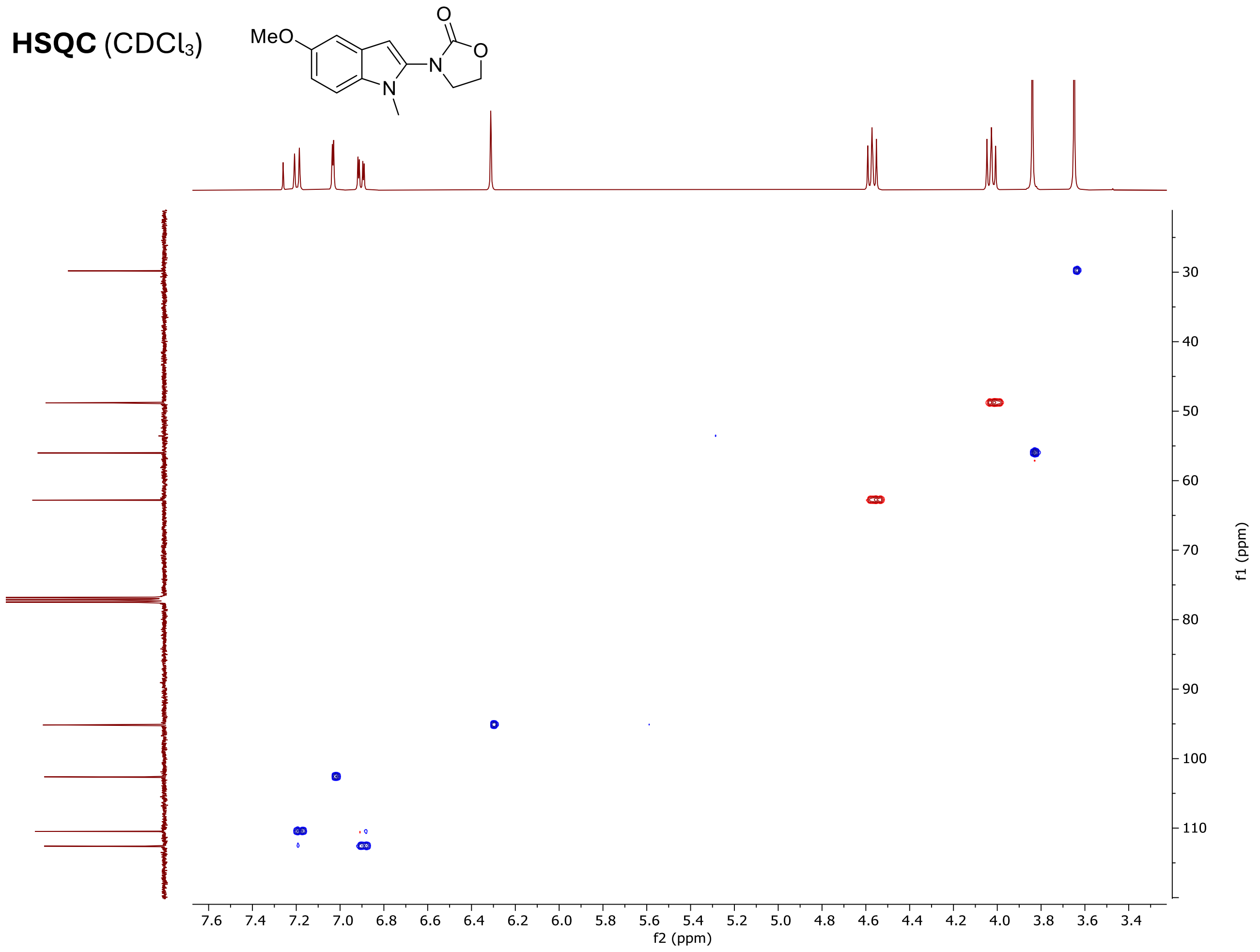

HMBC ($CDCl_3$)

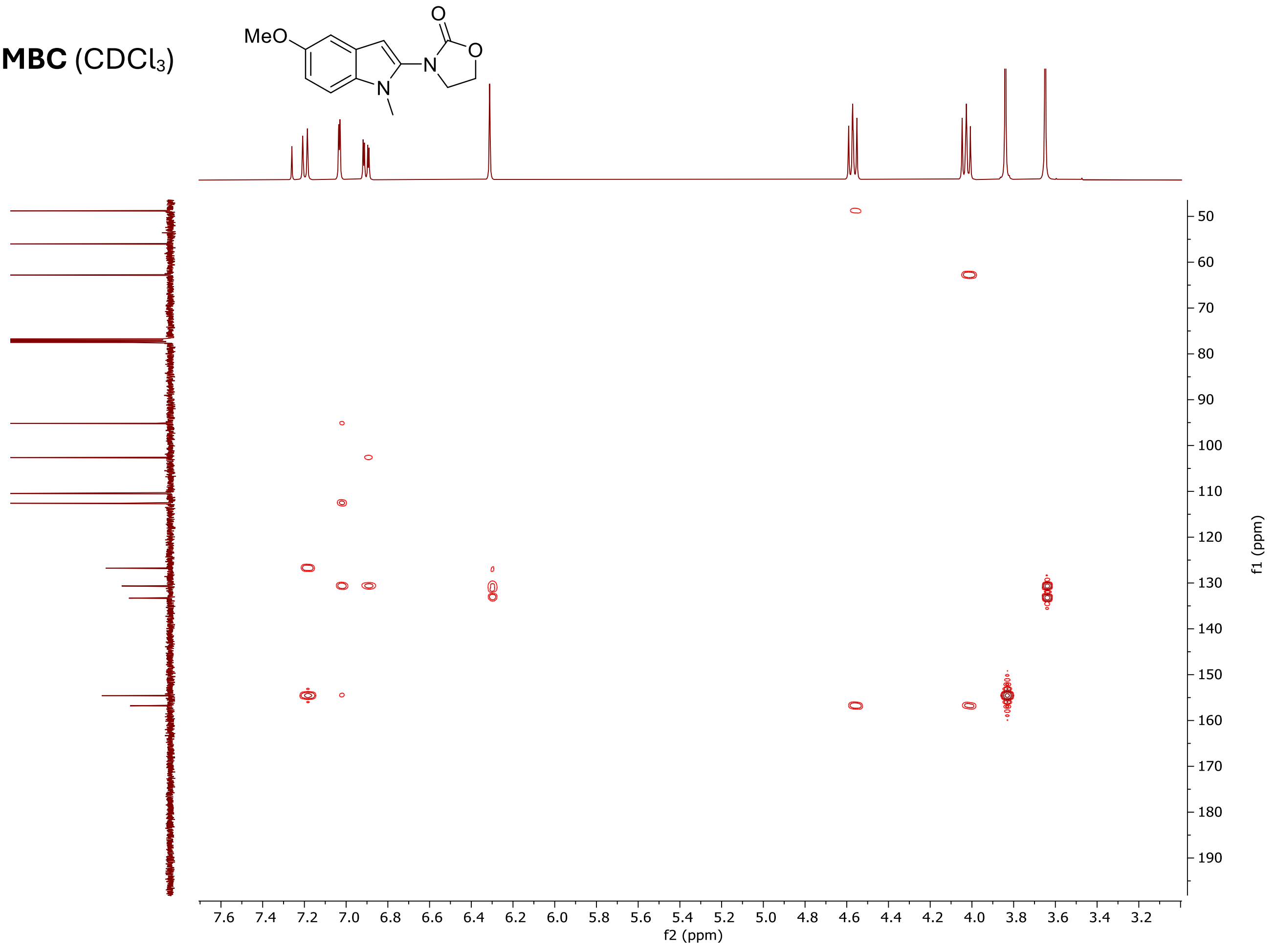

1-methyl-2-(2-oxooxazolidin-3-yl)-1H-indole-3-carbonitrile (5f) **^{1}H-NMR** (300 MHz; $CDCl_3$)

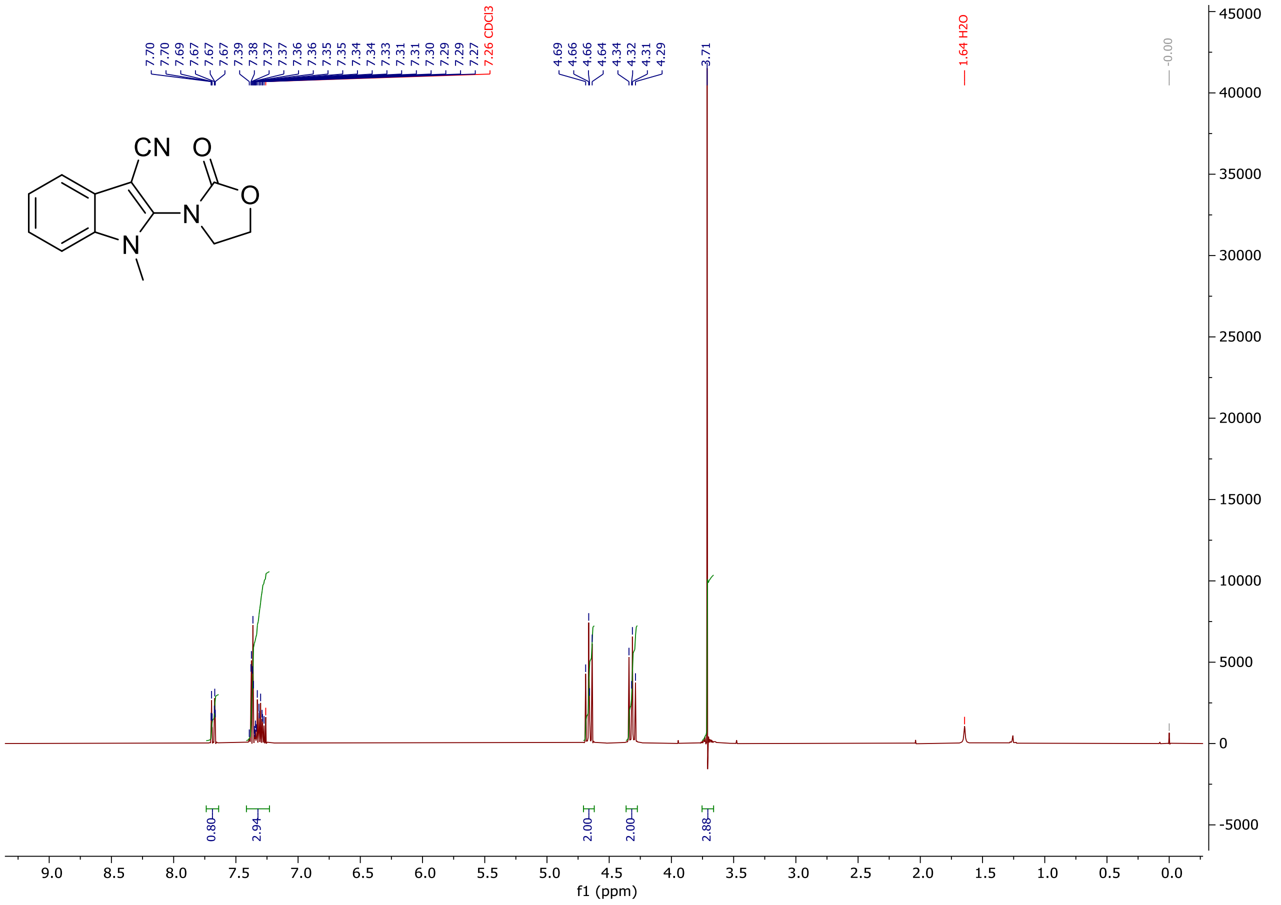

^{13}C-NMR (75 MHz; $CDCl_3$)

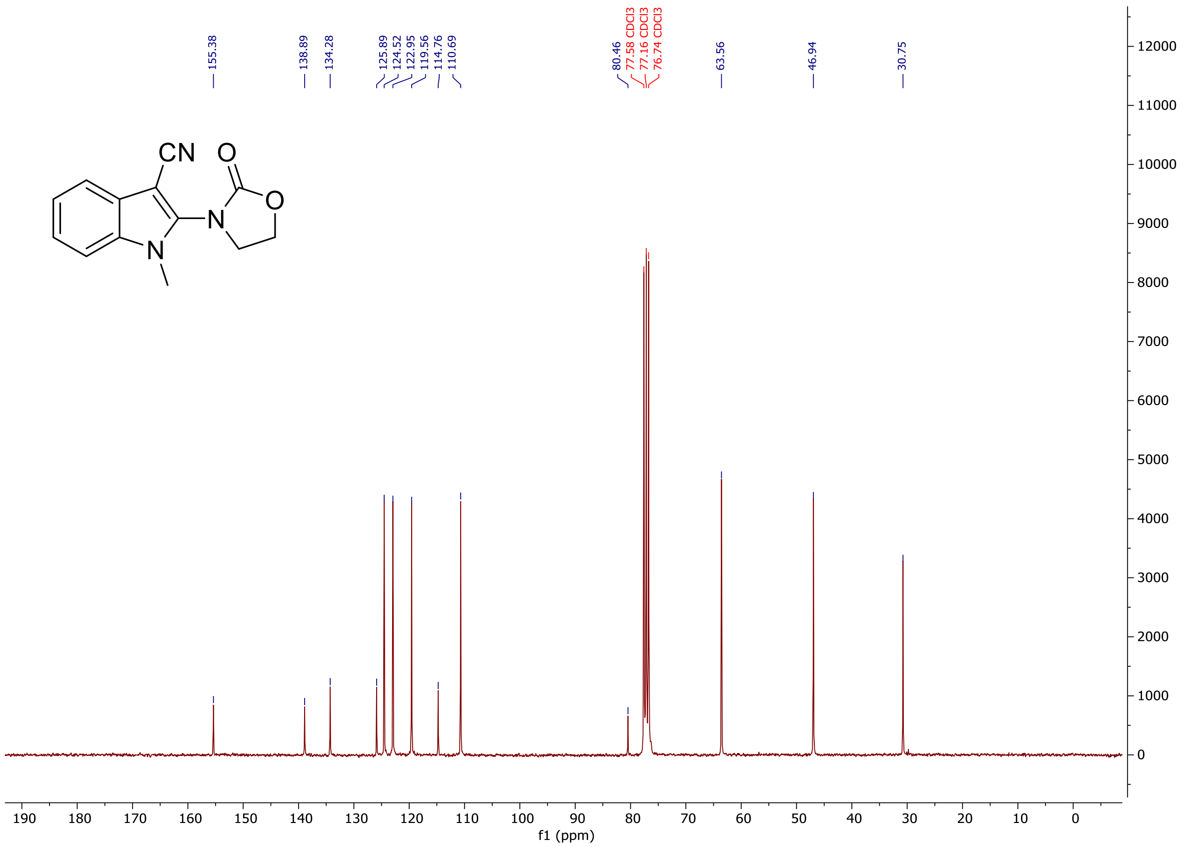

COSY ($CDCl_3$)

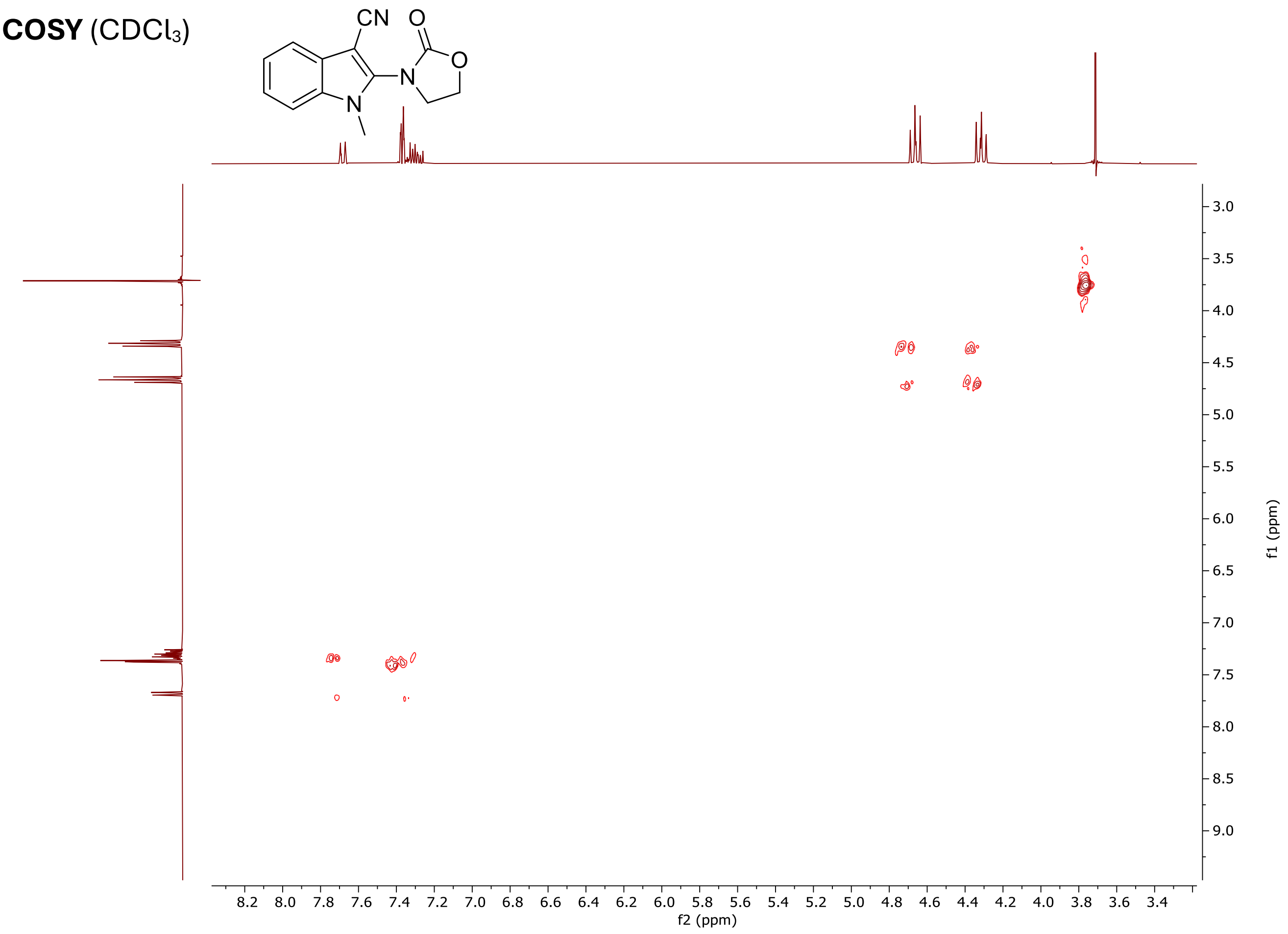

HSQC ($CDCl_3$)

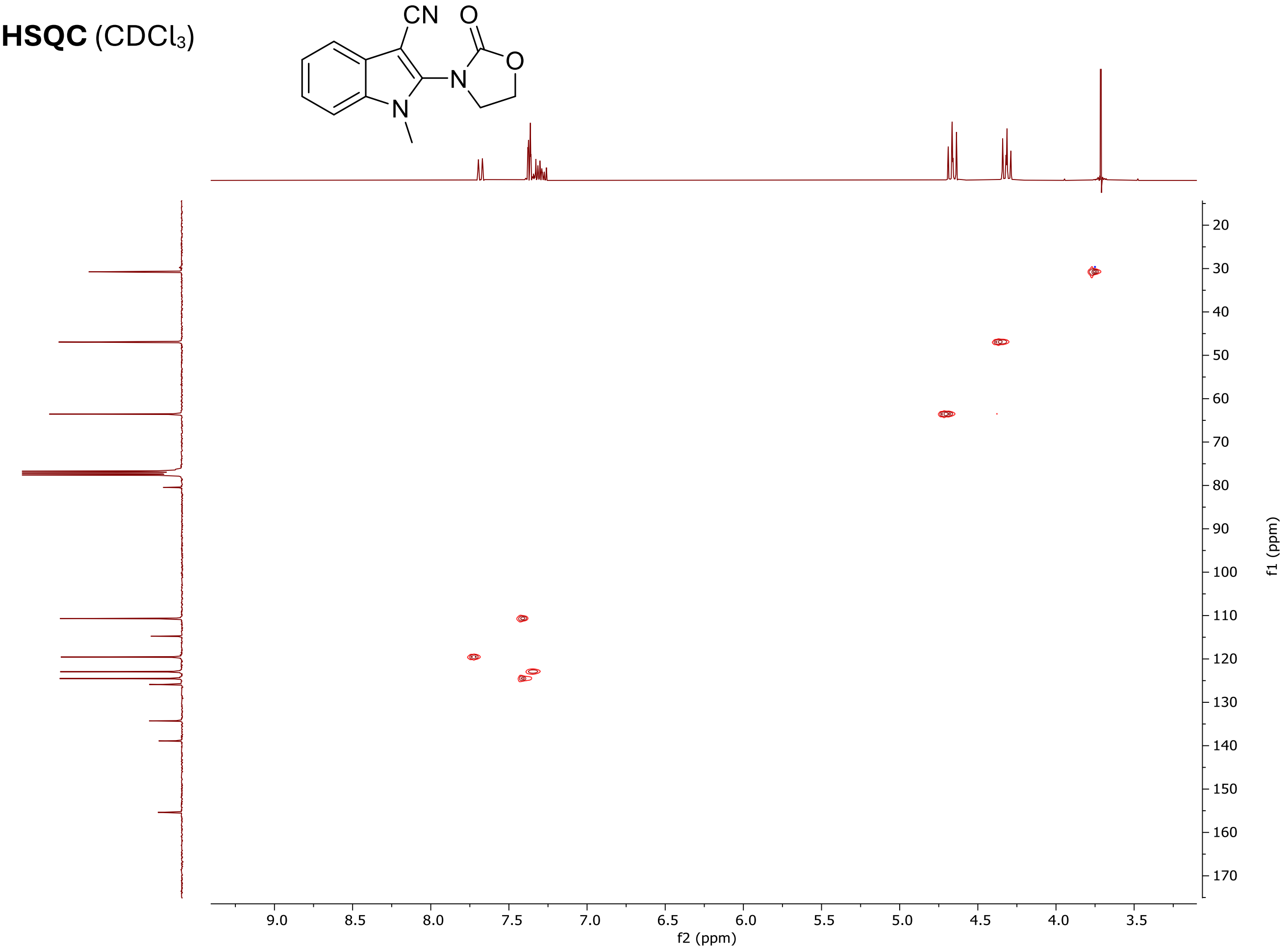

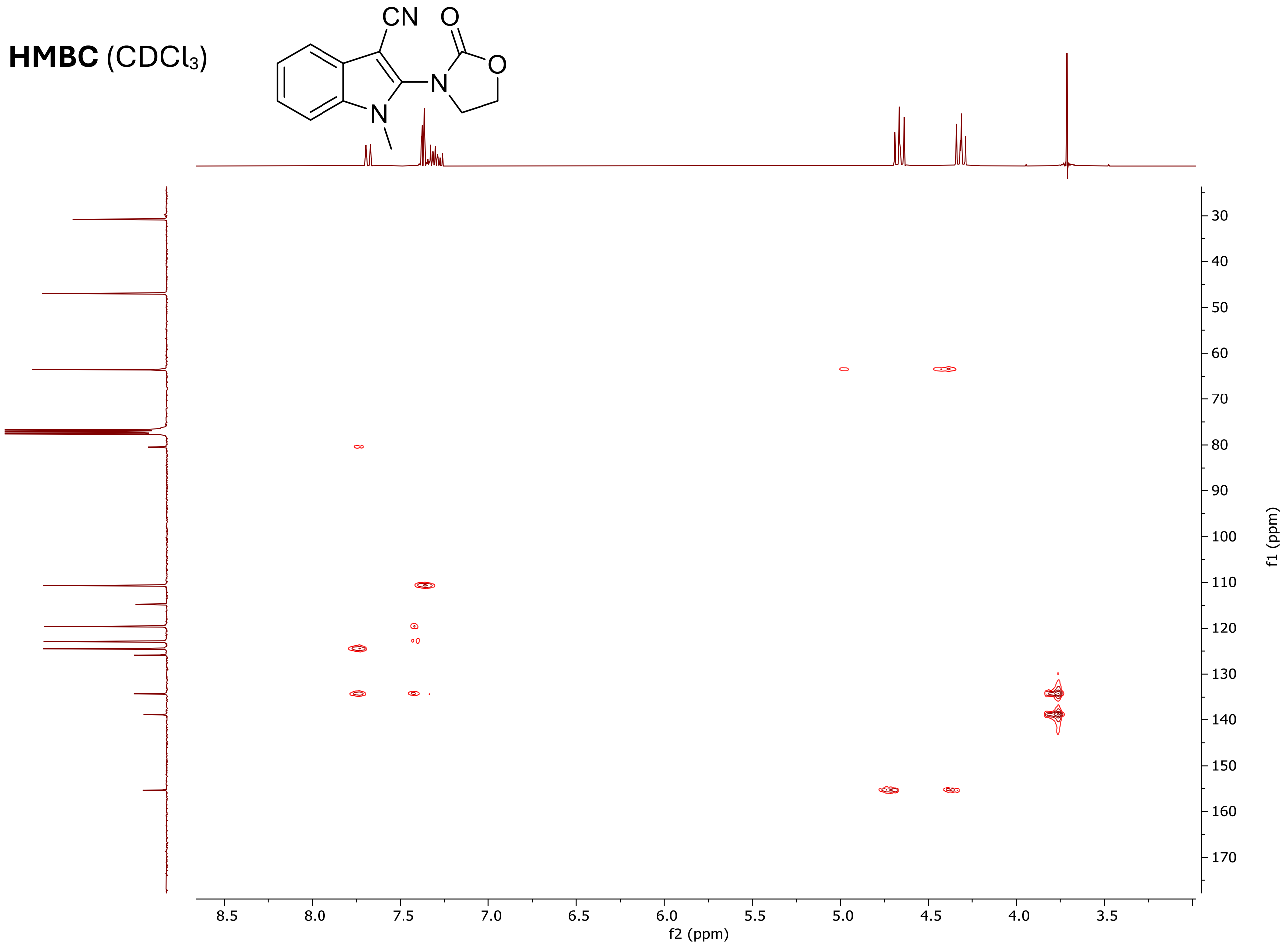

ethyl 1-methyl-2-(2-oxooxazolidin-3-yl)-1H-indole-3-carboxylate (5g) ^{1}H-NMR (300 MHz; $CDCl_3$)

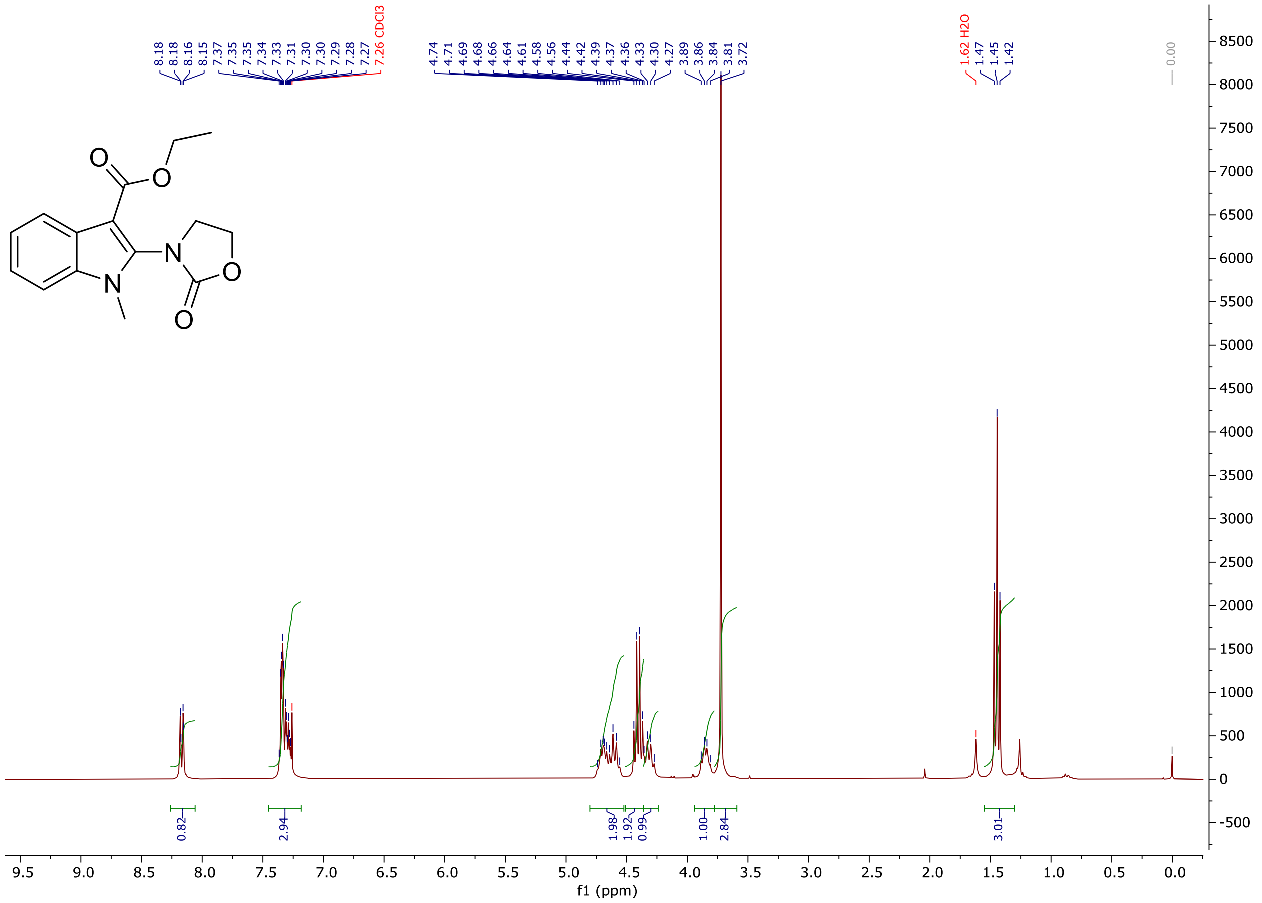

^{13}C-NMR (75 MHz; $CDCl_3$)

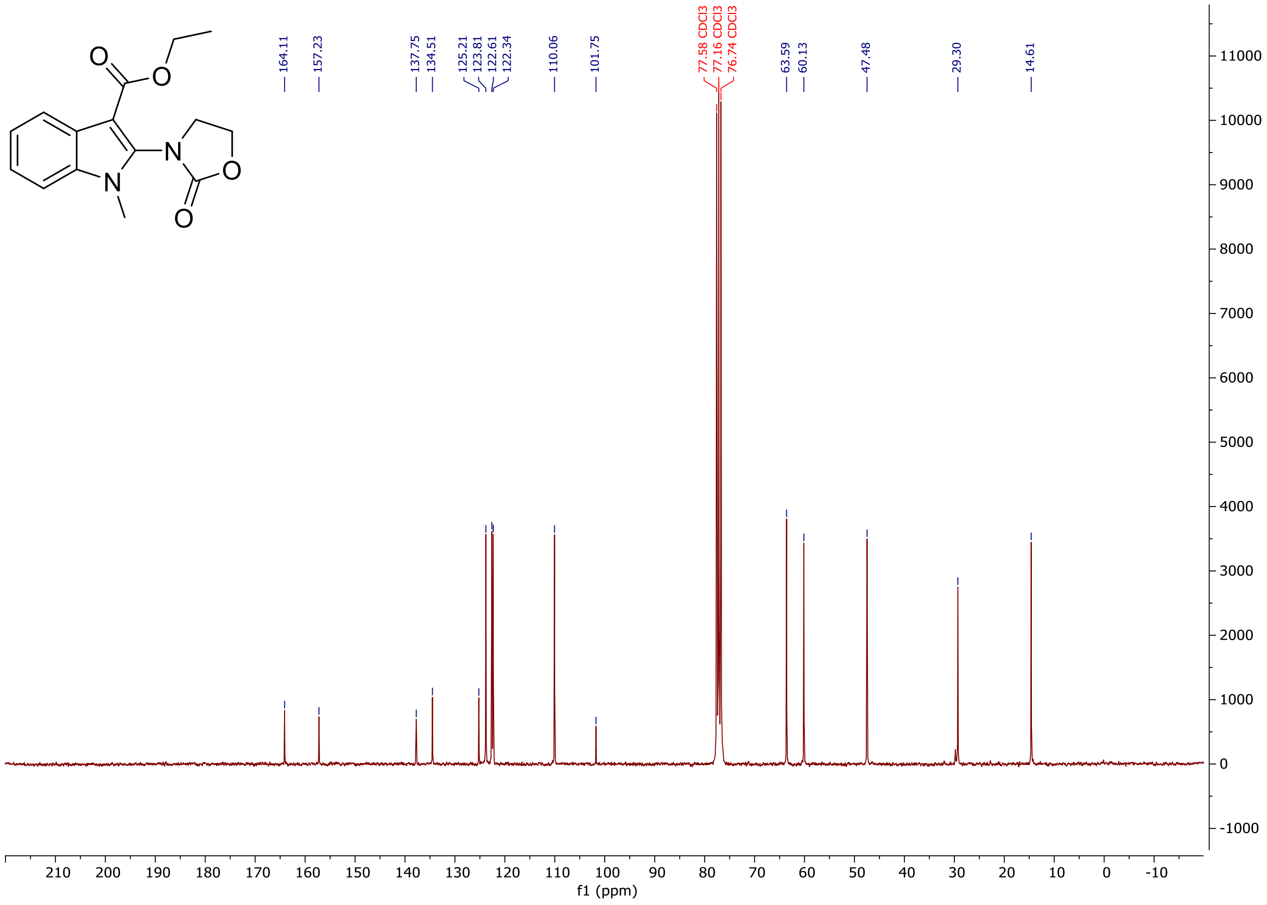

COSY ($CDCl_3$)

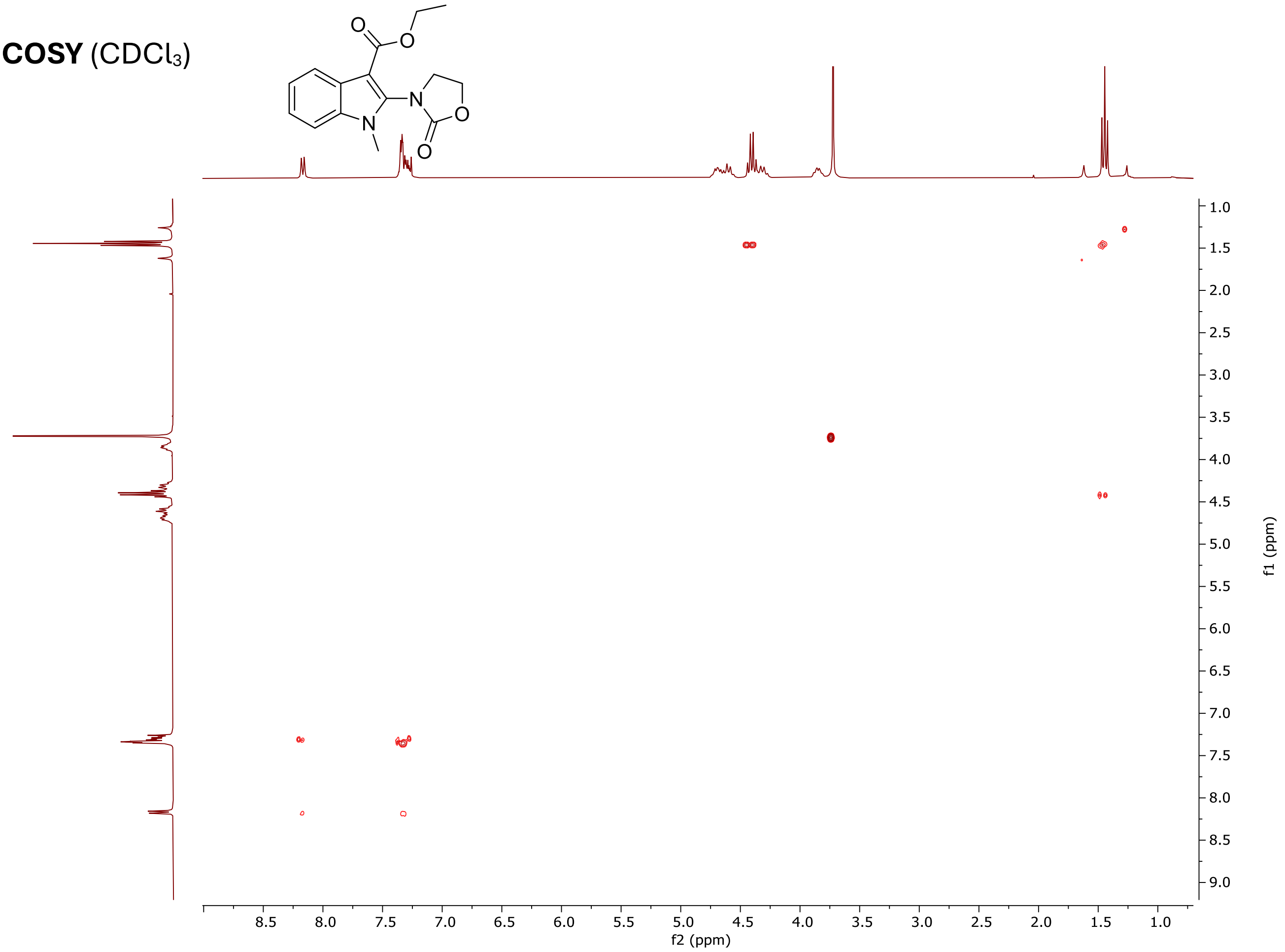

HSQC ($CDCl_3$)

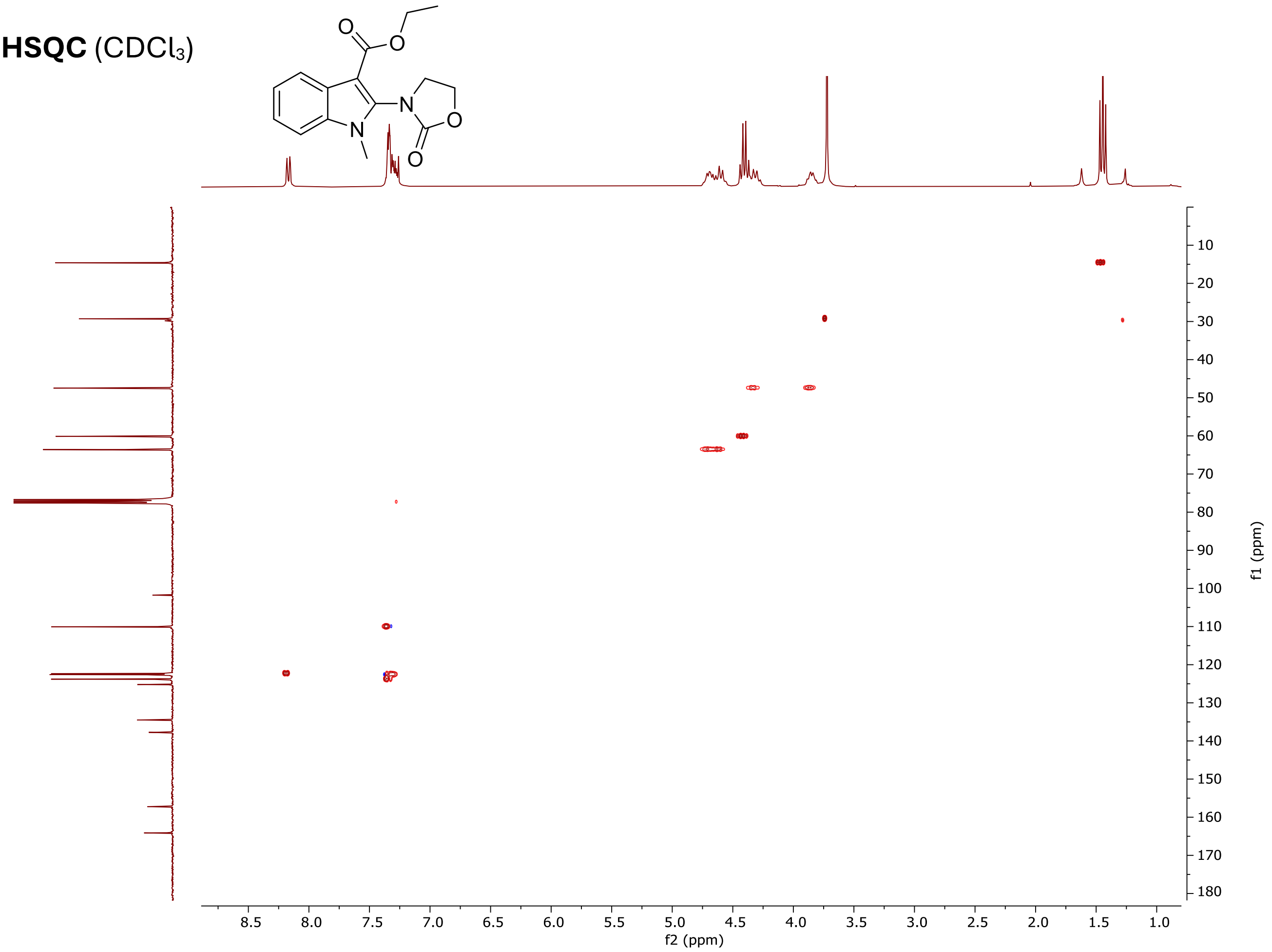

HMBC ($CDCl_3$)

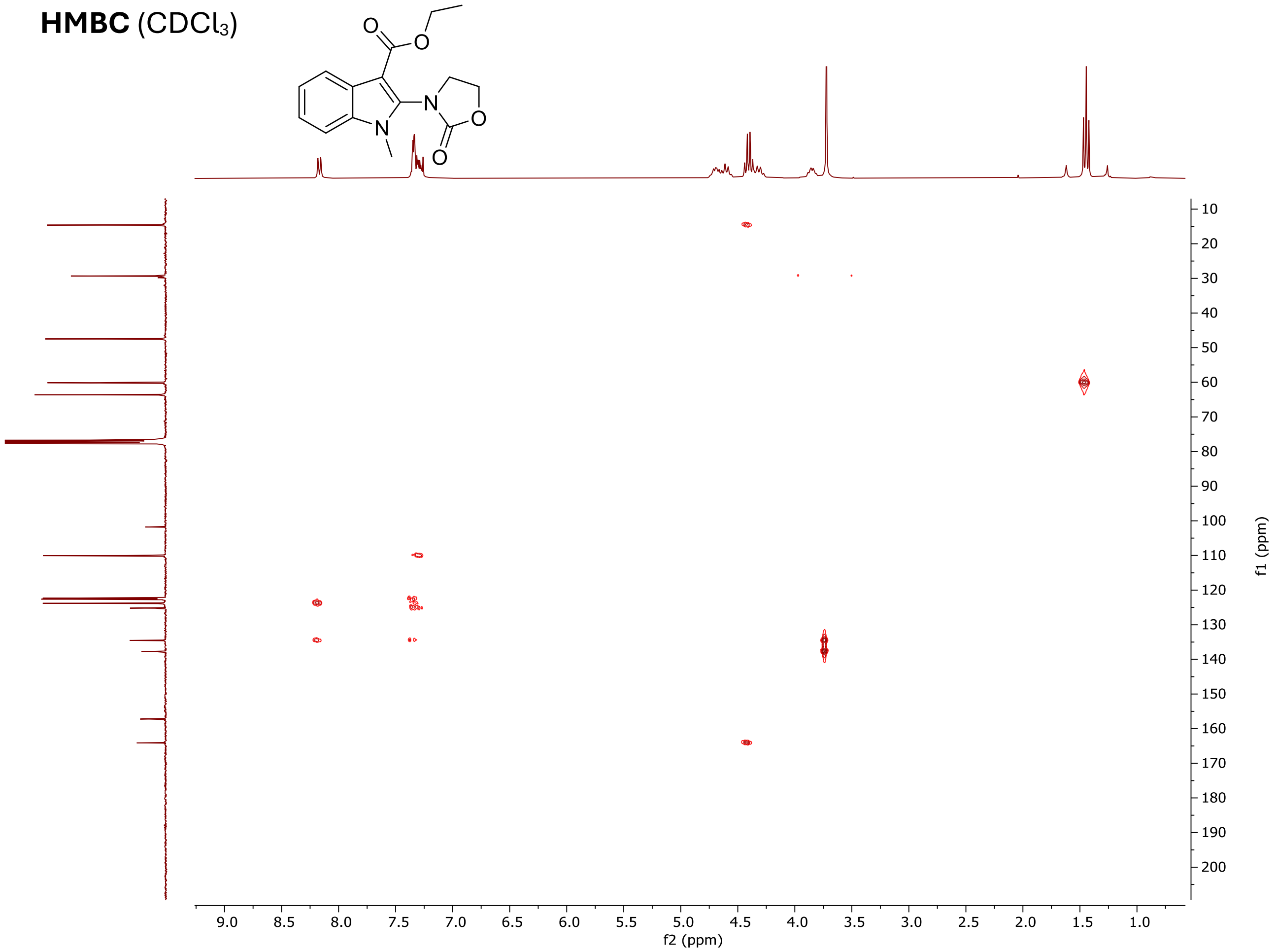

3-(1-phenyl-1H-indol-2-yl)oxazolidin-2-one (5h)

^{1}H-NMR (400 MHz; $CDCl_3$)

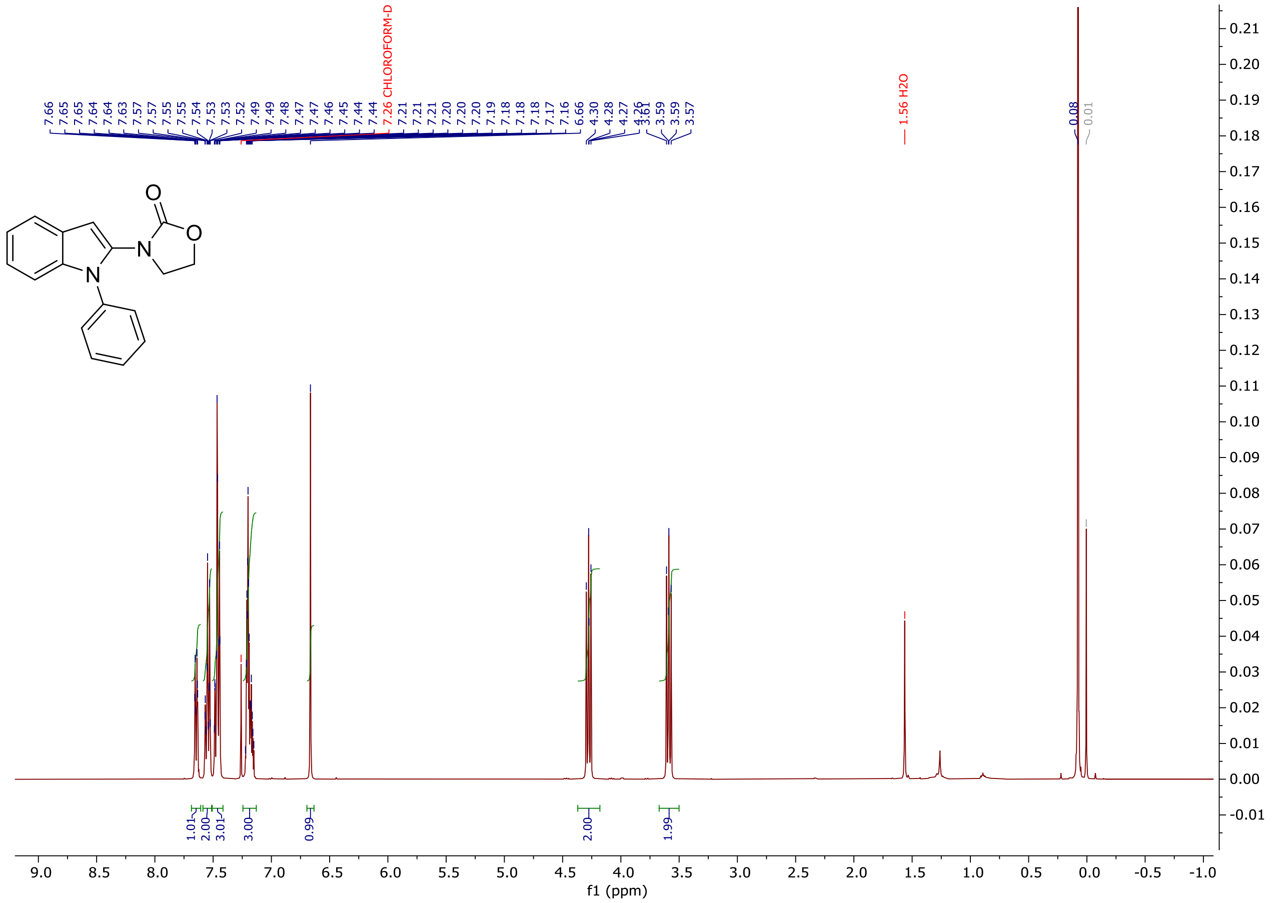

^{13}C-NMR (101 MHz; $CDCl_3$)

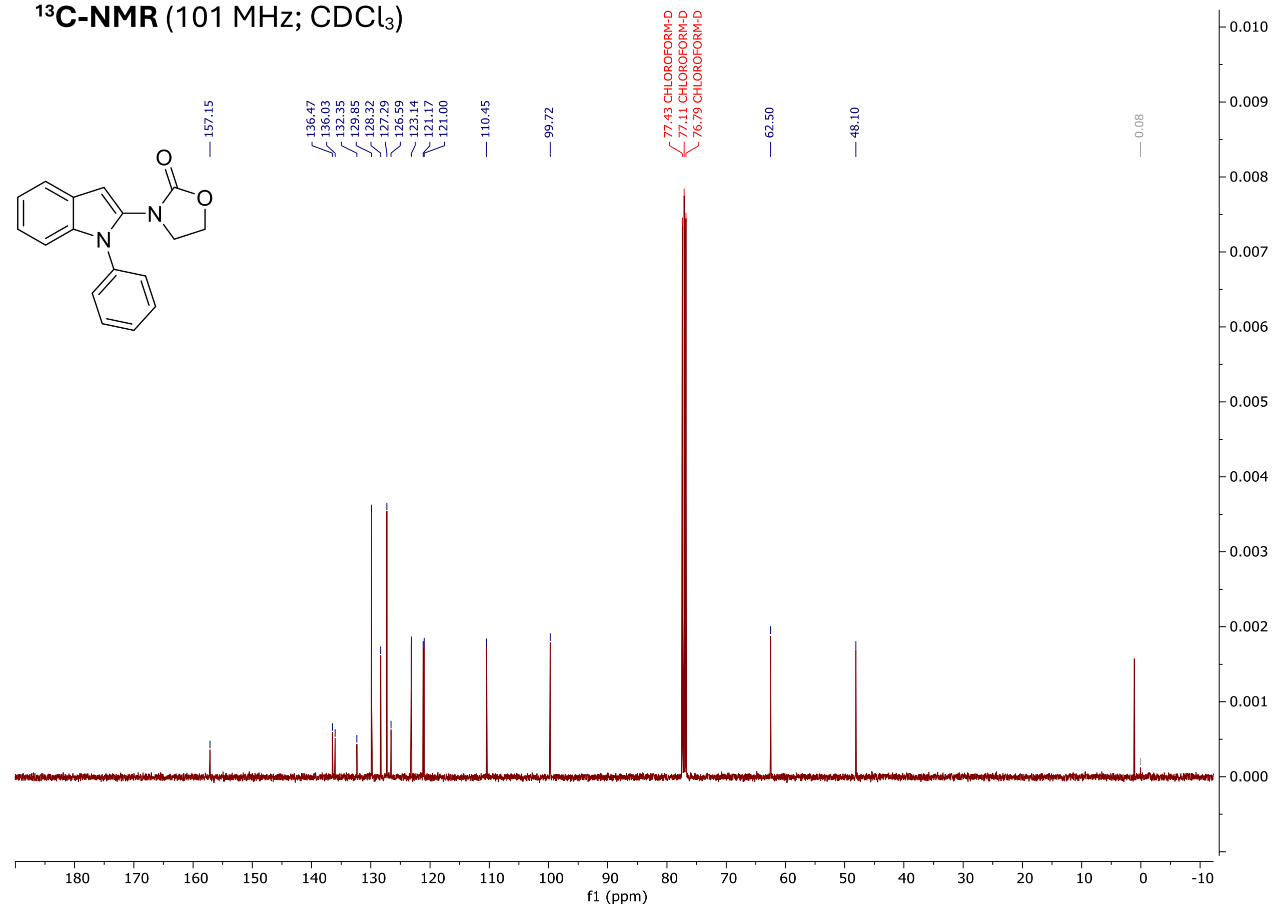

COSY ($CDCl_3$)

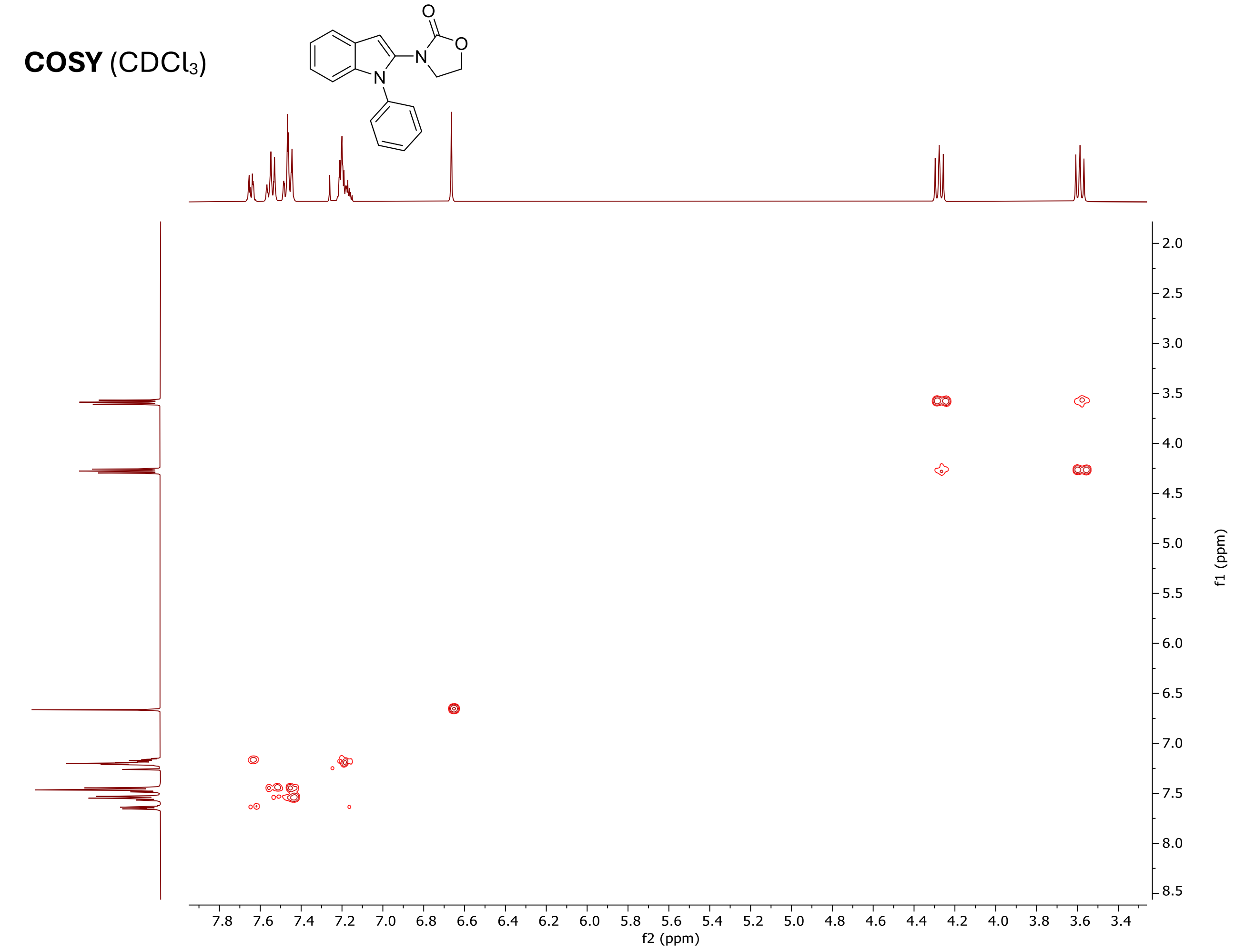

HSQC ($CDCl_3$)

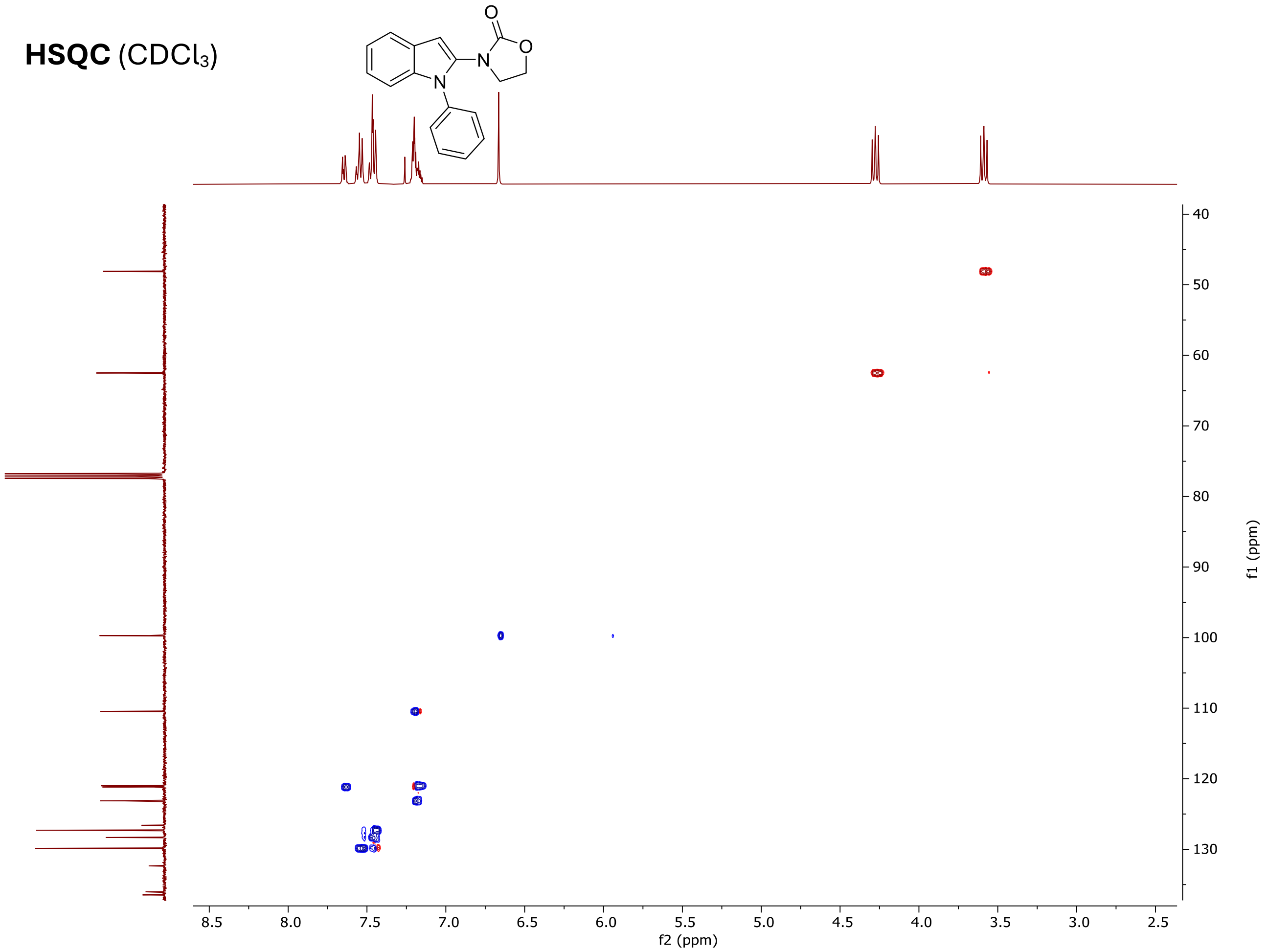

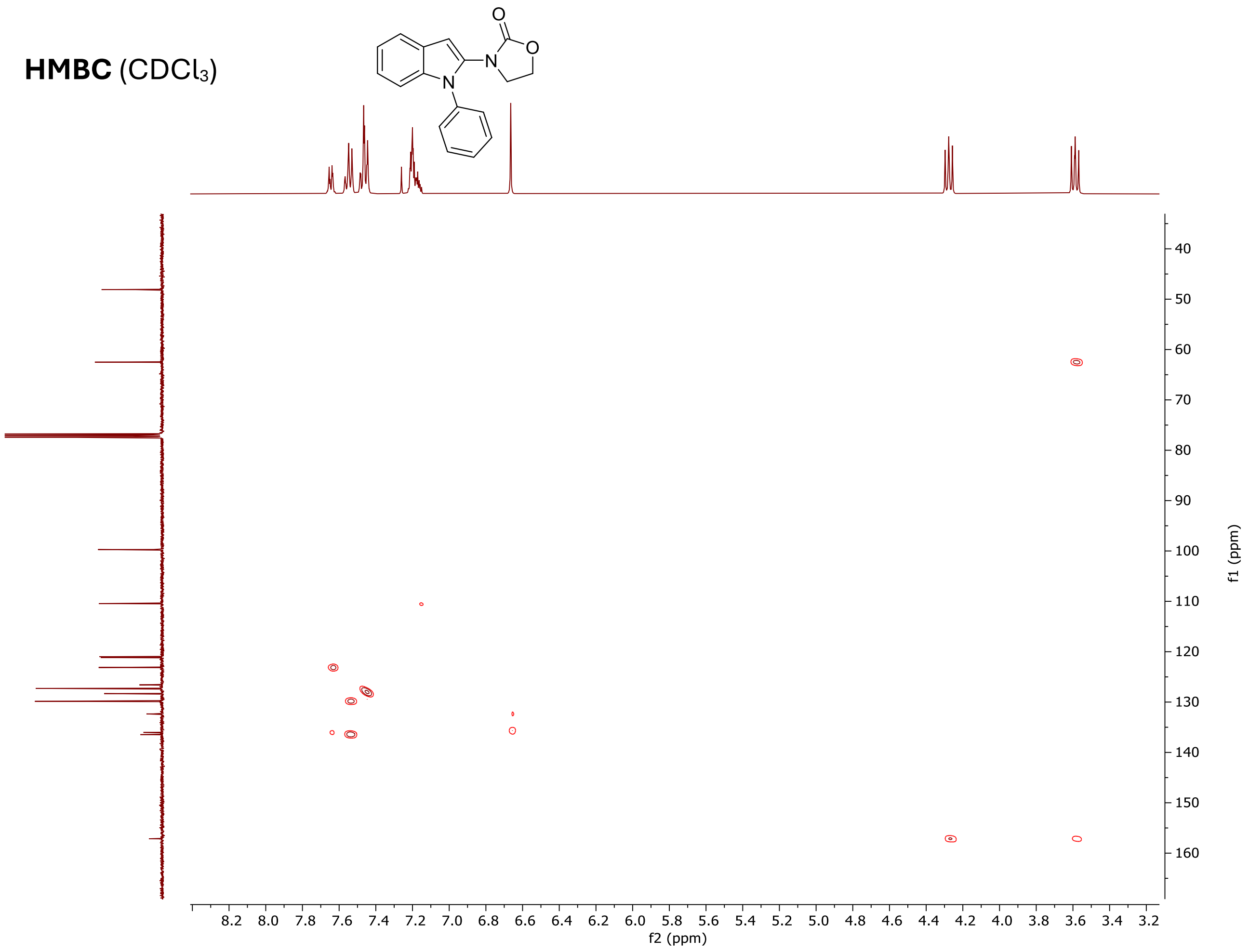

3-(5-bromo-1H-indol-2-yl)oxazolidin-2-one (5i)

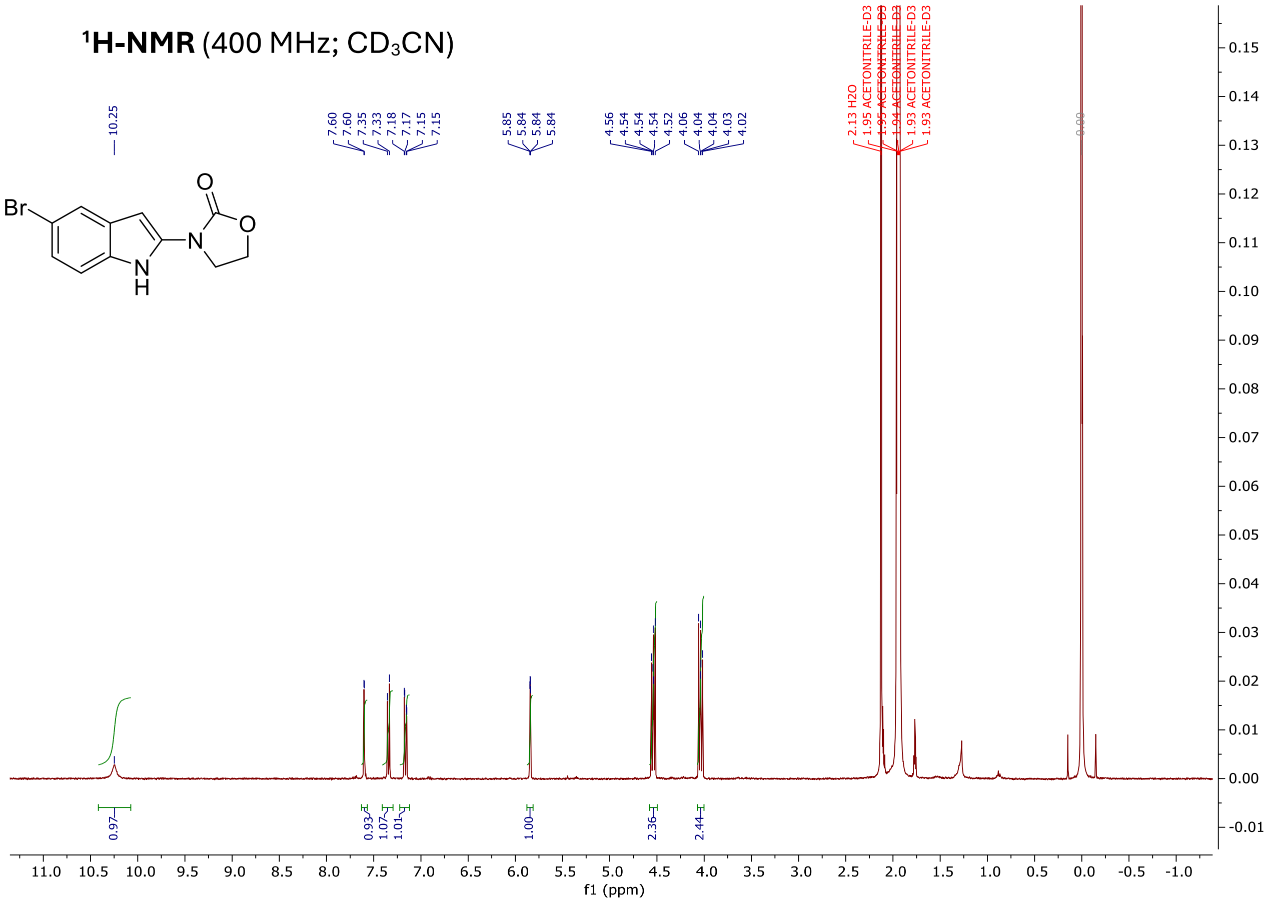

^{13}**C-NMR** (151 MHz; $CDCl_3$)

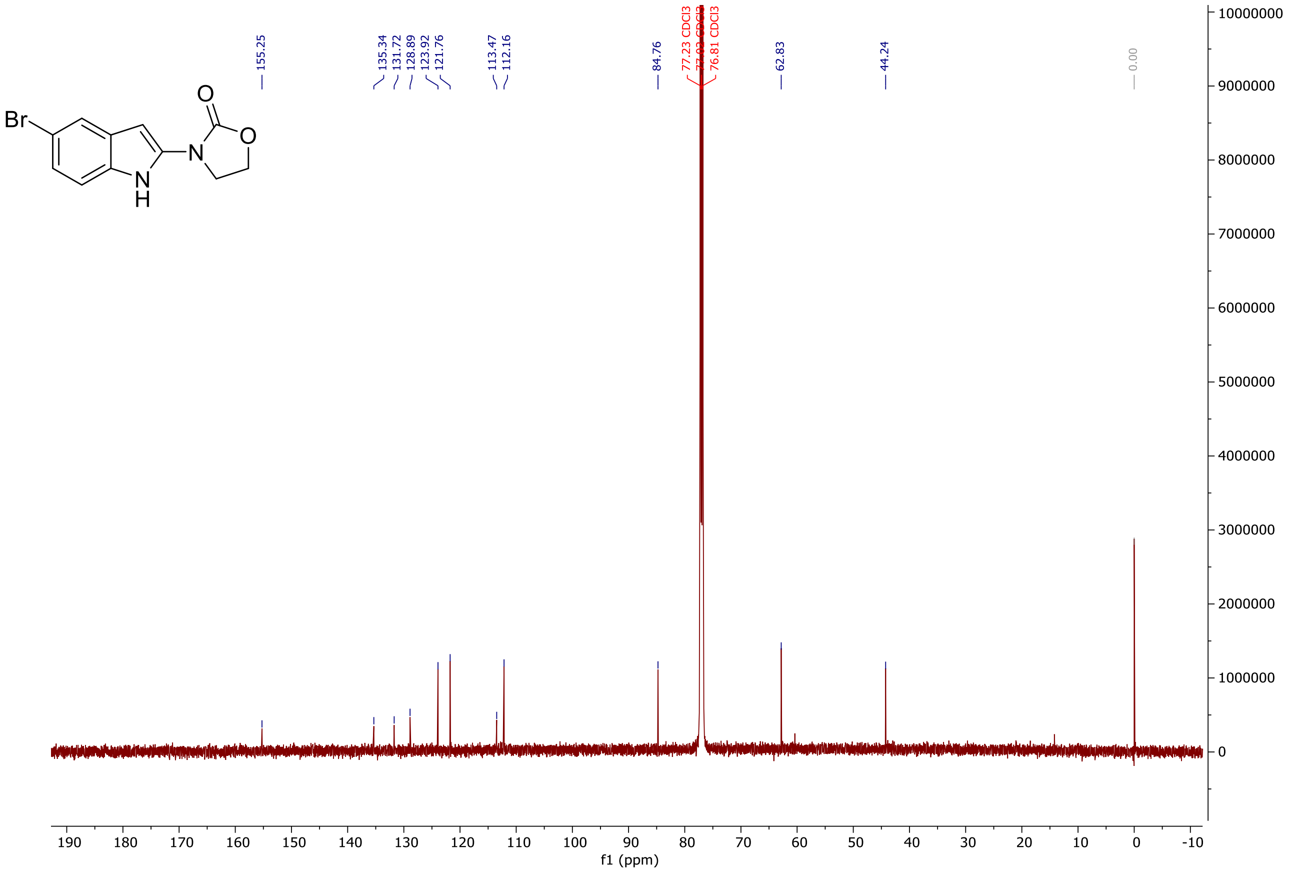

COSY (CD_3CN)

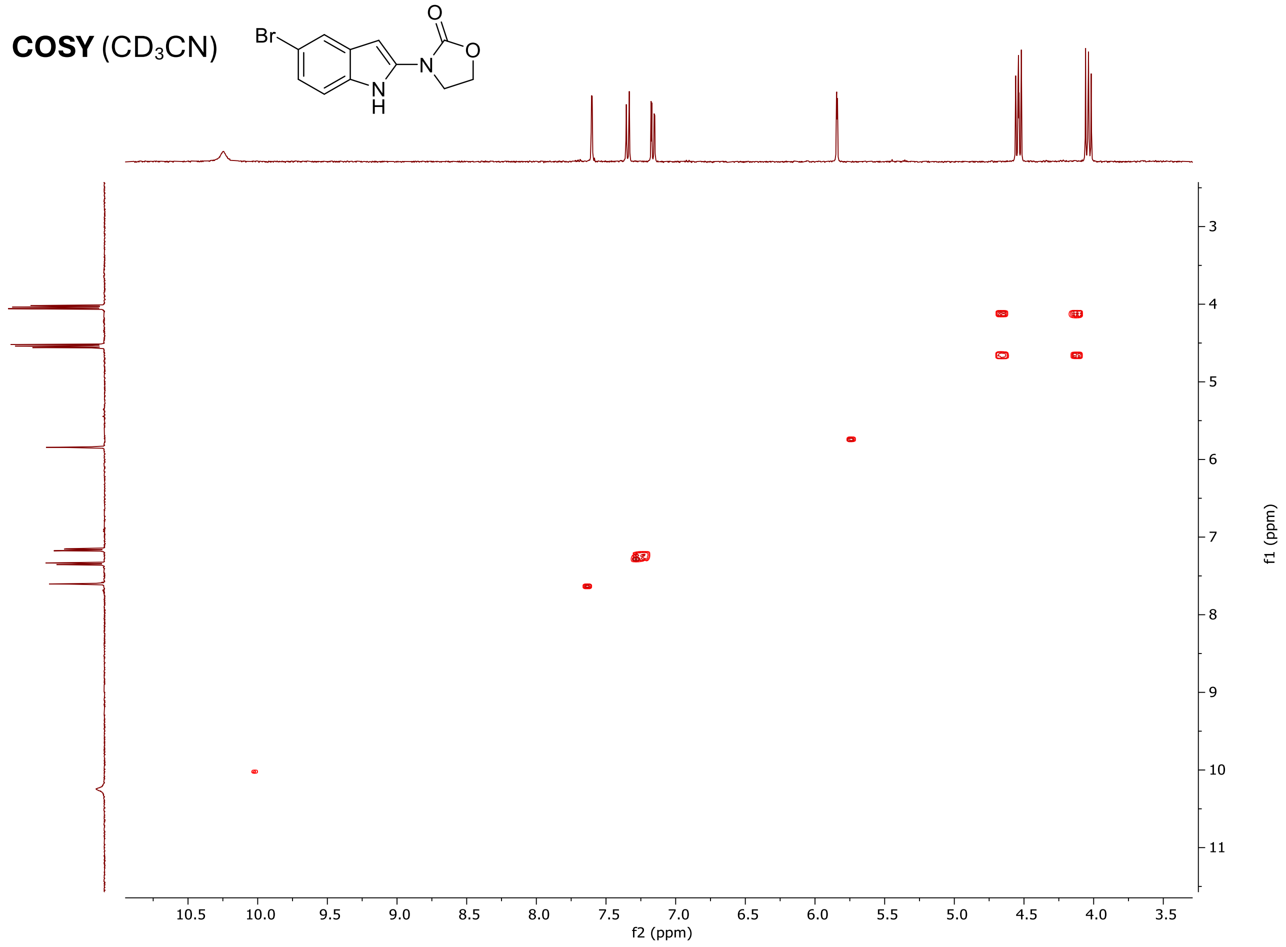

HSQC (CDCl$_3$)

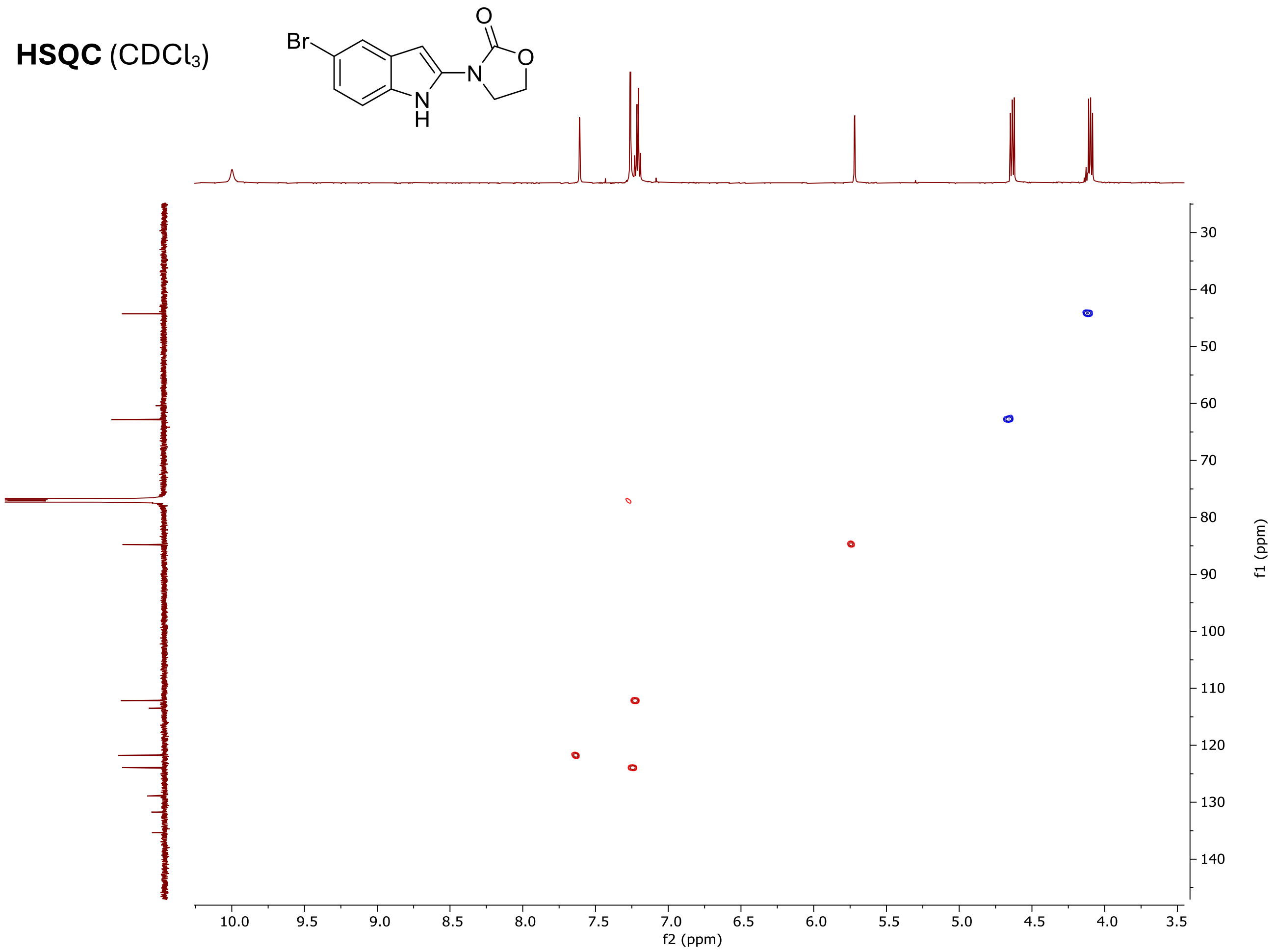

HMBC (CDCl$_3$)

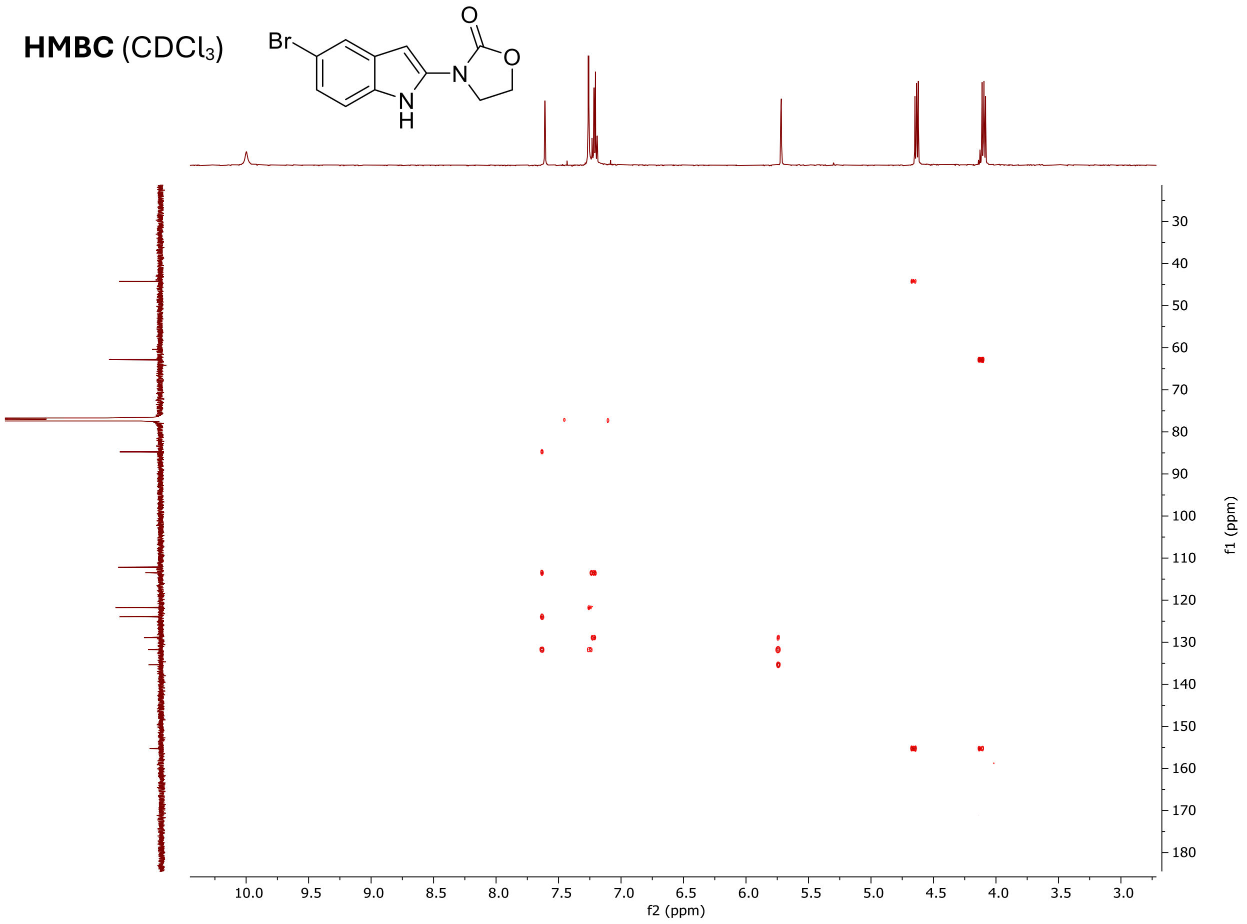

3-(1H-indol-2-yl)oxazolidin-2-one (5j) **^{1}H-NMR** (400 MHz; $CDCl_3$)

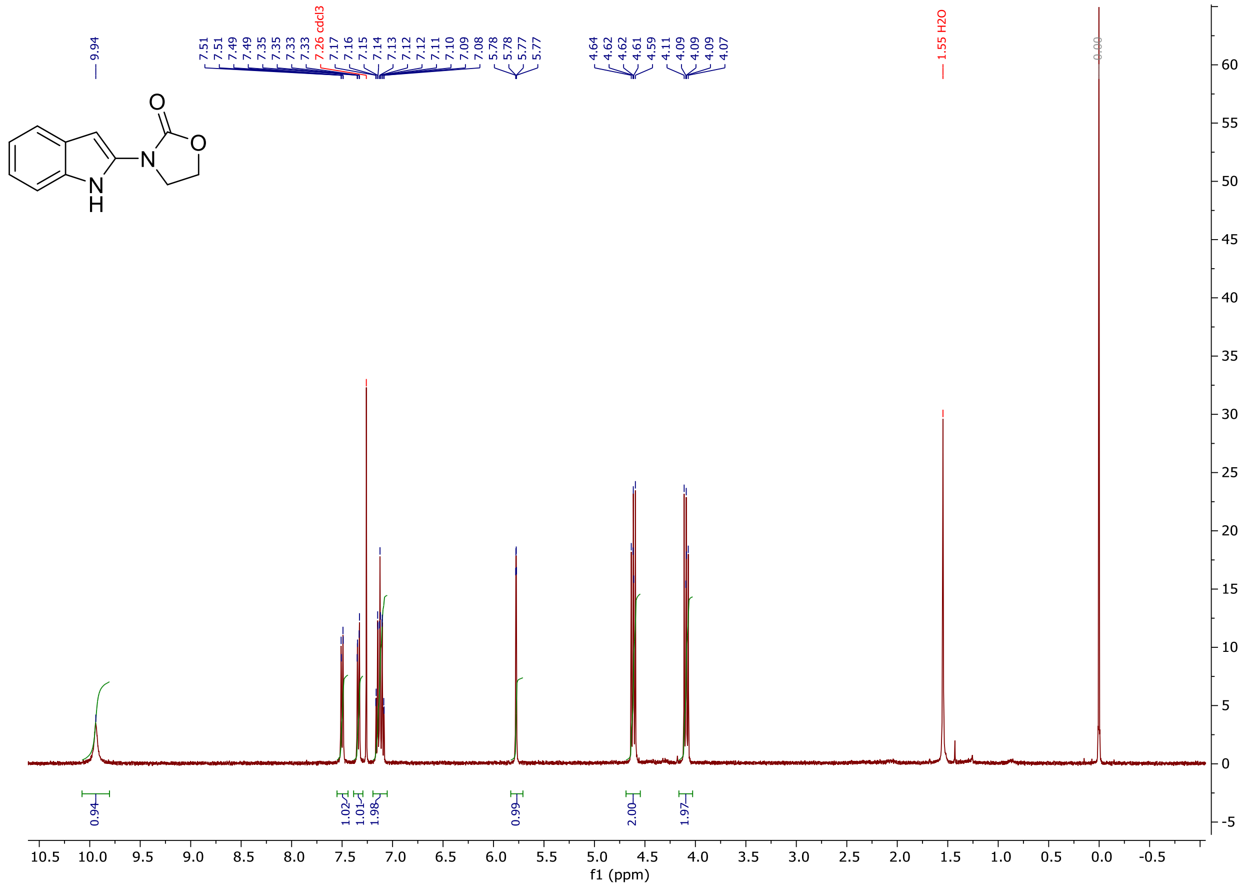

^{13}C-NMR (101 MHz; $CDCl_3$)

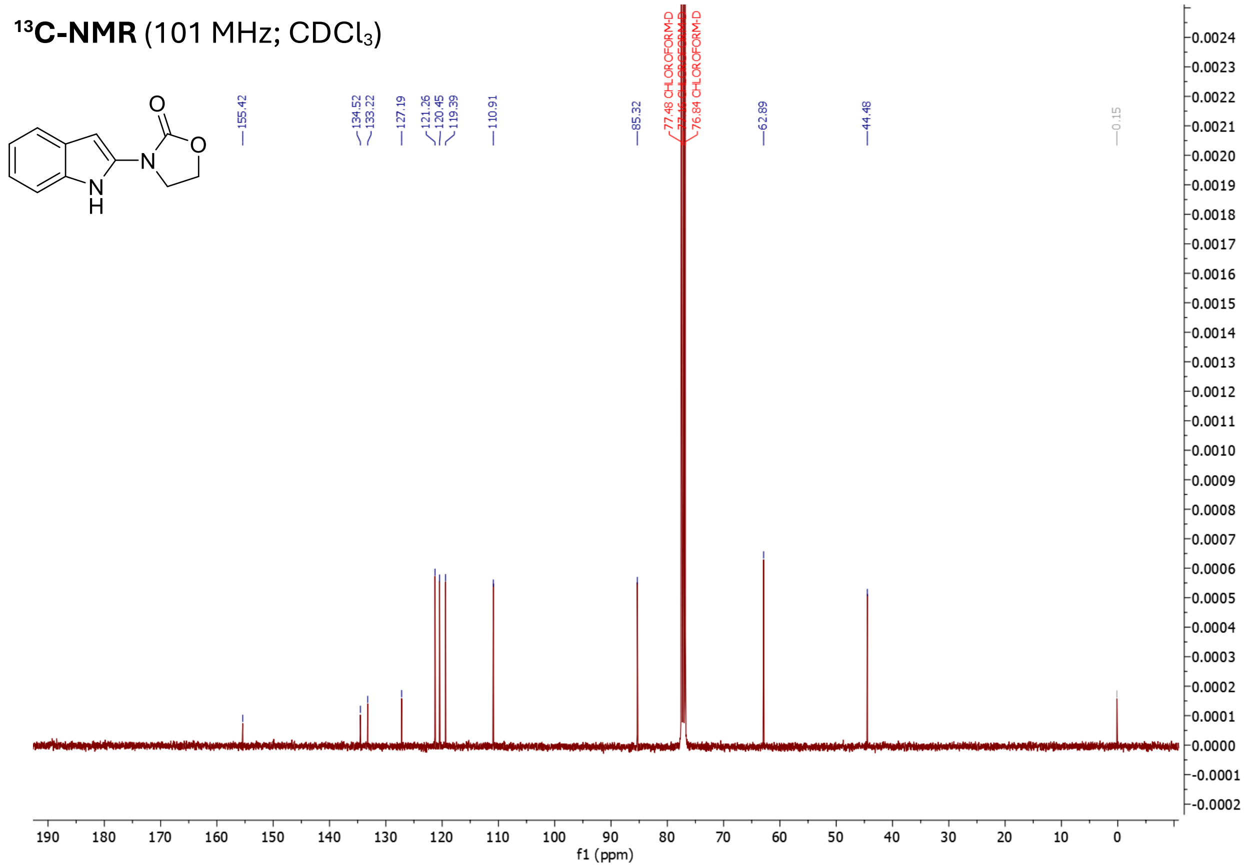

COSY ($CDCl_3$)

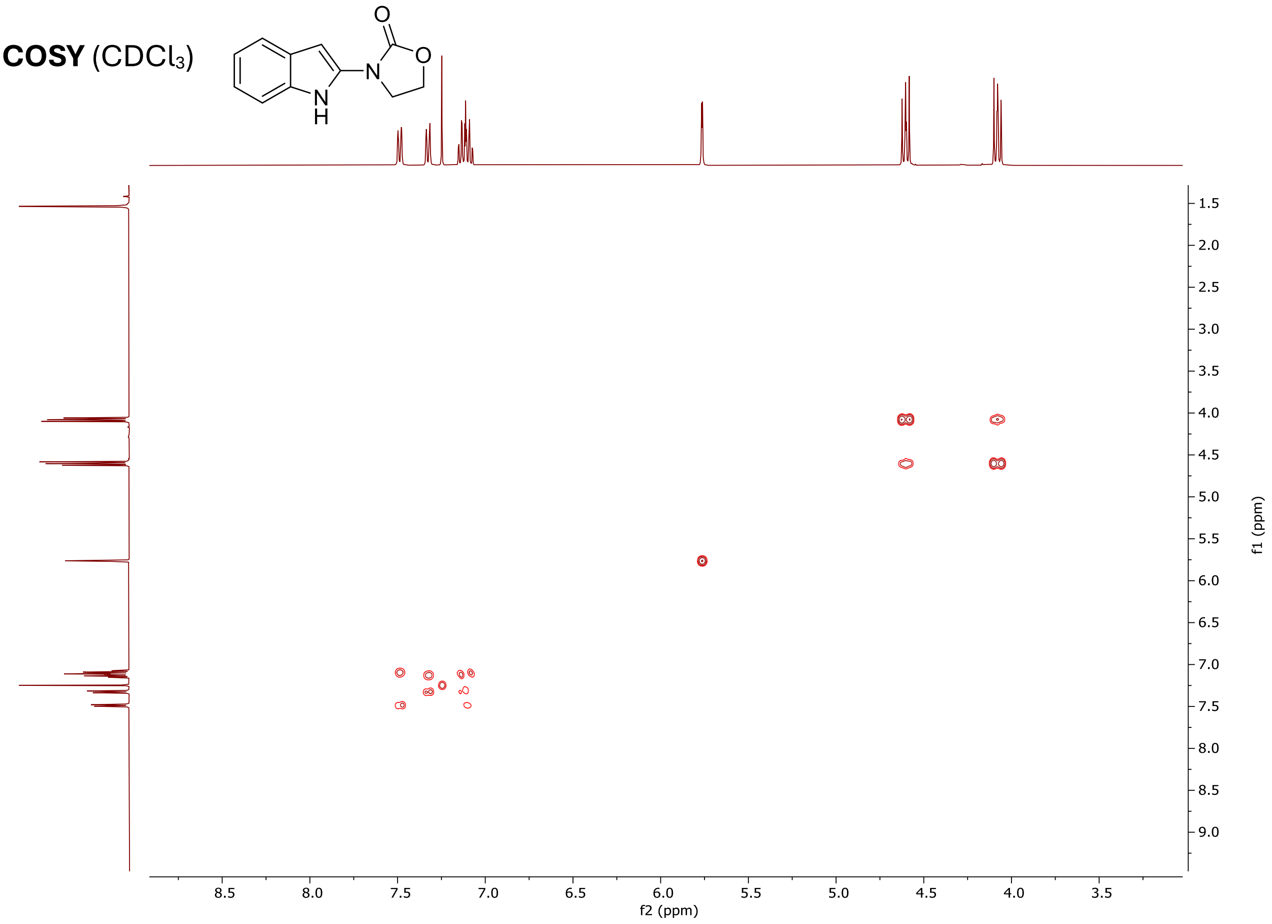

HSQC ($CDCl_3$)

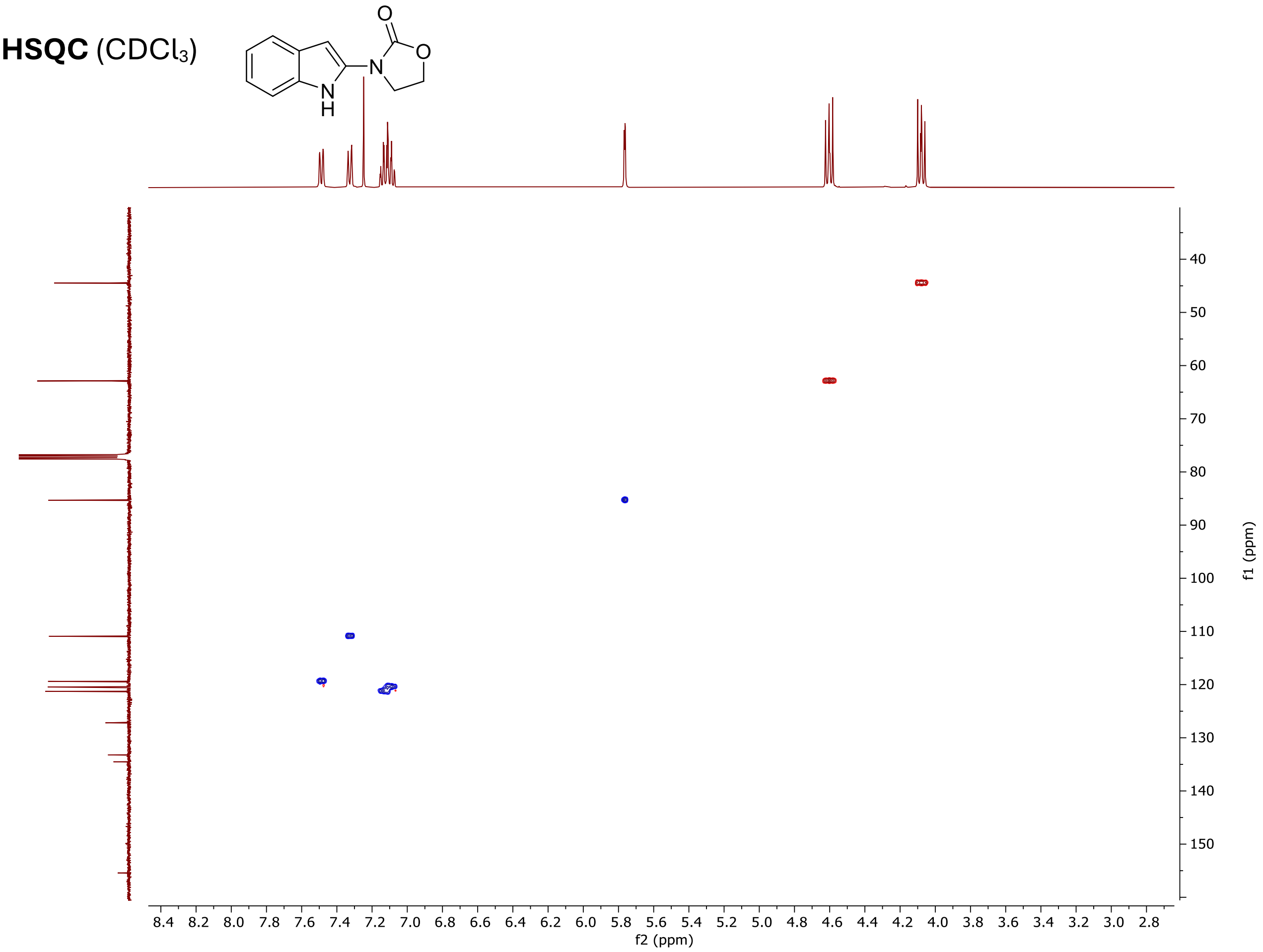

HMBC ($CDCl_3$)

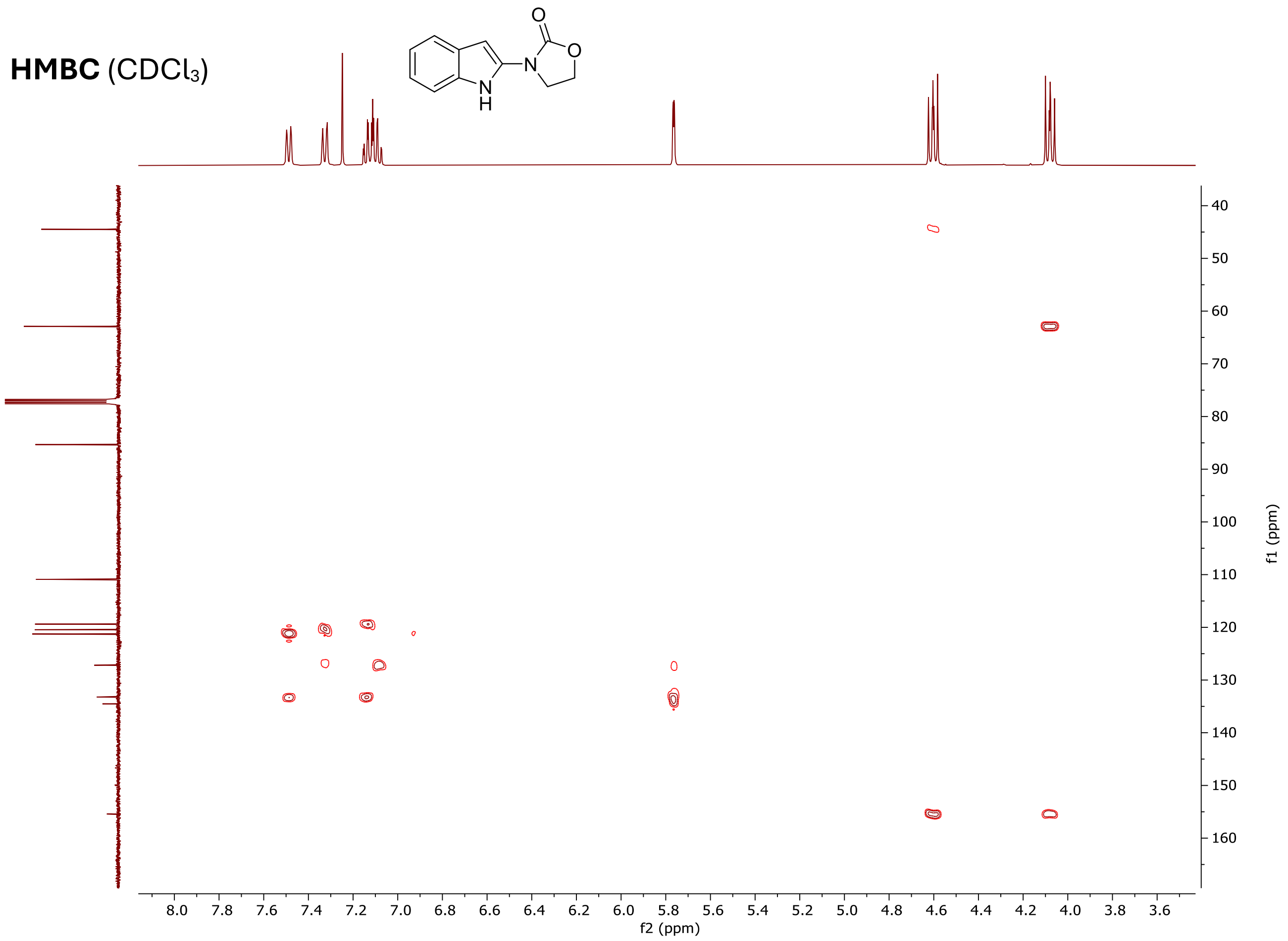

3-(1-methyl-1H-pyrrol-2-yl)oxazolidin-2-one (5k)

^{1}H-NMR (400 MHz; $CDCl_3$)

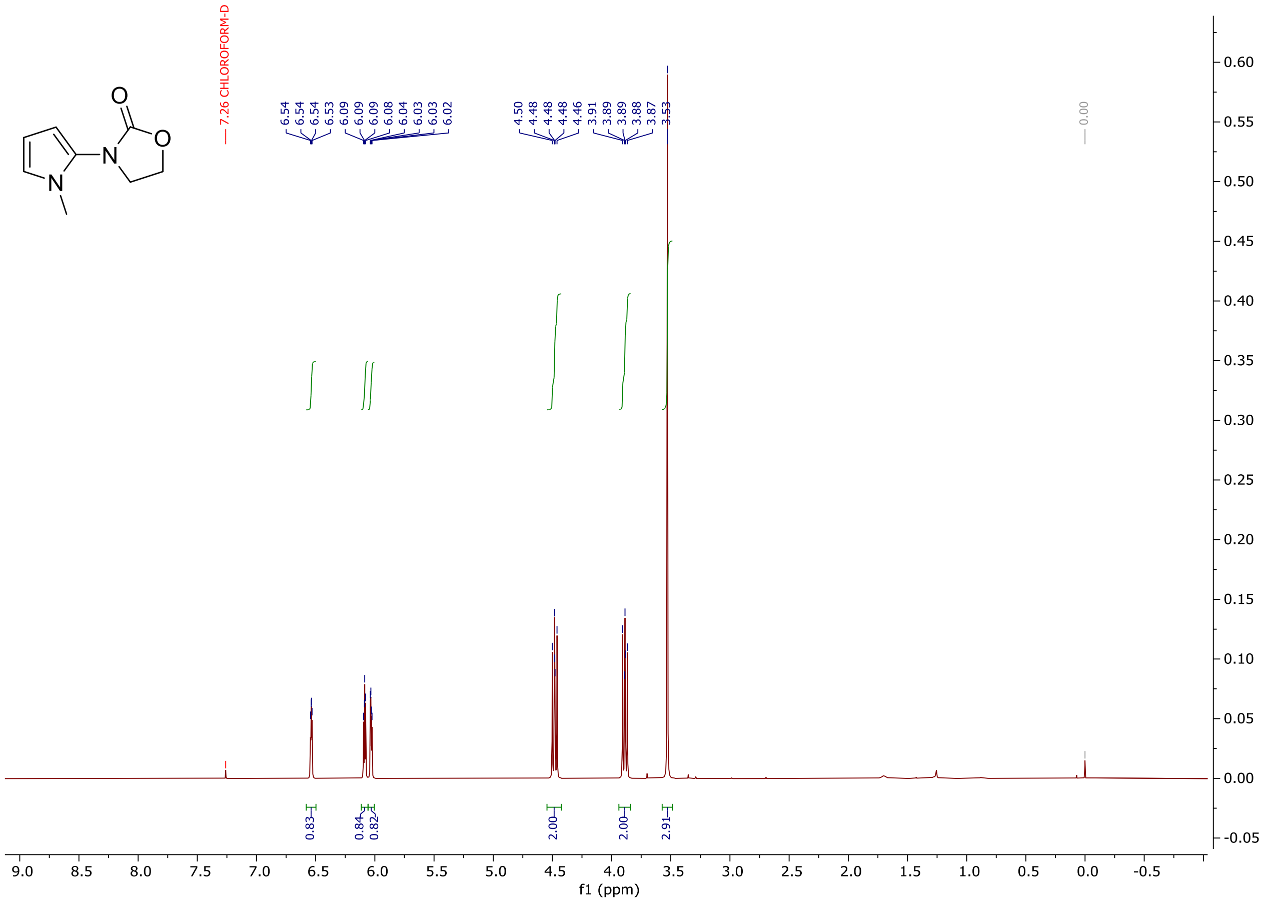

^{13}C-NMR (101 MHz; CDCl₃)

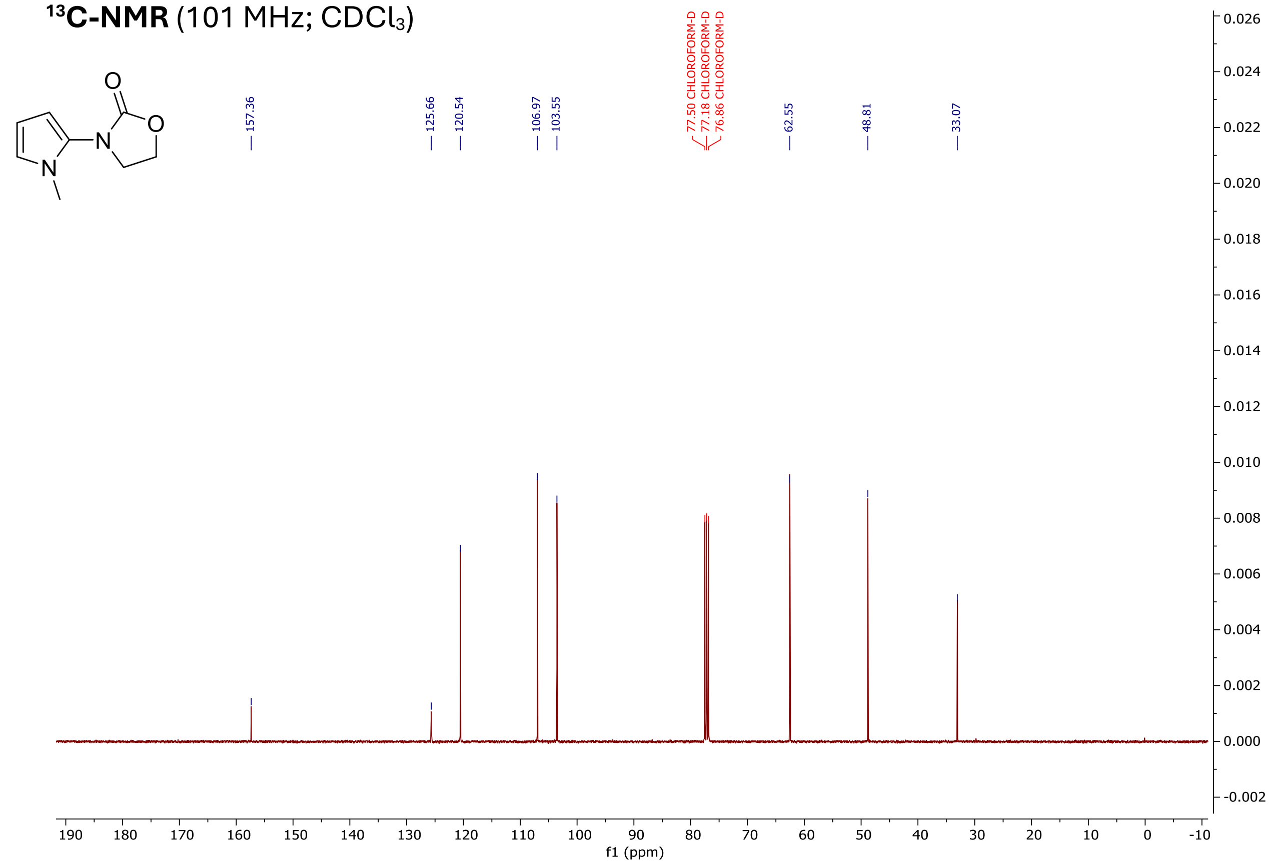

COSY (CDCl₃)

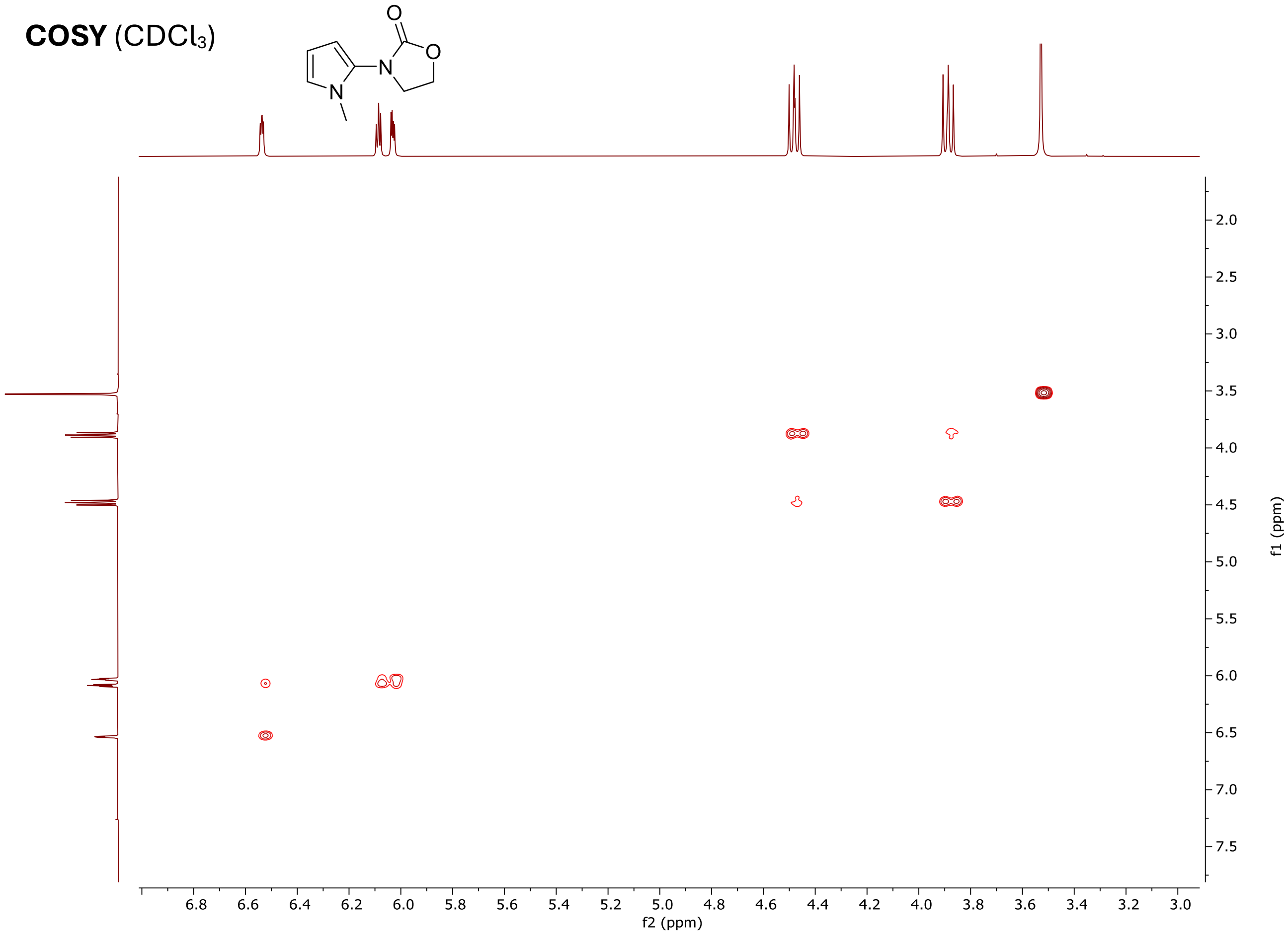

HSQC (CDCl$_3$)

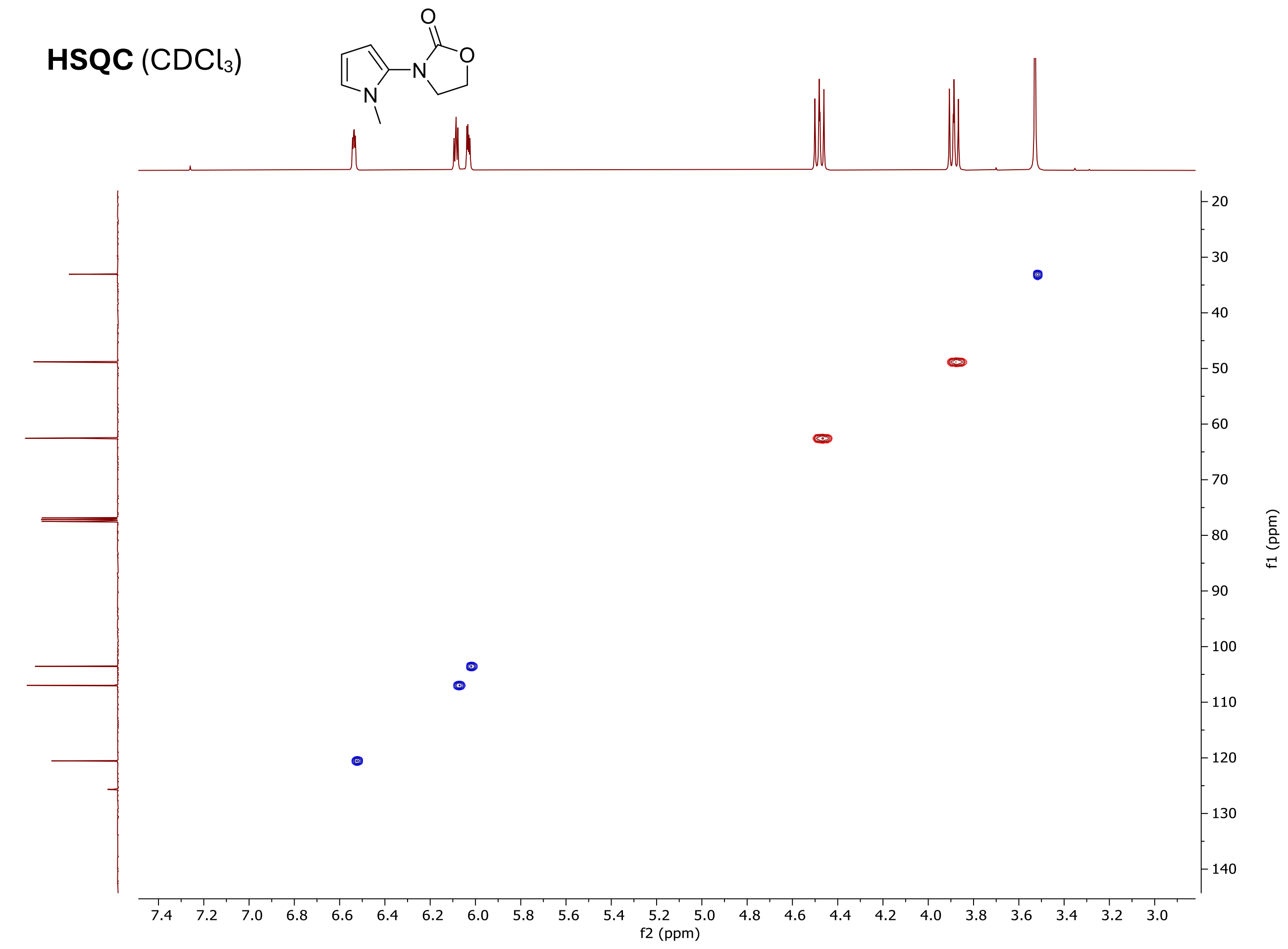

HMBC (CDCl$_3$)

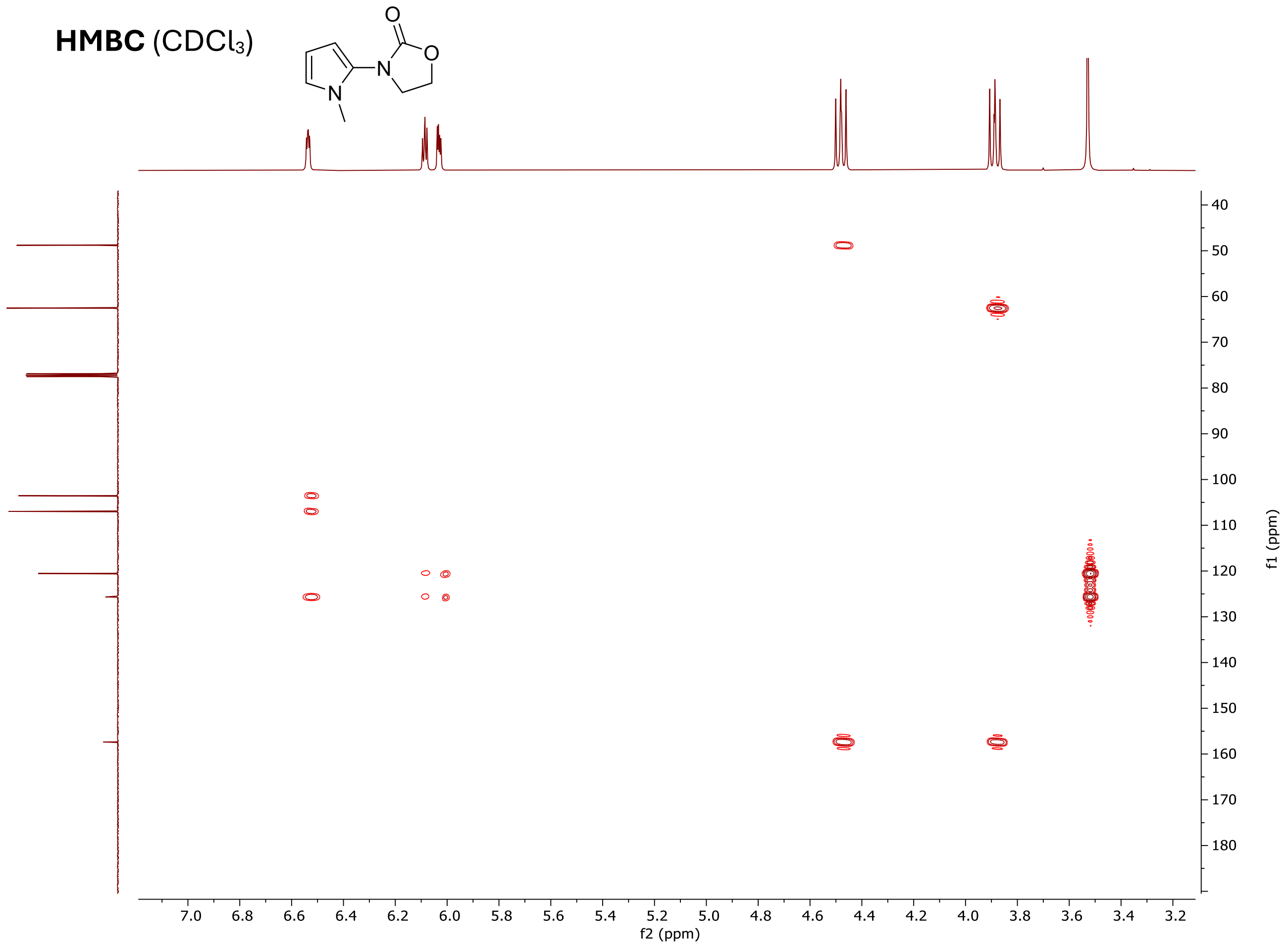

3-(1-phenyl-1H-pyrrol-2-yl)oxazolidin-2-one (5l) **^{1}H-NMR** (400 MHz; $CDCl_3$)

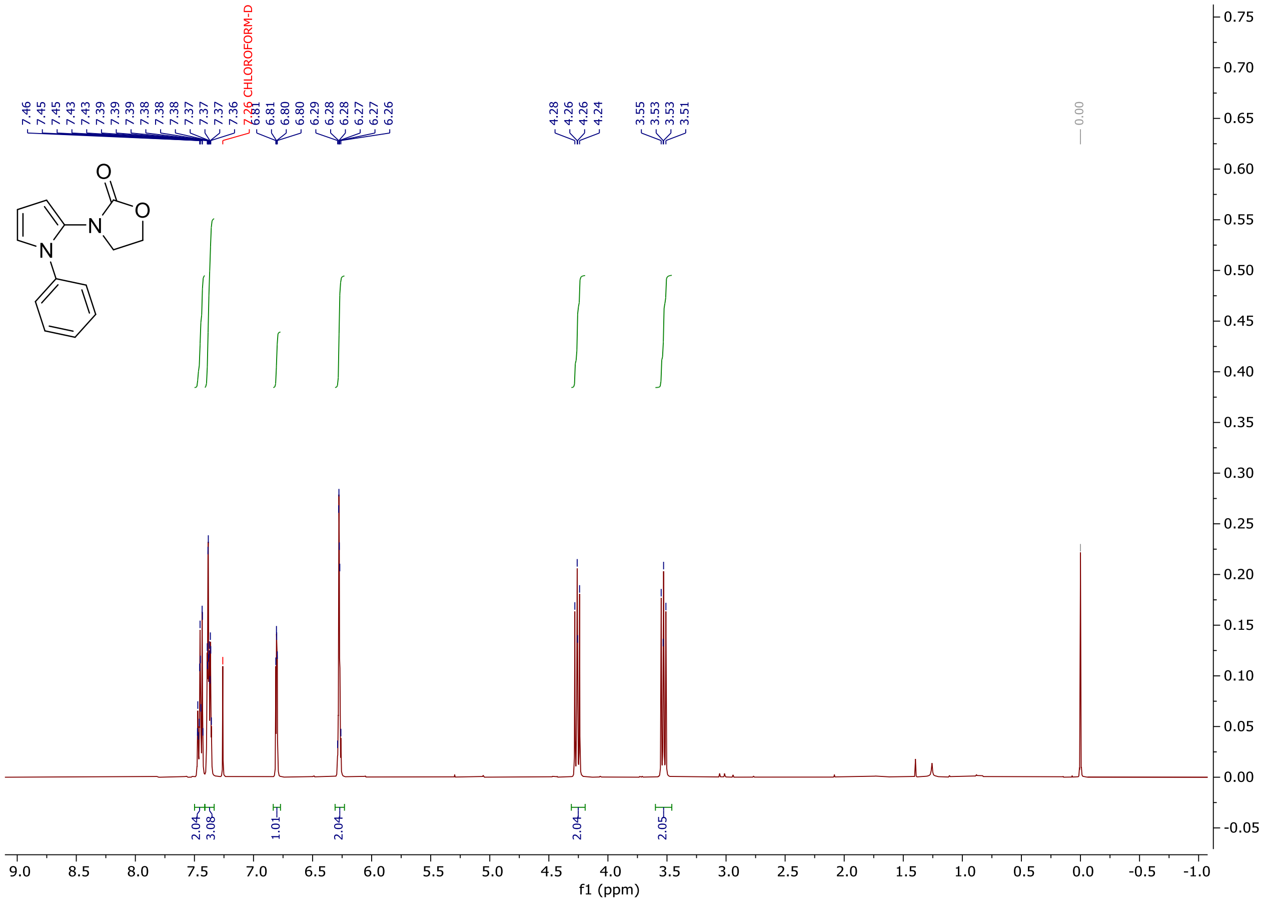

^{13}C-NMR (101 MHz; $CDCl_3$)

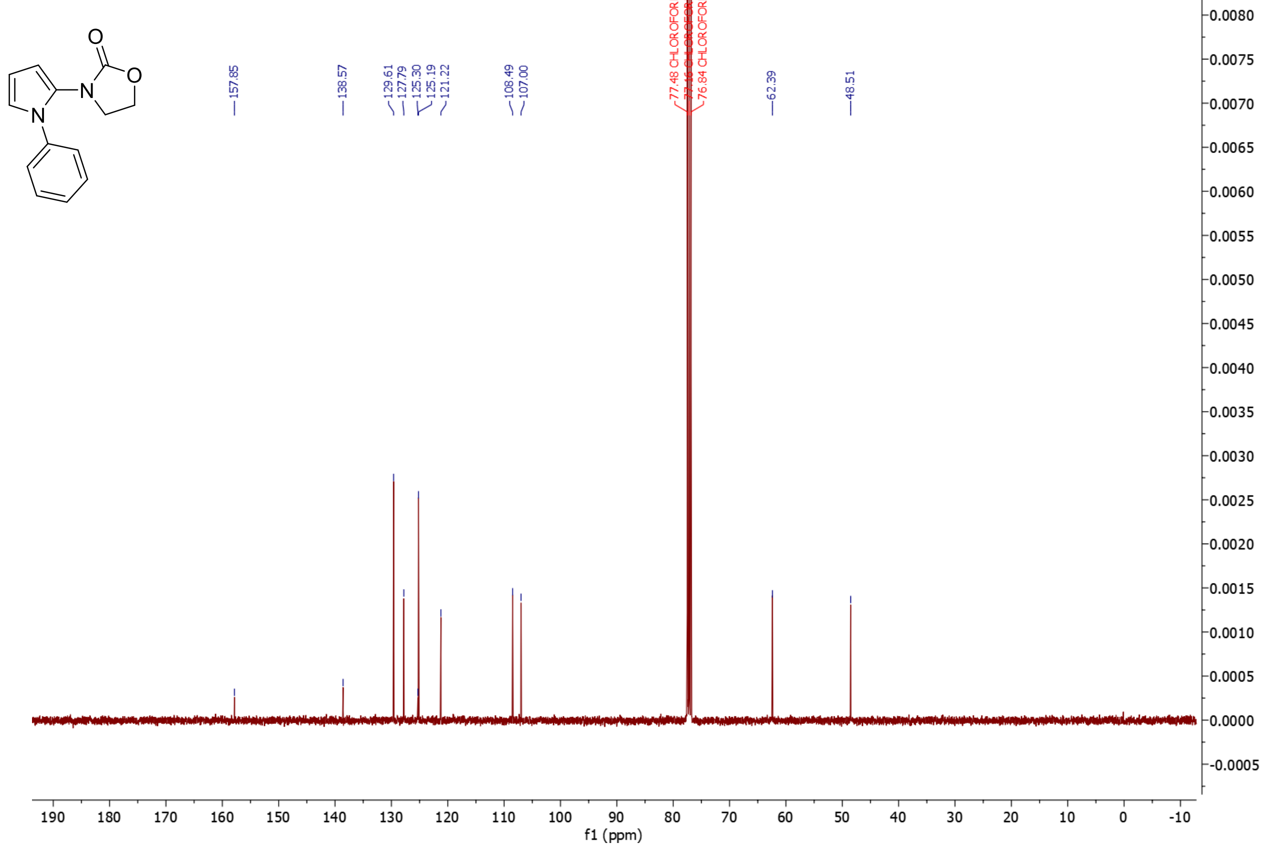

COSY ($CDCl_3$)

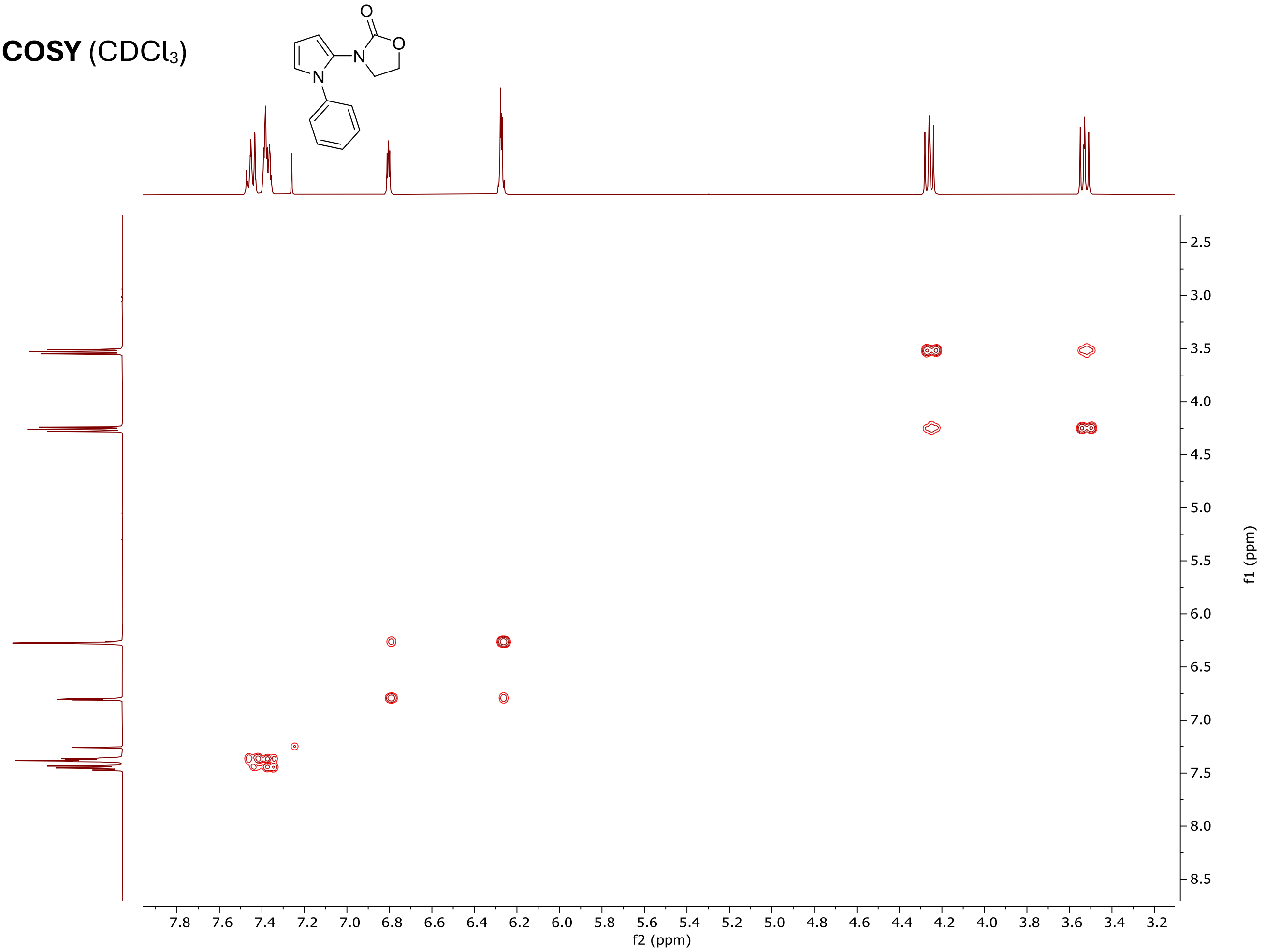

HSQC ($CDCl_3$)

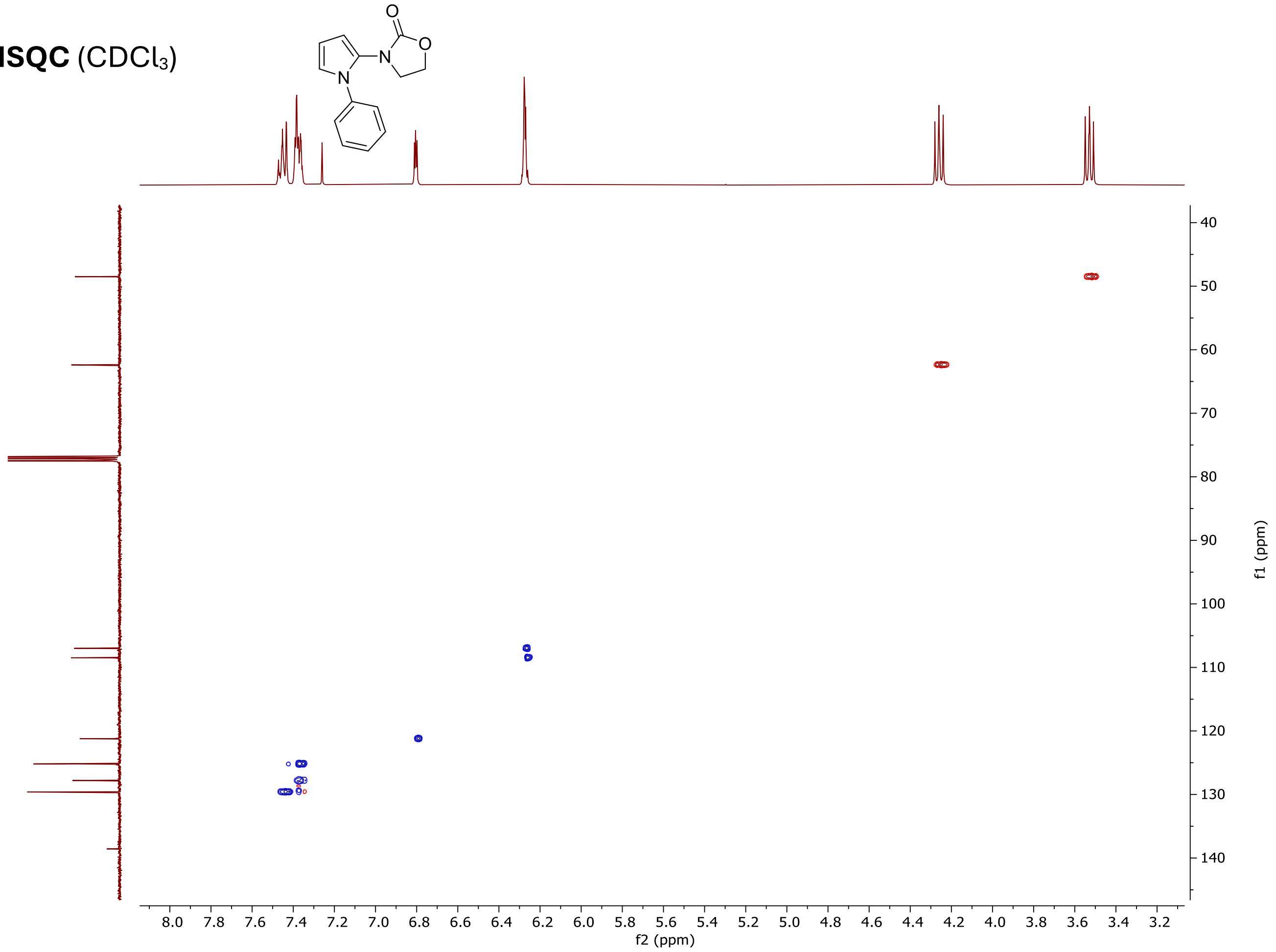

HMBC ($CDCl_3$)

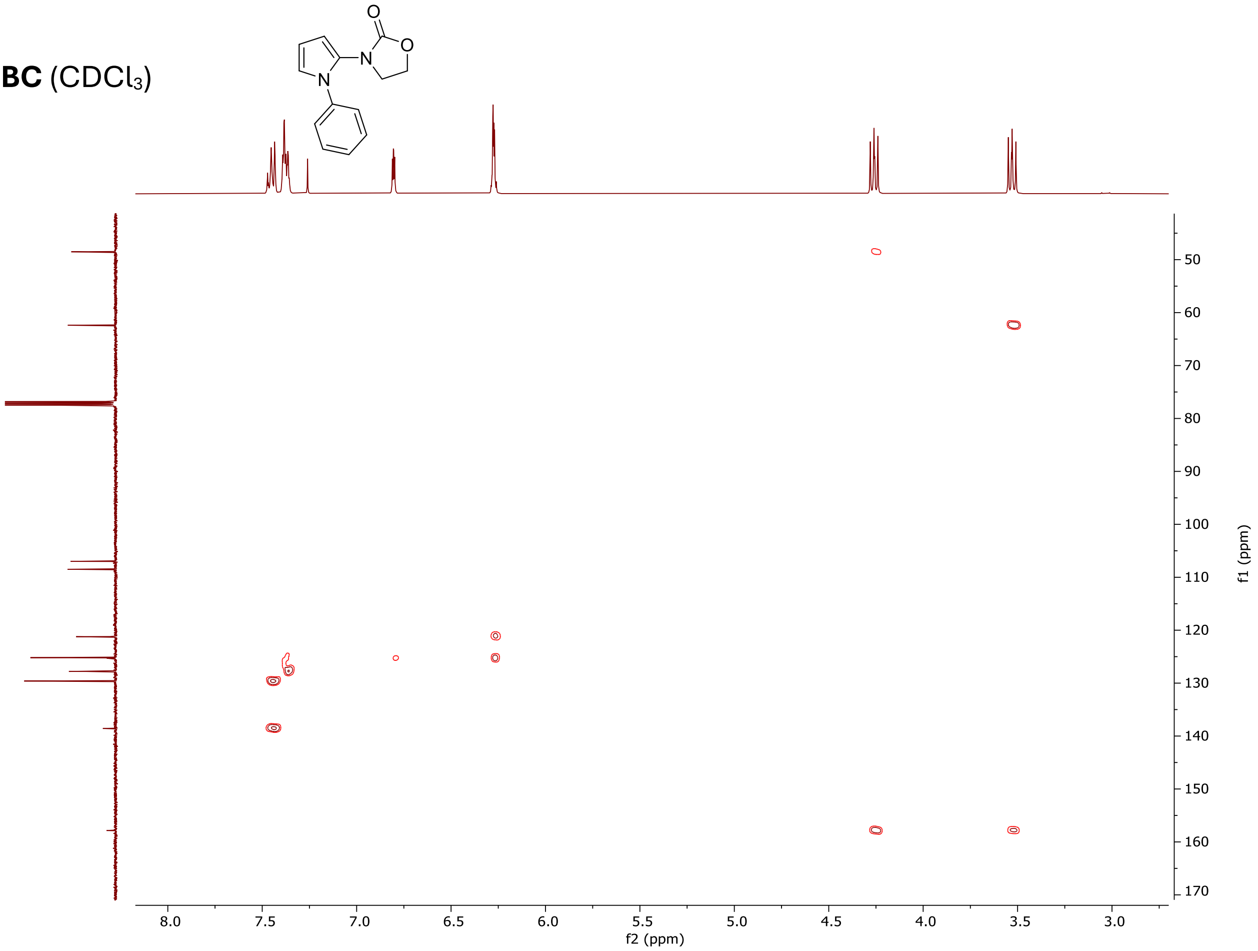

3-(benzofuran-2-yl)oxazolidin-2-one (5m)

¹H-NMR (400 MHz; $CDCl_3$)

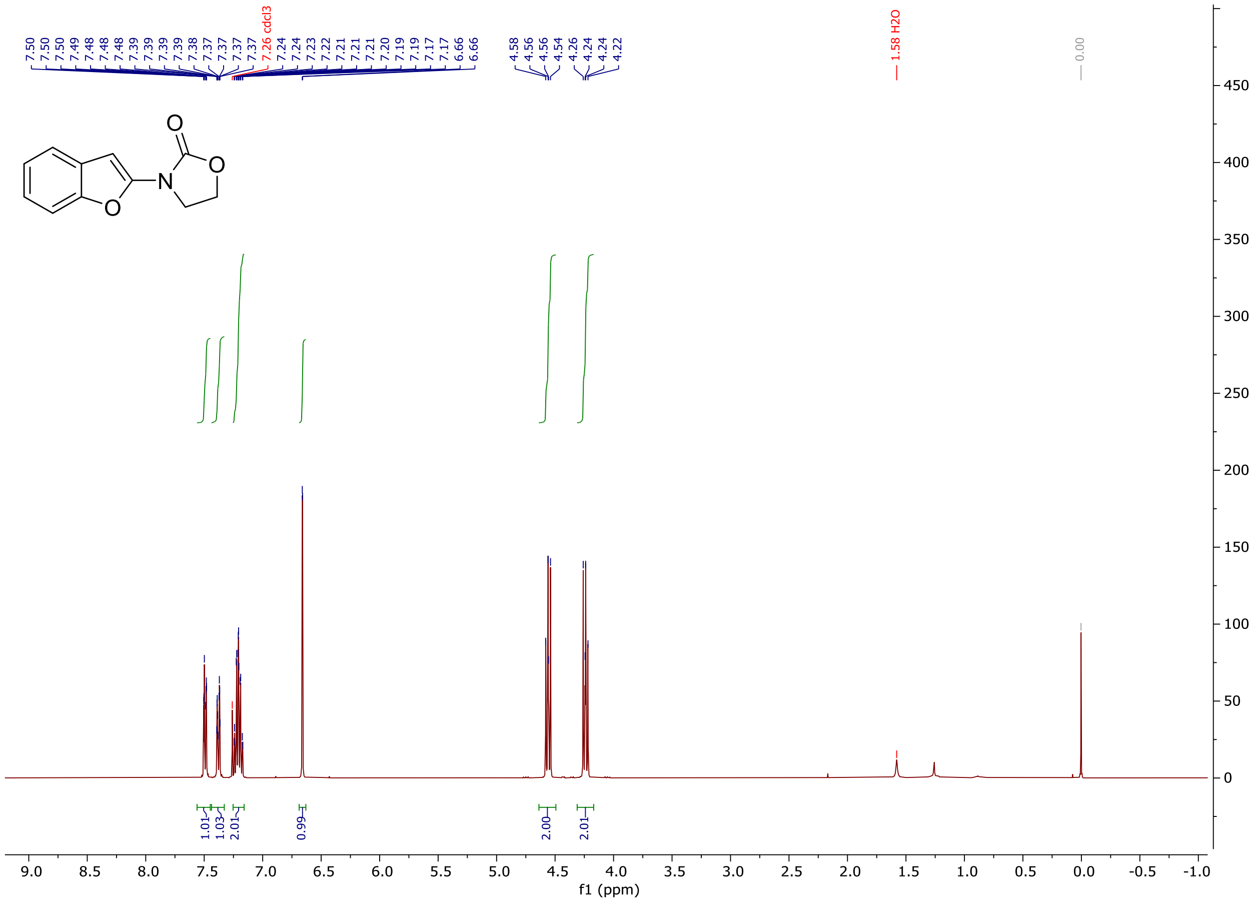

^{13}C-NMR (101 MHz; $CDCl_3$)

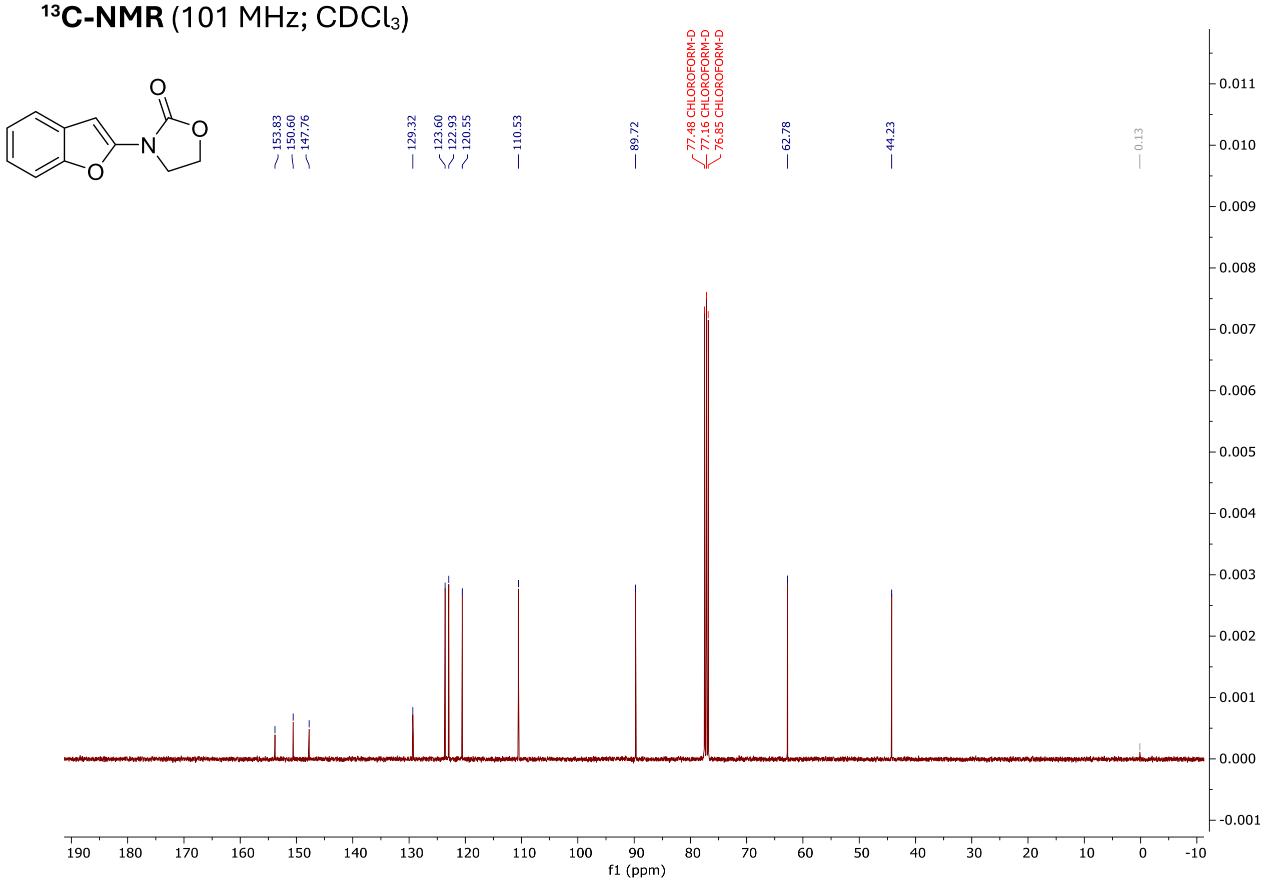

COSY ($CDCl_3$)

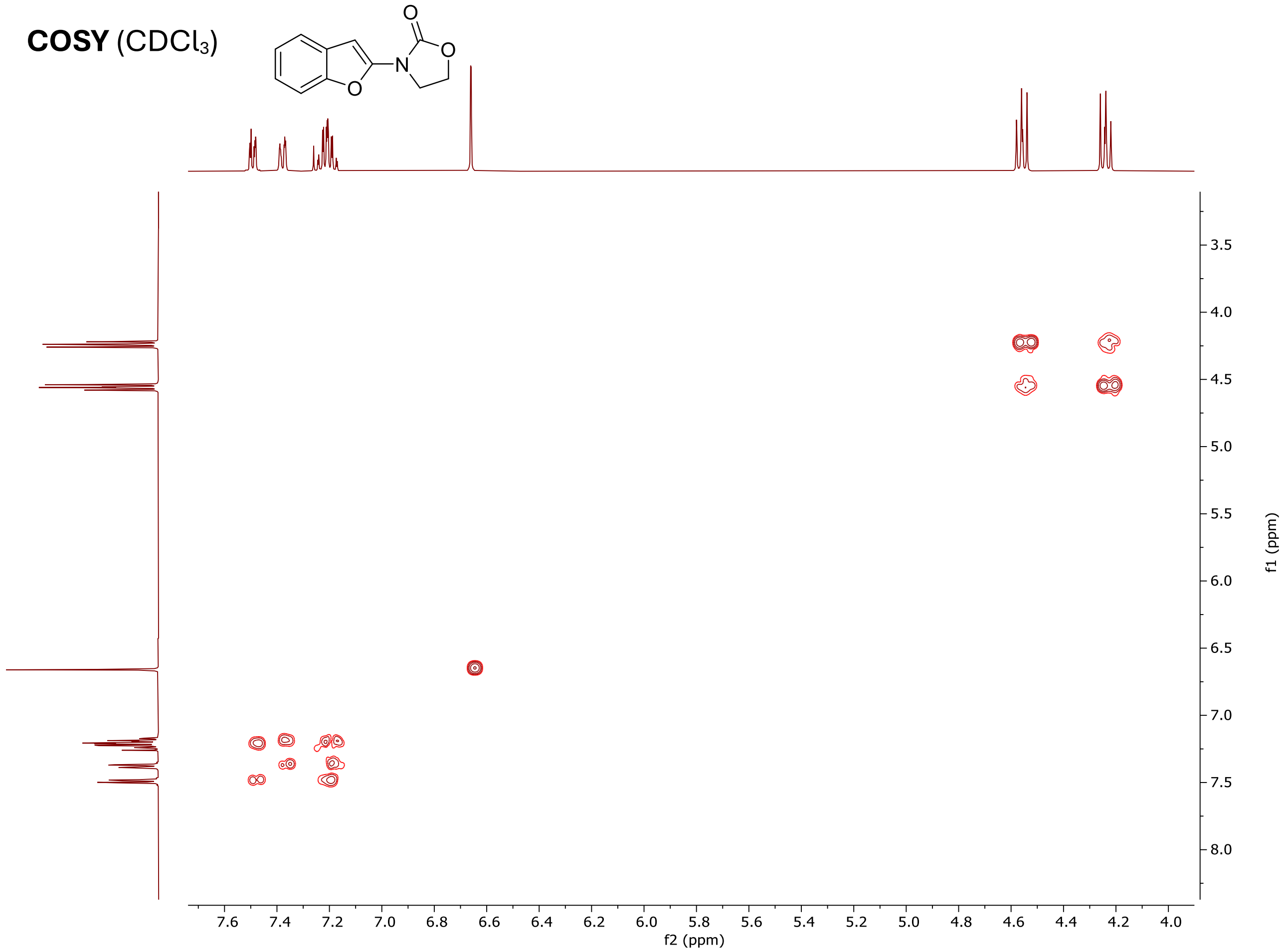

HSQC ($CDCl_3$)

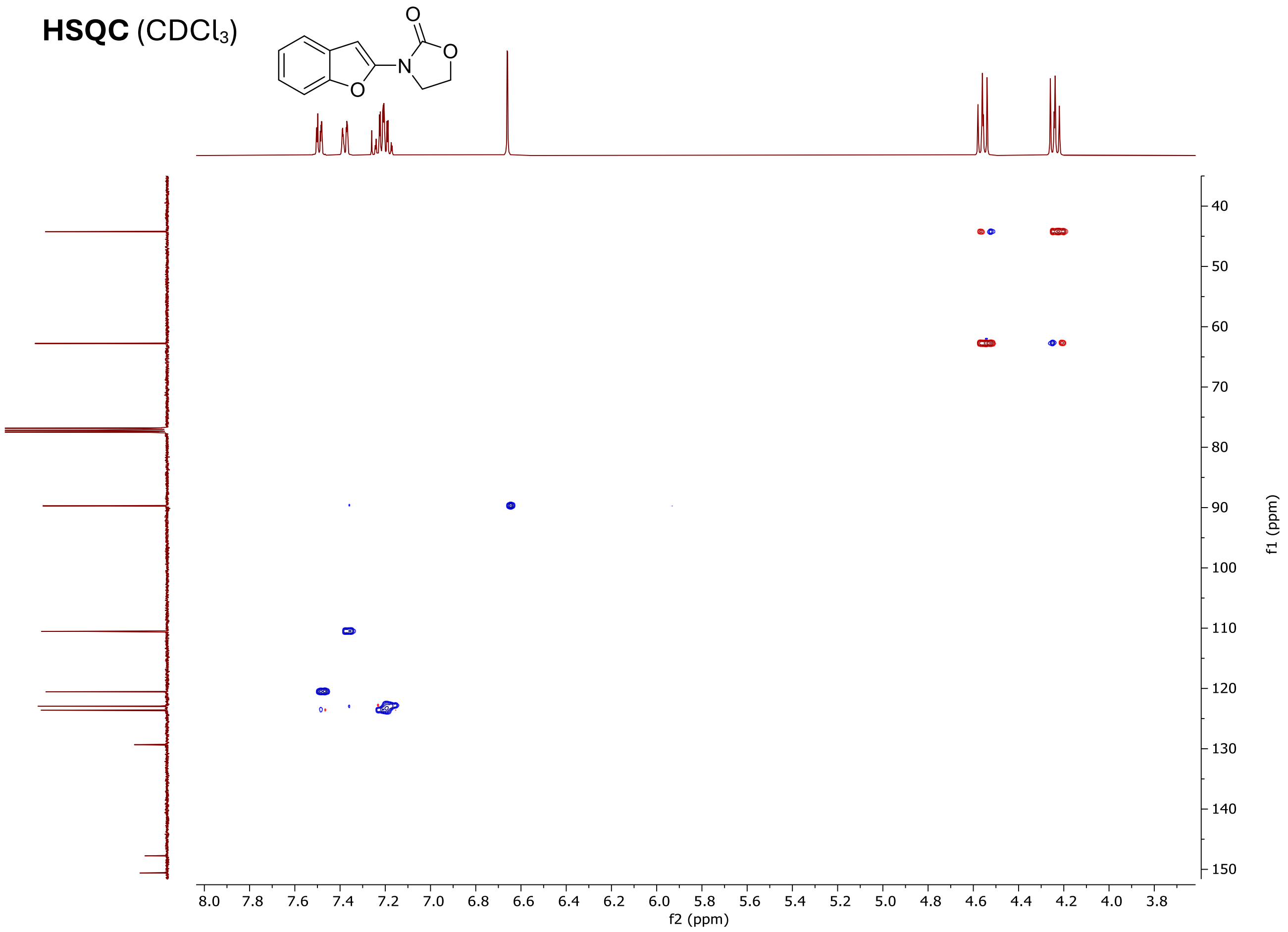

HMBC ($CDCl_3$)

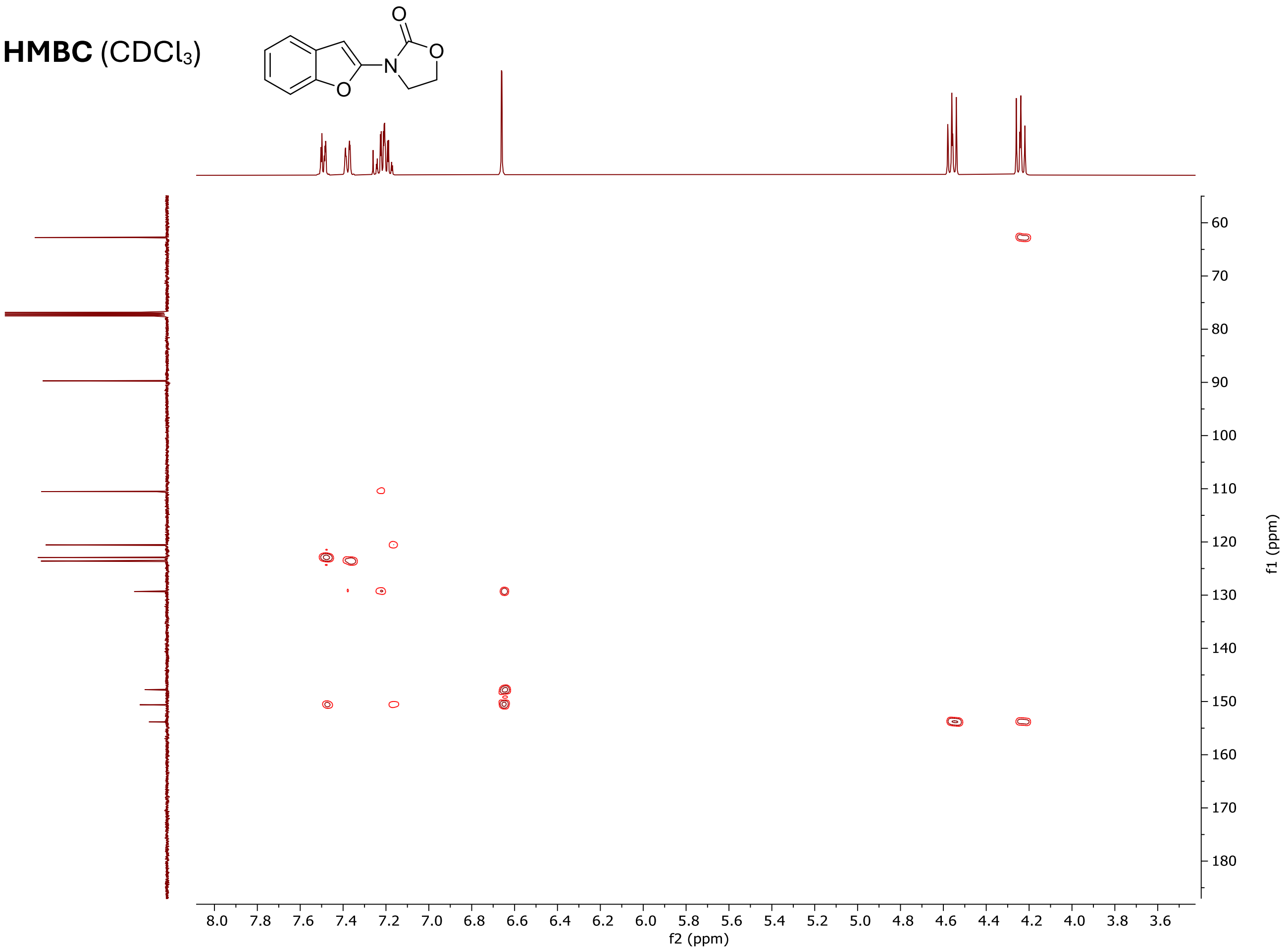

3-(anthracen-9-yl)oxazolidin-2-one (5n)

^{1}H-NMR (400 MHz; $CDCl_3$)

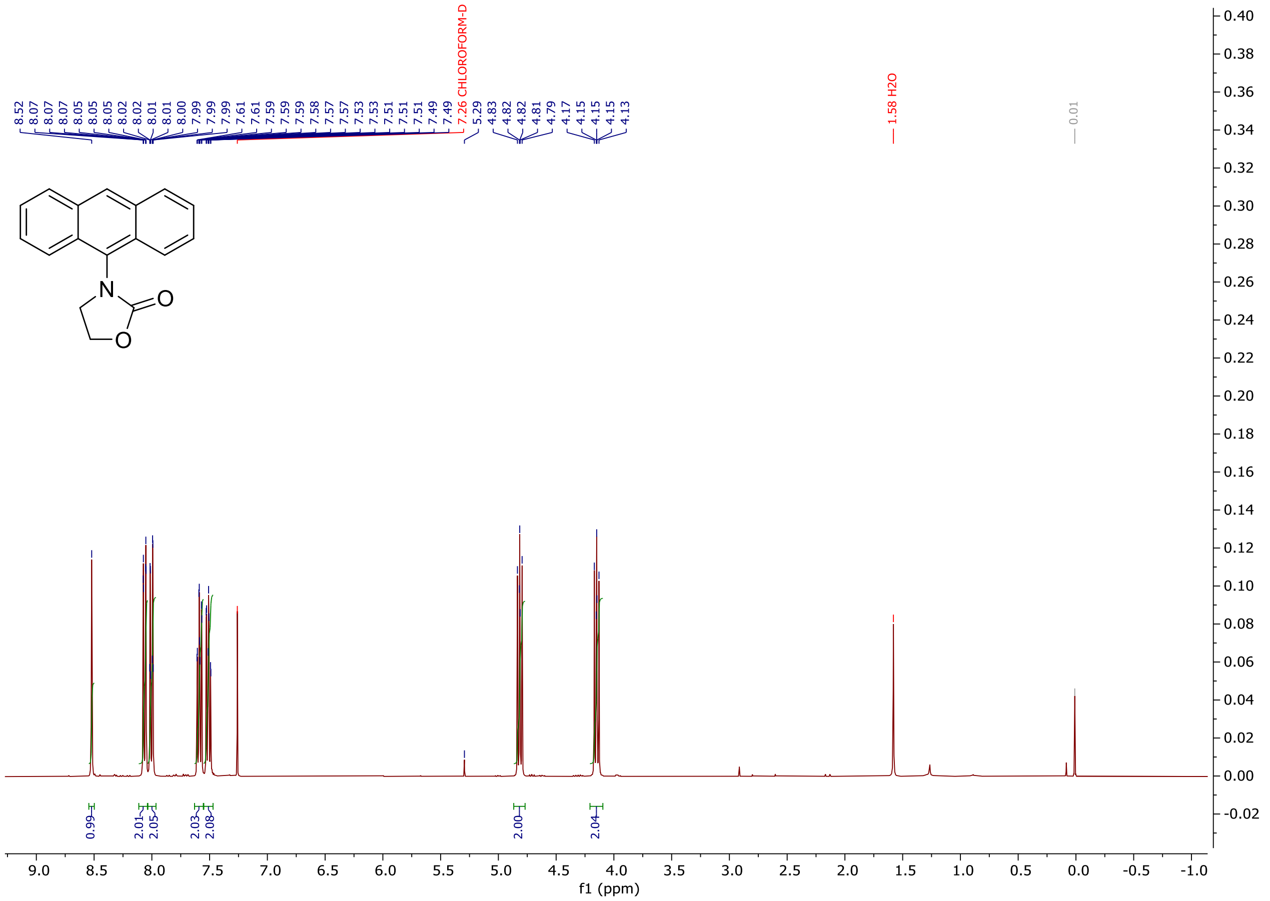

^{13}C-NMR (101 MHz; $CDCl_3$)

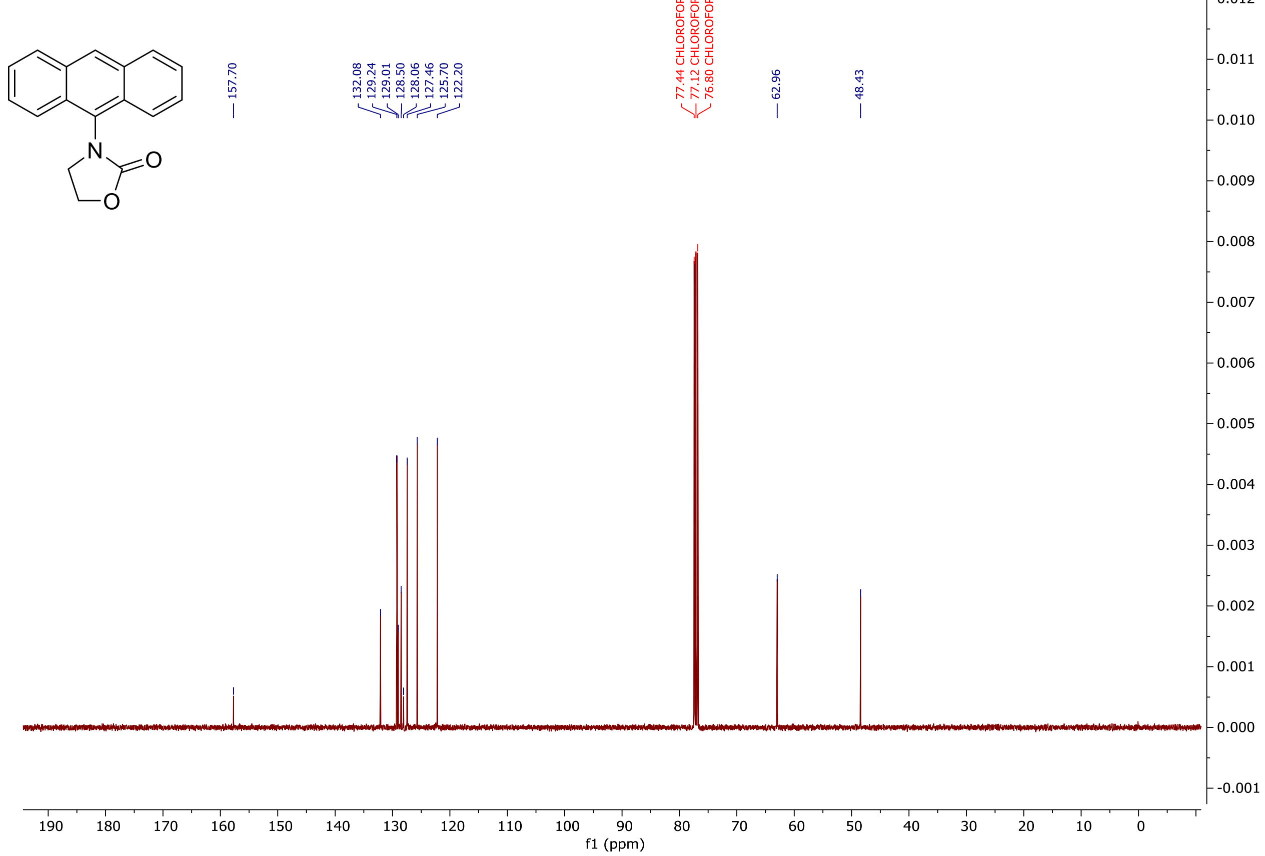

COSY (CDCl$_3$)

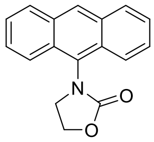

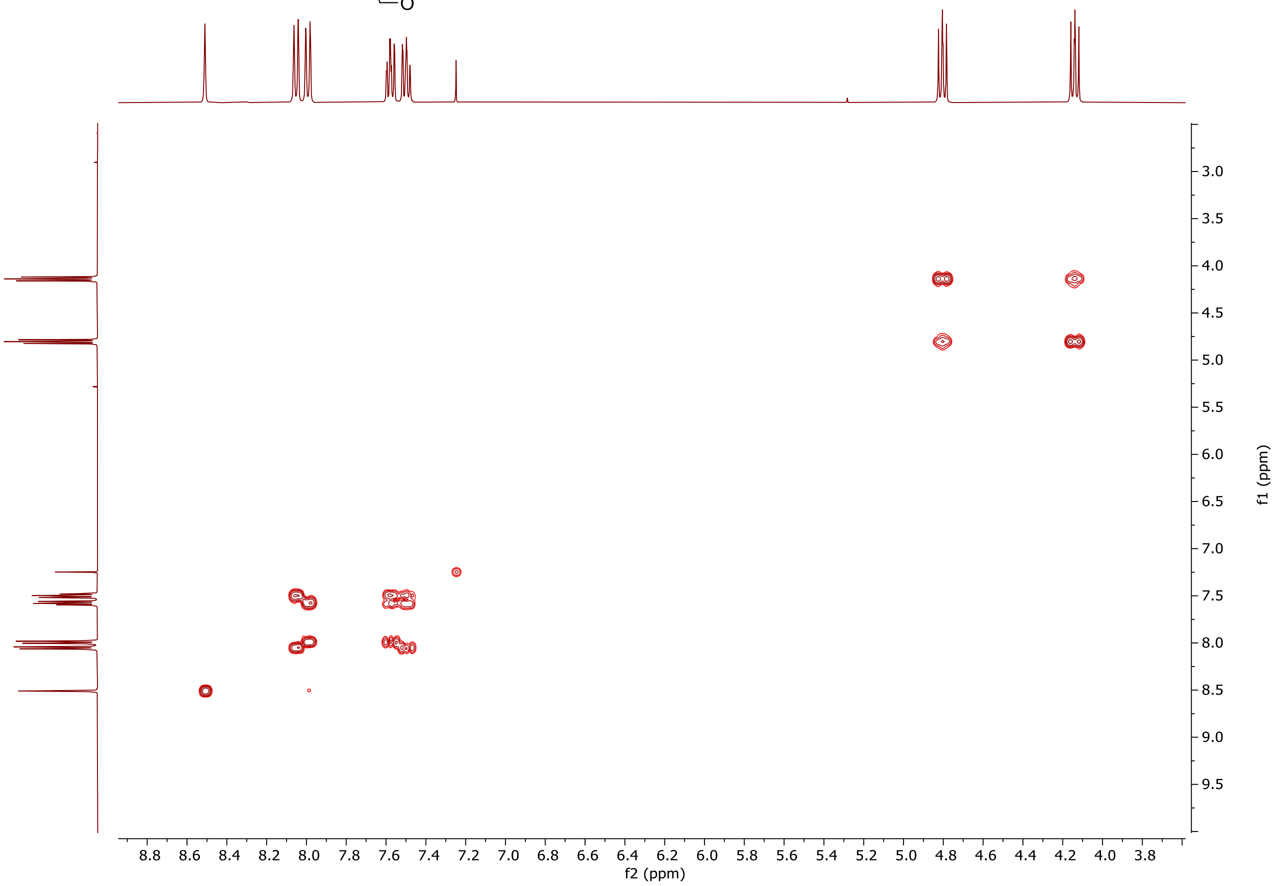

HSQC (CDCl$_3$)

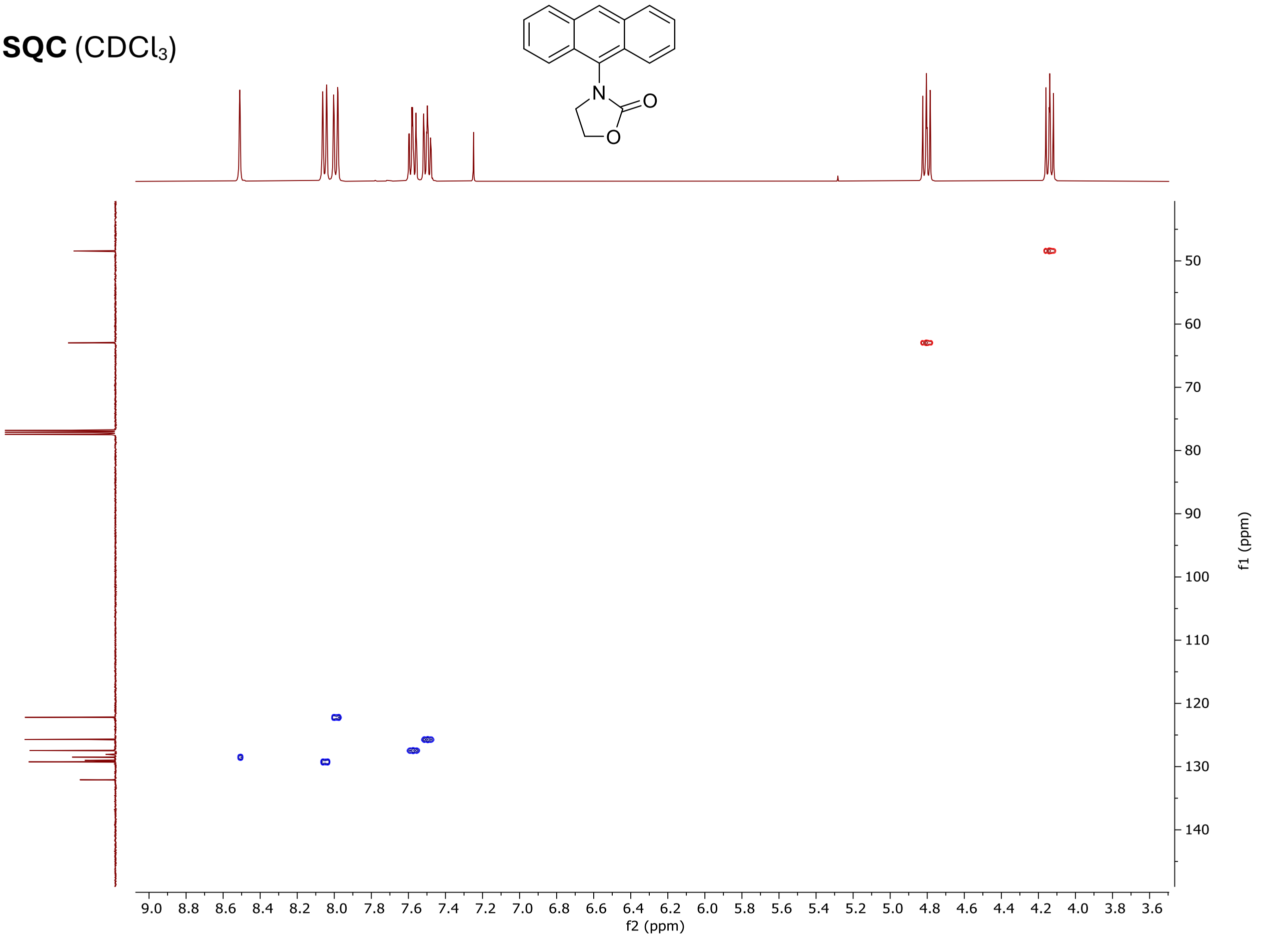

HMBC ($CDCl_3$)

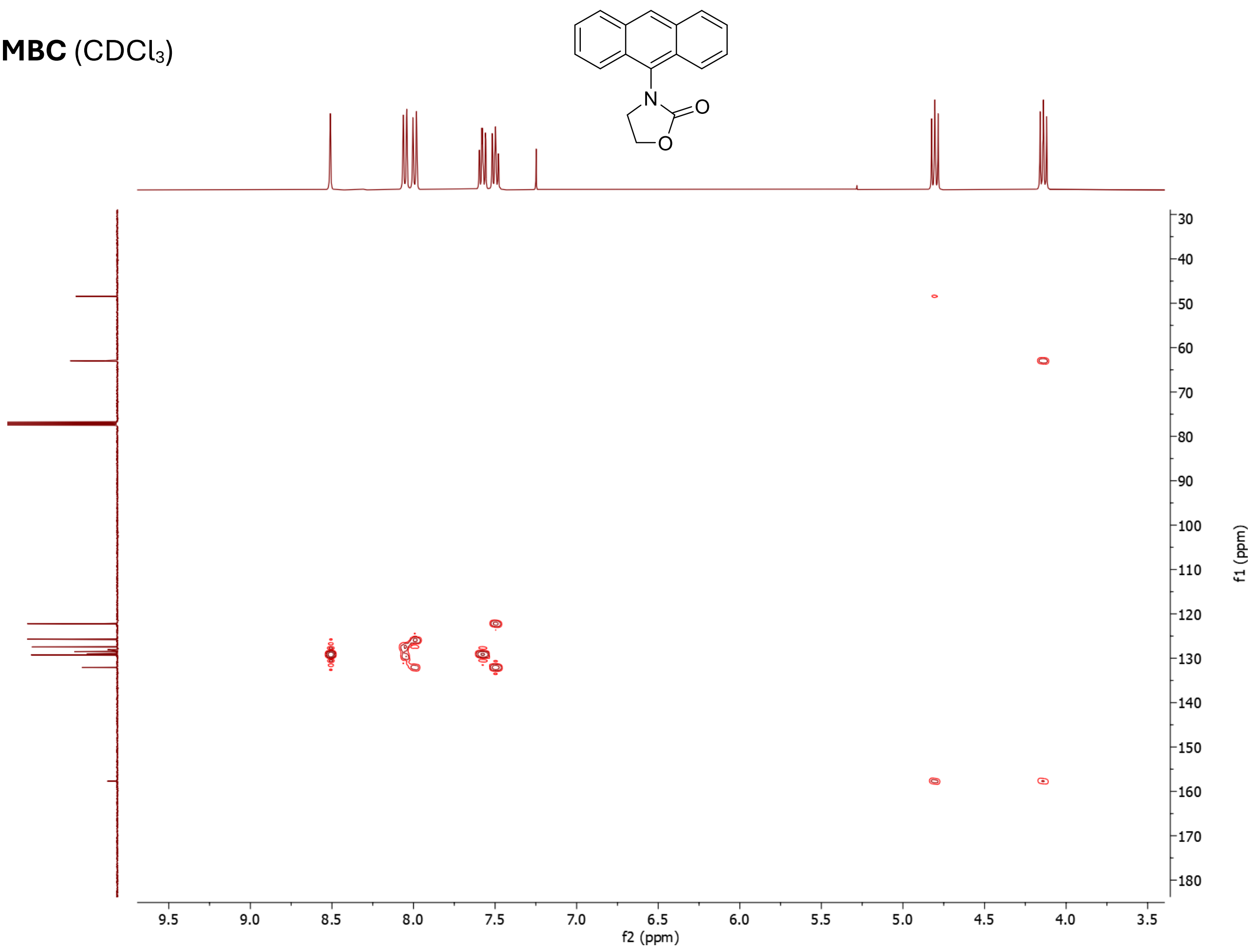

3-(2,2-diphenylvinyl)oxazolidin-2-one (5o)

^{1}H-NMR (400 MHz; $CDCl_3$)

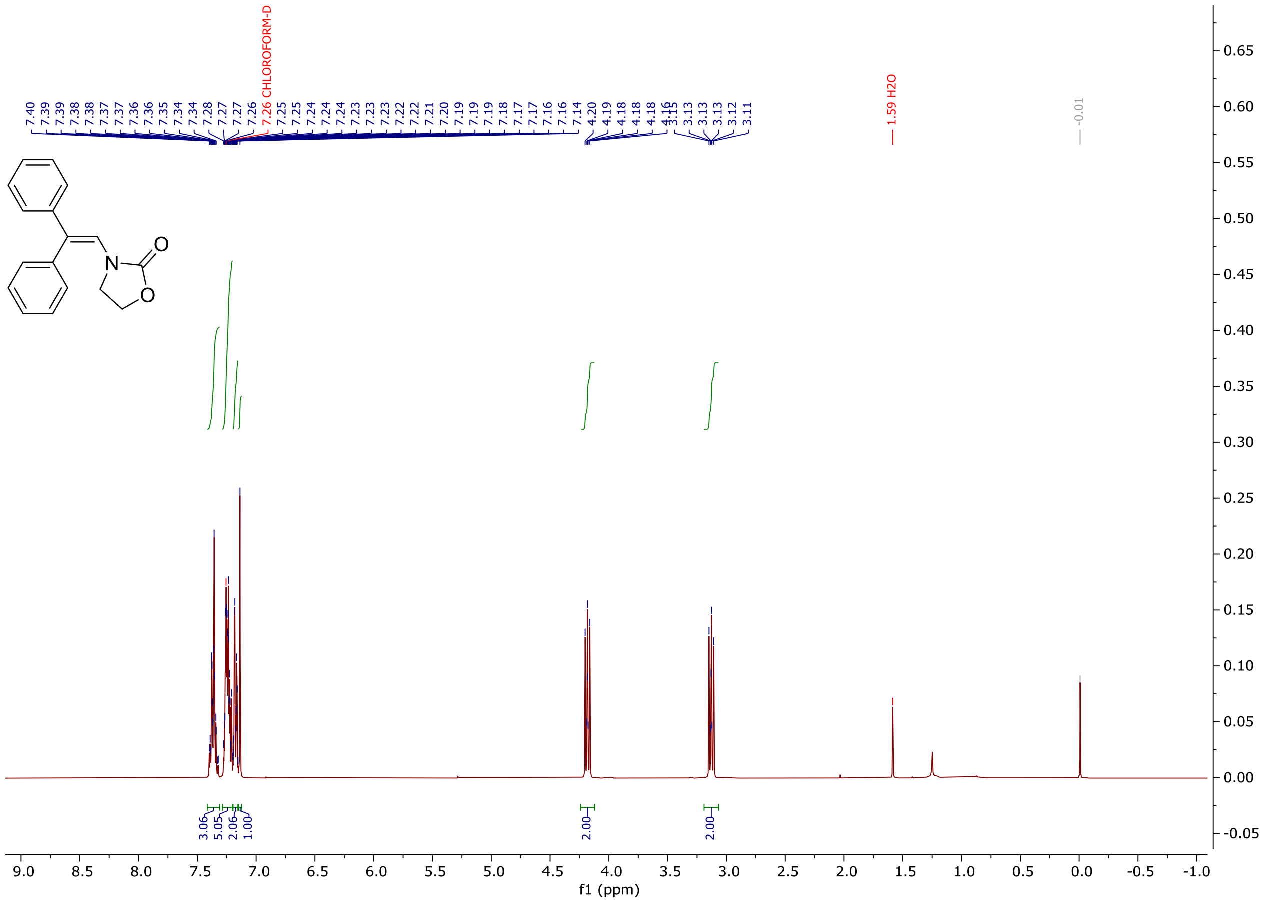

^{13}C-NMR (101 MHz; $CDCl_3$)

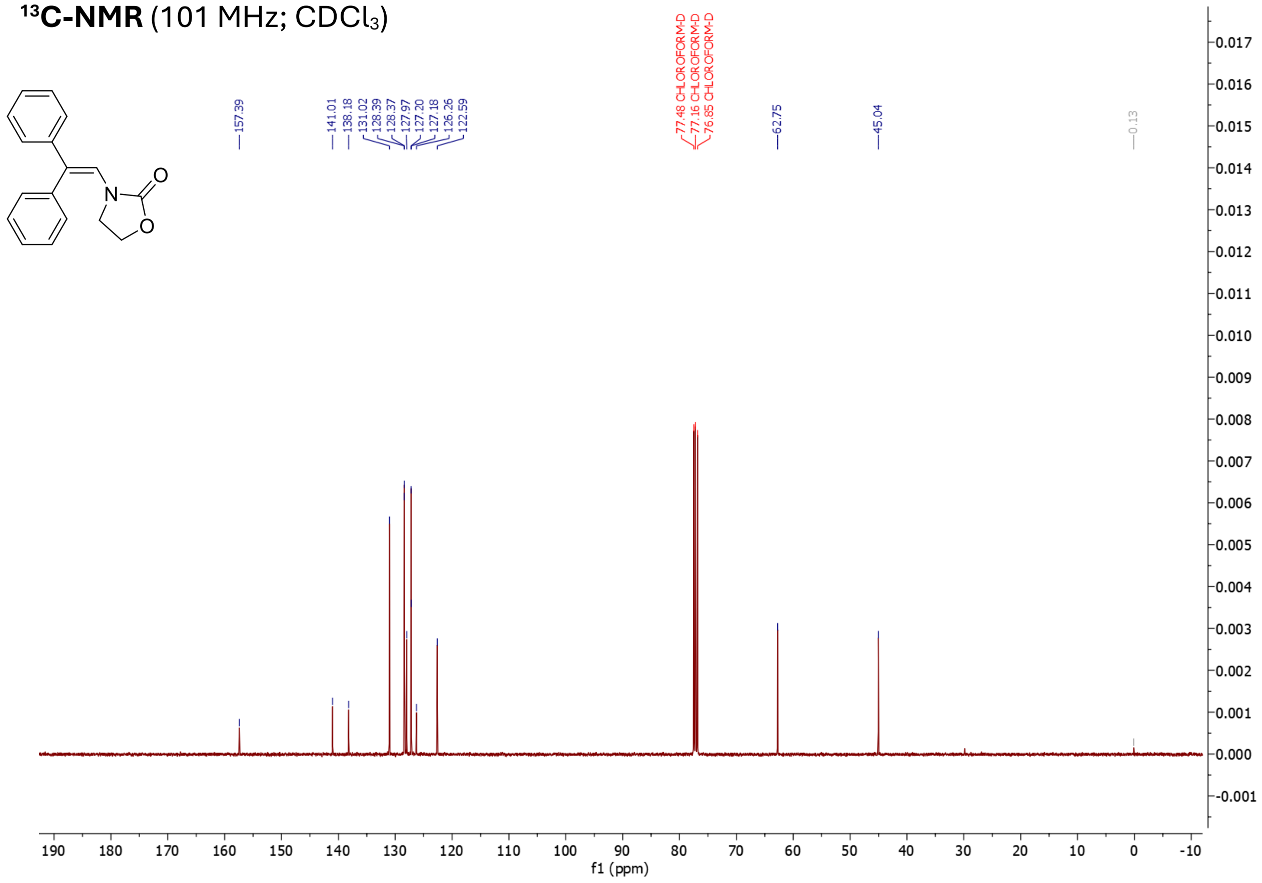

COSY ($CDCl_3$)

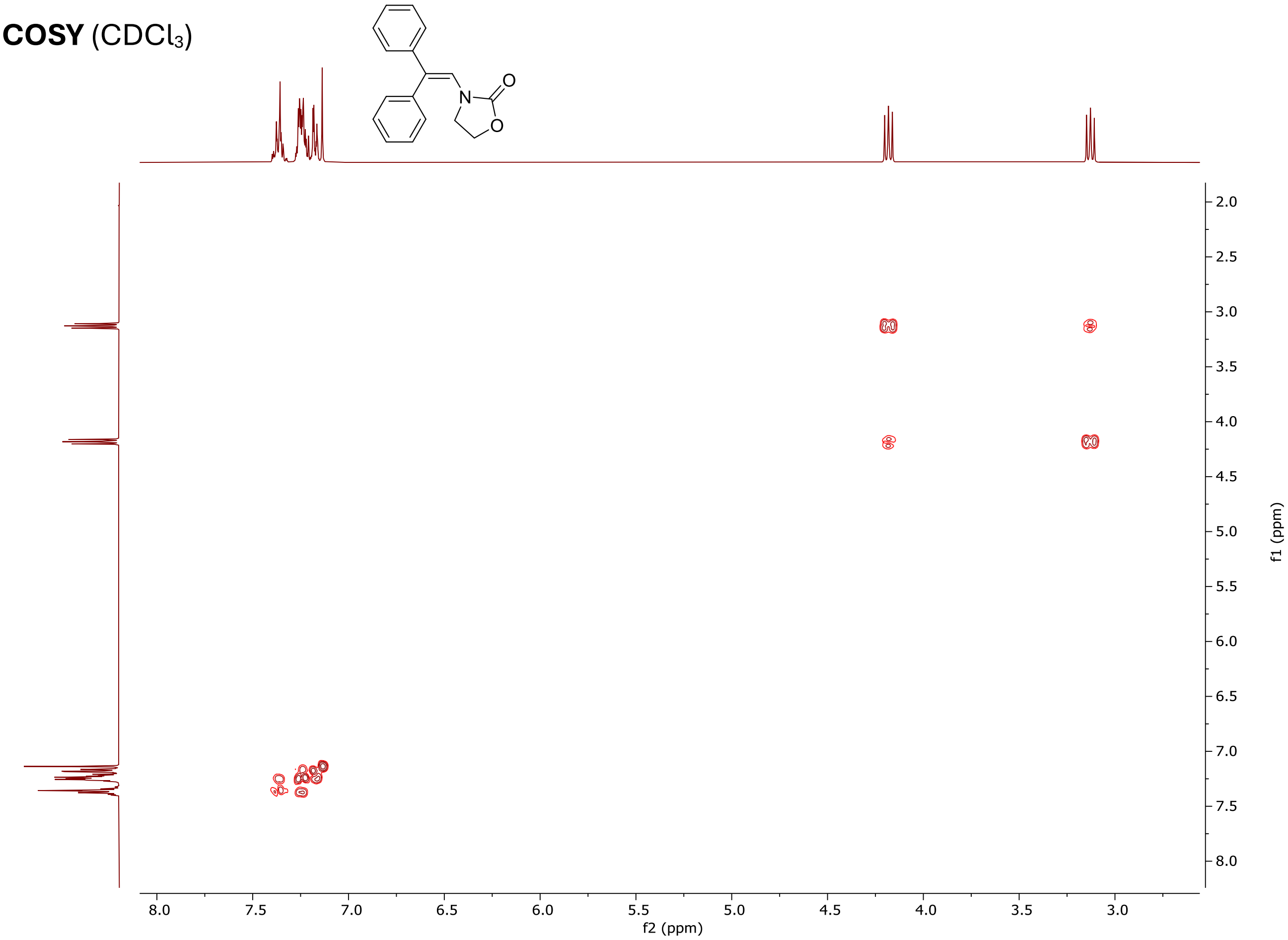

HSQC ($CDCl_3$)

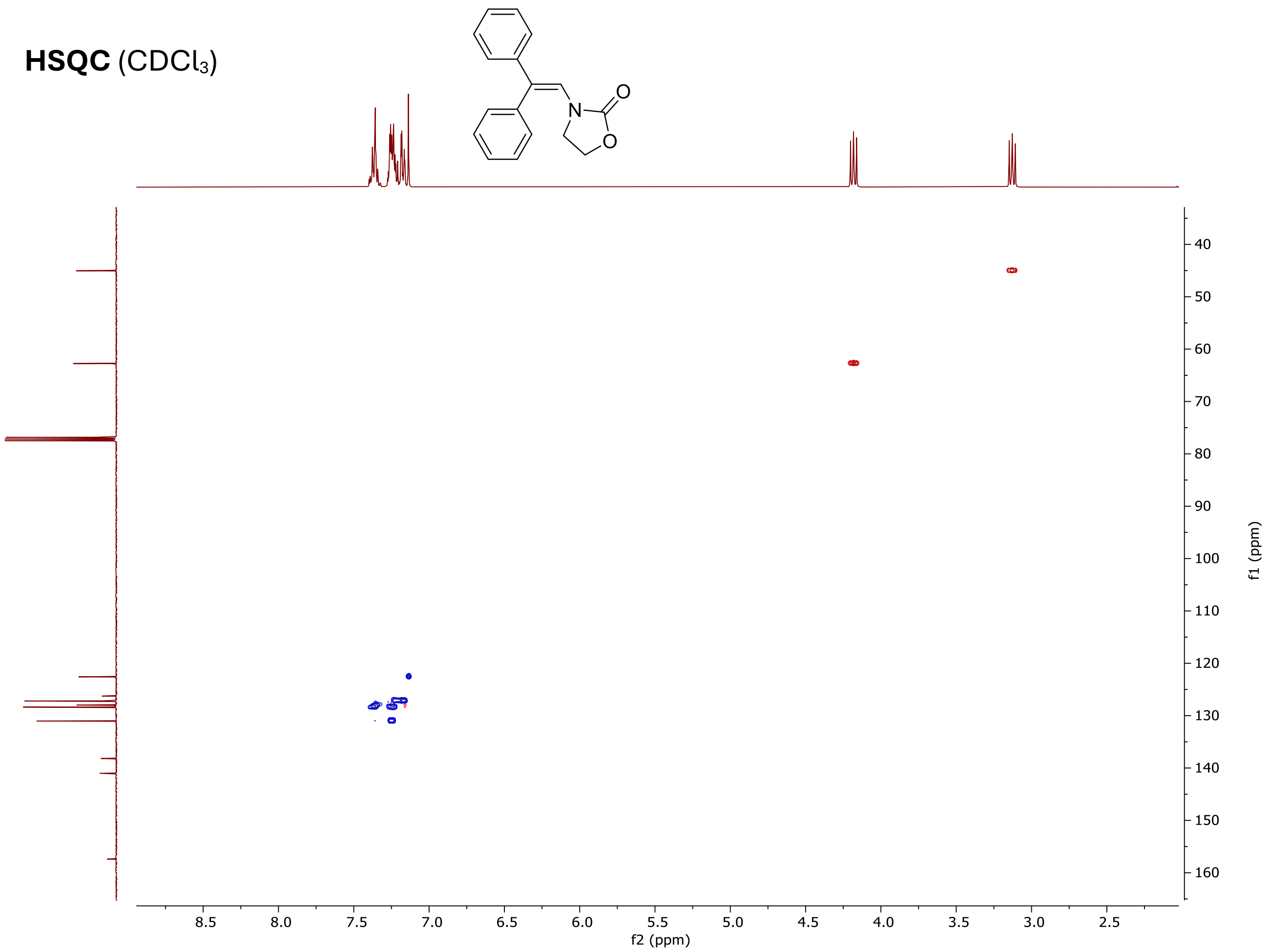

HMBC ($CDCl_3$)

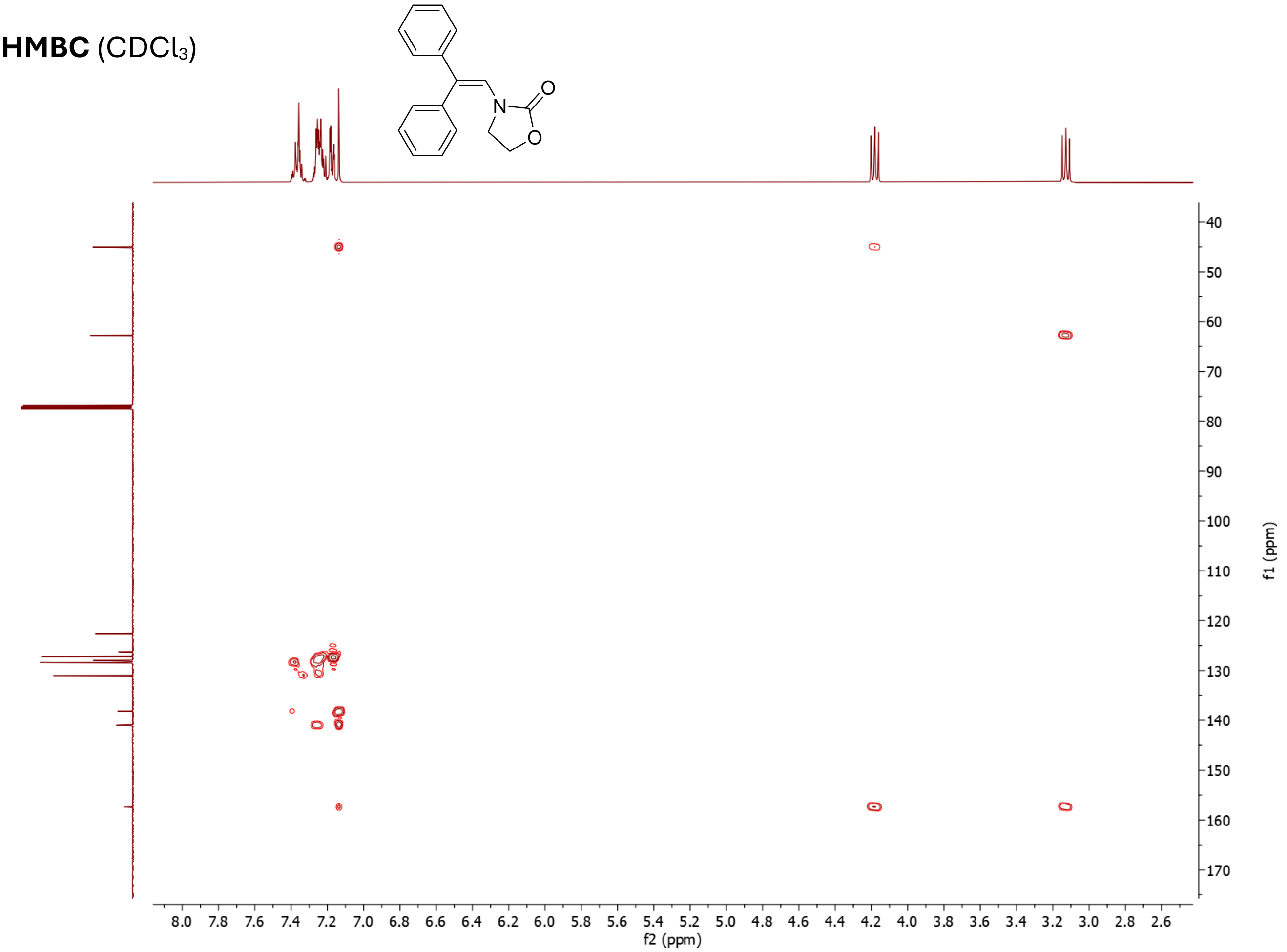

(Z)-3-(2-phenylprop-1-en-1-yl)oxazolidin-2-one (5p) **^{1}H-NMR** (300 MHz; $CDCl_3$)

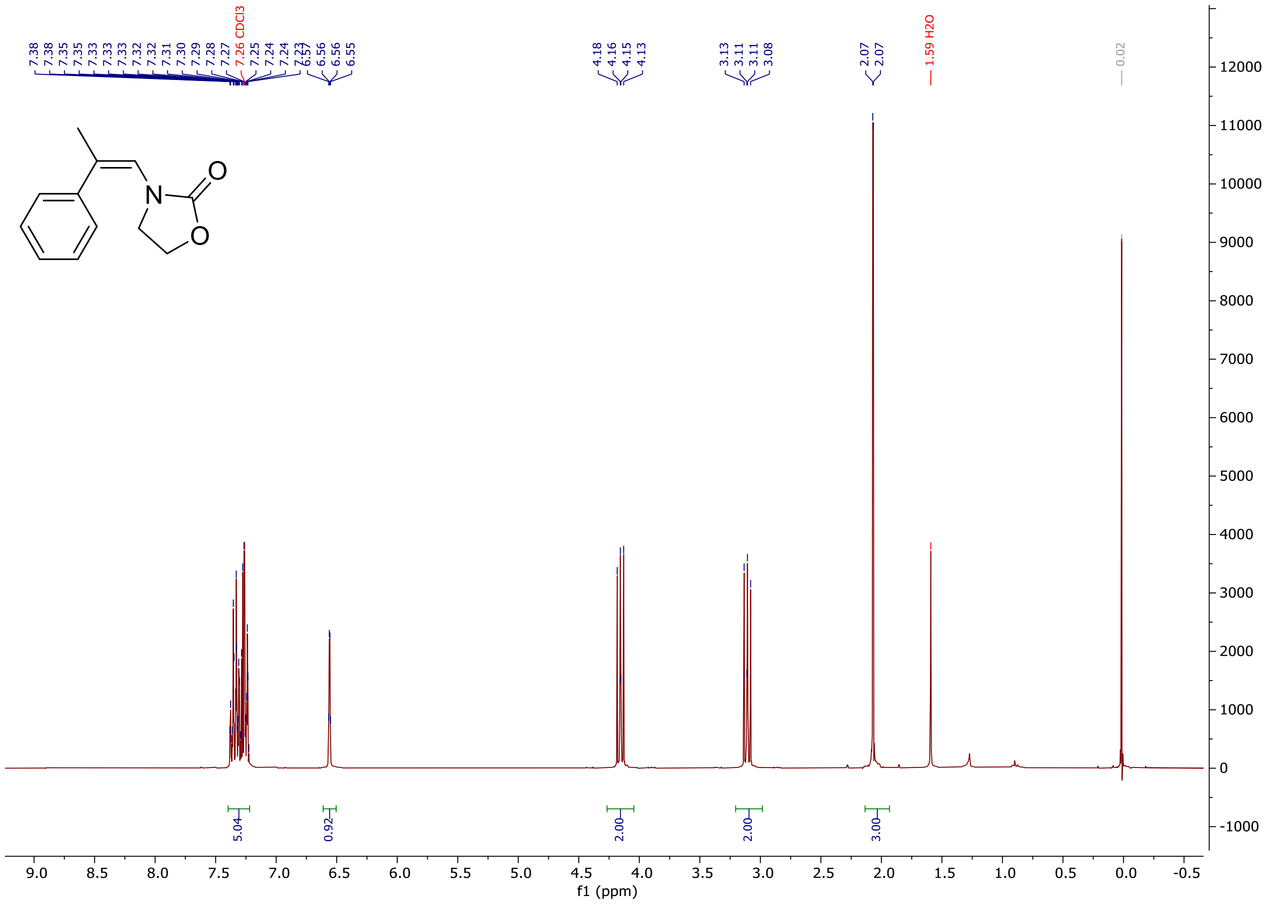

^{13}C-NMR (75 MHz; $CDCl_3$)

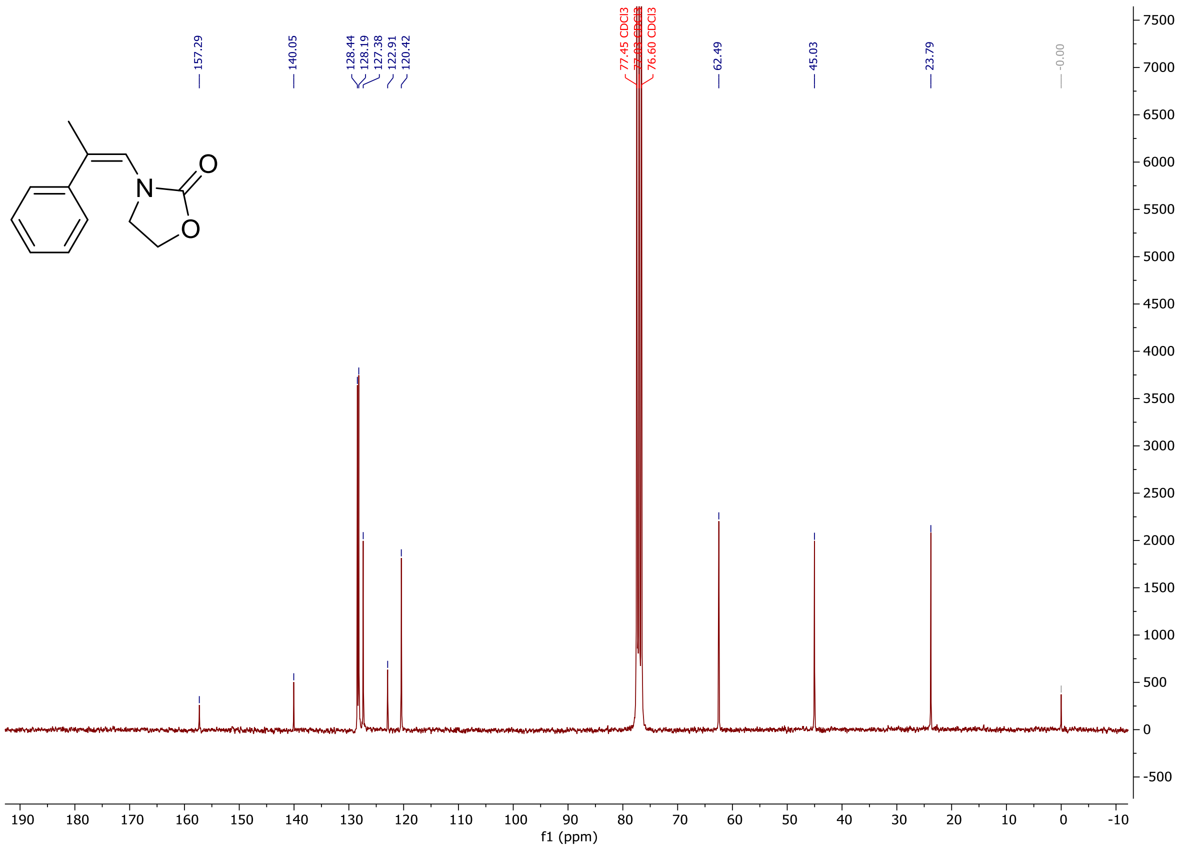

COSY ($CDCl_3$)

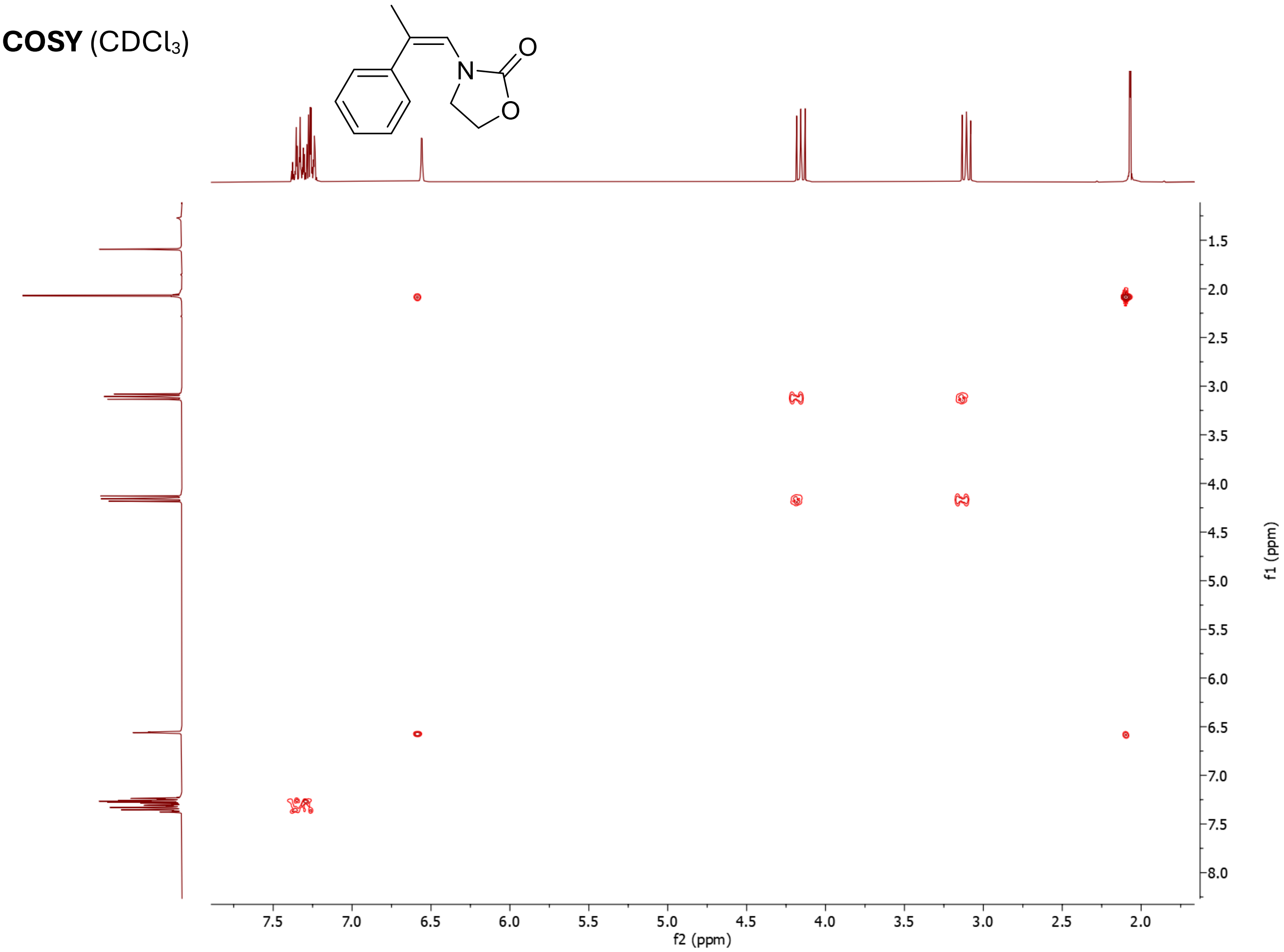

HSQC ($CDCl_3$)

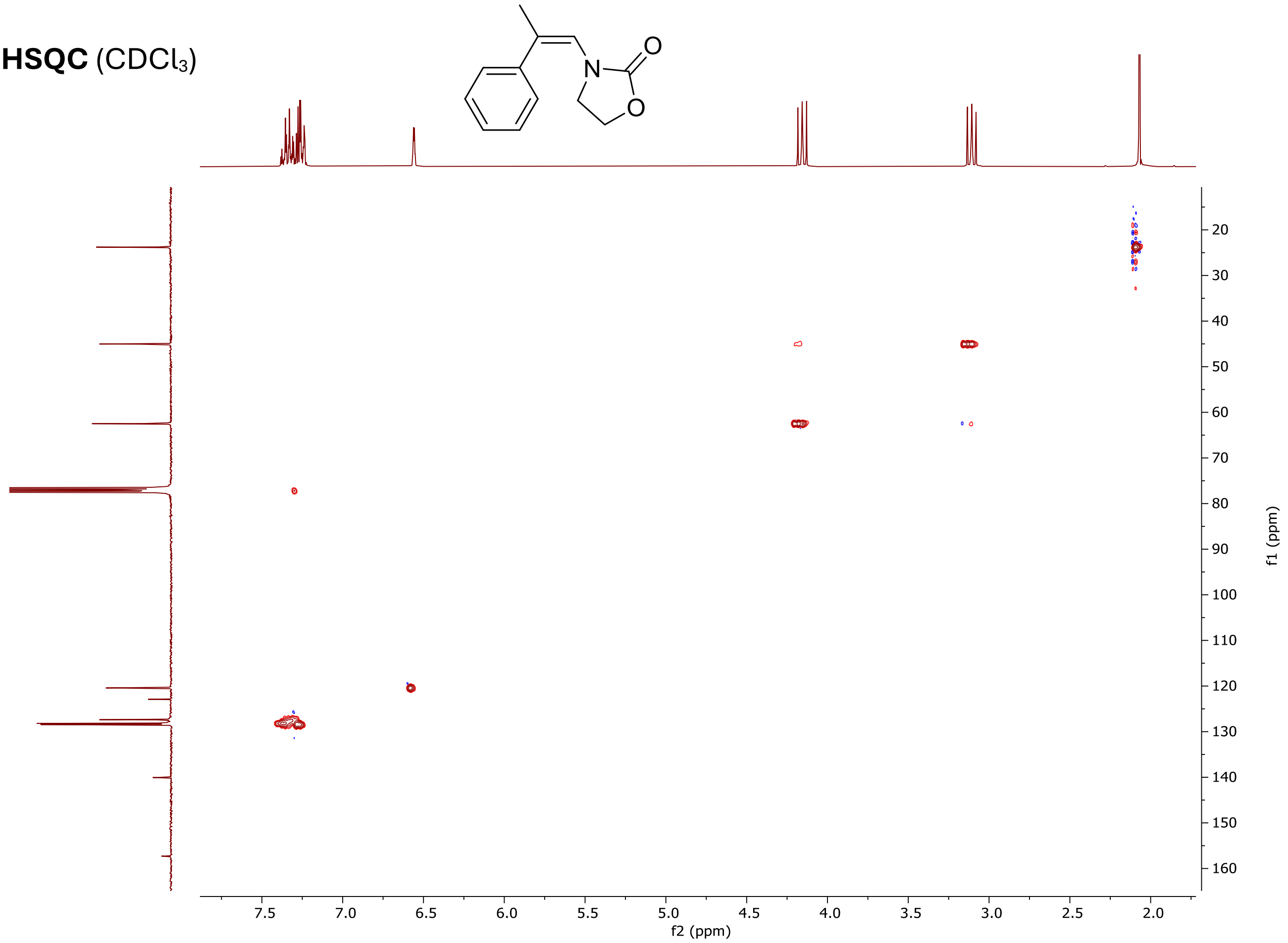

HMBC ($CDCl_3$)

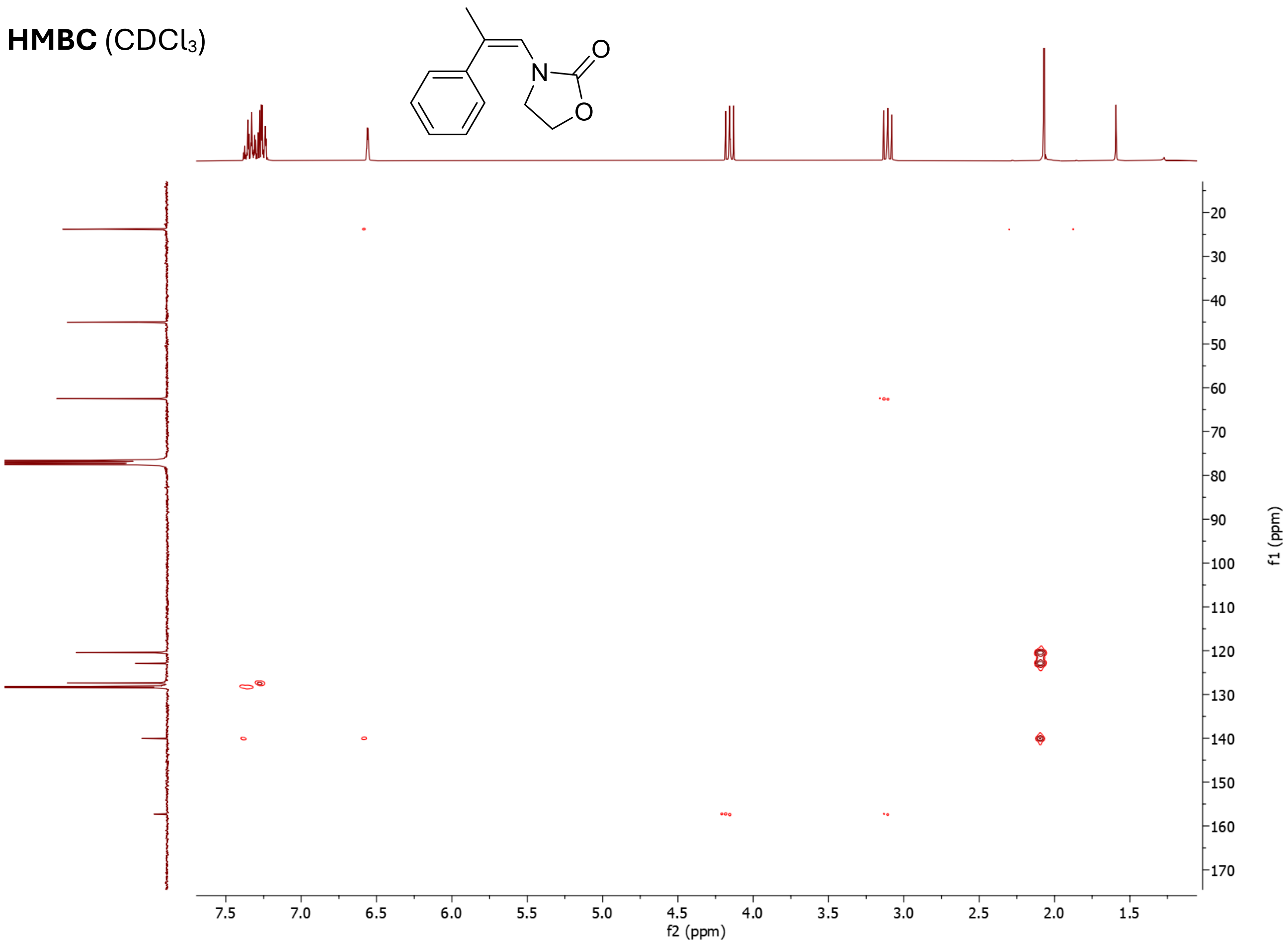

NOESY ($CDCl_3$)

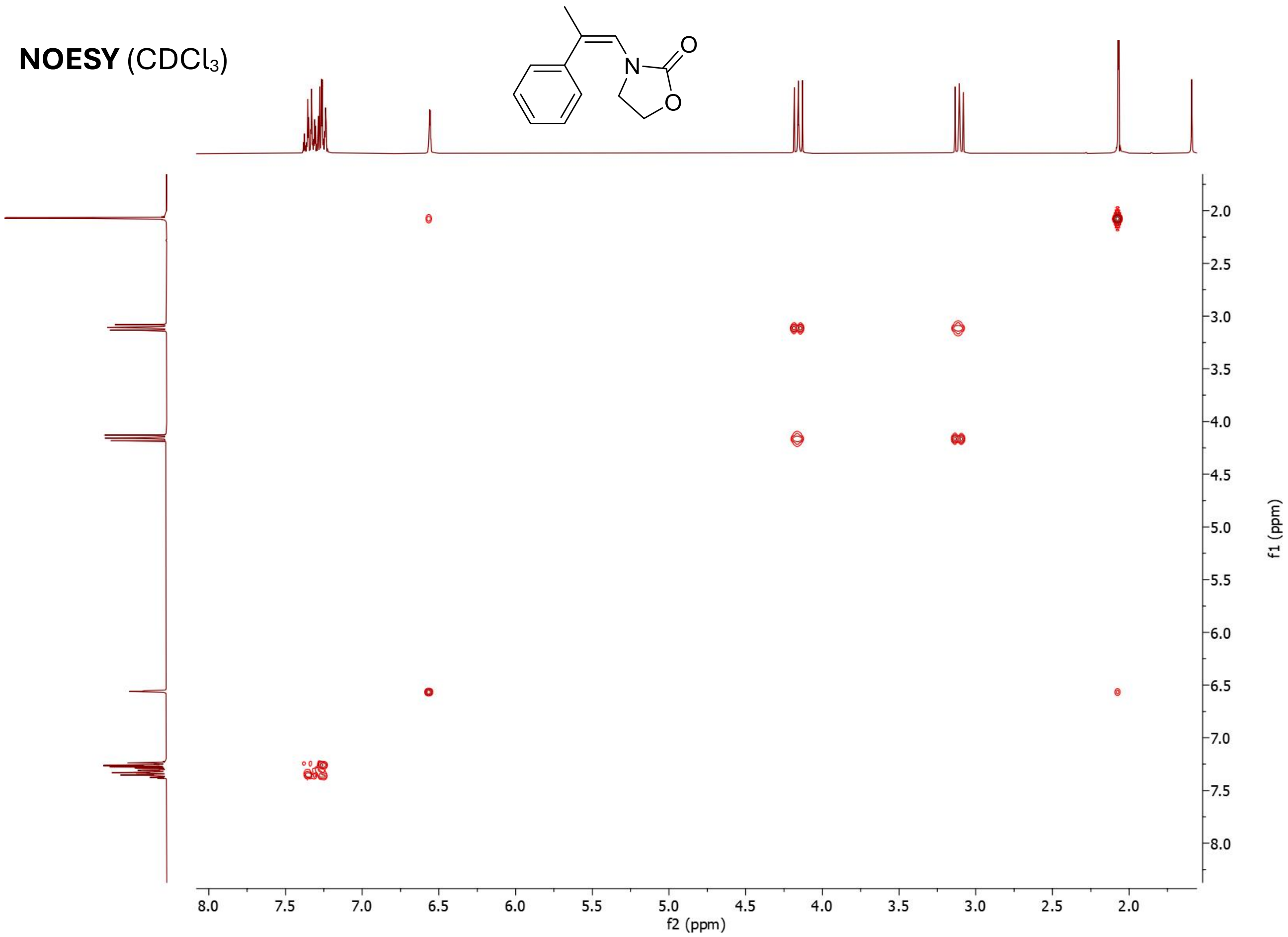

(Z)-3-(4-(tert-butyl)styryl)oxazolidin-2-one (5q) **^{1}H-NMR** (300 MHz; $CDCl_3$)

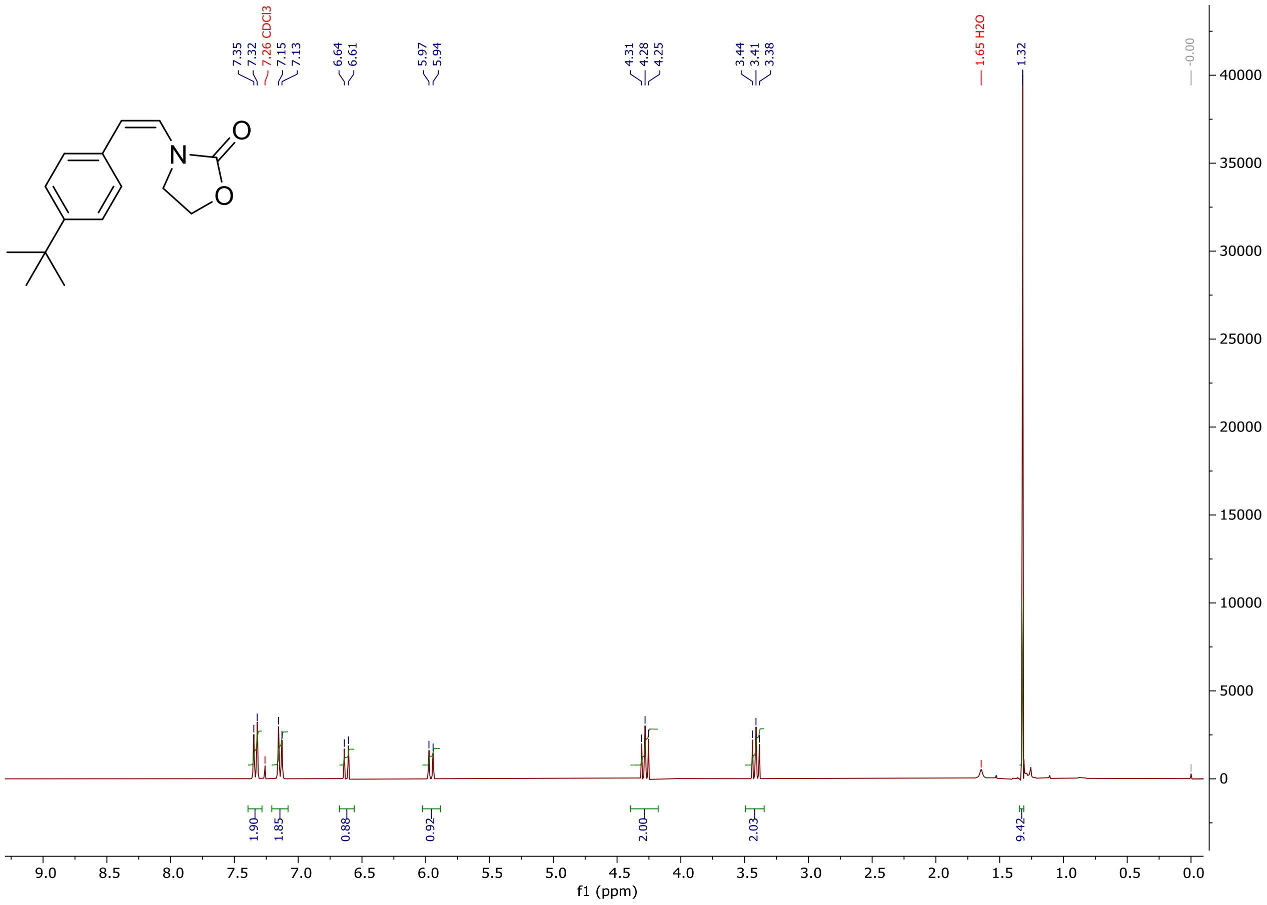

^{13}C-NMR (75 MHz; $CDCl_3$)

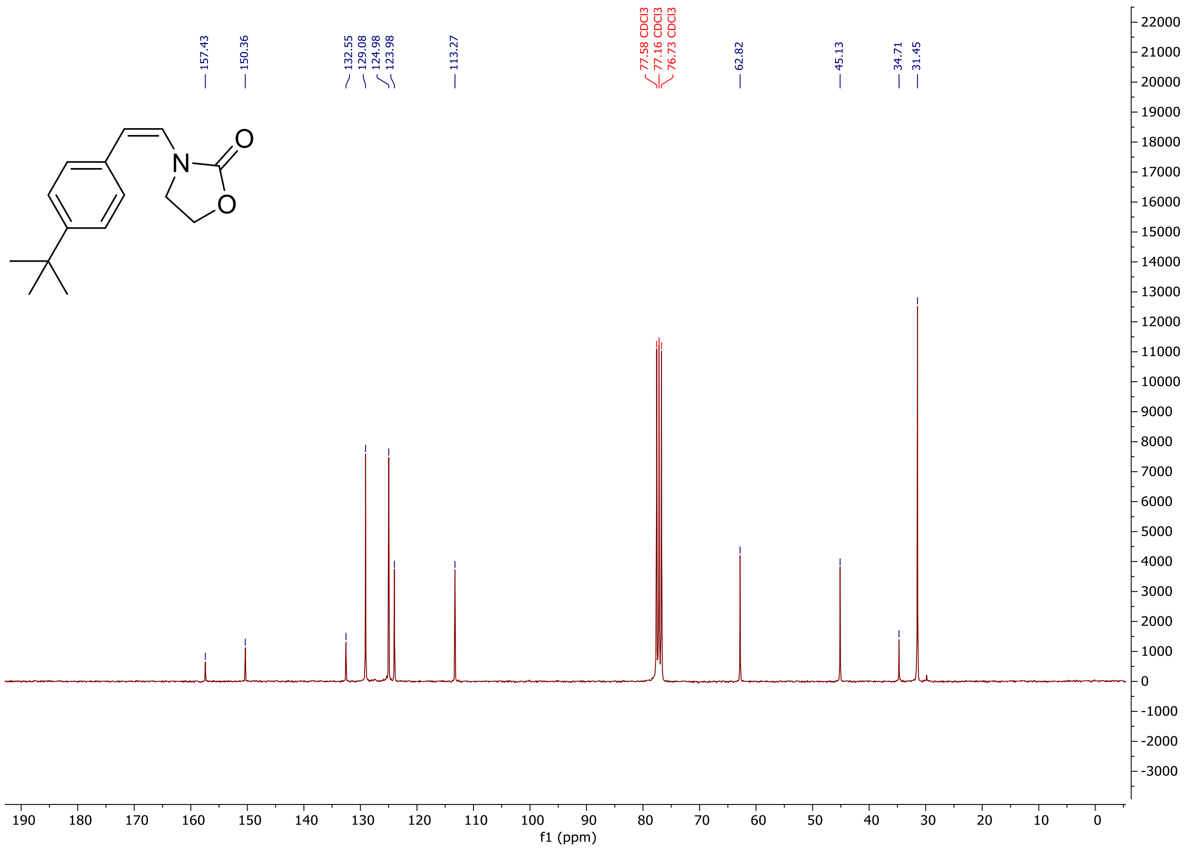

COSY ($CDCl_3$)

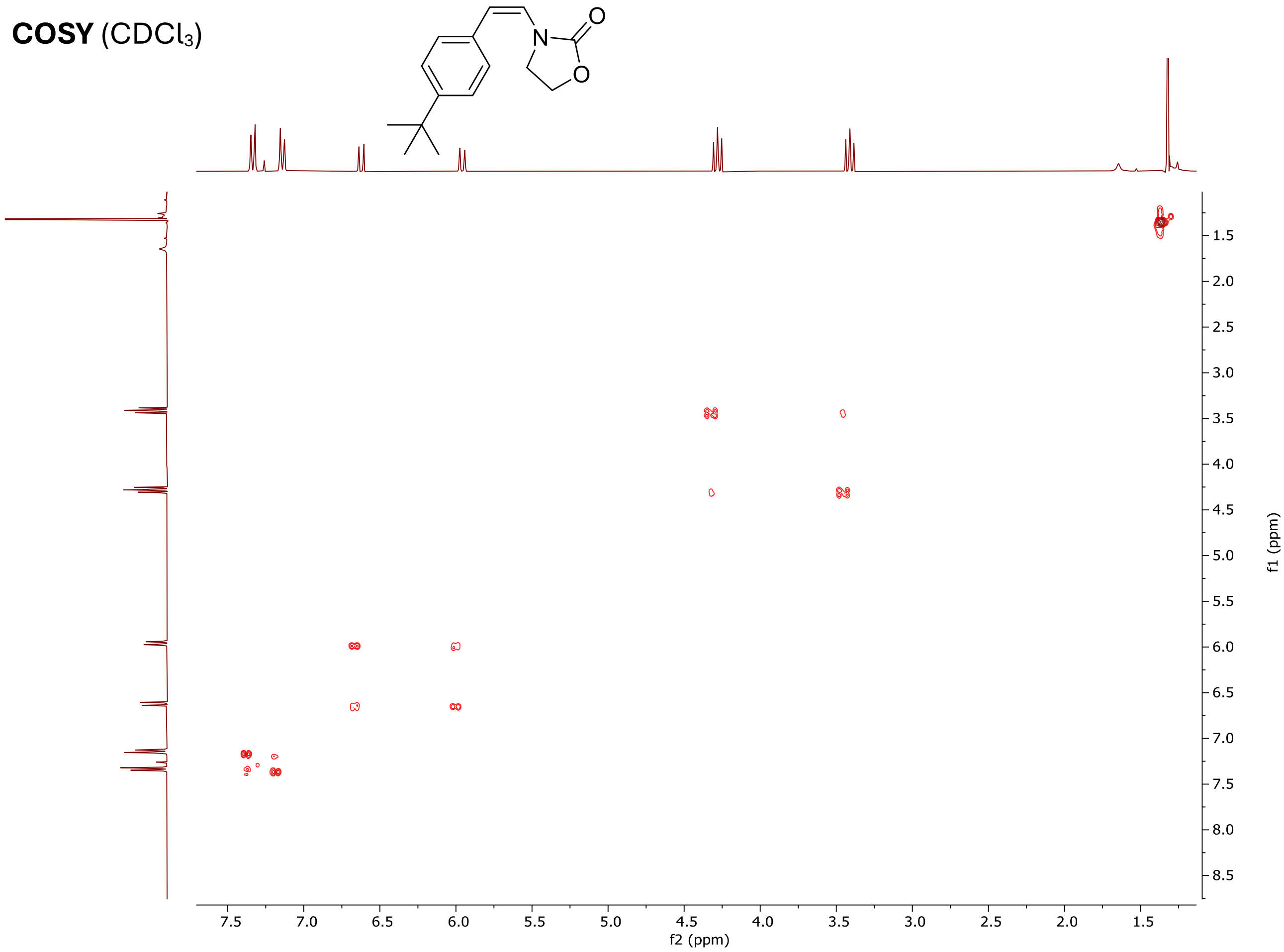

HSQC ($CDCl_3$)

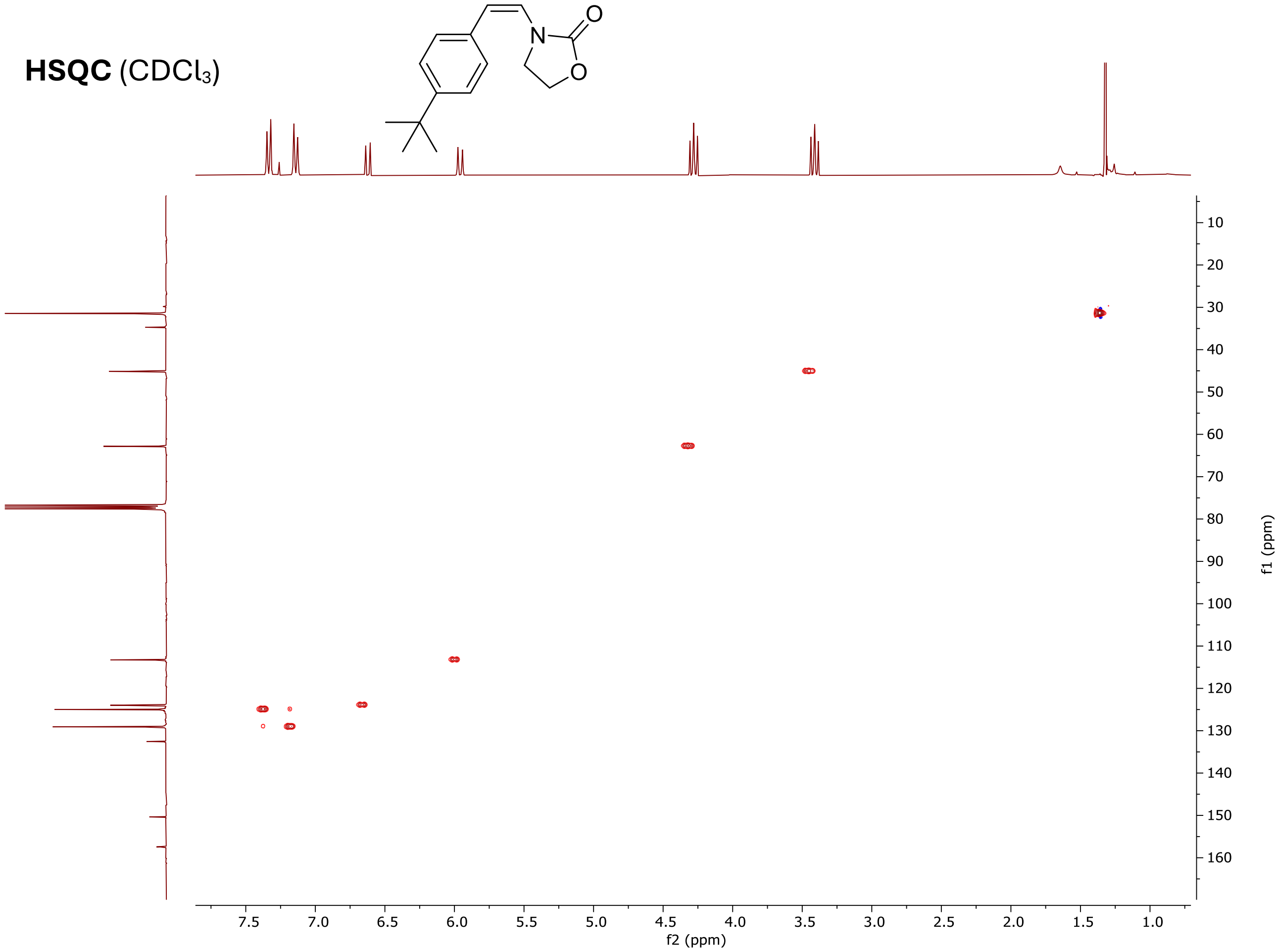

HMBC ($CDCl_3$)

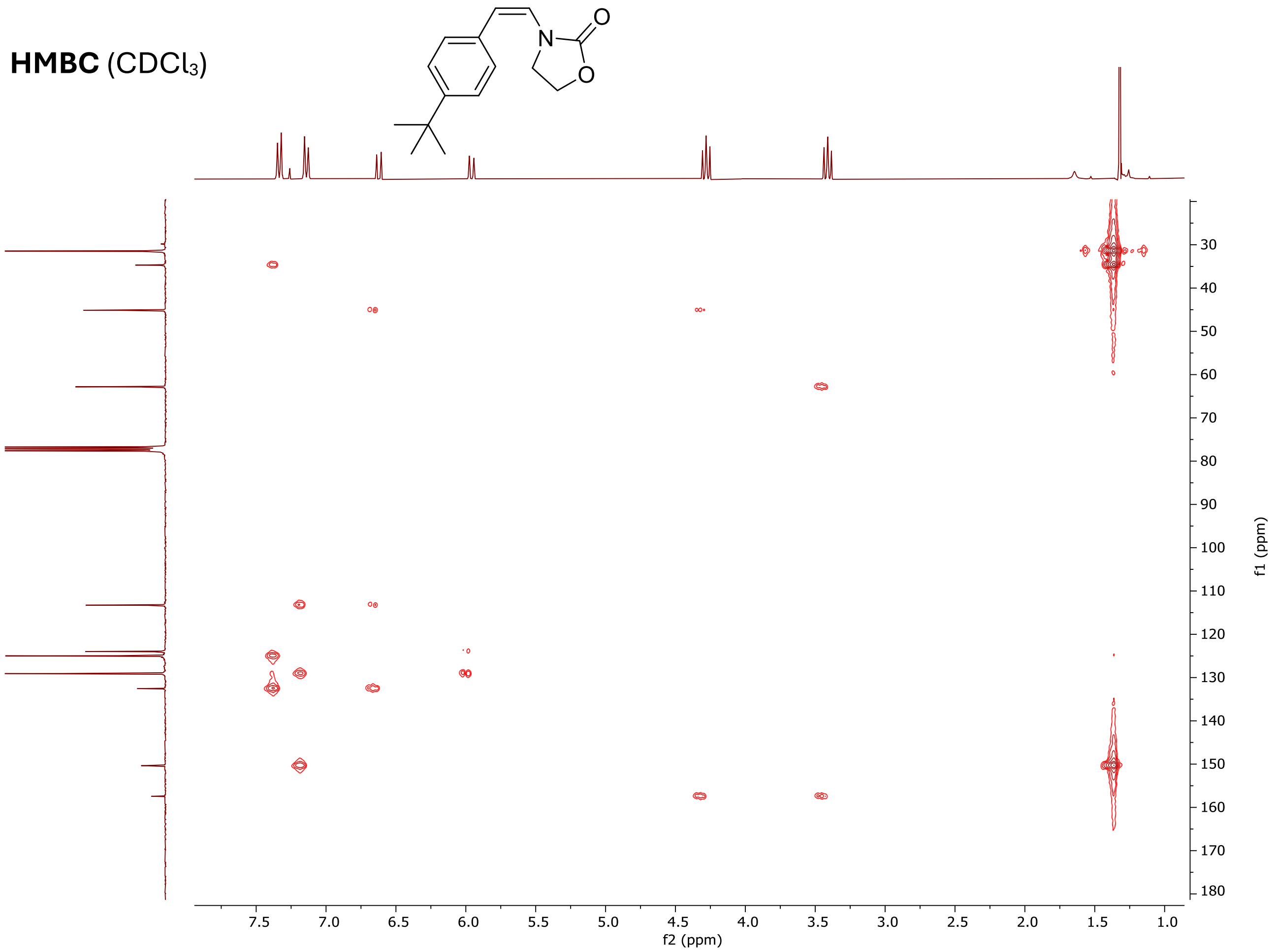

3-(2,4,6-trimethoxyphenyl) oxazolidin-2-one (5r) [1]**H-NMR** (300 MHz; $CDCl_3$)

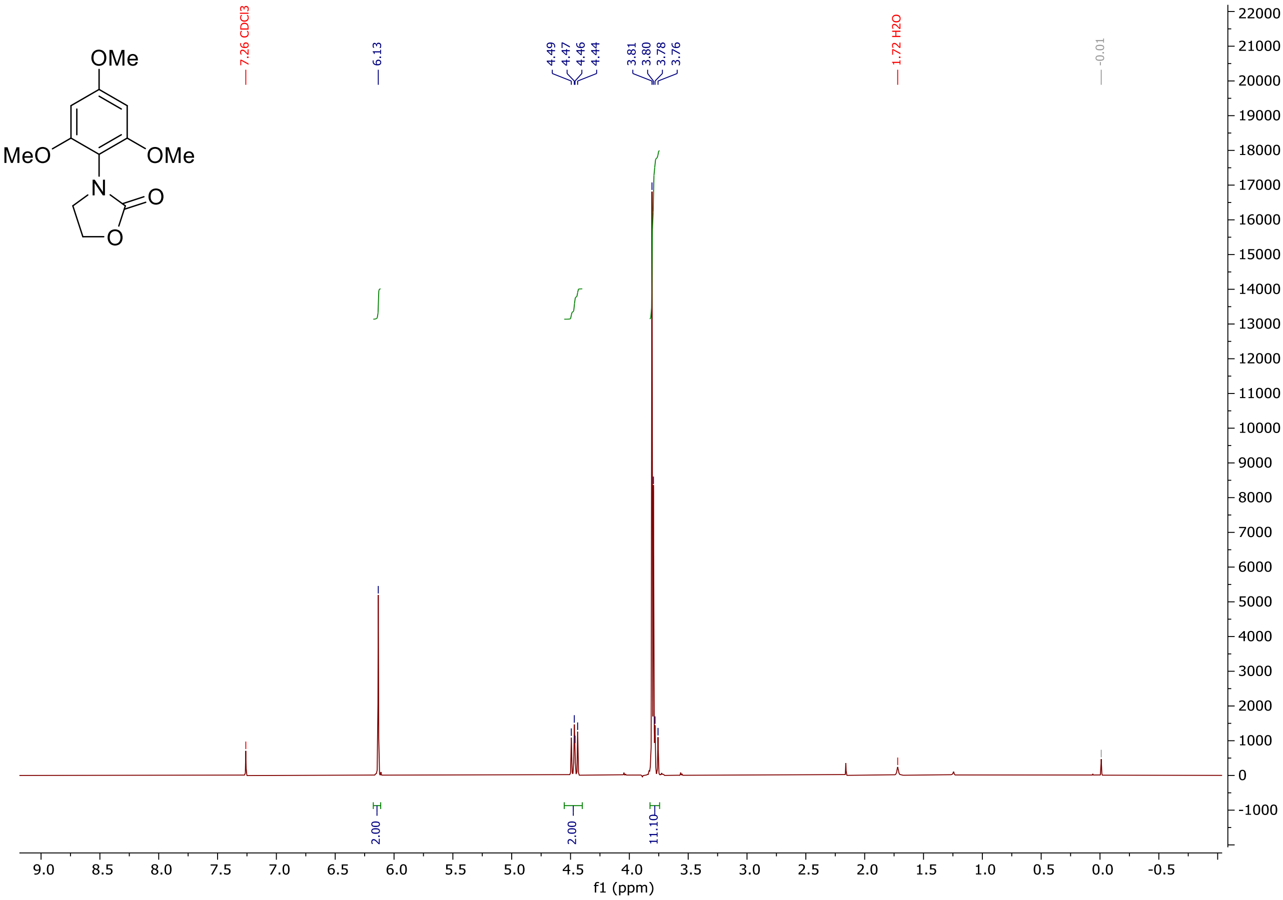

^{13}C-NMR (75 MHz; $CDCl_3$)

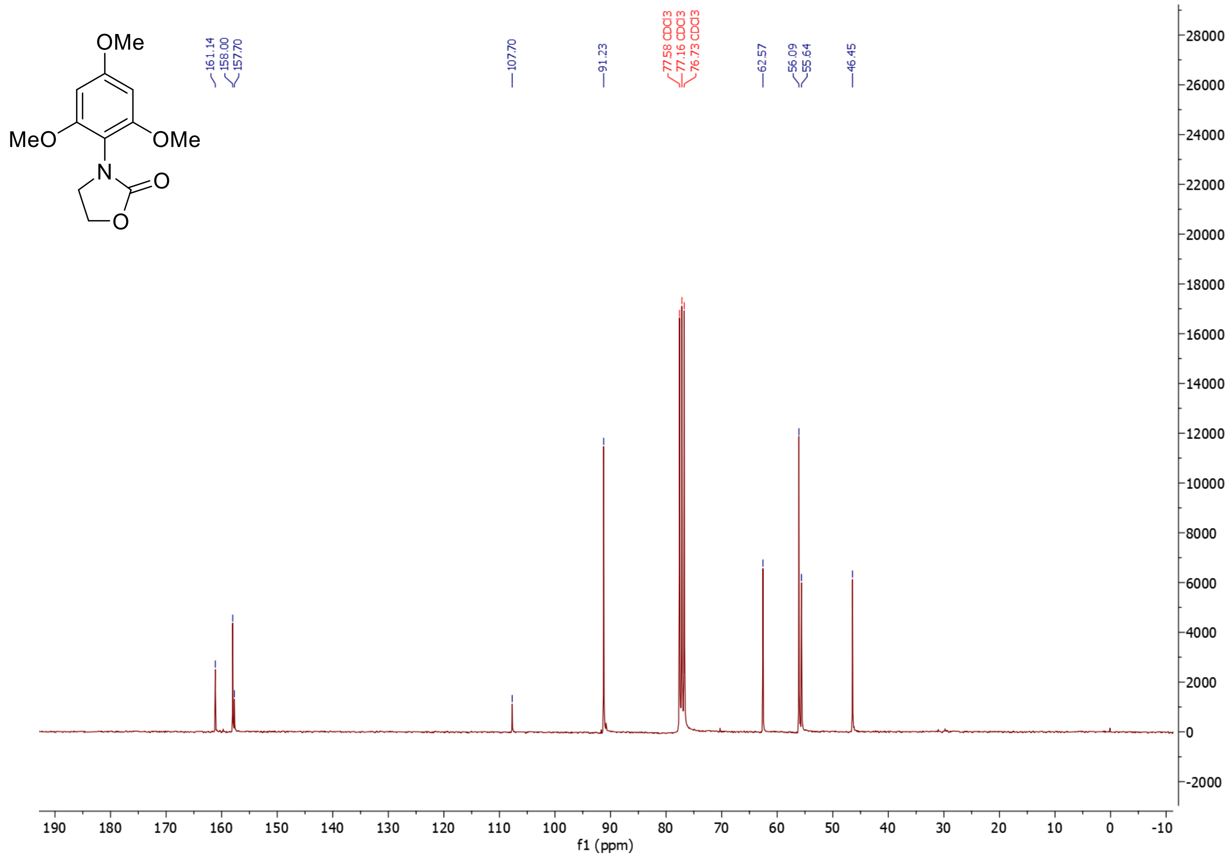

COSY ($CDCl_3$)

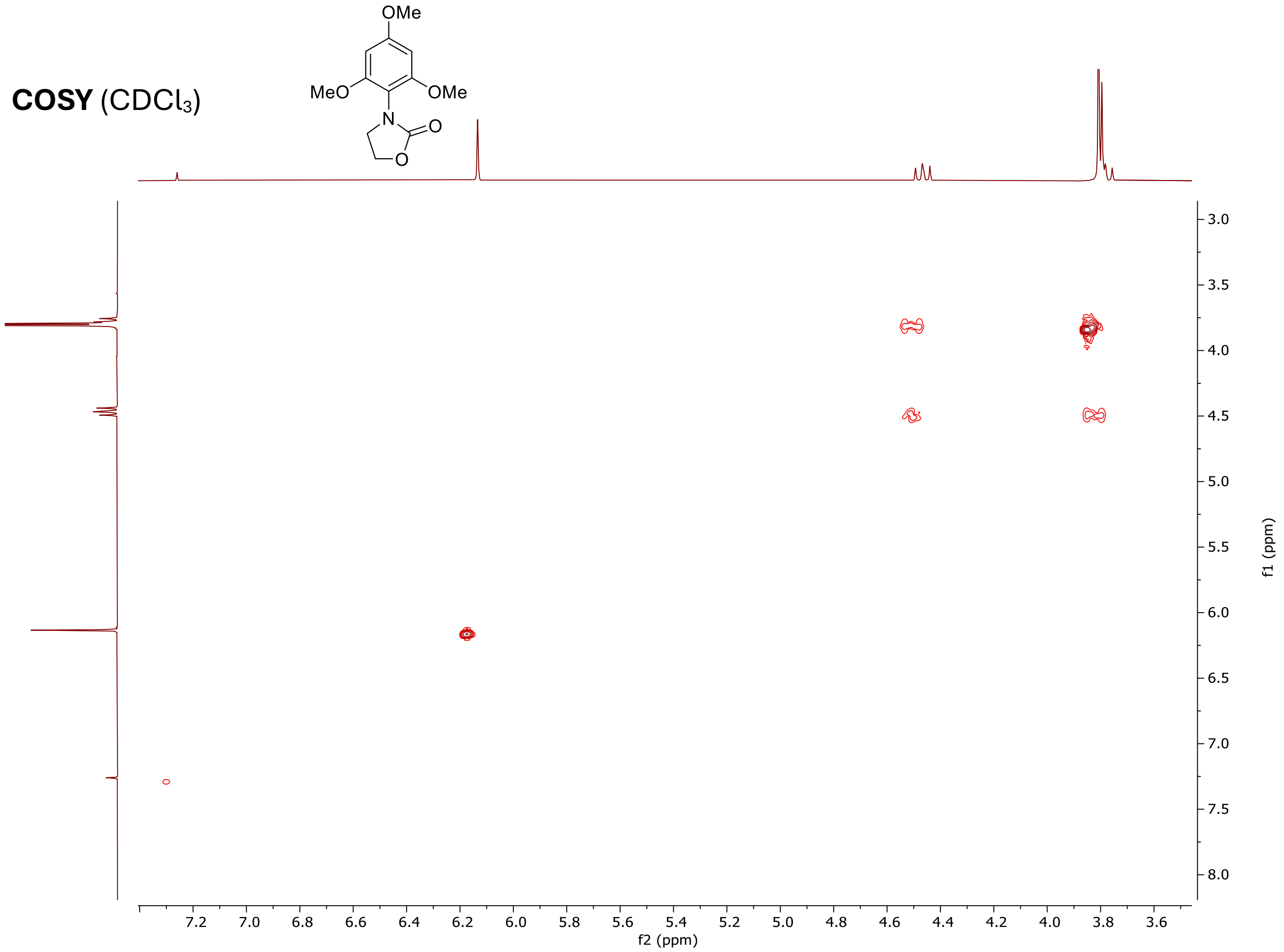

HSQC ($CDCl_3$)

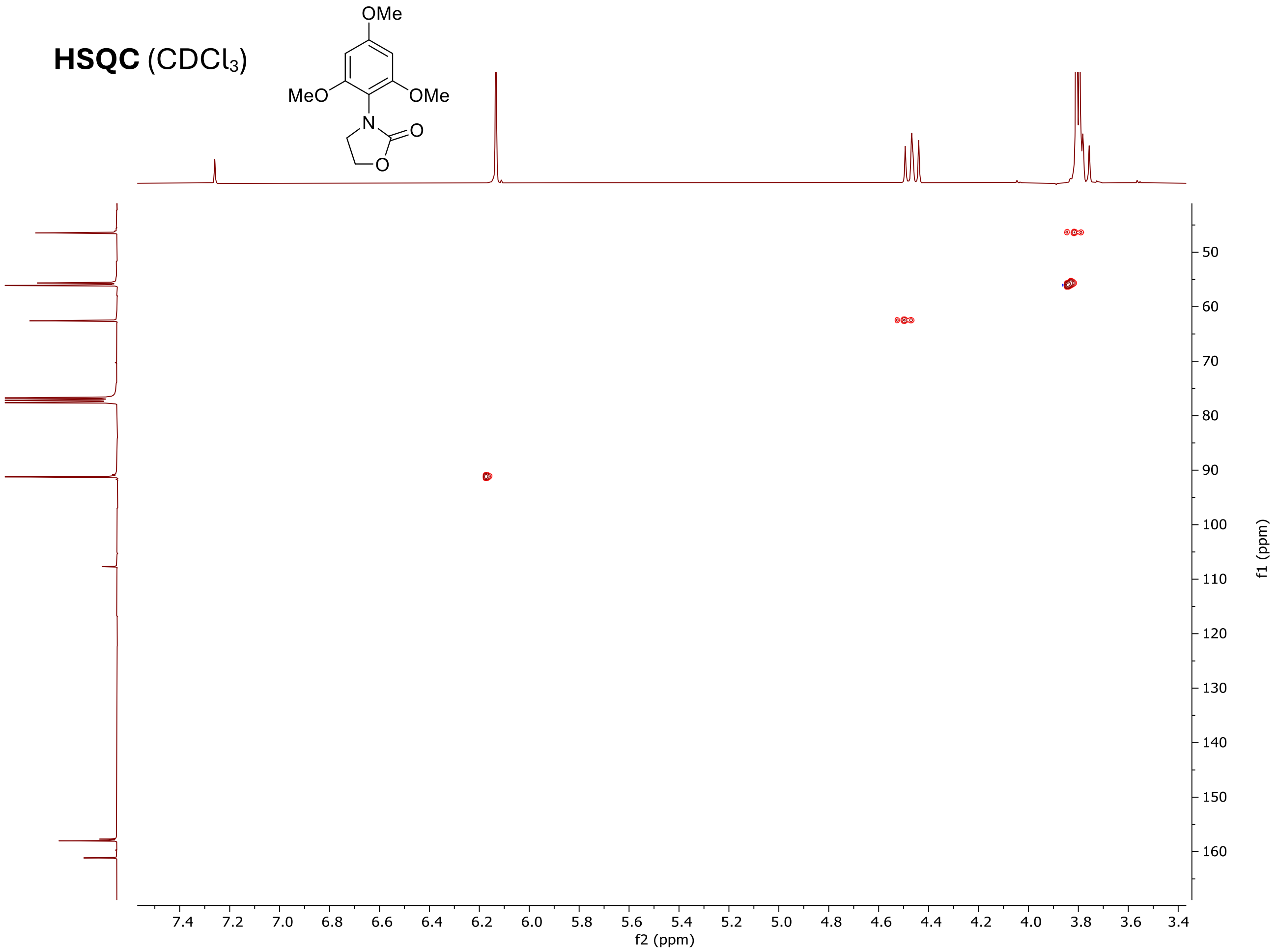

HMBC ($CDCl_3$)

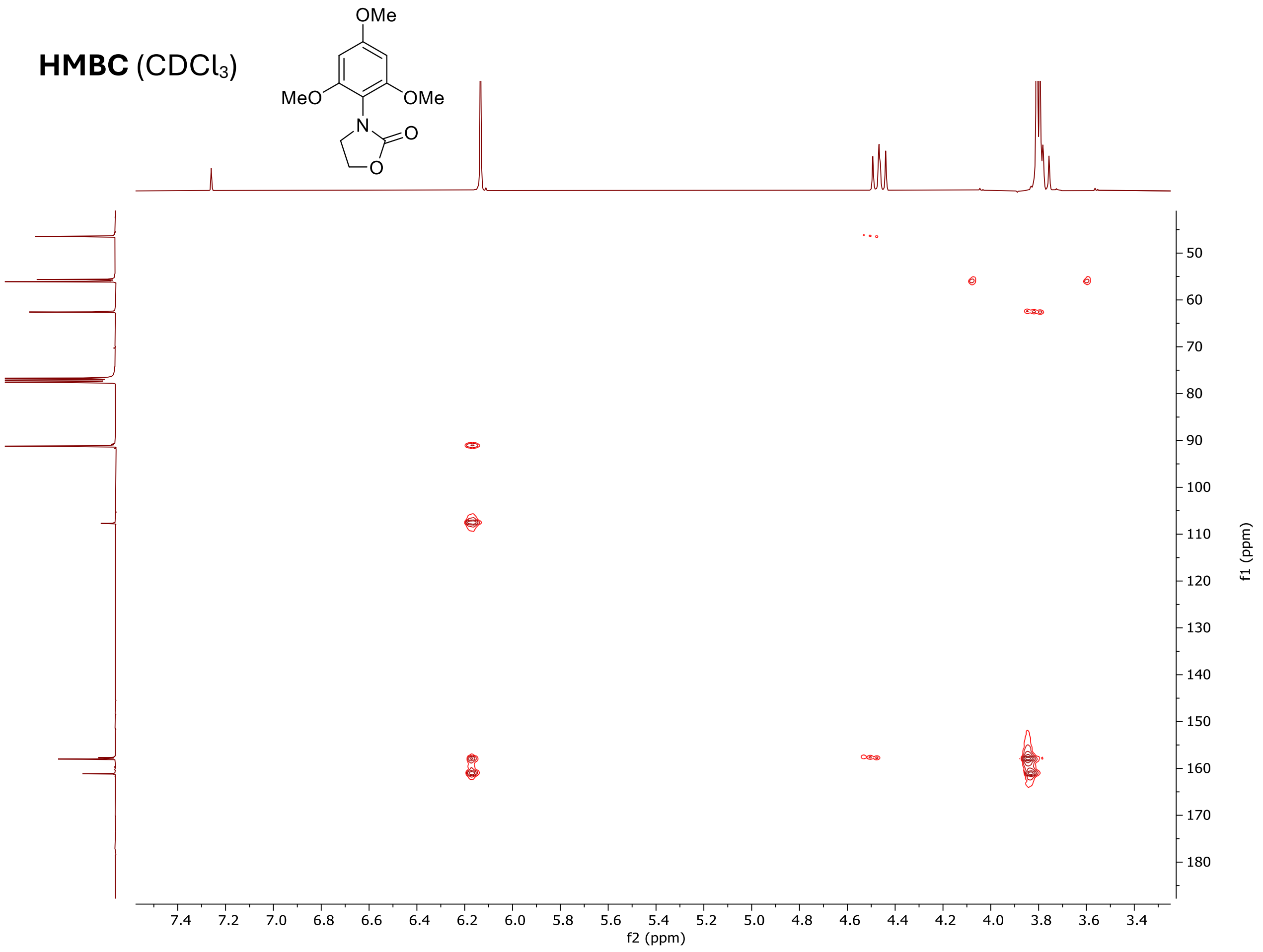

3,3'-(1-methyl-1H-indole-2,3-diyl)bis(oxazolidin-2-one) (6a)

^{1}H-NMR (400 MHz; $CDCl_3$)

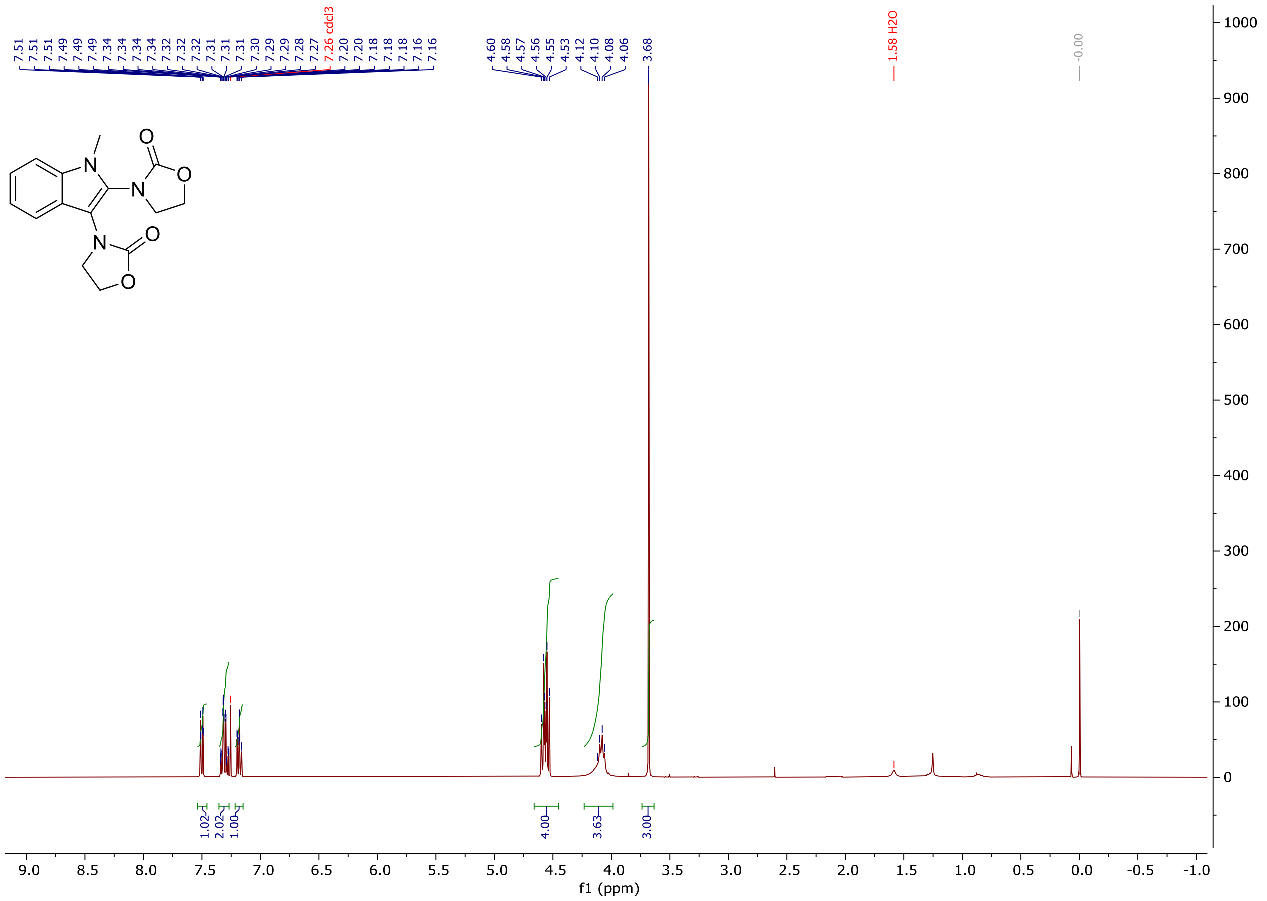

^{13}C-NMR (101 MHz; $CDCl_3$)

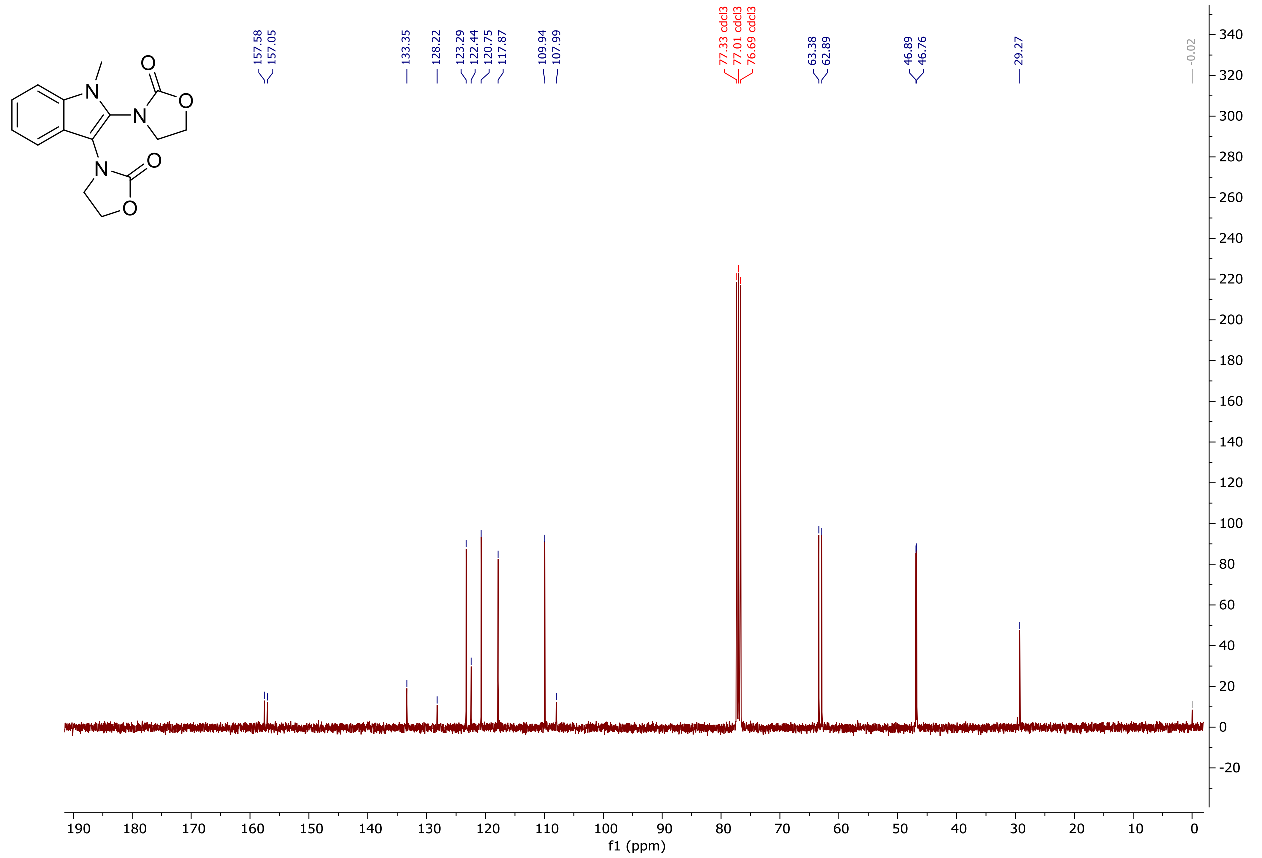

COSY ($CDCl_3$)

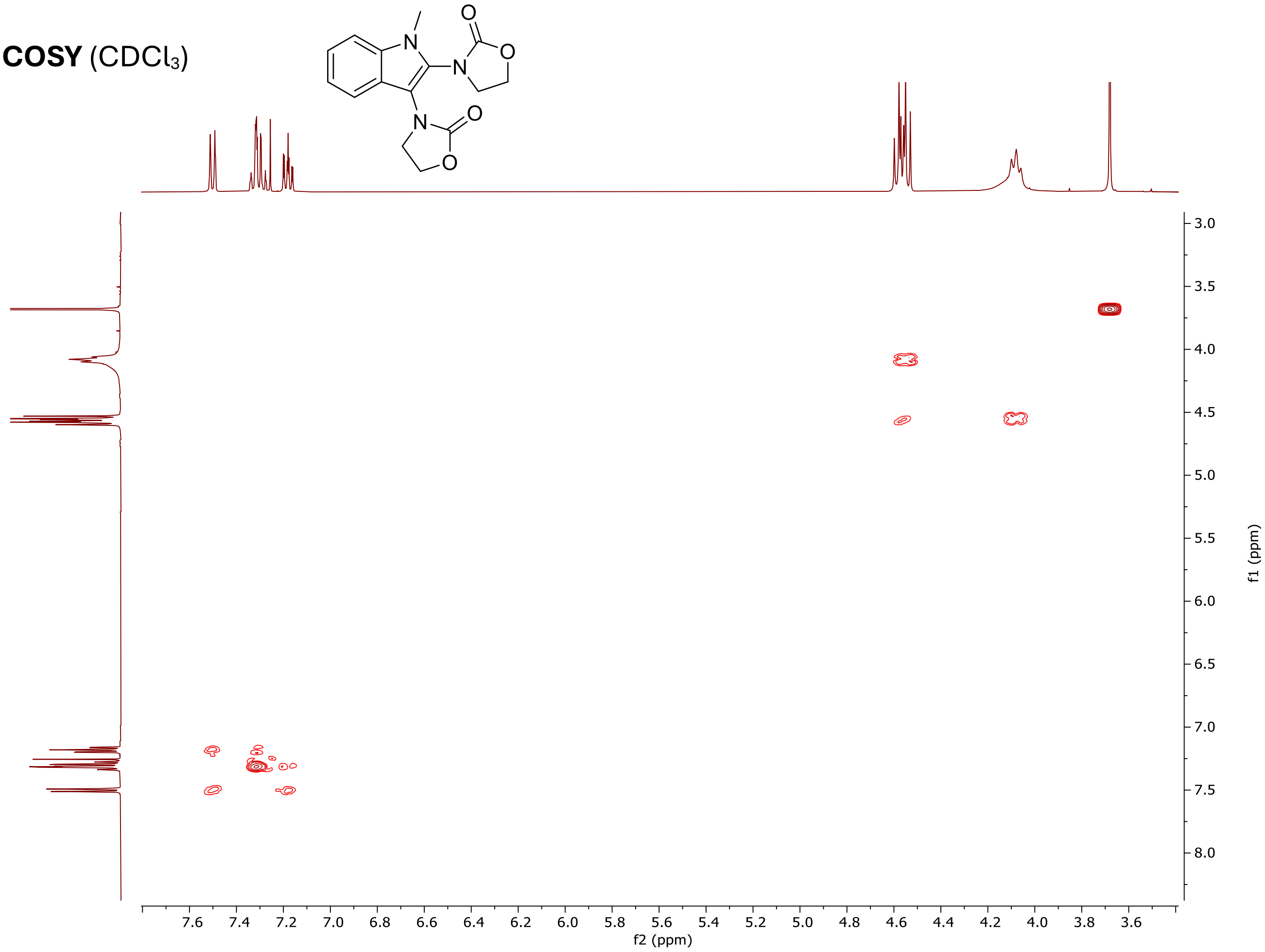

HSQC ($CDCl_3$)

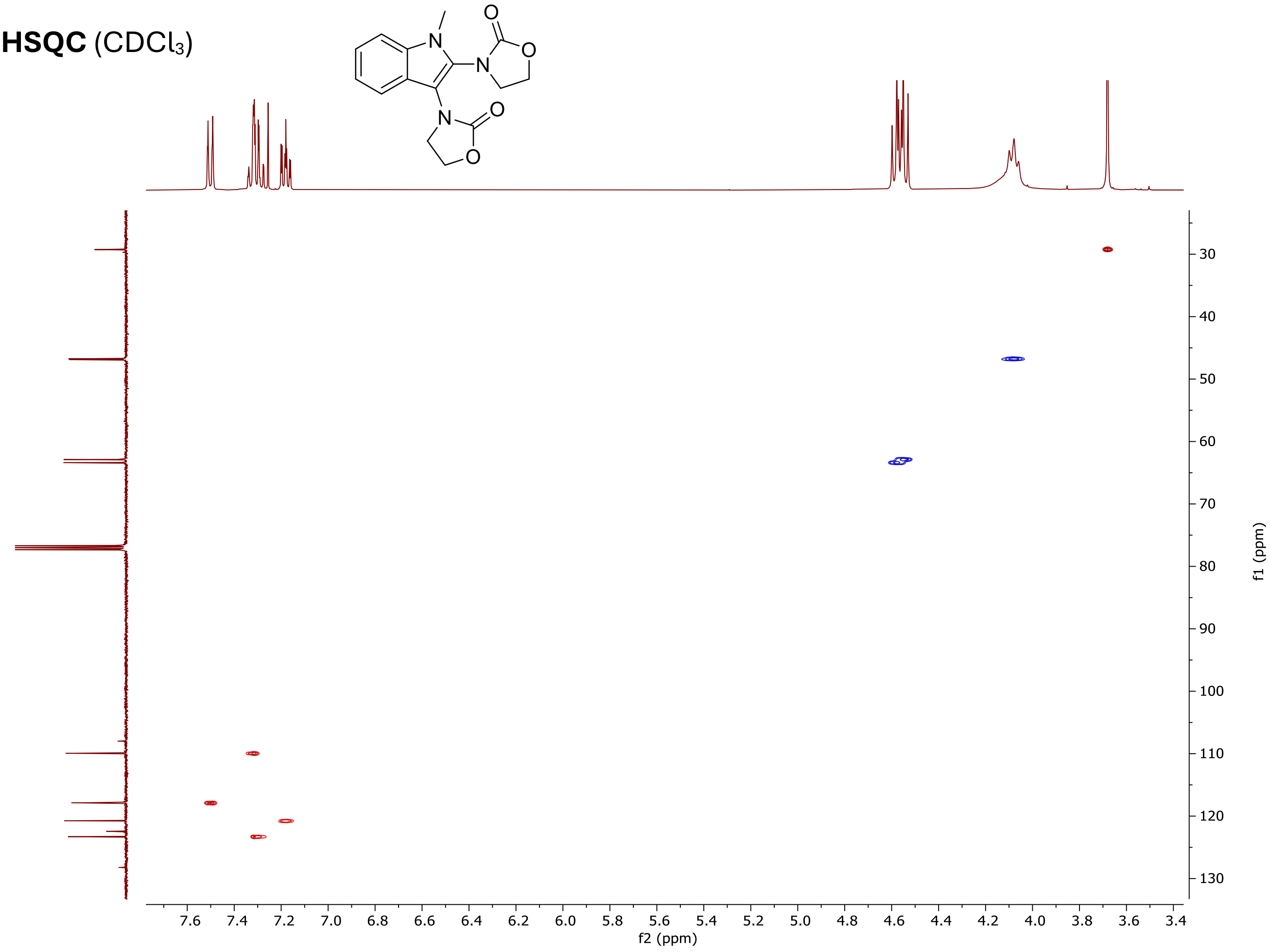

HMBC ($CDCl_3$)

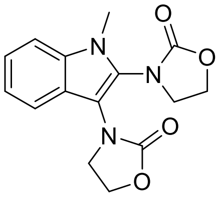

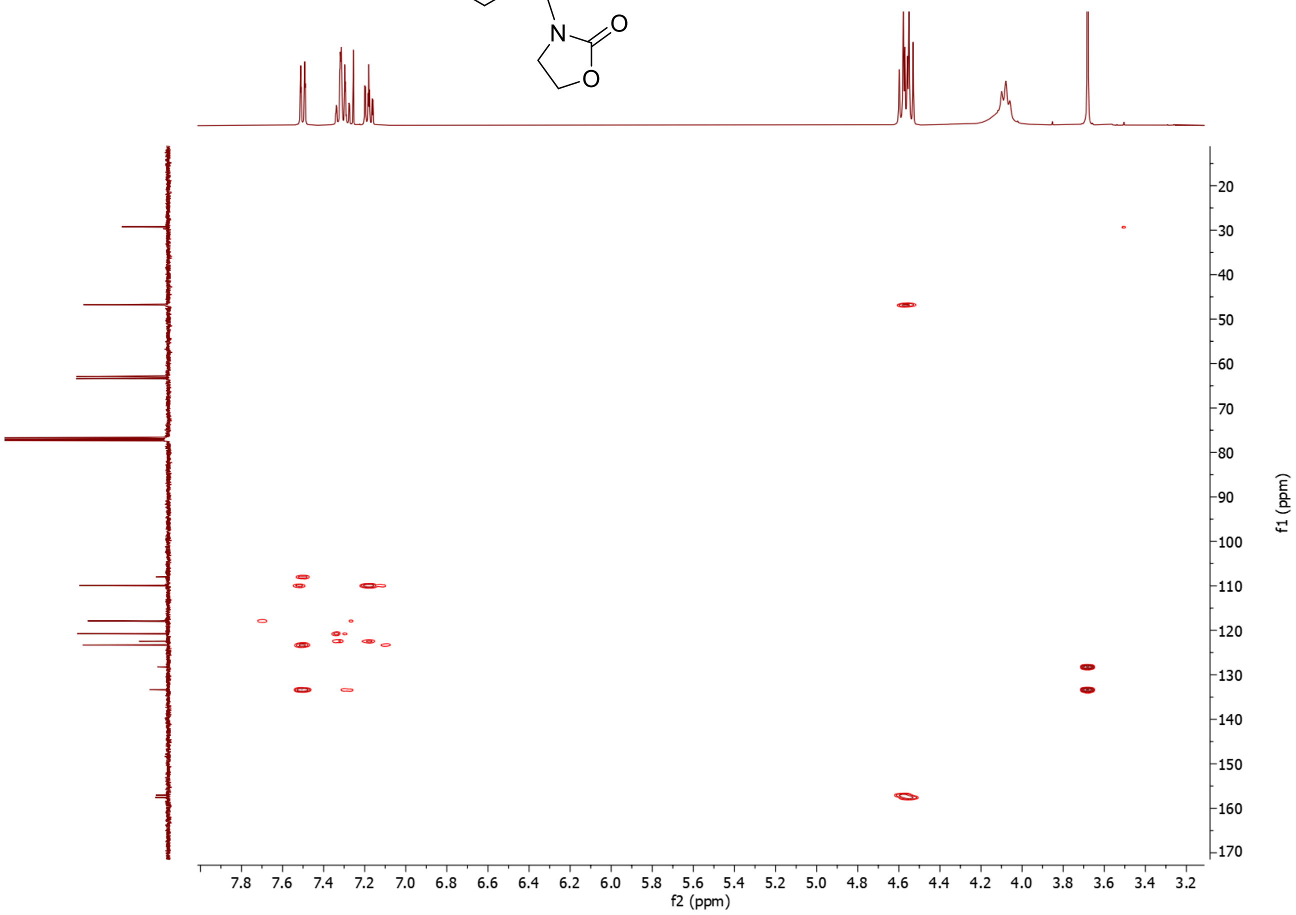

20
30
40
50
60
70
80
90
100
110
120
130
140
150
160
170
f1 (ppm)
7.8 7.6 7.4 7.2 7.0 6.8 6.6 6.4 6.2 6.0 5.8 5.6 5.4 5.2 5.0 4.8 4.6 4.4 4.2 4.0 3.8 3.6 3.4 3.2
f2 (ppm)

10. References

[1] Huang, H.; Li, X.; Yu, C.; Zhang, Y.; Mariano, P. S.; Wang, W. Visible-Light-Promoted Nickel- and Organic-Dye-Cocatalyzed Formylation Reaction of Aryl Halides and Triflates and Vinyl Bromides with Diethoxyacetic Acid as a Formyl Equivalent. *Angew. Chem. Int. Ed.* **2017**, *56*, 1500 –1505.

[2] Guo, M.; Zheng, Y.; Starks, R.; Opoku-Temeng, C.; Ma, X.; Sintim, H. O. 3-AminooxazolidinoneAHL analogs as hydrolytically-stable quorum sensingagonists in Gram-negative bacteria *Med. Chem. Commun.*, **2015**, *6*, 1086–1092.

[3] Balaban, A. T.; Boulton, A. J. *Organic Syntheses*, **1973**, *5*, 1112; *Organic Syntheses* **1969**, *49*, 121.

[4] Greulich, T. W.; Daniliuc, C. G.; Studer, A. N-Aminopyridinium Salts as Precursors for N-Centered Radicals - Direct Amidation of Arenes and Heteroarenes. *Org. Lett.*, **2015**, *17*, 254–257.

[5] Balaban, A. T.; Boulton, A. J. *Organic Syntheses*, **1973**, *5*, 1114.

[6] Boselli, M. F.; Ghosh, I.; Intini, N.; Fattalini, M.; Puglisi, A.; König, B.; Benaglia, M. Visible-Light Photoredox Catalytic Direct N-(Het)Arylation of Lactams. *Chem. Eur. J.* **2025**, *31*, e202404385.

[7] Tower, S. J.; Hetcher, W. J.; Myers, T. E.; Kuehl, N. J.; Taylor, M. T. Selective Modification of Tryptophan Residues in Peptides and Proteins Using a Biomimetic Electron Transfer Process. *J. Am. Chem. Soc.* **2020**, *142*, 9112−9118.

[8] CrysAlisPro, Version 1.171.43.143; Rigaku Oxford Diffraction: Yarnton, Oxfordshire, **2024**.

[9] Sheldrick, G. M. SHELXT – Integrated Space-Group and Crystal-Structure Determination. *Acta Crystallogr.*, Sect. A **2015**, *71*, 3–8.

[10] Sheldrick, G. M. Crystal Structure Refinement with SHELXL. *Acta Crystallogr.*, Sect. C **2015**, *71*, 3–8.

[11] Dolomanov, O. V.; Bourhis, L. J.; Gildea, R. J.; Howard, J. A. K.; Puschmann, H. OLEX2: A Complete Structure Solution, Refinement and Analysis Program. J. *Appl. Crystallogr.* **2009**, *42*, 339–341.

[12] Tiana, W.; Li, B.; Tiana, D.; Tang, W. Regioselective 2-alkylation of indoles with *α*-bromo esters catalyzed by Pd/P,P=O system. *Chinese Chemical Letters* **2022**, *33*, 197–200.

[13] Ding, T.-H.; Qu, J.-P.; Kang, Y.-B. Visible-Light-Induced, Base-Promoted Transition-Metal-Free Dehalogenation of Aryl Fluorides, Chlorides, Bromides, and Iodides. *Org. Lett.* **2020**, *22*, 3084–3088.

[14] Li, Y.; Jin, J.; Fan, W.; Huang, D. π-Extension of Indoles Using Acrolein Linker: Synthesis of Indolo[3,2-a]carbazole-6-carbaldehydes and Racemosin B. *Org. Lett.*, **2023**, *25*, 8284–8289.

[15] Yan, F.; Bai, J.-F.; Dong, Y.; Liu, S.; Li, C.; Du, C.-X.; Li, Y. Catalytic Cyanation of C-N Bonds with CO_2/NH_3. *JACS Au* **2022**, *2*, 2522−2528.

[16] Liu, L.; Zhang, Y.; Zhao, W.; Li, J. Electron Donor-Acceptor Complex induced Fused Indoles with Hypervalent Iodine(III) Reagents. *Org. Lett.* **2023**, *25*, 6251−6255.

[17] Xua, S.; Huanga, X.; Honga, X; Xu, B. Palladium-Assisted Regioselective C–H Cyanation of Heteroarenes Using Isonitrile as Cyanide Source. *Org. Lett.,* **2012**, *14*, 4614–4617.

[18] Donabauer, K.; Murugesan, K.; Rozman, U.; Crespi, S.; König, B. Photocatalytic Reductive Radical-Polar Crossover for a Base-Free Corey–Seebach Reaction. *Chem. Eur. J.* **2020**, *26*, 12945 – 12950.

[19] Lakowicz, J. R. Principles of fluorescence spectroscopy, Springer, NewYork, NY, third edition, **2010**.
